# Strong and Confined Acids Control Five Stereogenic Centers in Catalytic Asymmetric Diels–Alder Reactions of Cyclohexadienones with Cyclopentadiene

**DOI:** 10.1002/anie.202000307

**Published:** 2020-03-11

**Authors:** Santanu Ghosh, Sayantani Das, Chandra Kanta De, Diana Yepes, Frank Neese, Giovanni Bistoni, Markus Leutzsch, Benjamin List

**Affiliations:** ^1^ Max-Planck-Institut für Kohlenforschung Kaiser-Wilhelm-Platz 1 45470 Mülheim an der Ruhr Germany

**Keywords:** Brønsted acids, cyclohexadienones, Diels–Alder reaction, imidodiphosphorimidates, organocatalysis

## Abstract

We describe a highly enantioselective Diels–Alder reaction of cross‐conjugated cyclohexadienones with cyclopentadiene, in which five stereocenters are effectively controlled by a strongly acidic and confined imidodiphosphorimidate catalyst. Our approach provides tricyclic products in excellent stereoselectivity. We also report methods to convert the obtained products into useful intermediates and a computational study that aids in gaining deeper insight into the reaction mechanism and origin of stereoselectivity.

## Graphical abstract

**A highly enantio‐ and diastereoselective** Diels–Alder reaction of cross‐conjugated dienones containing electronically unbiased quaternary centers with cyclopentadiene has been developed. The chiral microenvironment and greater acidity of the imidodiphosphorimidate (IDPi) Brønsted acid motif control the desired outcome. The resulting cycloaddition adducts contain up to five stereogenic centers.

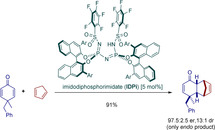

The Diels–Alder reaction is widely appreciated as one of the most powerful methods in chemical synthesis not only for its operational simplicity but also for the construction of molecular complexity and several contiguous stereocenters in a single step.^1^, ^2^ Despite the remarkable progress in advancing catalytic asymmetric variants of the Diels–Alder reaction,^3^, ^4^, ^5^ the use of cross‐conjugated 4,4‐dialkyl‐substituted cyclohexadienones as dienophiles, even with their high synthetic potential, is still extremely rare (Figure 1). The lack of general methods that address this problem may be due to the difficulty in controlling the complex stereochemistry and the reduced dienophilicity of the reacting olefin, situated adjacent to a quaternary center. Notably, such a desymmetrization approach would furnish decalin derivatives with up to five contiguous stereocenters, which are potentially useful building blocks for the synthesis of complex natural products and pharmaceuticals.^6^ In principle, reactions that create five new stereogenic elements can lead to 32 different stereoisomers. However, in Diels–Alder reactions, this number is normally reduced to eight because of the pericyclic [π^4^s+π^2^s] suprafaciality and the stereospecificity of the reaction. Furthermore, *endo* products are generally preferred, reducing the number of possible isomers to four, two pairs of enantiomers.

**Figure 1:**
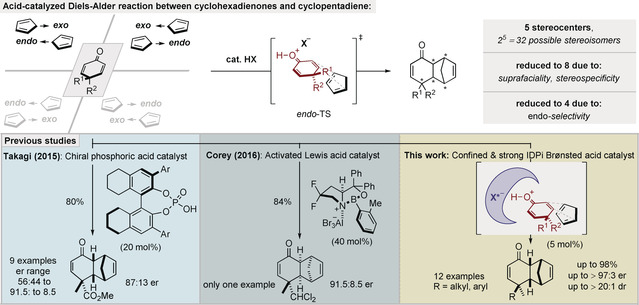
Outline of this study.

Previous catalytic approaches towards challenging cyclohexadienone Diels–Alder reactions have been reported by Takagi and co‐workers using a chiral phosphoric acid (CPA) catalyst and by Corey and co‐workers, who reported a single example of using a highly activated chiral oxazaborolidine Lewis acid catalyst.^7^ However, both of these studies required electronically biased dienones, high catalyst loadings (20–40 mol %), and gave only moderate enantioselectivities. We now show that strong and confined imidodiphosphorimidates (IDPi) enable a broadly useful and general catalytic Diels–Alder reaction of cross‐conjugated, 4,4‐dialkyl‐substituted cyclohexadienones with cyclopentadiene.

Encouraged by our more recent studies on catalytic asymmetric [4+2] cycloadditions, we envisioned that in order to improve the reactivity and selectivity of the targeted reaction, a) a significant LUMO lowering of the dienone would be enabled by a strongly acidic catalyst, and b) a catalyst with a confined active site would be required to control the complex stereochemical reaction outcome. We have recently disclosed novel and unique acid catalysts, including imidodiphosphates (IDP), iminoimidodiphosphates (iIDP), and imidodiphosphorimidates (IDPi) that display enzyme‐like, highly confined active sites and cover a broad range of acidities, approaching superacidic p*K*_a_ values.^8^ Given our recent success in applying such confined acids in both Brønsted and Lewis acid catalysis in various challenging asymmetric carbon–carbon^9^ and carbon–heteroatom^10^ bond‐forming reactions, including diverse enantioselective Diels–Alder reactions and other [4+2] cycloadditions,3c, 11 we hypothesized that our acids might also provide a suitable catalyst platform for the cyclohexadienone Diels–Alder reactions under study here.

At the onset of our studies we chose 4,4‐ethyl‐methyl‐cyclohexadienone (**1 a**) as a challenging model dienophile to react with cyclopentadiene in the presence of different chiral Brønsted acid catalysts, including CPA **3 a**, disulfonimide (DSI) **4 a**, IDP **5 a**, and IDPi catalyst **6 a** (Table 1). While catalysts **3 a**, **4 a**, and **5 a** gave only traces of conversion under the reaction conditions (toluene, −20 °C, 24 h; Table 1, entries 1–3), IDPi catalyst **6 a** gave full conversion and furnished the desired product in promising stereoselectivity and with complete *endo* selectivity (entry 4). To further improve the selectivity of our reaction, we set out to fine‐tune the active site of the catalyst. First, we varied the aryl substituents at the 3,3′‐positions of the binaphthyl backbone (catalysts **6 b** and **6 c**; entries 5 and 6). Indeed, a beneficial effect on the enantiomeric ratio of the major diastereomer was observed upon incorporating the 3‐Ph‐C_6_H_4_ substituent in catalyst **6 c**. Subsequent effort to modify the inner core of the catalyst led to the replacement of the CF_3_SO_2_ substituent on the nitrogen atom of catalyst **6 c** by a C_6_F_5_SO_2_ group in catalyst **6 d**, and, as a consequence, led to significantly enhanced enantiomeric ratios for both two diastereomers (entry 7). Gratifyingly, decreasing the temperature from −20 °C to −80 °C with this catalyst improved both the diastereoselectivity and the enantioselectivity (entry 8). Further lowering of the reaction temperature to −95 °C led to significantly diminished reactivity (entry 9). Hence, the reaction conditions shown in entry 8 were chosen for further investigations.

**Table 1:** Reaction development.^[a]^ 
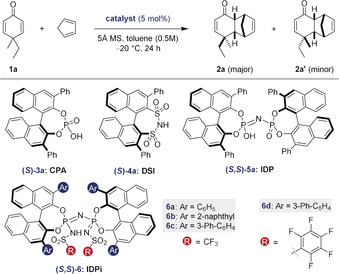

Entry	Catalyst	Conv. [%]^[b]^	dr^[b]^	er^[b]^ (**2 a**)	er^[b]^ (**2 a′**)
1	**3 a**	trace	–	–	–
2	**4 a**	trace	–	–	–
3	**5 a**	trace	–	–	–
4	**6 a**	100	3:1	65:35	74:26
5	**6 b**	65	3:1	49:51	47:53
6	**6 c**	100	3:1	67:33	71:29
7	**6 d**	100	4:1	82:18	84:16
8^[c]^	**6 d**	100	5:1	92:8	94:6
9^[d]^	**6 d**	38	5:1	93.5:6.5	96:4

[a] 0.05 mmol scale. The product formed exclusively (>99:1) from the *endo* approach as determined by NMR analysis of the products (see the Supporting Information for details). [b] The conversion and dr values were determined by ^1^H NMR analysis of the crude reaction mixture. The er values were determined by HPLC analysis on a chiral stationary phase. [c] Reaction at −80 °C for 4 d. [d] Reaction at −95 °C for 4 d.

The scope of the reaction was explored under these optimized reaction conditions (Table 2). The reaction tolerates a variety of dienones (**1**) that contain different aliphatic, allylic, or aromatic substituents next to the methyl group in the 4‐position. Generally, the cycloaddition adducts were obtained with good to excellent yields, enantioselectivities, and diastereoselectivities. As expected, increasingly sterically demanding and branched 4‐substituents such as isopropyl in dienone **1 d** led to significantly higher diastereoselectivity (>20:1 dr). The enantioselectivities remained high to excellent in essentially all studied cases. Notably, in comparison to earlier studies,7a functionalized substrates **1 j**, **1 k**, and **1 l** furnished the cycloaddition products with excellent er of up to 96:4 and diastereoselectivities of up to >20:1, demonstrating a significant improvement. Additionally, dienones **1 i**–**1 l** gave the other diastereomer **2′** (Tables S4–S6 in the Supporting Information). The absolute stereochemistry of Diels–Alder product **2 g** was determined by NMR spectroscopy of Mosher ester derivatives (see the Supporting Information); the relative configuration of products **2 a**–**h** was assigned accordingly. In addition, the absolute configuration of ketone **2 b** was assigned by derivatization (Figure 2) to a known product (see below). The absolute configuration of product **2 l′** was confirmed by an NMR study and by comparing the specific rotation with a literature report;7a the relative configurations of **2 i′**, **2 j′**, and **2 k′** were assigned by analogy.

**Figure 2:**
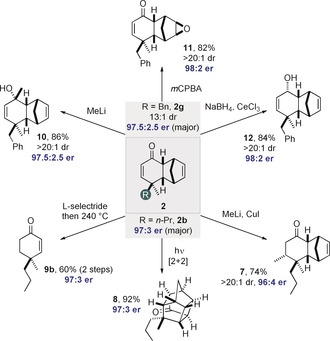
Derivatizations of Diels–Alder products (see the Supporting Information for details).

**Table 2:** Scope of the reaction.^[a,b]^ 
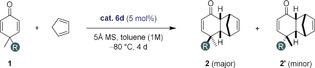

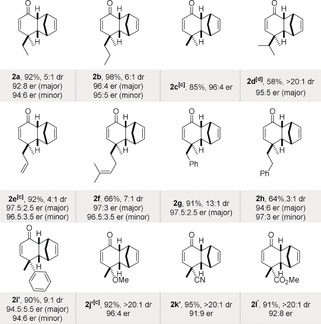

[a] Reactions on 0.2 mmol scale; the er values were determined by HPLC analysis on a chiral stationary phase (see the Supporting Information). [b] The dr values were determined by ^1^H NMR analysis after isolation of the desired product. [c] Reaction performed at −95 °C. [d] Reaction time 8 d.

In order to gain deeper insight into the reaction mechanism and to understand the origin of enantio‐ and diastereoselectivity of the process, we performed computational modeling using dienones **1 a** and **1 j**, cyclopentadiene, and catalyst (*S*,*S*)**‐6 d**. Mechanistic studies were carried out at the M06‐2X/def2‐TZVP+C‐PCM‐(toluene)//PBE‐D3(BJ)/def2‐SVP level of theory.^12^ For transition states, a thorough conformational sampling^13^ was performed (177 structures were optimized at the PBE‐D3 level), and single‐point energies were refined using high‐level domain‐based local pair natural orbital coupled cluster calculations (DLPNO‐CCSD(T)/def2‐TZVP; see the Supporting Information for details). Substrate activation is an exergonic process (Δ*G*=−11.5 kcal mol^−1^) that occurs immediately by protonation of dienone **1 a**. The resulting cationic product forms a strongly directional hydrogen bond with an oxygen atom of the ‐SO_2_C_6_F_5_ group of the counteranion (**CIP‐1 a** in Figure 3). The strong catalyst interaction in **CIP‐1 a** renders C‐4 stereogenic, and thus the computed early diastereoselectivity of 3.7:1 is in good agreement with the experimental value of 5:1. Afterwards, **CIP‐1 a** forms a reactant complex (**RC‐1 a**) with cyclopentadiene, which then leads to product complex **P‐2 a** by an *endo* attack. In the stereodetermining transition state **TS‐2 a**, cyclopentadiene interacts with the catalyst through a network of non‐conventional C−H⋅⋅⋅O and C−H⋅⋅⋅N hydrogen bonds.^14^ Finally, product **2 a** is released upon exchange with **1 a**, thus forming **CIP‐1 a** and concomitantly restoring the catalytic cycle (Figure 3 B).

**Figure 3:**
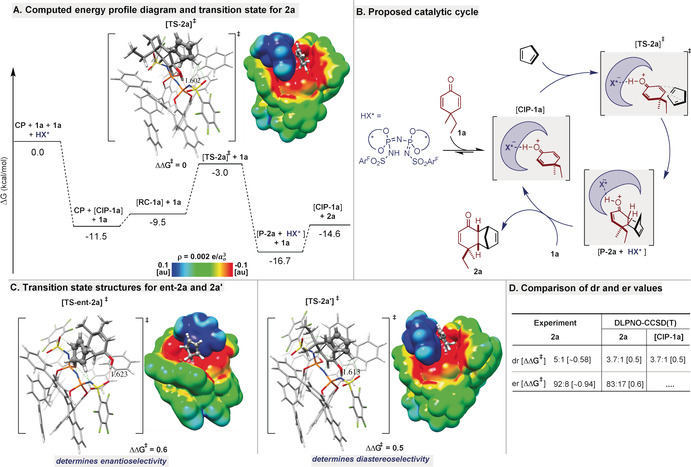
Computational studies. A) Proposed reaction mechanism and TS structures of **2 a**. B) Proposed catalytic cycle. C) Enantiomeric and diastereomeric TS structures with the corresponding molecular electrostatic potentials (see the Supporting Information for details). D) Experimental and computational dr and er values together with ΔΔ*G* ^≠^ values at −80 °C (in kcal mol^−1^). CIP and TS single‐point energies at the DLPNO‐CCSD(T)/def2‐TZVP+C‐PCM(toluene)//PBE‐D3/def2‐SVP level of theory.

It is noteworthy that the hydrogen bond established as the early molecular recognition between the activated substrate and the counteranion is conserved along the entire reaction path. It is also worth emphasizing that the R and Me substituents of **1** are oriented away from the chiral pocket of **6 d**, which is consistent with the high stereoselectivities obtained with different R groups (Table 2). Accurate dr and er values (Figure 3 D) were calculated as the relative free energies of the corresponding TSs (according to the Curtin–Hammett principle) at the DLPNO‐CCSD(T)/def2‐TZVP level of theory.^15^ For **2 a**, the experimentally determined 5:1 dr (ΔΔ*G* ^≠^=0.58 kcal mol^−1^) and 92:8 er (ΔΔ*G* ^≠^=0.94 kcal mol^−1^) are in good agreement with the computed dr and er values of 3.7:1 (ΔΔ*G* ^≠^=0.5 kcal mol^−1^) and 83:17 (ΔΔ*G* ^≠^=0.6 kcal mol^−1^), respectively.

To shed light on the physical factors responsible for the diastereoselectivity in the formation of **2 a** (5:1 dr), especially in comparison to the methoxy derivative **2 j′** (>20:1 dr), we conducted a local energy decomposition analysis (LED)^16^ of the DLPNO‐CCSD(T) interaction energy between the CIP and cyclopentadiene at the TS geometries. For **2 a**, the calculated energies indicate that the trajectory of cyclopentadiene is mainly controlled by steric factors, leading to favorable attack from the less congested face, which corresponds to that with the small group closer to the π‐system of cyclopentadiene. On the contrary, the inversion of the stereogenic center at C‐4 for **2 j′** is evident from the stability of the **TS‐2 j′** over **TS‐2 j** (ΔΔ*G* ^≠^=3.2 kcal mol^−1^; see the Supporting Information for details). This preference arises from the lower steric repulsion between the π‐system of cyclopentadiene and the methoxy oxygen atom ($\Delta E_{\mathrm{el}-\mathrm{prep}}^{\mathrm{HF}}$ (**TS‐2 j**′)=362.7 kcal mol^−1^) compared to that induced by the methylene group ($\Delta E_{\mathrm{el}-\mathrm{prep}}^{\mathrm{HF}}$ (**TS‐2 j**)=369.7 kcal mol^−1^; see the Supporting Information for more details).

To illustrate the synthetic utility of our method several highly stereoselective derivatizations of products **2 b** and **2 g** were performed without loss of enantiopurity of the functionalized products (Figure 2). For example, a conjugate addition of an in situ generated cuprate to **2 b** proceeded with an excellent dr of >20:1, giving highly enantioenriched product **7**. A [2+2] photocycloaddition reaction of enone **2 b** furnished product **8** in excellent yield, without any deterioration of enantiopurity. Along the same lines, a potentially attractive route to alkyl‐substituted Robinson‐annulation‐type products such as enone **9 b** was also developed. Accordingly, a conjugate reduction of enone **2 b** with l‐selectride followed by a thermal retro‐Diels–Alder cycloaddition provided the desired ketone **9 b** in good yield and with preservation of enantiopurity. The absolute configuration of **9 b** was confirmed by comparing its specific rotation with a literature value (see the Supporting Information).

We also developed a 1,2‐addition of methyllithium to enone **2 g**, giving the corresponding tertiary alcohol **10** in excellent yield and diastereoselectivity (>20:1). Epoxidation of the electron‐rich olefinic double bond of enone **2 g** was performed using *m*CPBA as the oxidant and gave epoxide **11** (>20:1 dr) in 82 % yield. A carbonyl reduction of enone **2 g** under Luche conditions proceeded with high diastereoselectivity (>20:1 dr) and provided the corresponding enantioenriched alcohol **12**.

In summary, we have developed an efficient Brønsted acid catalyzed asymmetric intermolecular Diels–Alder reaction of cross‐conjugated dienones containing electronically unbiased quaternary centers with cyclopentadiene that furnishes previously inaccessible products. The high acidity and confined structure of our IDPi catalyst directs the cycloaddition and provides the corresponding adducts with up to five stereocenters in excellent yields, enantio‐, and diastereoselectivities. Further studies of our IDPi catalysts as privileged motifs for Diels–Alder reactions are ongoing.

## Conflict of interest

The authors declare no conflict of interest.

## Supporting information

As a service to our authors and readers, this journal provides supporting information supplied by the authors. Such materials are peer reviewed and may be re‐organized for online delivery, but are not copy‐edited or typeset. Technical support issues arising from supporting information (other than missing files) should be addressed to the authors.

Supplementary
