# Supplementary material for: Strong and Confined Acids Control Five Stereogenic Centers in Catalytic Asymmetric Diels–Alder Reactions of Cyclohexadienones with Cyclopentadiene
Source: Angew Chem Int Ed Engl. 2020 Mar 11;59(30):12347–51. doi: 10.1002/anie.202000307 (PMC7383742; doi:10.1002/anie.202000307)
Supplement: Supplementary file 1 — Supplementary [file ANIE-59-12347-s001.pdf]

Angewandte Chemie
Eine Zeitschrift der Gesellschaft Deutscher Chemiker

Supporting Information

Strong and Confined Acids Control Five Stereogenic Centers in Catalytic Asymmetric Diels–Alder Reactions of Cyclohexadienones with Cyclopentadiene

*Santanu Ghosh, Sayantani Das, Chandra Kanta De, Diana Yepes, Frank Neese, Giovanni Bistoni, Markus Leutzsch, and Benjamin List**

anie_202000307_sm_miscellaneous_information.pdf

Supporting Information

Table of Contents

Table of Contents	Page No.
General Information	2
Synthesis and Characterization of Catalysts	3-5
Synthesis and Characterization of Cyclohexenones	5-6
Synthesis and Characterization of Cyclohexedienones	7-8
Reaction Development	8-10
Characterization of Products	10-23
Functionalization of Products	23-29
Absolute Stereochemistry Assignment	30-35
Computational Studies	35-41
References and Notes	41-42
Spectral Data	43-95
HPLC and GC Traces	96-124

General Information

Unless otherwise stated, all reagents were purchased from commercial suppliers and used without further purification. Solvents were distilled using appropriate drying agents and kept under argon. Thin layer chromatography (TLC) on silica gel pre-coated plastic sheets (0.2 mm, Macherey-Nagel) or glass plates (SIL G-25 UV254, 0.25 mm, (Macherey-Nagel) were used to monitor the progress of the reactions and visualized by UV light at 254 nm, and/or phosphomolybdic acid (PMA) stain, and/or anisaldehyde stain, and/or parmanganate stain. SIL G-25 UV254, Alox G-25 UV254 with 0.25 mm SiO_2 or Al_2O_3 layer coated glass plates were used for the preparative thin-layer chromatography (PrepTLC). Merck silica gel (60, particle size 0.040–0.063 mm) was used for the column chromatography. Nuclear Magnetic Resonance (NMR) spectra of ^{1}H, ^{13}C, ^{19}F, ^{31}P nucleus were recorded on Bruker AV-600, AV-500 spectrometer in $CDCl_3$ at 25 °C unless otherwise stated. The resonance multiplicity is reported as s (singlet), d (doublet), t (triplet), q (quadruplet), p (pentet), hept (heptet), m (multiplet), and br (broad) and coupling constant are reported in Hz. The reported data were processed with Bruker TOPSPIN or MestReNova suits of programs. The residual $CDCl_3$ signal relative to tetramethylsilane (TMS) was used as internal reference for the NMR signal e.g. $CDCl_3$ = 7.26 ppm for ^{1}H, 77.16 ppm for ^{13}C. The Bruker APEX III FTMS (7 T magnet) was used to record the high resolution mass spectrum. Specific rotation was recorded by Autopol IV polarimeter (Rudolph Research Analytical) at 589 nm and 20 °C, concentration (*c*; g/100 mL), and $CHCl_3$ was used as solvent. ^{1}H NMR spectra of the crude reaction mixtures, or HPLC analysis employing a chiral stationary phase were utilized to determine the diastereomeric ratios (dr). Enantiomeric excess (ee) or ratios (er) were determined by GC or HPLC analysis employing a chiral stationary phase column indicated in the individual experiment, with the comparison of corresponding racemic sample. All isolated yields were reported, unless otherwise specified. Compounds (**9a**, **9i**, **1a**, **1i**),[2a] **9b**,[2b] **9c**,[2c] **9d**,[2d] **9e**,[2e] **1e**,[2f] (**9f**, **1f**),[3] **1c**,[4] (**1j**, **1k**, **1l**),[5] **3**,[6] **4**,[7] **5**,[8] **6**,[9a-b] **14**,[9c] are synthesized according to known or modified literature procedure and characterization was confirmed in comparison with the literature data.

1,3-Cyclopentadiene was prepared from dicyclopentadiene *via* pyrolysis at 160 °C[1] and was stored at –78 °C in a Schlenk flask under argon.

Synthesis and Characterization of Catalysts

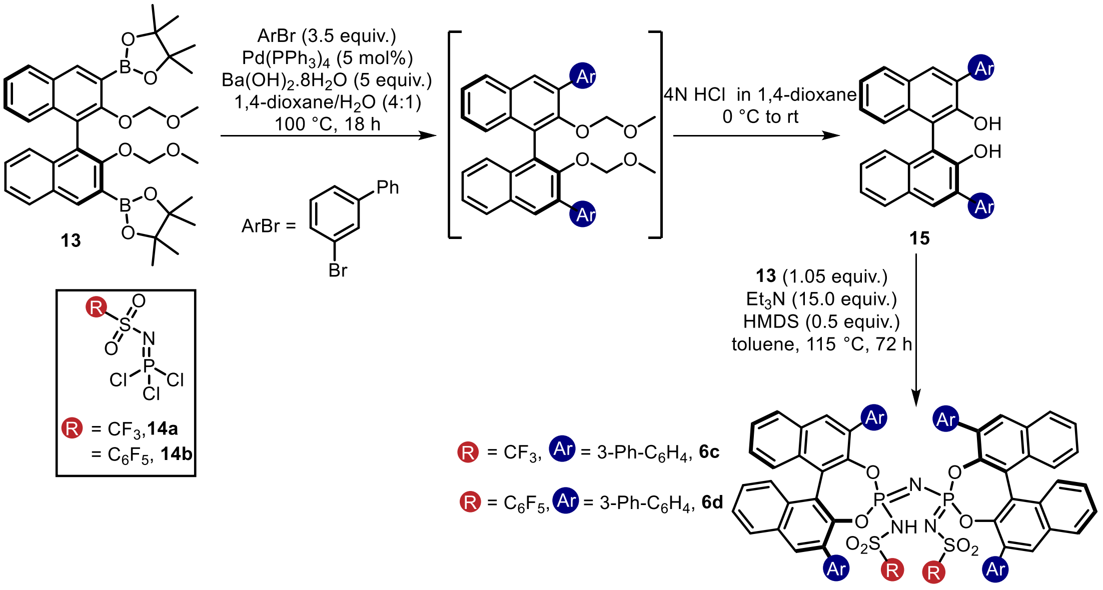

Synthesis of (*S*)-3,3'-di([1,1'-biphenyl]-3-yl)-[1,1'-binaphthalene]-2,2'-diol 15: A Schlenk flask was charged with MOM-protected (*S*)-BINOL-boronate ester (**13**) (1.0 equiv., 3.99 mmol, 2.5 g), ArBr (3.5 equiv., 13.97 mol, 3.26 g) and $Ba(OH)_2 \cdot 8H_2O$ (4.0 equiv., 15.97 mmol, 3.02 g) in 1,4-dioxane/H_2O (4:1) (25 mL). Then the reaction mixture was cooled to –78 °C and the system was degassed under argon (3 times). The reaction flask was warmed up to rt and $Pd(PPh_3)_4$ (0.05 equiv., 0.2 mmol, 0.23 g) was added and subsequently the reaction flask was sealed and placed over a 100 °C oil bath and was stirred for 16 h. The reaction flask was cooled to rt, diluted with Et_2O and H_2O. The organic layer was separated, washed with brine, dried over anhydrous Na_2SO_4, and concentrated under reduced pressure to afford the crude material. The crude material was dissolved in THF and placed over a 0 °C ice bath and 4N HCl in 1,4-dioxane (10 mL) was added and the reaction mixture was stirred for 5 h. After that the reaction mixture was evaporated and purified by column chromatography using 10% EtOAc in hexane as eluent, and afforded the enantiopure diol (2.1 g, 89% over 2 steps). **^{1}H NMR** (501 MHz, $CDCl_3$) δ 8.11 (s, 1H), 7.99 (t, *J* = 1.7 Hz, 1H), 7.97–7.93

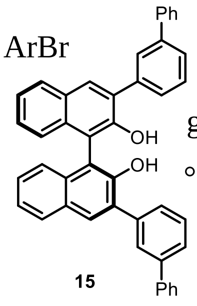

15

(m, 1H), 7.75 (dt, J = 7.6, 1.4 Hz, 1H), 7.71–7.63 (m, 3H), 7.58 (t, J = 7.7 Hz, 1H), 7.47 (t, J = 7.7 Hz, 2H), 7.42 (ddd, J = 8.0, 6.8, 1.2 Hz, 1H), 7.40–7.31 (m, 2H), 7.30–7.23 (m, 1H), 5.45 (d, J = 1.5 Hz, 1H); **^{13}C NMR** (126 MHz, $CDCl_3$) δ 150.3, 141.7, 141.2, 138.1, 133.2, 131.6, 130.7, 129.6, 129.0, 128.9, 128.7, 128.6, 127.6, 127.5, 127.5, 126.7, 124.6, 124.4, 112.6; $[\boldsymbol{\alpha}]_{\boldsymbol{D}}^{\boldsymbol{20}}$ = –33.50 (c 0.39, $CHCl_3$).

N-((11bS)-2,6-di([1,1'-biphenyl]-3-yl)-4-(((11bS)-2,6-di([1,1'-biphenyl]-3-yl)-4-(((trifluoromethyl)sulfonyl)imino)-4l5-dinaphtho[2,1-d:1',2'-f][1,3,2]dioxaphosphepin-4-yl)imino)-4l5-dinaphtho[2,1-d:1',2'-f][1,3,2]dioxaphosphepin-4-yl)-1,1,1-trifluoromethanesulfonamide 6c: Compound was prepared according to known literature procedure[9c] in (5 mmol) scale. The crude catalyst was purified by column chromatography using 40-70% Et_2O in pentane to as eluent and obtained to afford the desired salt. The corresponding Brønsted acid (S,S)**-6c** was obtained after acidification with aq. HCl (6M) in CH_2Cl_2 and the organic layer was separated and dried under high vacuum as off-white solid in 69% yield. **^{1}H NMR** (501 MHz, $CDCl_3$) δ 8.07 (s, 1H), 7.93 (d, J = 8.2 Hz, 2H), 7.75 (t, J = 7.5 Hz, 1H), 7.67 (br, 1H), 7.54 (m, 2H), 7.52–7.46 (m, 2H), 7.42–7.38 (m, 3H), 7.37–7.32 (m, 2H), 7.32–7.29 (m, 2H), 7.27 (m, 2H), 7.25–7.16 (m, 4H), 7.16–7.08 (m, 2H), 6.97–6.90 (m, 3H), 6.15 (d, J = 7.7 Hz, 1H), 5.98 (br, 1H); **^{13}C NMR** (126 MHz, $CDCl_3$) δ 143.8 (m), 142.9 (m), 142.3, 141.4, 141.3, 140.4, 138.0, 136.0, 135.9, 133.7, 133.3, 132.2, 132.2, 132.0, 131.8, 131.2, 129.2, 129.1, 129.0, 128.8, 128.8, 128.7, 128.5, 128.4, 128.4, 128.3, 128.2, 127.6, 127.5, 127.4, 127.3, 127.1, 127.0, 126.9, 126.9, 126.6, 125.4, 123.6, 121.8, 120.2, 117.6; **^{19}F NMR** (471 MHz, $CDCl_3$) δ –78.35; **^{31}P NMR** (203 MHz, $CDCl_3$) δ –16.56; **HRMS** (ESI-neg) m/z calculated for $C_{90}H_{56}N_3O_8P_2S_2F_6$ $[M-H]^-$: 1546.289386; found 1546.289280.

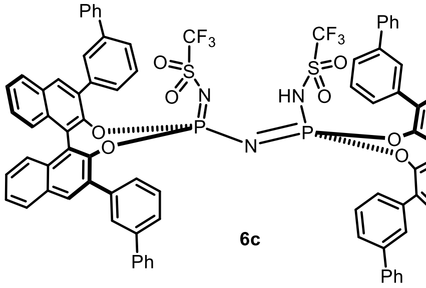

Synthesis of N-((11b*S*)-2,6-di([1,1'-biphenyl]-3-yl)-4-(((11b*S*)-2,6-di([1,1'-biphenyl]-3-yl)-4-(((perfluorophenyl)sulfonyl)imino)-4l5-dinaphtho[2,1-d:1',2'-f][1,3,2]dioxaphosphepin-4-yl)imino)-4l5-dinaphtho[2,1-d:1',2'-f][1,3,2]dioxaphosphepin-4-yl)-2,3,4,5,6-pentafluorobenzenesulfonamide 6d: Compound was prepared according to known literature procedure[9c] in (1.88 mmol) scale. The crude material was purified by column chromatogrphy on silica gel using 10 to 30% EtOAc in hexane as eluents, to afford the desired salt. The corresponding Brønsted acids (*S*,*S*)-**6d** was obtained after acidification with aq. HCl (6M) in CH_2Cl_2 and the organic layer was separated and dried under high vacuum as off-white solid in 45% yield. **^{1}H NMR** (600 MHz, $CDCl_3$) δ 8.06 (s, 1H), 7.96 (d, *J* = 8.3 Hz, 2H), 7.75 (ddd, *J* = 8.0, 6.8, 1.1 Hz, 1H), 7.67 (m, 1H), 7.55–7.49 (m, 4H), 7.47–7.45 (m, 3H), 7.40–7.33 (m, 5H), 7.32–7.29 (m, 3H), 7.26–7.23 (m, 3H), 7.22–7.19 (m, 1H), 7.02–6.98 (m, 2H), 6.82 (t, *J* = 7.7 Hz, 1H), 6.34 (m, 1H), 4.76 (s, 1H); **^{13}C NMR** (151 MHz, $CDCl_3$) δ 143.8 (dd, *J* = 261.6, 10.8 Hz), 143.7 (t, *J* = 5.1 Hz), 143.2 (dt, *J* = 261.1, 10.8 Hz), 143.1 (t, *J* = 5.3 Hz), 141.3, 140.9, 140.6, 140.5, 137.0 (dt, *J* = 253.3, 13.0 Hz), 136.1, 135.8, 133.5, 133.3 (t, *J* = 1.8 Hz), 131.8, 131.76, 131.7, 131.6, 131.4, 131.2, 129.1, 128.9, 128.8, 128.7, 128.4, 128.2, 128.1, 127.9, 127.6, 127.3, 127.2, 127.19, 127.1, 126.9, 126.8, 126.78, 126.6, 126.57, 126.5, 126.4, 117.2 (t, *J* = 10.8 Hz); **^{19}F NMR** (471 MHz, $CDCl_3$) δ –135.81 (d, *J* = 22.0 Hz), –146.01, –159.33 (m).; **^{31}P NMR** (203 MHz, $CDCl_3$) δ –14.06; **HRMS** (ESI-neg) *m/z* calculated for $C_{100}H_{56}F_{10}N_3O_8P_2S_2$ $[M-H]^-$: 1742.283000; found 1742.284050.

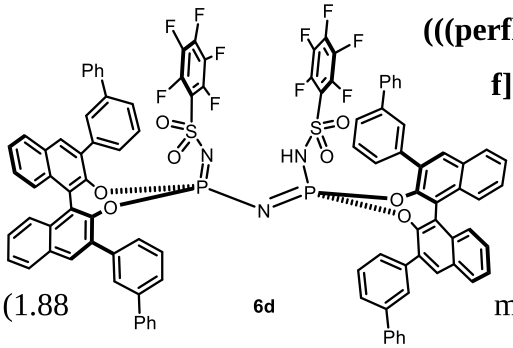

Synthesis and Characterization of Cyclohexenones

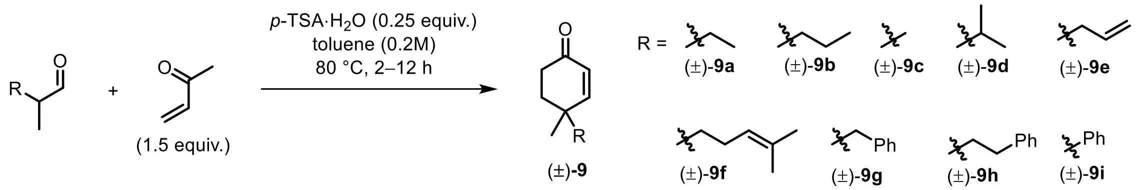

Following a literature reported procedure[2a] a flame-dried Schlenk flask equipped with a magnetic stirring bar was charged with aldehyde (1 equiv.) and methyl vinyl ketone (1.5 equiv.) in

toluene (0.2M) at rt. To that solution was added *p*-TSA·H_2O (0.25 equiv.) and the reaction mixture was heated to 80 °C for 2 to 12 h. Upon completion (monitored by TLC analysis) the reaction mixture was cooled to rt, diluted with Et_2O, and treated with saturated aqueous $NaHCO_3$ solution. The organic layer was separated and washed with brine (100 mL), dried over anhydrous $MgSO_4$ and concentrated under reduced pressure to obtain the crude product. The crude material was purified by column chromatography using 7–10% EtOAc in hexane as eluent to afford the desired enone (±)-**9**.

4-benzyl-4-methylcyclohex-2-en-1-one (±)-**9g**: Prepared according to the representative

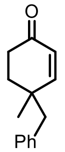

(±)-**9g**

procedure with aldehyde (1.0 equiv., 24.97 mmol, 3.7 g) and methyl vinyl ketone (1.5 equiv., 37.45 mmol, 3.03 mL), *p*-TSA·H_2O (0.25 equiv., 6.24 mmol, 1.19 g), at 80 °C for 12 h. The product was purified by column chromatography using 7% EtOAc in hexane as eluent and obtained as yellow solid (2.8 g, 56%). ^{1}**H NMR** (501 MHz, $CDCl_3$) δ 7.32–7.28 (m, 2H), 7.27–7.24 (m, 1H), 7.16–7.13 (m, 2H), 6.70 (dt, *J* = 10.2, 0.9 Hz, 1H), 5.91 (d, *J* = 10.2 Hz, 1H), 2.77 (d, *J* = 2.2 Hz, 2H), 2.45 (dd, *J* = 7.4, 6.3 Hz, 2H), 2.01–1.96 (m, 1H), 1.81–1.74 (m, 1H), 1.14 (s, 3H). ^{13}**C NMR** (126 MHz, $CDCl_3$) δ 199.6, 158.5, 137.1, 130.6, 128.3, 127.8, 126.8, 46.9, 36.9, 34.3, 33.9, 25.4; **HRMS** (ESI) *m/z* calculated for $C_{14}H_{16}O$ $[M]^+$: 200.119565; found 200.119710.

4-methyl-4-phenethylcyclohex-2-en-1-one (±)-**9h**: Prepared according to the representative

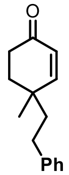

(±)-**9h**

procedure with aldehyde (1.0 equiv., 9.25 mmol, 1.5 g) and methyl vinyl ketone (1.5 equiv., 13.87 mmol, 3.24 mL), *p*-TSA.H_2O (0.5 equiv., 4.62 mmol, 0.88 g), at 70 °C for 12 h. The product was purified by column chromatography using 12% Et_2O in hexane and obtained as yellow liquid. (0.912 g, 46%). ^{1}**H NMR** (501 MHz, $CDCl_3$) δ 7.31–7.27 (m, 2H), 7.22–7.17 (m, 3H), 6.73 (dt, *J* = 10.2, 0.8 Hz, 1H), 5.92 (d, *J* = 10.2 Hz, 1H), 2.70–2.58 (m, 2H), 2.54–2.44 (m, 2H), 2.06 (ddd, *J* = 14.3, 8.8, 5.9 Hz, 1H), 1.85 (dddd, *J* = 14.2, 7.5, 5.9, 1.4 Hz, 1H), 1.81–1.72 (m, 2H), 1.23 (s, 3H); ^{13}**C NMR** (126 MHz, $CDCl_3$) δ 199.6, 158.8, 142.2, 128.7, 128.4, 127.8, 126.2, 43.2, 35.9, 34.3, 33.7, 30.9, 25.0; **HRMS** (ESI) *m/z* calculated for $C_{15}H_{18}O$ $[M]^+$: 214.135215; found 214.135220.

Synthesis and Characterization of Cyclohexedienones

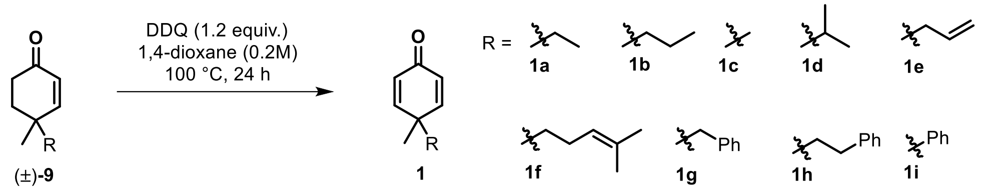

Dienone (**1**) was prepared according to the literature procedure.[2a] Typically a flame-dried Schlenk flask equipped with a teflon coated magnetic stirring bar and fitted with a reflux condenser was charged with cyclohexenone (±)-**9** (1 equiv.) in 1,4-dioxane (0.2M) at rt. Then DDQ (1.2 equiv.) was added to the solution and the reaction flask was placed on a 100 °C preheated oil bath and was stirred for 18 to 24 h (upon completion of the reaction judged by TLC analysis). Then the reaction flask was cooled to rt, diluted with Et_2O and the solid was removed by filtration through a celite frit. The filtrate was quenched with saturated aqueous $NaHCO_3$ solution. The organic layer was separated, dried over anhydrous $MgSO_4$ and concentrated under reduced pressure. The crude mixture was purified by column chromatography using 5–10% EtOAc in hexane as eluent to afford the title compound **1**.

9f, **1e** and **1f** were prepared according to literature procedure[3]

4-methyl-4-propylcyclohexa-2,5-dien-1-one 1b: Prepared according to the general procedure

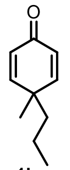

1b

with enone (±)-**9b** (1.0 equiv., 1.97 mmol, 0.3 g) and DDQ (1.3 equiv., 2.56 mmol, 0.581 g), at 100 °C for 18 h. The product was purified by column chromatography using 8% EtOAc in hexane and was obtained as yellow oil. (0.21 g, 71%). **^{1}H NMR** (501 MHz, $CDCl_3$) δ 6.78–6.75 (m, 2H), 6.27–6.23 (m, 2H), 1.60–1.57 (m, 2H), 1.23 (s, 3H), 1.18–1.11 (m, 2H), 0.85 (t, J = 7.3 Hz, 3H); **^{13}C NMR** (126 MHz, $CDCl_3$) δ 186.6, 156.2, 128.8, 43.0, 42.3, 26.2, 18.5, 14.5; **HRMS** (GC-EI) m/z calculated for $C_{10}H_{14}O$ $[M]^+$: 150.103915; found 150.103960.

4-isopropyl-4-methylcyclohexa-2,5-dien-1-one 1d: Prepared according to the general procedure

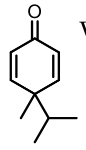

1d

with enone (±)-**9d** (1.0 equiv., 4.6 mmol, 0.7 g) and DDQ (1.2 equiv., 5.52 mmol, 1.25 g), at 100 °C for 24 h. The product was purified by column chromatography using 10% EtOAc in hexane and was obtained as yellow oil. (0.321 g, 46%). **^{1}H NMR** (501 MHz, $CDCl_3$) δ

6.83–6.80 (m, 2H), 6.31–6.27 (m, 2H), 1.83 (hept, *J* = 6.9 Hz, 1H), 1.23 (s, 3H), 0.91 (d, *J* = 6.9 Hz, 6H); **^{13}C NMR** (126 MHz, $CDCl_3$) δ 186.6, 155.1, 129.4, 44.9, 36.9, 24.0, 18.1; **HRMS** (GC-EI) *m/z* calculated for $C_{10}H_{14}O$ $[M]^+$: 150.103915; found 150.103940.

4-benzyl-4-methylcyclohexa-2,5-dien-1-one 1g: Prepared according to the general procedure

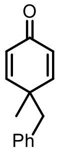
1g

with enone (±)-**9g** (1.0 equiv., 3.25 mmol, 0.650 g) and DDQ (1.2 equiv., 3.89 mmol, 0.884 g), at 100 °C for 24 h. The product was purified by column chromatography using 8 % EtOAc in hexane and was obtained as yellow solid. (0.643 g, 64%). **^{1}H NMR** (501 MHz, $CDCl_3$) δ 7.27–7.20 (m, 3H), 7.08–7.06 (m, 2H), 6.86–6.83 (m, 2H), 6.20–6.17 (m, 2H), 2.87 (s, 2H), 1.29 (s, 3H); **^{13}C NMR** (126 MHz, $CDCl_3$) δ 186.2, 155.2, 136.1, 130.2, 128.6, 128.2, 127.1, 47.0, 42.6, 25.1; **HRMS** (GC-EI) *m/z* calculated for $C_{14}H_{14}O$ $[M]^+$: 198.103915; found 198.104030.

4-methyl-4-phenethylcyclohexa-2,5-dien-1-one 1h: Prepared according to the general procedure

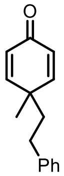
1h

with enone (±)-**9h** (1.0 equiv., 3.73 mmol, 0.8 g) and DDQ (1.2 equiv., 4.48 mmol, 1.01 g), at 100 °C for 24 h. The product was purified by column chromatography using 8% EtOAc in hexane and was obtained as yellow oil (0.531 g, 67%). **^{1}H NMR** (501 MHz, $CDCl_3$) δ 7.28–7.25 (m, 2H), 7.20–7.16 (m, 1H), 7.11–7.09 (m, 2H), 6.84–6.80 (m, 2H), 6.34–6.30 (m, 2H), 2.45–2.41 (m, 2H), 1.96–1.93 (m, 2H), 1.30 (s, 3H); **^{13}C NMR** (126 MHz, $CDCl_3$) δ 186.4, 155.5, 141.5, 129.3, 128.6, 128.3, 126.3, 42.4, 42.2, 31.6, 26.3; **HRMS** (GC-EI) *m/z* calculated for $C_{15}H_{16}O$ $[M]^+$: 212.119565; found 212.119550.

Reaction Development

Table 1. Optimization studies[a],[b]

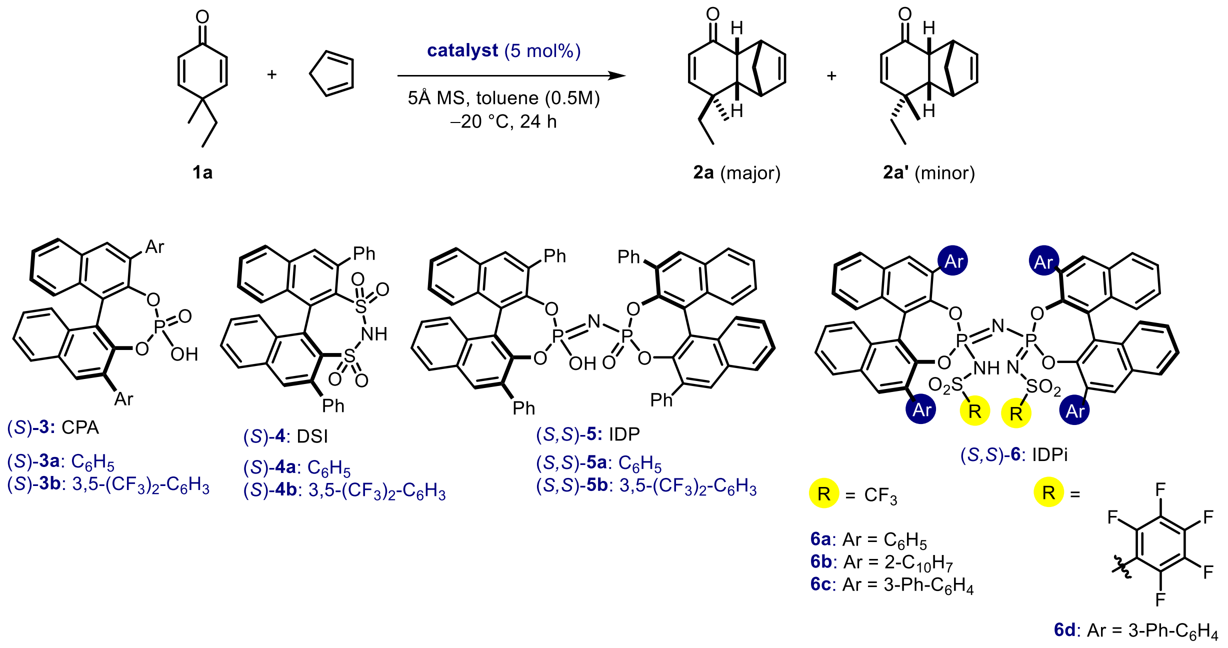

entry	catalyst	solvent	Conv. (%)	dr	er (**2a**)	er (**2a′**)
1	**3a**	toluene	trace	--	--	--
2	**3b**	toluene	trace	--	--	--
3	**4a**	toluene	trace	--	--	--
4	**4b**	toluene	trace	--	--	--
5	**5a**	toluene	trace	--	--	--
6	**5b**	toluene	trace	--	--	--
7	**6a**	toluene	100	3:1	65:35	74:26
8	**6b**	toluene	65	3:1	49:51	47:53
9	**6c**	toluene	100	3:1	67:33	71:29
12	**6d**	toluene	100	4:1	82:18	84:16
13	**6d**	pentane	100	5:1	79:21	79:21
14	**6d**	1,4-dioxane	100	4:1	79:21	81:19
15	**6d**	$CHCl_3$	67	5:1	78:22	80:20
16[c]	**6d**	toluene	100	5:1	92:8	94:6
17[d]	**6d**	toluene	38	5:1	93.5:6.5	96:4

[a]0.05 mmol scale. [b]Conversion. [c]Reaction at −80 °C for 4 d. [d]Reaction at −95 °C for 4 d. and dr were determined by ^{1}H NMR of crude reaction mixture. er determined by HPLC using chiral stationary phase.

General procedure for the catalytic Diels–Alder reaction

A 2 mL oven-dried GC vial was charged with magnetic stir bar, catalyst (5 mol%, 0.05 equiv., 0.01 mmol), 5Å MS (20 mg) and toluene (1M, 200 μL). Then the dienone **1a** (1 equiv., 0.2 mmol) was added to the reaction vial and was cooled to –80 °C or –95 °C.[11] After 10 minutes at this temperature, cyclopentadiene (10 equiv., 2 mmol, 165 μL) was added through the wall of the reaction vial and stirred for the specific reaction time. The reaction was quenched with Et_3N (20 μL). After 10 minutes the reaction mixture was warmed up to rt. The dr was determined by crude ^{1}H NMR. Purification was performed by column chromatography or preparative thin layer chromatography on silica gel using EtOAc/hexane as eluents.

Racemates were synthesized by either using *p*-TSA·H_2O or racemic acid (**6**).

Characterization of Products

(1*R*,4*S*,4a*R*,8*S*,8a*S*)-8-ethyl-8-methyl-4,4a,8,8a-tetrahydro-1,4-methanonaphthalen-5(1*H*)-

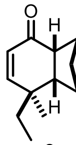

2a

one 2a: Prepared according to the representative procedure. The product was purified by column chromatography using 5 to 8% EtOAc in hexane and was obtained as yellow liquid (92%, dr = 5:1). **^{1}H NMR** (501 MHz, $CDCl_3$, spectra contained dr = 5:1) **for major diastereomer** δ 6.21 (dd, *J* = 10.3, 1.6 Hz, 1H), 6.06 (dd, *J* = 5.7, 2.8 Hz, 1H), 5.84 (d, *J* = 10.3 Hz, 1H), 5.81 (dd, *J* = 5.7, 2.9 Hz, 1H), 3.33 (m, 1H), 3.02 (m, 1H), 3.00 (dd, *J* = 8.9, 4.5 Hz, 1H), 2.38 (ddd, *J* = 8.8, 3.3, 1.6 Hz, 1H), 1.53–1.44 (m, 1H), 1.43–1.39 (m, 1H), 1.38–1.28 (m, 2H), 1.13 (s, 3H), 0.81 (t, *J* = 7.5 Hz, 3H); **For minor diastereomer** δ 6.49 (dd, *J* = 10.2, 1.7 Hz, 1H), 5.99 (dd, *J* = 5.7, 2.9 Hz, 1H), 5.78 (dd, *J* = 5.7, 2.9 Hz, 1H), 5.73 (d, *J* = 10.2 Hz, 1H), 3.33 (m, signal merge with the major diastereomer, 1H), 3.09 (dd, *J* = 8.5, 4.6 Hz, 1H), 3.02 (m, signal merge with the major diastereomer, 1H), 2.44 (ddd, *J* = 8.5, 3.2, 1.7 Hz, 1H), 1.60–1.53 (m, 1H), 1.53–1.44 (m, 1H), 1.43–1.39 (m, signal merge with the major diastereomer 1H), 1.38–1.28 (m, signal merge with the major diastereomer, 1H), 1.06 (s, 3H), 1.01 (t, *J* = 7.5 Hz, 3H); **^{13}C NMR** (126 MHz, $CDCl_3$, spectra contained both the diastereomers) δ 202.0, 156.7, 156.2, 135.6, 135.6, 134.6, 134.3, 130.0, 129.6, 50.5, 50.4, 49.7, 49.5, 48.8, 48.1, 47.4, 47.3, 46.5, 45.7, 40.6, 37.4, 31.8, 31.1, 25.0, 8.6, 8.3 (spectra contained dr = 5:1, two signals are overlapped); **HRMS** (GC-CI)

m/z calculated for C_{14} H_{18} O $[M]^+$: 202.135215; found 202.135100; The enantiomeric ratio was determined by HPLC on a chiral stationary phase. **HPLC** Daicel Chiralcel IC-3, ^{i}PrOH/heptane = 5/95, 1 mL/min, 25 °C, 254 nm, t_R (major enantiomer for major diastereomer) = 11.05 min, t_R (minor enantiomer for major diastereomer) = 13.58 min, er = 92:8; t_R (major enantiomer for minor diastereomer) = 11.71 min, t_R (minor enantiomer for minor diastereomer) = 17.15 min, er = 94:6.

(1*R*,4*S*,4a*R*,8*S*,8a*S*)-8-methyl-8-propyl-4,4a,8,8a-tetrahydro-1,4-methanonaphthalen-5(1*H*)-one 2b: Prepared according to the representative procedure. The product was purified by column chromatography using 5 to 8% EtOAc in hexane and obtained as yellow liquid (98%, dr = 6:1).

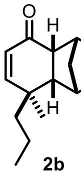

2b

^{1}H NMR (501 MHz, $CDCl_3$, spectra contained dr = 6:1) **for major diastereomer** δ 6.23 (dd, *J* = 10.2, 1.6 Hz, 1H), 6.04 (dd, *J* = 5.7, 2.8 Hz, 1H), 5.85–5.79 (m, 2H), , 3.32 (m, 1H), 3.02 (m, 1H), 2.99 (dd, *J* = 8.8, 4.6 Hz, 1H), 2.40 (ddd, *J* = 8.9, 3.3, 1.6 Hz, 1H), 1.47 – 1.36 (m, 2H), 1.36–1.28 (m, 1H), 1.28–1.17 (m, 3H), 1.13 (s, 3H), 0.84 (t, *J* = 7.0 Hz, 3H); **For minor diastereomer** δ 6.47 (dd, *J* = 10.2, 1.7 Hz, 1H), 6.00 (dd, *J* = 5.7, 2.9 Hz, 1H), 5.78 (dd, *J* = 5.5, 2.8 Hz, 1H), 5.72 (d, *J* = 10.2 Hz, 1H), 3.32 (m, signal merge with the major diastereomer, 1H), 3.07 (dd, *J* = 8.5, 4.6 Hz, 1H), 3.02 (m, signal merge with the major diastereomer, 1H), 2.44 (ddd, *J* = 8.6, 3.3, 1.6 Hz, 1H), 1.49–1.36 (m, signal merge with the major diastereomer, 2H), 1.36–1.28 (m, signal merge with the major diastereomer, 1H), 1.28–1.17 (m, signal merge with the major diastereomer, 3H), 1.06 (s, 3H), 0.99 (m, 3H); **^{13}C NMR** (126 MHz, $CDCl_3$, spectra contained both the diastereomers) δ 202.0, 157.0, 156.6, 135.6, 135.59, 134.6, 134.3, 129.7, 129.6, 50.8, 50.5, 50.4, 49.7, 49.5, 48.7, 48.0, 47.5, 47.4, 46.5, 46.1, 41.2, 37.2, 32.4, 25.3, 17.3, 17.1, 15.0, 14.6 (spectra contained dr = 6:1, two signals are overlapped); **HRMS** (GC-CI) *m/z* calculated for $C_{15}H_{20}O$ $[M]^+$: 216.150865; found 216.150690; The enantiomeric ratio was determined by HPLC on a chiral stationary phase. **HPLC** Daicel Chiralcel IC-3, ^{i}PrOH/heptane = 5/95, 1 mL/min, 25 °C, 254 nm, t_R (major enantiomer for major diastereomer) = 9.83 min, t_R (minor enantiomer for major diastereomer) = 11.59 min, er = 96:4; t_R (major enantiomer for minor diastereomer) = 11.01 min, t_R (minor enantiomer for minor diastereomer) = 16.91 min, er = 95:5; For the major diastereomer $[\boldsymbol{\alpha}]_{\boldsymbol{D}}^{\boldsymbol{20}}$ = –129.8 (*c* 0.41, $CHCl_3$).

(1*R*,4*S*,4a*R*,8a*S*)-8,8-dimethyl-4,4a,8,8a-tetrahydro-1,4-methanonaphthalen-5(1*H*)-one 2c:

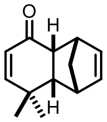

2c

Prepared according to the representative procedure. The product was purified by column chromatography using 5 to 10% EtOAc in hexane and obtained as yellow liquid (85%). **^{1}H NMR** (501 MHz, $CDCl_3$) δ 6.37 (dd, *J* = 10.2, 1.6 Hz, 1H), 6.04 (dd, *J* = 5.7, 2.8 Hz, 1H), 5.81 (dd, *J* = 5.7, 2.9 Hz, 1H), 5.71 (d, *J* = 10.2 Hz, 1H), 3.34 (m, 1H), 3.06 (dd, *J* = 8.8, 4.5 Hz, 1H), 3.01 (m, 1H), 2.42 (ddd, *J* = 8.7, 3.3, 1.6 Hz, 1H), 1.41 (dt, *J* = 8.3, 1.9 Hz, 1H), 1.31 (dt, *J* = 8.5, 1.5 Hz, 1H), 1.15 (s, 3H), 1.12 (s, 3H); **^{13}C NMR** (126 MHz, $CDCl_3$) δ 201.7, 158.3, 135.7, 134.2, 129.0, 50.6, 49.6, 47.8, 47.7, 47.2, 36.2, 33.9, 26.6; **HRMS** (GC-CI) *m/z* calculated for $C_{13}H_{16}O$ $[M]^+$: 188.119565; found 188.119500; The enantiomeric ratios was determined by HPLC on a chiral stationary phase. **HPLC** Daicel Chiralcel IC-3, ^{i}PrOH/heptane = 5/95, 1 mL/min, 25 °C, 254 nm, t_R (major) = 10.87 min, t_R (minor) = 15.01 min, er = 96:4; $[\boldsymbol{\alpha}]_{\boldsymbol{D}}^{\mathbf{20}}$ = –152.2 (*c* 0.43, $CHCl_3$).

(1*R*,4*S*,4a*R*,8*S*,8a*S*)-8-isopropyl-8-methyl-4,4a,8,8a-tetrahydro-1,4-methanonaphthalen-5(1*H*)-one 2d:

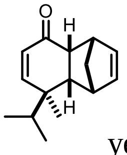

2d

Prepared according to the representative procedure. The product was purified by column chromatography using 5 to 10% EtOAc in hexane and obtained as yellow liquid. (58%, dr >20:1). **^{1}H NMR** (501 MHz, $CDCl_3$) δ 6.18 (dd, *J* = 10.4, 1.5 Hz, 1H), 6.07 (dd, *J* = 5.7, 2.8 Hz, 1H), 5.88 (d, *J* = 10.4 Hz, 1H), 5.82 (dd, *J* = 5.7, 2.9 Hz, 1H), 3.32 (m, 1H), 2.99 (m, 1H), 2.95 (dd, *J* = 8.9, 4.5 Hz, 1H), 2.43 (ddd, *J* = 9.0, 3.3, 1.6 Hz, 1H), 1.53 (hept, *J* = 6.8 Hz, 1H), 1.41 (dt, *J* = 8.3, 1.9 Hz, 1H), 1.33 (dt, *J* = 8.3, 1.5 Hz, 1H), 1.12 (s, 3H), 0.88 (d, *J* = 6.7 Hz, 3H), 0.84 (d, *J* = 6.9 Hz, 3H); **^{13}C NMR** (126 MHz, $CDCl_3$) δ 202.0, 156.1, 135.5, 134.9, 130.2, 50.2, 49.6, 49.5, 47.8, 43.4, 41.7, 39.8, 23.0, 18.0, 17.2; **HRMS** (GC-CI) *m/z* calculated for $C_{15}H_{20}O$ $[M]^+$: 216.150865; found 216.150850; The enantiomeric ratios was determined by HPLC on a chiral stationary phase. **HPLC** Daicel Chiralcel IC-3, ^{i}PrOH/heptane = 5/95, 1 mL/min, 25 °C, 254 nm, t_R (major) = 10.63 min, t_R (minor) = 12.56 min, er = 95:5; $[\boldsymbol{\alpha}]_{\boldsymbol{D}}^{\mathbf{20}}$ = –115.0 (*c* 0.24, $CHCl_3$).

(1*R*,4*S*,4a*R*,8*S*,8a*S*)-8-allyl-8-methyl-4,4a,8,8a-tetrahydro-1,4-methanonaphthalen-5(1*H*)-one 2e:

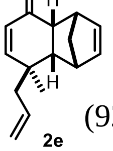

2e

Prepared according to the representative procedure The product was purified by column chromatography using 15% EtOAc in hexane and obtained as yellow liquid (92%, dr = 4:1). **^{1}H NMR** (501 MHz, $CDCl_3$, spectra contained dr = 4:1) **for major diastereomer** δ 6.24 (dd, *J* = 10.2, 1.6 Hz, 1H), 6.04 (dd, *J* = 5.6, 2.8 Hz, 1H), 5.82 (d, *J* = 10.4

Hz, 1H), 5.81 (dd, J = 5.7, 3.0 Hz, 1H), 5.71–5.63 (m, 1H), 5.10–5.01 (m, 2H), 3.33–3.30 (m, 1H), 3.00 (m, 1H), 2.97 (dd, J = 8.8, 4.5 Hz, 1H), 2.45 (m, 1H), 2.19–2.13 (m, 1H), 2.04 (ddt, J = 13.4, 7.3, 1.2 Hz, 1H), 1.40 (dt, J = 8.4, 2.0 Hz, 1H), 1.34–1.30 (m, 1H), 1.17 (s, 3H); **For minor diastereomer** δ 6.50 (dd, J = 10.3, 1.7 Hz, 1H), 6.02 (dd, J = 5.5, 2.6 Hz, 1H), 5.97–5.87 (dddd, J = 16.7, 10.1, 8.3, 6.3 Hz, 1H), 5.81–5.79 (m, 1H), 5.74 (d, J = 10.3 Hz, 1H), 5.22–5.15 (m, 2H), 3.34–3.33 (m, 1H), 3.11–3.08 (m, 2H), 2.45 (m, signal merge with the major diastereomer, 1H), 2.32 (ddt, J = 13.8, 6.3, 1.4 Hz, 1H), 2.19–2.13 (m, signal merge with the major diastereomer,1H) 1.43 (dt, J = 8.3, 1.9 Hz, 1H), 1.34–1.30 (m, signal merge with the major diastereomer, 1H), 1.09 (s, 3H); **^{13}C NMR** (126 MHz, $CDCl_3$, spectra contained both the diastereomers) δ 201.9, 201.9, 156.3, 155.4, 135.8, 135.8, 134.7, 134.1, 133.8, 133.4, 130.0, 129.7, 119.5, 119.2, 52.0, 50.5, 50.4, 49.7, 49.6, 48.6, 48.1, 47.7, 47.3, 46.5, 45.7, 43.2, 37.8, 36.6, 32.8, 25.1; **HRMS** (GC-CI) *m/z* calculated for $C_{15}H_{19}O$ $[M+H]^+$: 215.143040; found 215.142730; The enantiomeric ratio was determined by HPLC on a chiral stationary phase. **HPLC** 150 mm Chiralcel AS-3R 4.6 mm i.D. Acetonitrile/Water - gradient: 50 % B - 10 - 90% B 1.0 ml/min, 22.0 MPa, 25 °C, 220 nm, 1 mL/min; t_R (minor enantiomer for major diastereomer) = 5.82 min, t_R (major enantiomer for major diastereomer) = 6.62 min, er = 2.5:97.5; t_R (major enantiomer for minor diastereomer) = 7.18 min, t_R (minor enantiomer for minor diastereomer) = 7.63 min, er = 96.5:3.5.

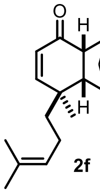

2f

(1*R*,4*S*,4a*R*,8*S*,8a*S*)-8-methyl-8-(4-methylpent-3-en-1-yl)-4,4a,8,8a-tetrahydro-1,4-methanonaphthalen-5(1*H*)- 2f: Prepared according to the representative procedure. The product was purified by column chromatography using 8% EtOAc in hexane and obtained as colorless liquid (66%, dr = 7:1). **^{1}H NMR** (500 MHz, $CDCl_3$, spectra contained dr = 7:1) **for major diastereomer** δ 6.26 (dd, J = 10.3, 1.6 Hz, 1H), 6.05 (dd, J = 5.8, 2.8 Hz, 1H), 5.83 (d, J = 10.3 Hz, 1H), 5.81 (dd, J = 5.8, 2.8 Hz, 1H), 5.00 (tp, J = 7.0, 1.5 Hz, 1H), 3.33 (m, 1H), 3.03–3.00 (m, 2H), 2.42 (ddd, J = 8.7, 3.3, 1.5 Hz, 1H), 1.89 (m, 2H), 1.65 (d, J = 1.4 Hz, 3H), 1.54 (brs, 3H), 1.49–1.43 (m, 1H), 1.43–1.39 (m, 1H), 1.34–1.24 (m, 2H), 1.15 (s, 3H); **For minor diastereomer** δ 6.50 (dd, J = 10.3, 1.7 Hz, 1H), 6.00 (dd, J = 5.7, 2.8 Hz, 1H), 5.78 (dd, J = 5.7, 2.9 Hz, 1H), 5.73 (d, J = 10.2 Hz, 1H), 5.14 (tdd, J = 6.9, 2.9, 1.5 Hz, 1H), 3.33 (signal merge with the major dr, 1H), 3.09 (dd, J = 8.6, 4.6 Hz, 1H), 3.03–3.00 (signal merge with the major dr, 1H), 2.45 (ddd, J = 8.4, 3.1, 1.6 Hz, 1H), 2.08 (m, 2H), 1.72 (d, J = 1.3 Hz, 3H), 1.58 (brs, 3H), 1.49–1.24 (signal merge with the major diastereomer, 4H), 1.10 (s, 3H); **^{13}C NMR** (126 MHz, $CDCl_3$, spectra contained both the diastereomers) δ 201.8, 156.8, 156.2, 135.6, 135.6,

134.6, 134.3, 132.2, 129.9, 129.7, 124.2, 123.8, 50.5, 50.4, 49.7, 49.6, 48.7, 48.3, 48.1, 47.5, 47.4, 46.6, 46.1, 38.8, 37.2, 32.4, 29.9, 25.9, 25.8, 25.1, 22.9, 22.6, 17.9, 17.8 (two signals at 201.8 and 132.2 are overlapped); **HRMS** (ESI) *m/z* calculated for $C_{18}H_{24}ONa$ $[M+Na]^+$: 279.171934; found 279.171790; The enantiomeric and diastereomeric ratios were determined by HPLC on a chiral stationary phase; **HPLC** Daicel Chiralcel IC-3, ^{i}PrOH/heptane = 5/95, 1 mL/min, 25 °C, 254 nm, t_R (major enantiomer for major diastereomer) = 8.66 min, t_R (minor enantiomer for major diastereomer) = 10.32 min, e.r. = 97:3; t_R (major enantiomer for minor diastereomer) = 9.67 min, t_R (minor enantiomer for minor diastereomer) = 14.68 min, er = 96.5:3.5.

(1*R*,4*S*,4a*R*,8*S*,8a*S*)-8-benzyl-8-methyl-4,4a,8,8a-tetrahydro-1,4-methanonaphthalen-5(1*H*)-one 2g: Prepared according to the representative procedure. The product was purified by column

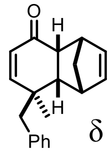

2g

chromatography using 15% EtOAc in hexane and obtained as yellow liquid (91%, dr = 13:1). **^{1}H NMR** (600 MHz, $CDCl_3$, spectra contained dr = 13:1) **for major diastereomer** δ 7.26–7.22 (m, 3H), 7.06–7.04 (m, 2H), 6.18 (dd, *J* = 10.2, 1.6 Hz, 1H), 6.03 (dd, *J* = 5.7, 2.8 Hz, 1H), 5.82 (d, *J* = 10.2 Hz, 1H), 5.76 (dd, *J* = 5.7, 2.9 Hz, 1H), 3.22 (m, 1H), 3.00 (m, 1H), 2.75 (d, *J* = 13.0 Hz, 1H), 2.61–2.54 (m, 3H), 1.37 (dt, *J* = 8.2, 1.9 Hz, 1H), 1.28–1.26 (m, 1H), 1.22 (s, 3H); **For minor diastereomer** δ 7.35–7.25 (m, 3H), 7.18–7.19 (m, 2H), 6.57 (dd, *J* = 10.3, 1.7 Hz, 1H), 6.19–6.17 (signal merge with major dr, 1H), 5.88 (dd, *J* = 5.7, 2.8 Hz, 1H), 5.71 (d, *J* = 10.3 Hz, 1H), 3.38 (m, 1H), 3.22 (signal merge with major dr, 1H), 3.14 (dd, *J* = 8.7, 4.6 Hz, 1H), 2.79 (br, 2H), 2.59 (signal merge with major dr, 1H), 1.49 (dt, *J* = 8.4, 1.9 Hz, 1H), 1.38 (signal merge with major dr, 1H), 1.05 (s, 3H); **^{13}C NMR** (150 MHz, $CDCl_3$, 76.98 ppm for ^{13}C of $CDCl_3$) **for major diastereomer** δ 201.2, 155.8, 136.3, 135.6, 134.5, 131.1, 130.4, 128.0, 126.7, 53.5, 50.3, 49.5, 48.0, 47.2, 45.8, 38.6, 25.0; **For minor diastereomer** δ 201.7, 154.2, 136.8, 135.9, 133.8, 131.1, 129.1, 128.2, 126.6, 50.4, 49.5, 48.9, 48.1, 46.5, 44.1, 37.1, 33.5; **HRMS** (GC-CI) m/z calculated for $C_{19}H_{20}O$ $[M]^+$: 264.150865; found 264.151120; The enantiomeric ratio was determined by HPLC on a chiral stationary phase; **HPLC** Daicel Chiralcel OJ-3, ^{i}PrOH/heptane = 5/95, 0:5 mL/min, 25 °C, 254 nm, t_R (major) = 12:38 min, t_R (minor) = 13:92 min, er 97.5:2.5.

Suggested Structure of major diastereomer

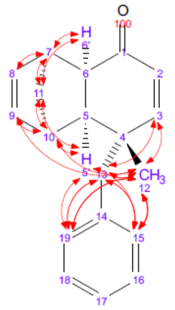

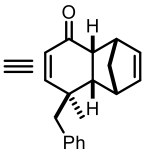

2g

User Report
GHS-GA-575-01

Assignments						
Atom	Chemical Shift	J	COSY	HSQC	HMBC	NOESY
1 C	201.24				2, 3, 5', 6', 11"	
2 C	130.39			2		
H	5.82	10.20(3)	3	2	1, 4, 6	
3 C	155.78			3	12, 13', 13"	
H	6.18	10.20(2), 1.60(5')	2, 5'	3	1, 4, 5, 12	12, 13', 13"
4 C	38.63				2, 3, 5', 6', 12, 13', 13"	
5 C	45.78			5'	3, 11", 12, 13', 13"	
5' H	2.55	1.60(3), 3.30(10), 8.80(6')	3, 6', 10	5	1, 4, 6, 9, 13	10, 11', 19
6 C	47.96			6'	2, 5', 10, 11"	
6' H	2.60	4.60(7), 8.80(5')	5', 7	6	1, 4, 7	7, 11'
7 C	50.29			7	6', 8, 9, 10	
H	3.22	4.60(6')	6', 8, 11', 11"	7	9	6', 8, 11', 11"
8 C	135.61			8	9, 10, 11'	
H	5.76	5.70(9)	7, 8, 9	8	7, 9, 10	7
9 C	134.49			9	5', 7, 8, 11'	
H	6.03	5.70(8)	8, 10	9	7, 8, 10, 11	10, 12
10 C	47.18			10	8, 9	
H	3.00	3.30(5')	5', 9, 11', 11"	10	6, 7, 8	5', 9, 11', 11", 12
11 C	49.46			11', 11"	9	
H'	1.27	8.20(11")	7, 10	11	8, 9	5', 6', 7, 10
H"	1.37	8.20(11')	7, 10	11	1, 5, 6	7, 10

Assignments						
Atom	Chemical Shift	J	COSY	HSQC	HMBC	NOESY
12 C	25.03			12	3, 13', 13"	
H3	1.22			12	3, 4, 5, 13	3, 9, 10, 13', 13", 19
13 C	53.48			13', 13"	5', 12, 15, 19	
H'	2.58	13.00(13")		13	3, 4, 5, 12, 14, 15, 19	3, 12, 19
H"	2.75	13.00(13')		13	3, 4, 5, 12, 14, 15, 19	3, 12, 19
14 C	136.28				13', 13"	
15 C	131.12			15	13', 13", 17, 19	
H	7.05			15	13, 17, 19	
16 C	127.98			16		
H	7.25			16		
17 C	126.72			17	15, 19	
H	7.24			17	15, 19	
18 C	127.98			18		
H	7.25			18		
19 C	131.12			19	13', 13", 15, 17	
H	7.05			19	13, 15, 17	5', 12, 13', 13"
100 O						

Chem3D model of structure

NOE contacts

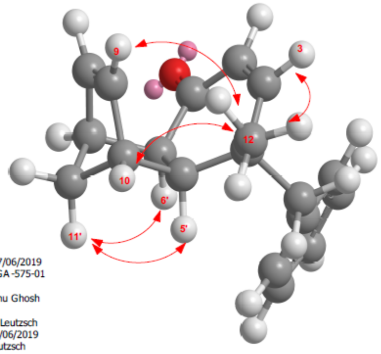

P-ID: ML00000
Measured on: 17/06/2019
CHIFFRE: GHS-GA-575-01
ELNA #: 2946
Client: Dr. Santanu Ghosh
Group: List
Spectroscopist: Leutzsch
Analysed on: 17/06/2019
Analysed by: Leutzsch
Amount: 4 mg
Solvent: CDCl3
Reference: solvent
Temperature: 298 K
Spectrometer: AV600a
Probe: Z44896_0147 (CP TCI 600S3 H-C/N-D-05 Z)
Experiments: 1H-zg30, 13C-zgdc30, [1H, 1H]-cosygpmfphpp, [13C, 1H]-ASAP_hsqcedetgpsp, [13C, 1H]-hmbcetgpl3nd, [1H, 1H]-noesygpph

Table 2. Peak table for **2g** (major diastereomer), COSY, HMBC, HSQC, NOESY signals for the assignment of relative stereochemistry.

Suggested Structure for minor diastereomer

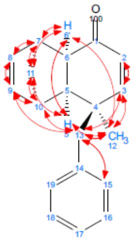

Structure could not be fully assigned due to overlap, however most relevant NOEs could be extracted

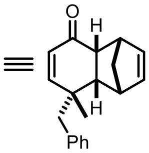

2g'

Assignments			
Atom	Chemical Shift	HSQC	NOESY
1 C	201.71		
2 C	129.05	2	
H	5.71	2	3
3 C	154.17	3	
H	6.57	3	2, 12, 13
4 C	37.05		
5 C	48.85	5'	
5' H	2.59	5	6', 11", 12
6 C	48.12	6'	
6' H	3.14	6	5', 7, 11", 12
7 C	50.35	7	
H	3.38	7	6', 8, 11', 11"
8 C	135.88	8	
H	5.88	8	7
9 C	133.83	9	
H	6.18	9	10, 13
10 C	46.47	10	
H	3.22	10	9, 11', 11", 13
11 C	49.54	11', 11"	
H'	1.49	11	7, 10
H"	1.38	11	5', 6', 7, 10

Assignments			
Atom	Chemical Shift	HSQC	NOESY
12 C	33.46	12	
H3	1.05	12	3, 5', 6', 13
13 C	44.13	13	
H2	2.79	13	3, 9, 10, 12, 13, 15
14 C	136.80		
15 C	131.12	15	
H	7.19	15	13
16 C	128.16	16	
H	7.33	16	
17 C	126.56	17	
H		17	
18 C	128.16	18	
H	7.33	18	
19 C	131.12	19	
H	7.19	19	
100 O			

Chem3D model of structure

NOE contacts

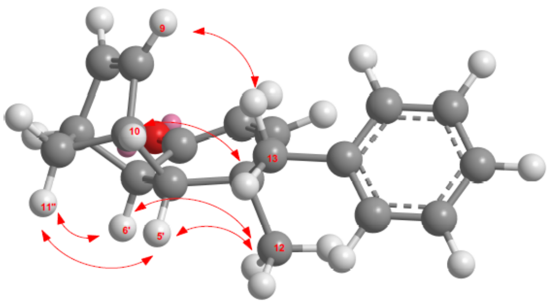

Table 3. Peak table for **2g′** (minor diastereomer): COSY, HMBC, HSQC, NOESY signals for the assignment of relative stereochemistry

(1*R*,4*S*,4a*R*,8*S*,8a*S*)-8-methyl-8-phenethyl-4,4a,8,8a-tetrahydro-1,4-methanonaphthalen-5(1*H*)-one 2h: Prepared according to the representative procedure. The product was purified by column chromatography using 15% EtOAc in hexane and obtained as colorless liquid (64%, dr = 3:1). **^{1}H NMR** (500 MHz, $CDCl_3$, data contained mixture of diastereomers with dr = 2.7:1) **for major diastereomer** δ 7.29–7.22 (m, 3H), 7.11–7.10 (m, 2H), 6.32 (dd, *J* = 10.3, 1.6 Hz, 1H), 6.08 (dd, *J* = 5.7, 2.8 Hz, 1H), 5.89 (d, *J* = 10.3 Hz, 1H), 5.84 (dd, *J* = 5.7, 2.9 Hz, 1H), 3.36 (m, 1H), 3.09–3.02 (m, 2H), 2.58–2.48 (m, 3H), 1.86–1.68 (m, 1H), 1.64–1.57 (m, 1H), 1.44 (dt, *J* = 8.3, 1.8 Hz, 1H), 1.35 (ddt, *J* = 8.4, 6.5, 1.5 Hz, 1H), 1.22 (s, 3H); **For minor diastereomer** δ 7.35–7.32 (m, 2H), 7.18–7.15 (m, 3H), 6.54 (dd, *J* = 10.2, 1.7 Hz, 1H), 6.00 (dd, *J* = 5.7, 2.9 Hz, 1H), 5.80 (dd, *J* = 5.7, 2.8 Hz, 1H), 5.77 (d, *J* = 10.2 Hz, 1H), 3.36 (signal merge with the major dr, 1H), 3.13 (dd, *J* = 8.5, 4.6 Hz, 1H), 3.10 (m, 1H), 2.78–2.68 (m, 2H), 2.58–2.48 (signal merge with the major dr, 1H), 1.86–1.68 (signal merge with the major dr, 1H), 1.64–1.57 (m, signal merge with the major dr, 1H), 1.44 (signal merge with the major dr, 1H), 1.35 (signal merge with the major dr, 1H), 1.20 (s, 3H); **^{13}C NMR** (126 MHz, $CDCl_3$, spectra contains both the diastereomers) δ 201.9, 201.7, 156.4, 155.8, 142.0, 135.7, 134.6, 134.2, 130.2, 129.9, 128.7, 128.6, 128.4, 126.2, 126.1, 50.5, 50.5, 50.4, 49.8, 49.6, 48.7, 48.1, 47.5, 47.4, 46.6, 46.2, 41.1, 37.3, 36.7, 32.5, 30.7, 30.5, 25.2 (3 signals are overlapped); **HRMS** (GC-EI) *m/z* calculated for $C_{20}H_{22}O$ $[M]^{+}$: 278.166515; found 278.166690; The enantiomeric and diastereomeric ratios were determined by HPLC on a chiral stationary phase. **HPLC** Daicel Chiralcel IC-3, ^{i}PrOH/heptane = 5/95, 1 mL/min, 25 °C, 254 nm, t_R (major enantiomer for major diastereomer) = 14.41 min, t_R (minor enantiomer for major diastereomer) = 17.95 min, er = 94:6; t_R (major enantiomer for minor diastereomer) = 13.56 min, t_R (minor enantiomer for minor diastereomer) = 22.23 min, er = 97:3.

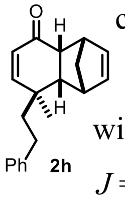

2h

(1R,4S,4aR,8R,8aS)-8-methyl-8-phenyl-4,4a,8,8a-tetrahydro-1,4 methanonaphthalen-5(1H)-one 2i′: Prepared according to the representative procedure. The product was purified by column chromatography using 15% EtOAc in hexane and obtained as yellow liquid (90%, dr = 9:1). **^{1}H NMR** (501 MHz, $CDCl_3$ data contained mixture of diastereomers with dr = 9:1) **for major diastereomer** δ 7.41–7.37 (m, 2H), 7.33–7.28 (m, 3H), 7.04 (dd, *J* = 10.3, 1.6

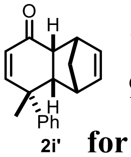

2i′

Hz, 1H), 5.97 (d, J = 10.4 Hz, 1H), 5.82 (dd, J = 5.6, 2.8 Hz, 1H), 5.78 (ddd, J = 5.7, 2.8, 1.0 Hz, 1H), 3.38–3.35 (m, 1H), 3.15 (dd, J = 8.7, 4.6 Hz, 1H), 2.82 (ddd, J = 8.7, 3.2, 1.6 Hz, 1H), 2.18 (m, 1H), 1.56 (s, 3H), 1.27 – 1.20 (m, 2H); **For minor diastereomer** δ 7.41–7.19 (m, 5H), 6.35 (dd, J = 10.2, 1.6 Hz, 1H), 6.17 (dd, J = 5.5, 2.8 Hz, 1H), 6.05 (d, J = 10.2 Hz, 1H), 5.94 (dd, J = 5.7, 2.9 Hz, 1H), 3.38 – 3.35 (m, 1H), 3.19 (m, 1H), 2.98 (dd, J = 8.9, 4.6 Hz, 1H), 2.76 (ddd, J = 8.8, 3.2, 1.6 Hz, 1H), 1.60 (s, 3H), 1.27–1.20 (m, 2H); **^{13}C NMR** (126 MHz, $CDCl_3$) **for the major diastereomer** δ 201.3, 155.5, 146.0, 135.5, 134.4, 129.9, 128.5, 126.8, 126.8, 50.8, 50.6, 49.7, 48.4, 47.8, 41.8, 34.8; (^{13}C NMR for minor diastereomer not included due to the week signals into the spectra); **HRMS** (GC-CI) *m/z* calculated for $C_{18}H_{18}O$ $[M]^+$: 250.135215; found 250.135020; The enantiomeric ratios were determined by 2-dimension HPLC on a chiral stationary phase 150 mm Chiralcel OJ-3R, 4.6 mm i.D., Acetonitrile / Water = 70:30, 1.0 ml/min, 20.0 MPa, 25 °C, UV, 220 nm, , t_R (major enantiomer for major diastereomer) = 4.9 min, t_R (minor enantiomer for major diastereomer) = 5.69 min, er = 94.5:5.5; t_R (major enantiomer for minor diastereomer) = 3.58 min, t_R (minor enantiomer for minor diastereomer) = 4.33 min, er = 94:6.

Major diasteromer as determined by 1D and 2D NMR analysis:

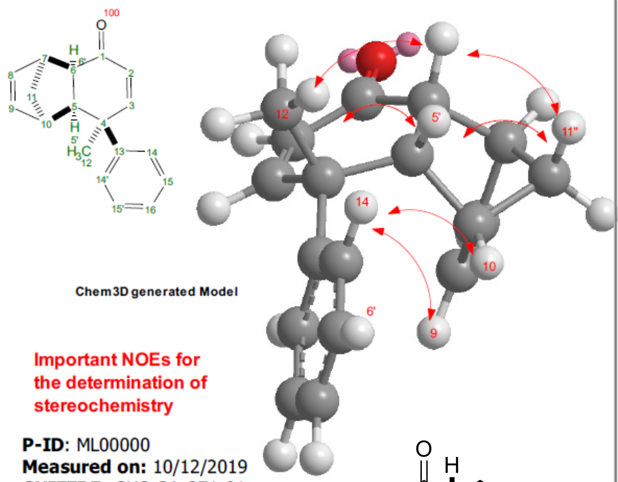

Atom	δ (ppm)	J	COSY	HSQC	HMBC	NOESY
1 C	201.16				3, 5', 6'	
2 C	129.76			2	6'	
H	5.97	10.40(3)	3	2	4, 6, 12	3
3 C	155.33			3	5', 12	
H	7.04	10.40(2), 1.60(5')	2	3	1, 4, 5, 13	2, 12
4 C	41.63				2, 3, 5', 6', 12, 14, 14'	
5 C	50.38			5'	3, 6', 11', 11", 12	
5' H	2.82	1.60(3), 3.20(10), 8.70(6')	6', 10	5	1, 3, 4, 6, 9, 10, 12	6', 10, 11", 12, 14, 14'
6 C	47.63			6'	2, 5', 11', 11"	
6' H	3.15	4.60(7), 8.70(5')	5', 7	6	1, 2, 4, 5, 7, 8, 10	5', 7, 11", 12
7 C	50.62			7	6', 8	
H	3.36	2.80(8), 4.60(6'), 1.90(11')	6', 8, 9	7		6', 8, 11"
8 C	135.31			8	6', 9	
H	5.82	2.80(7), 5.70(9)	7, 9, 10	8	7, 9	7
9 C	134.24			9	5', 8, 11', 11"	
H	5.78	2.80(10), 5.70(8)	7, 8, 10	9	8, 10	10, 14, 14'
10 C	48.23			10	5', 6', 9	
H	2.17	2.80(9), 3.20(5'), 1.90(11')	5', 8, 9	10		5', 9, 11", 14, 14'

Atom	δ (ppm)	J	COSY	HSQC	HMBC	NOESY
11 C	49.50			11', 11"		
H'	1.25	1.90(7), 1.90(10), 8.50(11")		11	5, 6, 9	
H"	1.22	8.50(11')		11	5, 6, 9	5', 6', 7, 10
12 C	34.64			12	2, 5'	
H3	1.56			12	3, 4, 5, 13	3, 5', 6', 14, 14'
13 C	145.83				3, 12, 15, 15'	
14 C	126.61				14', 16	
H	7.31				4, 14', 16	5', 9, 10, 12
14' C	126.61				14, 16	
H	7.31				4, 14, 16	5', 9, 10, 12
15 C	128.36			15	15'	
H	7.39			15	13, 15'	
15' C	128.36			15'	15	
H	7.39			15'	13, 15	
16 C	126.65			16	14, 14'	
H	7.31			16	14, 14'	
100 O						

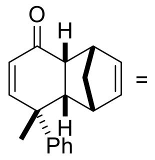

2i'

P-ID: ML00000
Measured on: 10/12/2019
CHIFFRE: GHS-GA-371-01
ELNA#: 3434
Client: Dr. Santanu Ghosh
Group: List
Spectroscopist: Leutzsch
Analysed on: 10/12/2019
Analysed by: Leutzsch
Amount: 10 mg
Solvent: CDCl3
Reference: solvent
Temperature: 298 K
Spectrometer: AV500as
Probe: 5 mm PABBO BB-1H/D Z-GRD Z119470/0004
Experiments: 1H-zg30, [13C, 1H]-hsqcedetgp, [1H, 1H]-clipcosysp.cf, [13C, 1H]-hmbcgpl2ndqf, 13C-zgdc30, [1H, 1H]-noesygpph

Table 4. Peak table for **2i′**: COSY, HMBC, HSQC, NOESY signals for the assignment of relative stereochemistry.

(1*R*,4*S*,4a*R*,8*R*,8a*S*)-8-methoxy-8-methyl-4,4a,8,8a-tetrahydro-1,4-methanonaphthalen-5(1*H*)-one 2j′: Prepared according to the representative procedure. The product was obtained as

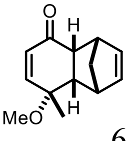

2j'

diastereomer (>20:1 dr) and purified by column chromatography using 15% EtOAc in hexane and obtained as yellow liquid (92%, dr >20:1). **^{1}H NMR** (501 MHz, $CDCl_3$) δ 6.57 (dd, J = 10.4, 1.6 Hz, 1H), 6.03 (dd, J = 5.6, 2.9 Hz, 1H), 5.74 (dd, J = 5.5, 2.8 Hz, 1H), 5.70 (d, J = 10.4 Hz, 1H), 3.35 (s, 3H), 3.32–3.29 (m, 1H), 3.14–3.11 (m, 1H), 2.99 (dd, J = 8.7, 4.4 Hz, 1H), 2.76 (ddd, J = 8.7, 3.3, 1.6 Hz, 1H), 1.40 (dt, J = 8.4, 1.9 Hz, 1H), 1.34 (s, 3H), 1.31 (dt, J = 8.4, 1.5 Hz, 1H);**^{13}C NMR** (126 MHz, $CDCl_3$) δ 201.2, 151.7, 135.7, 134.3, 129.1, 73.7, 50.9, 50.4, 48.9, 48.2, 46.7, 46.6, 29.7; **HRMS** (ESI) *m/z* calculated for $C_{13}H_{17}O_2$ $[M+H]^+$: 205.122305; found 205.122490; The enantiomeric ratio was determined by HPLC on a chiral stationary phase. **HPLC** Daicel Chiralcel IC-3, ^{i}PrOH/heptane = 5/95, 1 mL/min, 25 °C, 254 nm, t_R (major) = 9.48 min, t_R (minor) = 12.13 min, er = 96:4; $[\alpha]_D^{20}$ = −144.5 (c 0.31, $CHCl_3$).

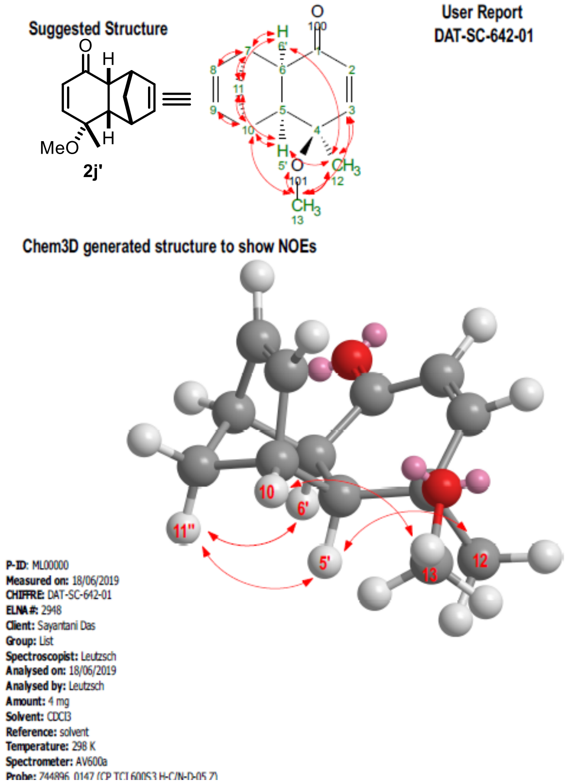

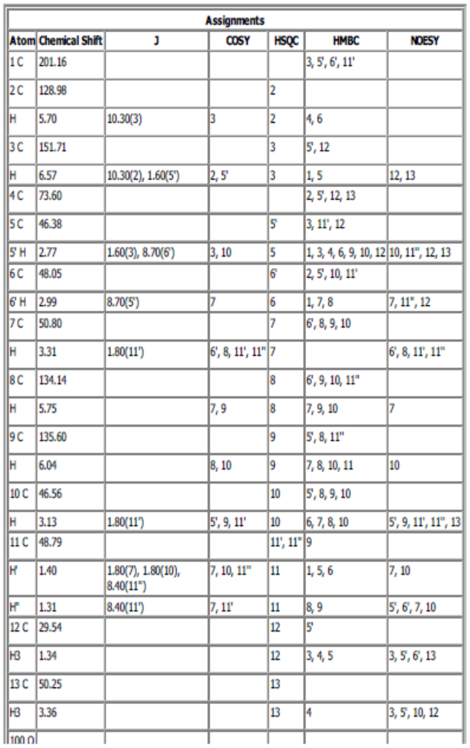

Assignments						
Atom	Chemical Shift	J	COSY	HSQC	HMBC	NOESY
1 C	201.16				3, 5', 6', 11'	
2 C	128.98			2		
H	5.70	10.30(3)	3	2	4, 6	
3 C	151.71			3	5', 12	
H	6.57	10.30(2), 1.60(5')	2, 5'	3	1, 5	12, 13
4 C	73.60				2, 5', 12, 13	
5 C	46.38			5'	3, 11', 12	
5' H	2.77	1.60(3), 8.70(6')	3, 10	5	1, 3, 4, 6, 9, 10, 12	10, 11", 12, 13
6 C	48.05			6'	2, 5', 10, 11'	
6' H	2.99	8.70(5')	7	6	1, 7, 8	7, 11", 12
7 C	50.80			7	6', 8, 9, 10	
H	3.31	1.80(11')	6', 8, 11', 11"	7		6', 8, 11', 11"
8 C	134.14			8	6', 9, 10, 11"	
H	5.75		7, 9	8	7, 9, 10	7
9 C	135.60			9	5', 8, 11"	
H	6.04		8, 10	9	7, 8, 10, 11	10
10 C	46.56			10	5', 8, 9, 10	
H	3.13	1.80(11')	5', 9, 11'	10	6, 7, 8, 10	5', 9, 11', 11", 13
11 C	48.79			11', 11"	9	
H'	1.40	1.80(7), 1.80(10), 8.40(11")	7, 10, 11"	11	1, 5, 6	7, 10
H"	1.31	8.40(11')	7, 11'	11	8, 9	5', 6', 7, 10
12 C	29.54			12	5'	
H3	1.34			12	3, 4, 5	3, 5', 6', 13
13 C	50.25			13		
H3	3.36			13	4	3, 5', 10, 12
100 O						

Table 5. Peak table for **2j′**: COSY, HMBC, HSQC, NOESY signals for the assignment of relative stereochemistry.

(1*S*,4*R*,4a*S*,5*R*,8a*R*)-5-methyl-8-oxo-1,4,4a,5,8,8a-hexahydro-1,4-methanonaphthalene-5-carbonitrile 2k′: Prepared according to the representative procedure. The product was purified by

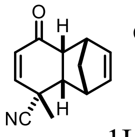

2k'

column chromatography using 10 to 15% EtOAc in hexane, and obtained as colorless semisolid. (95%, dr >20:1). **^{1}H NMR** (501 MHz, $CDCl_3$) δ 6.57 (dd, *J* = 10.2, 1.5 Hz, 1H), 6.30 (dd, *J* = 5.7, 2.8 Hz, 1H), 5.94 (dd, *J* = 5.8, 2.9 Hz, 1H), 5.90 (d, *J* = 10.2 Hz, 1H), 3.41 (m, 1.5 Hz, 2H), 3.08 (dd, *J* = 9.0, 4.2 Hz, 1H), 2.71 (ddd, *J* = 8.9, 3.4, 1.5 Hz, 1H), 1.61 (s, 3H), 1.54 (dt, *J* = 8.7, 1.9 Hz, 1H), 1.42–1.40 (m, 1H); **^{13}C NMR** (126 MHz, $CDCl_3$) δ 198.2, 146.7, 136.8, 134.1, 130.2, 121.8, 51.3, 49.5, 49.3, 47.7, 45.3, 35.5, 32.5; **HRMS (ESI)** *m/z* calculated for $C_{13}H_{13}NONa$ $[M+Na]^+$: 222.088933; found 222.088830; Spectral analysis matched with literature reported value. The enantiomeric ratio was determined by HPLC on a chiral stationary phase. **HPLC** Daicel Chiralcel IC-3, ^{i}PrOH/heptane = 10/90, 1 mL/min, 25 °C, 254 nm, t_R (major) = 10.58 min, t_R (minor) = 12.87 min, er = 91:9; $[\boldsymbol{\alpha}]_D^{20}$ = –132.3 (*c* 0.26, $CHCl_3$)

methyl (1*S*,4*R*,4a*S*,5*R*,8a*R*)-5-methyl-8-oxo-1,4,4a,5,8,8a-hexahydro-1,4-methanonaphthalene-5-carboxylate 2l′: Prepared according to the representative procedure. The

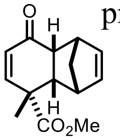

2l'

product was purified by column chromatography using 10 to 15% EtOAc in hexane, obtained as colorless semisolid (91%, dr >20:1). **^{1}H NMR** (501 MHz, $CDCl_3$) δ 6.94 (dd, *J* = 10.4, 1.6 Hz, 1H), 5.94 (dd, *J* = 5.7, 2.9 Hz, 1H), 5.85–5.80 (m, 2H), 3.82 (s, 3H), 3.41–3.28 (m, 1H), 3.10 (dd, *J* = 8.6, 4.4 Hz, 1H), 2.85 (br, 1H), 2.75 (ddd, *J* = 8.7, 3.2, 1.6 Hz, 1H), 1.43–1.41 (m, 1H), 1.41 (s, 3H), 1.36–1.34 (m, 1H);**^{13}C NMR** (126 MHz, $CDCl_3$) δ 200.6, 175.0, 150.3, 135.8, 134.1, 129.5, 52.3, 50.8, 49.8, 48.1, 47.3, 46.2, 45.2, 31.6; **HRMS** (ESI) *m/z* calculated for $C_{14}H_{17}O_3$ $[M+H]^+$: 233.117220; found 233.117200.The enantiomeric ratios were determined by HPLC on a chiral stationary phase. **HPLC** Daicel Chiralcel IC-3, ^{i}PrOH/heptane = 5/95, 1 mL/min, 25 °C, 254 nm, t_R (major) = 12.32 min, t_R (minor) = 15.5 min, er = 92:8; $[\boldsymbol{\alpha}]_D^{20}$= –99.5 (*c* 0.46, $CHCl_3$). The absolute configuration of **2l′** was confirmed in comparison of specific rotation with the literature report[10] $[\boldsymbol{\alpha}]_D^{27}$= –74.3 (*c* = 2.22, $CHCl_3$, for er = 87:13).

NMR data supports a structure with the following relative stereochemistry:

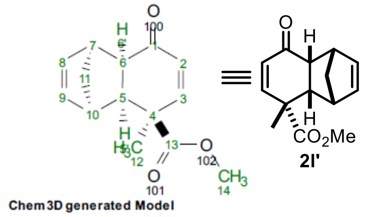

Chem3D generated Model

NOEs

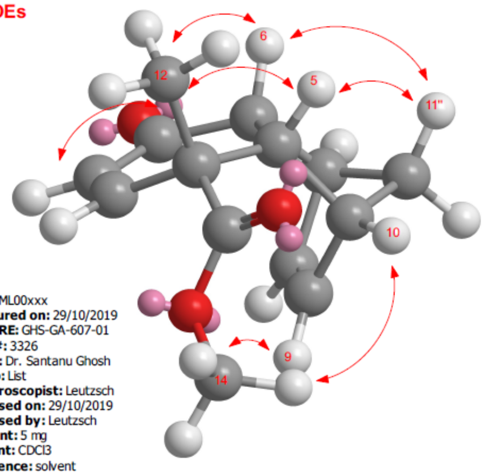

User Report
GHS-GA-607-01

Assignments						
**Atom**	**Chemical Shift**	**J**	**COSY**	**HSQC**	**HMBC**	**NOESY**
1 C	200.447				3, 6'	
2 C	129.350			2	6'	
H	5.829	10.40(3)	3	2	4, 6, 12	3
3 C	150.142			3	5', 12	
H	6.937	10.40(2), 1.50(5')	2, 5'	3	1, 4, 5, 12, 13	2, 12
4 C	45.006				2, 3, 5', 12, 14	
5 C	46.011			5'	3, 6', 11', 11", 12	
5' H	2.750	1.50(3), 3.20(10), 8.60(6')	3, 6', 10	5	3, 4, 6, 9, 10, 12	6', 10, 11", 12
6 C	47.145			6'	2, 5', 11', 11"	
6' H	3.095	4.40(7), 8.60(5')	5', 7	6	1, 2, 5, 7, 8, 10	5', 7, 11", 12
7 C	50.589			7	6', 8, 9, 11"	
H	3.359	3.00(8), 4.40(6')	6', 8, 11', 11"	7		6', 8, 11', 11"
8 C	135.605			8	6', 9, 11"	
H	5.816	3.00(7), 5.60(9)	7, 9	8	7, 9, 10, 11	7
9 C	133.897			9	5', 8, 11"	
H	5.942	2.90(10), 5.60(8)	8, 10	9	7, 8, 10, 11	10, 14
10 C	47.937			10	5', 6', 8, 9, 11"	
H	2.853	2.90(9), 3.20(5'), 1.60(11')	5', 9, 11', 11"	10		5', 9, 11', 11", 14
11 C	49.592			11', 11"	8, 9	
H'	1.417	1.60(10), 8.50(11")	7, 10	11	5, 6	7, 10
H"	1.354	8.50(11')	7, 10	11	5, 6, 7, 8, 9, 10	5', 6', 7, 10
12 C	31.437			12	2, 3, 5'	
H3	1.414			12	3, 4, 5, 13	3, 5', 6'
13 C	174.818				3, 12, 14	
14 C	52.139			14		
H3	3.818			14	4, 13	9, 10
100 O						
101 O						
102 O						

P-ID: ML00xxx
Measured on: 29/10/2019
CHIFFRE: GHS-GA-607-01
ELNA#: 3326
Client: Dr. Santanu Ghosh
Group: List
Spectroscopist: Leutzsch
Analysed on: 29/10/2019
Analysed by: Leutzsch
Amount: 5 mg
Solvent: CDCl3
Reference: solvent
Temperature: 298 K
Spectrometer: AV500as
Probe: 5 mm PABBO BB-1H/D Z-GRD Z119470/0004
Experiments: 1H-zg30, 13C-zgdc30, [13C, 1H]-hsqcedetgp, [1H, 1H]-noesygpph, [1H, 1H]-clipcosysp.cf, [13C, 1H]-hmbcgpl2ndqf

Table 6. Peak table for **2l′**: COSY, HMBC, HSQC, NOESY signals for the assignment of stereochemistry.

Reaction studies with isoprene:

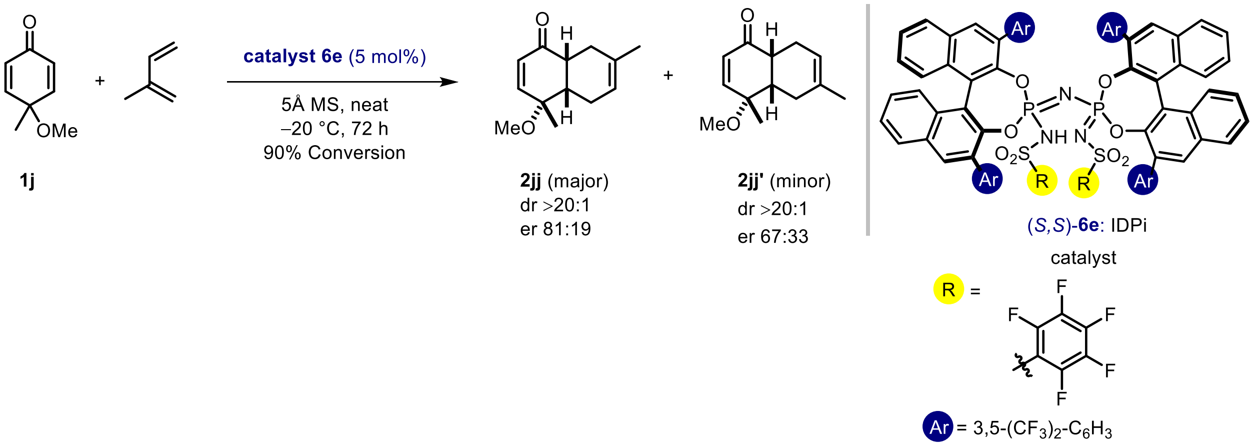

The preliminary studies using catalyst **6d** under similar reaction conditions led to only traces of the desired product. Improved results were obtained when we used catalyst **6e**, which was synthesized using the general procedure mentioned above.

Procedure for the synthesis of 2jj and 2jj′:

A 2 mL oven-dried GC vial was charged with magnetic stir bar, catalyst (5 mol%, 0.05 equiv., 0.0018 mmol), 5Å MS (10 mg). Then the dienone **1j** (1 equiv., 0.04 mmol) was added to the reaction vial and was cooled to –20 °C. After 10 minutes at this temperature, isoprene (15 equiv., 0.54 mmol, 47 μL) was added through the wall of the reaction vial and stirred for 72 h. The reaction was treated with Et_3N (10 μL). After 10 minutes, the reaction mixture was warmed up to rt. The regioisomeric ratio and the conversion was determined by crude ^1H NMR. (conversion 90%; regioisomeric ratio **3:1**); **^{1}H NMR** (500 MHz, $CDCl_3$ for major regioisomer) δ 6.60 (dd, *J* = 10.3, 2.5 Hz, 1H), 5.87 (d, *J* = 10.3 Hz, 1H), 5.32–5.30 (m, 1H), 3.28 (s, 3H), 2.79–2.76 (m, 1H), 2.75–2.70 (m, 1H signal merge with minor regioisomer), 2.54–2.49 (m, 1H), 2.08–1.93 (m, 3H signal merge with minor regioisomer), 1.68 (m, 3H), 1.54 (s, 3H); **^{1}H NMR** (500 MHz, $CDCl_3$ for minor regioisomer) δ 6.62 (dd, *J* = 10.3, 2.4 Hz, 1H), 5.85 (d, *J* = 10.3, Hz, 1H), 5.36-5.34 (m, 1H), 3.30 (s, 3H), 2.85–2.80 (m, 1H), 2.75–2.70 (m, 1H signal merge with major regioisomer), 2.61–2.57 (m, 1H), 2.08–1.93 (m, 3H signal merge with major regioisomer), 1.68 (m, 3H), 1.54 (s, 3H); **^{13}C NMR** (126 MHz, $CDCl_3$ for major regioisomer) δ 199.2, 151.5, 132.3, 127.1, 119.7, 76.6, 49.1, 44.0, 42.4, 29.0, 23.5, 23.4, 21.4; **^{13}C NMR** (126 MHz, $CDCl_3$) δ 199.4, 151.3, 132.6, 127.1, 119.1, 76.5, 49.2, 43.2, 43.0, 28.2, 24.5, 23.8, 21.4; **HRMS** (GC-EI) *m/z* calculated for $C_{13}H_{18}O_2$ $[M]^+$: 206.130130; found 206.130270; The enantiomeric ratios were determined by 2-dimension HPLC on a chiral stationary phase 150 mm 3-AmyCoat RP, 4.6 mm i.D., Säule 6, Acetonitril / Wasser-Gradient:30% - 10' - 50% B 1.0 ml/min, 24.5 MPa, 298 K, UV, 220 nm; t_R (major enantiomer for major regioisomer) = 8.62 min, t_R (minor enantiomer for major regioisomer) = 9.90 min, er = 81:19; t_R (major enantiomer for minor regioisomer) = 7.44 min, t_R (minor enantiomer for minor diastereomer) = 8.79 min, er = 67:33.

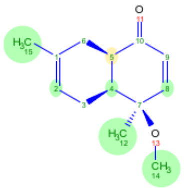

Isomer I
major isomer

Assignments					
**Atom**	**Chemical Shift**	**J**	**HSQC**	**HMBC**	**NOESY**
1 C	132.19			5, 6", 15	
2 C	119.58		2	6', 6", 15	
H	5.37		2	4, 15	3, 15
3 C	23.39		3	4, 5	
H2	2.09		3		2, 4
4 C	42.26		4	2, 5, 6', 8, 12	
H	2.58	2.50(8)	4	3, 5, 6, 10, 12	3, 5, 12, 14
5 C	43.89		5	4, 6'	
H	2.83		5	1, 3, 4, 6, 10	4, 6', 6", 12
6 C	28.90		6', 6"	4, 5, 9, 15	
H'	2.78		6	2, 4, 5	5, 6', 15
H"	2.06		6	1, 2, 10	5, 15
7 C	76.51			12, 14	
8 C	151.39		8	12	
H	6.65	2.50(4), 10.30(9)	8	4, 10, 12	12, 14
9 C	127.01		9	12	
H	5.93	10.30(8)	9	6, 10, 12	
10 C	199.13			4, 5, 6", 8, 9	
11 O					
12 C	21.31		12	4, 8, 9	
H3	1.60		12	4, 7, 8, 9	4, 5, 8, 14
13 O					
14 C	49.01		14		
H3	3.34		14	7	4, 8, 12
15 C	23.34		15	2	
H3	1.74		15	1, 2, 6	2, 6', 6"

P-ID: ML00000
Measured on: 16/02/2019
CHIFFRE: GHS-GA-459-01
ELNA#: 2638
Client: Santanu Ghosh
Group: List
Spectroscopist: Leutzsch
Analysed on: 16/02/2019
Analysed by: Leutzsch
Amount: 20 mg
Solvent: CDCl3
Reference: solvent
Temperature: 298 K
Spectrometer: AV500as
Probe: 5 mm PABBO BB-1H/D Z-GRD Z119470/0004
Experiments: 1H-zg30, 13C-zgdc30, [1H, 1H]-noesygpph, [13C, 1H]-hsqcedetgp, [13C, 1H]-hmbcgpl2ndqf

Table 7. Peak table for **2jj**: HMBC, HSQC, NOESY signals for the assignment of relative stereochemistry.

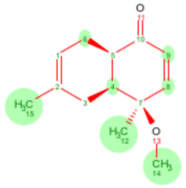

Isomer II
minor isomer

Assignments					
**Atom**	**Chemical Shift**	**J**	**HSQC**	**HMBC**	**NOESY**
1 C	119.01		1	5, 15	
H	5.41		1	5, 15	6', 6", 15
2 C	132.54			3, 6', 15	
3 C	28.06		3	4, 5, 15	
H2	2.01		3	2	4, 5, 15
4 C	42.88		4	6', 8, 12	
H	2.65	2.50(8)	4	3, 5, 10, 12	3, 5, 12
5 C	43.07		5	1, 4, 6', 9	
H	2.78		5	1, 3, 6, 10	3, 4, 12
6 C	24.38		6', 6"	5	
H'	2.89		6	2, 4, 5	1, 6"
H"	2.08		6		1, 6'
7 C	76.45			9, 12, 14	
8 C	151.24		8	12	
H	6.67	2.50(4), 10.35(9)	8	4, 10, 12	14
9 C	127.01		9	12	
H	5.93	10.35(8)	9	5, 7, 10	
10 C	199.27			4, 5, 8, 9	
11 O					
12 C	21.31		12	4, 8	
H3	1.60		12	4, 7, 8, 9	4, 5, 14
13 O					
14 C	49.06		14		
H3	3.36		14	7	8, 12
15 C	23.74		15	1	
H3	1.66		15	1, 2, 3	1, 3

Table 8. Peak table for **2jj′**: HMBC, HSQC, NOESY signals for the assignment of relative stereochemistry.

Functionalization of Products

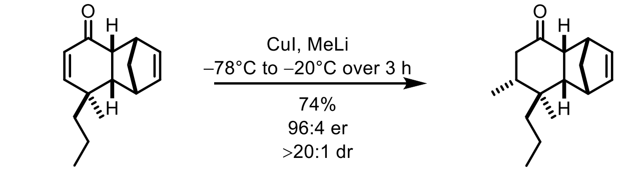

2b
major dr
(separated by
column chromatography)
96:4 er

7

Procedure for the synthesis of (1*R*,4*S*,4a*R*,7*R*,8*S*,8a*S*)-7,8-dimethyl-8-propyl-4,4a,6,7,8,8a-hexahydro-1,4-methanonaphthalen-5(1*H*)-one 7: A flame-dried Schlenk flask equipped with a teflon coated magnetic stirring bar was cooled under argon and charged with CuI (2.0 equiv., 0.314 mmol, 60 mg) in Et_2O (0.4 mL) and placed at 0 °C temperature bath. A solution of MeLi (1.6M in Et_2O) (4.0 equiv., 0.628 mmol, 0.393 mL) was added to the reaction mixture dropwise over 5 minutes and the mixture was allowed to stir for 30 minutes at the same temperature. Afterwards the reaction flask was cooled to −78 °C, and the solution of **2b** (major diastereomer **2b** was separated by column chromatography) (34 mg, 0.157 mmol, 1.0 equiv.) in anhydrous Et_2O (0.5 ml) was added slowly over 30 minutes. Then the bath temperature was slowly warmed to −20 °C over 2 h and the reaction mixture was quenched with saturated aqueous NH_4Cl solution (1 mL) and diluted with Et_2O (10 mL). The organic layer was separated and the aqueous layer was washed with Et_2O (2x10 mL). The combined organic layer was dried over anhydrous Na_2SO_4. The crude product was purified by column chromatography using 12% Et_2O in hexane as eluent and afforded the desired product as colorless oil (27 mg, 74% as single diastereomer). **^{1}H NMR** (600 MHz, $CDCl_3$) δ 6.17 (dd, *J* = 5.7, 3.0 Hz, 1H), 5.92 (dd, *J* = 5.7, 2.9 Hz, 1H), 3.12 (m, 1H), 2.89 (dd, *J* = 11.5, 3.8 Hz, 1H), 2.87–2.85 (m, 1H), 2.74 (dd, *J* = 11.5, 2.9 Hz, 1H), 2.26 (dd, *J* = 19.1, 6.3 Hz, 1H), 2.08–2.01 (m, 1H), 1.80 (dd, *J* = 19.1, 12.2 Hz, 1H), 1.47–1.39 (m, 2H), 1.39–1.29 (m, 4H), 0.95 (t, *J* = 6.9 Hz, 3H), 0.81 (d, *J* = 6.7 Hz, 3H), 0.35 (s, 3H); **^{13}C NMR** (151 MHz, $CDCl_3$) δ 214.9, 136.1, 134.9, 53.2, 50.9, 49.5, 44.6, 44.5, 43.4, 43.3, 38.2, 34.7, 17.8, 16.8, 15.2, 14.4; **HRMS** (GC-EI) *m/z* calculated for $C_{16}H_{24}O$ $[M]^+$: 232.182165; found 232.182280; The

enantiomeric ratio was determined by HPLC on a chiral stationary phase. **HPLC** Daicel Chiralcel IC-3, ^{i}PrOH/heptane = 1/99, 1 mL/min, 25 °C, 254 nm, t_R (major) = 10.2 min, t_R (minor) = 10.79 min, er = 96:4; $[\alpha]_D^{20}$ = –126.4 (*c* 0.22, $CHCl_3$).

Suggested relative stereoconfiguration from 2D NMR measurements

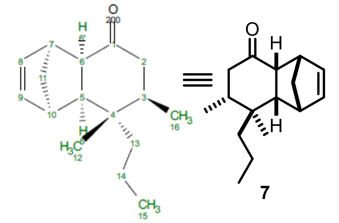

Chem3D model showing important NOEs for stereoassignment

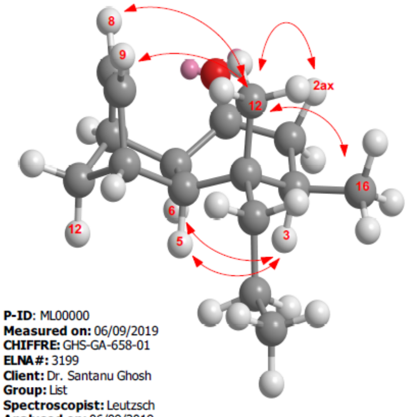

P-ID: ML00000
Measured on: 06/09/2019
CHIFFRE: GHS-GA-658-01
ELNA#: 3199
Client: Dr. Santanu Ghosh
Group: List
Spectroscopist: Leutzsch
Analysed on: 06/09/2019
Analysed by: Leutzsch
Amount: 4 mg
Solvent: CDCl3
Reference: solvent
Temperature: 298 K
Spectrometer: spect
Probe: Z44896_0147 (CP TCI 600S3 H-C/N-D-05 Z)
Experiments: 1H-zg30, 13C-zgdc30, [1H, 1H]-cosygpmfphpp, [1H, 1H]-noesygpph, 13C-deptsp135, [13C, 1H]-ASAP_hsqcedetgpsp, [13C, 1H]-hmbcetgpl3nd

User Report
GHS-GA-658-01

Assignments						
Atom	Chemical Shift	J	COSY	HSQC	HMBC	NOESY
1 C	214.707				2ax, 2eq, 5', 6', 16	
2 C	44.382			2ax, 2eq	3, 5', 16	
Hax	1.796	12.20(3), 19.10(2eq)	2eq, 3	2	1, 3, 6, 16	12, 16
Heq	2.258	6.40(3), 19.10(2ax)	2ax, 3	2	1, 3, 4	16
3 C	34.508			3	2ax, 2eq, 12, 16	
H	2.043	6.40(2eq), 6.50(16), 12.20(2ax)	2ax, 2eq, 16	3	2, 4, 5, 12, 16	5', 6', 13', 13", 14', 14", 16
4 C	38.034				2eq, 3, 5', 12, 13', 13", 14', 14", 16	
5 C	49.369			5'	3, 6', 10, 12, 13', 13"	
5' H	2.740	2.90(10), 11.50(6')	6'	5	1, 2, 4, 6, 7, 9, 12	3, 6', 11', 13', 13", 14', 14"
6 C	53.043			6'	2ax, 5', 10	
6' H	2.888	3.80(7), 11.50(5')	5', 7	6	1, 5, 8, 13	3, 5', 7, 11'
7 C	43.241			7	5', 8, 9	
H	3.122	3.80(6')	6', 8, 11', 11"	7		6', 8, 11', 11"
8 C	135.899			8	6', 9, 11', 11"	
H	6.166	5.70(9)	7, 9	8	7, 9, 11	7, 12
9 C	134.737			9	5', 8, 11', 11"	
H	5.916	5.70(8)	8	9	7, 8, 11	10, 12
10 C	44.412			10		
H	2.858	2.90(5')	11', 11"	10	5, 6	9, 11', 11", 12, 13', 13", 14', 14"

Assignments						
Atom	Chemical Shift	J	COSY	HSQC	HMBC	NOESY
11 C	50.738			11', 11"	8, 9	
H'	1.308	8.20(11")	7, 10	11	8, 9	5', 6', 7, 10
H"	1.451	8.20(11')	7, 10	11	8, 9	7, 10
12 C	17.641			12	3, 5', 13', 13"	
H3	0.345			12	3, 4, 5, 13	2ax, 8, 9, 10, 13', 13", 14', 14", 16
13 C	43.101			13', 13"	6', 12, 14', 14", 15	
H'	1.391		13", 14', 14"	13	4, 5, 12, 14, 15	3, 5', 10, 12, 16
H"	1.340		13', 14', 14"	13	4, 5, 12, 14, 15	3, 5', 10, 12, 16
14 C	16.605			14', 14"	13', 13", 15	
H'	1.431		13', 13", 15	14	4, 13, 15	3, 5', 10, 12
H"	1.345		13', 13", 15	14	4, 13, 15	3, 5', 10, 12
15 C	15.030			15	13', 13", 14', 14"	
H3	0.947		14', 14"	15	13, 14	
16 C	14.272			16	2ax, 3	
H3	0.807	6.50(3)	3	16	1, 2, 3, 4	2ax, 2eq, 3, 12, 13', 13"
200 O						

Table 9. Peak table for **7**: COSY, HMBC, HSQC, NOESY signals for the assignment of stereochemistry.

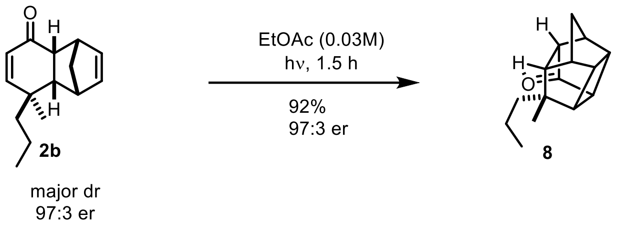

Procedure for the synthesis of (2*R*)-2-methyl-2-propyloctahydro-1H-3,5,1-(epiethane[1,1,2]triyl)cyclobuta[cd]pentalen-7-one 8: A microwave vial was charged with enantiopure enone **2b** (1.0 equiv., 0.171 mmol, 37 mg) in EtOAc (6 mL). The reaction vial was degassed with argon by freeze and thaw method (3 times). Then the reaction vial was placed in a

closed shell having a UV lamp (310 to 380 nm) and was stirred for 1.5 h. The reaction mixture was evaporated under reduced pressure to afford compound **8** (34 mg, 92% as single diastereomer). **^{1}H NMR** (501 MHz, $CDCl_3$) δ 2.99–2.94 (m, 1H), 2.79 (m, 1H), 2.65 (dtt, *J* = 8.8, 5.6, 1.6 Hz, 1H), 2.54–2.46 (m, 3H), 2.38–2.30 (m, 2H), 1.79 (dt, *J* = 10.8, 1.6 Hz, 1H), 1.49 (dt, *J* = 10.9, 1.7 Hz, 1H), 1.42–1.36 (m, 2H), 1.35–1.16 (m, 2H), 0.85 (t, *J* = 7.2 Hz, 3H), 0.81 (s, 3H); **^{13}C NMR** (126 MHz, $CDCl_3$) δ 220.0, 57.8, 51.4, 47.0, 46.8, 44.8, 43.3, 42.8, 42.4, 37.3, 36.6, 35.0, 24.7, 19.3, 14.8; **HRMS** (GC-EI) *m/z* calculated for $C_{15}H_{20}O$ $[M]^+$: 216.150865; found 216.150740; The enantiomeric ratio was determined by GC on a BGB-176IC-3 chiral column, 0.50 bar H_2 gas pressure, t_R (minor) = 122.03 min, t_R (major) = 125.60 min, er = 3:97; $[\alpha]_D^{20}$= –23.0 (*c* 0.235, $CHCl_3$).

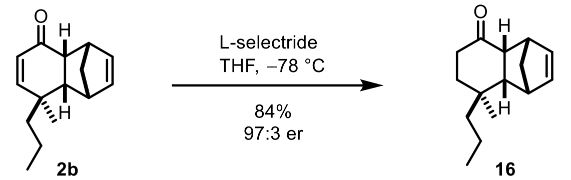

(1*R*,4*S*,4a*R*,8*S*,8a*S*)-8-methyl-8-propyl-4,4a,6,7,8,8a-hexahydro-1,4-methanonaphthalen-5(1H)-one 16: A flame dried Schlenk flask equipped with a teflon coated magnetic stirring bar was charged with enantiopure enone **2b** (1.0 equiv., 0.093 mmol, 20 mg) in anhydrous THF (0.3 ml) at –78 °C. To that reaction mixture a solution of L-selectride (1M in THF) (1.2 equiv., 0.11 mmol, 0.12 mL) was added dropwise. The clear solution was stirred at the same temperature for 2 h (reaction was monitor by TLC). Then the reaction mixture was quenched with aqueous sat. NH_4Cl (1 mL) and diluted with Et_2O (5 mL). The organic layer was separated and aqueous phase was extracted with Et_2O (2 x 5 mL), then the combined organic layer was washed with brine and dried over anhydrous Na_2SO_4. The crude product was purified by flash column chromatography using 5% (EtOAc/hexane) as eluents and afforded the saturated ketone (17 mg, 84%). **^{1}H NMR** (501 MHz, $CDCl_3$) δ 6.15 (dd, *J* = 5.7, 2.9 Hz, 1H), 6.08 (dd, *J* = 5.7, 2.9 Hz, 1H), 3.23 (m, 1H), 2.98 (m, 1H), 2.84 (ddt, *J* = 9.7, 4.4, 1.2 Hz, 1H), 2.32 (ddd, *J* = 9.7, 3.2, 1.5 Hz, 1H), 2.12 (dddd, *J* = 18.9, 10.2, 7.3, 1.3 Hz, 1H), 1.99 (dddd, *J* = 19.1, 6.3, 4.3, 0.9 Hz, 1H), 1.51 (ddd, *J* = 14.0, 10.2, 6.3 Hz, 1H), 1.44–1.37 (m, 2H), 1.35–1.22 (m, 5H), 0.91 (m, 3H), 0.88 (s, 3H); **^{13}C NMR** (126 MHz, $CDCl_3$) δ 215.4, 135.5, 51.0, 50.8, 50.5, 47.3, 46.1, 45.2, 35.6, 34.1, 29.8, 24.7, 16.7, 15.0 (one signal missing due to overlap); **HRMS** (GC-EI) *m/z* calculated for $C_{15}H_{22}O$ $[M]^+$: 218.166515;

found 218.166520. The enantiomeric ratio was determined by HPLC on a chiral stationary phase. HPLC Daicel Chiralcel IC-3, ^{i}PrOH/heptane = 5/95, 1 mL/min, 25 °C, 254 nm, t_R (major) = 5.76 min, t_R (minor) = 6.7 min, er = 97:3; $[\boldsymbol{\alpha}]_{\boldsymbol{D}}^{\mathbf{20}}$ = –84.3 (*c* 0.32, $CHCl_3$).

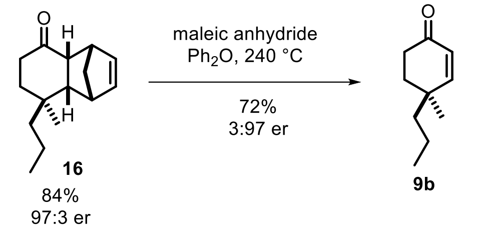

Procedure for the synthesis of (*S*)-4-methyl-4-propylcyclohex-2-en-1-one 9b: A flame-dried Schlenk flask under argon was charged with enantiopure ketone **16** (1.0 equiv., 0.055 mmol, 12 mg) and maleic anhydride (2.0 equiv., 0.11 mmol, 11 mg) in Ph_2O (1 mL). Then the reaction mixture was heated at 240 °C for 2 h. The reaction mixture was cooled to rt. The crude product was purified by column chromatography on silica gel using (5 to 10%) Et_2O in hexane as eluents to afford the desired product as colorless oil (6 mg, 72%). **^{1}H NMR** (501 MHz, $CDCl_3$) δ 6.68 (dt, *J* = 10.2, 0.8 Hz, 1H), 5.86 (d, *J* = 10.2 Hz, 1H), 2.50–2.40 (m, 2H), 1.96 (ddd, *J* = 13.6, 8.8, 5.9 Hz, 1H), 1.76 (dddd, *J* = 13.7, 6.9, 5.7, 1.1 Hz, 1H), 1.48–1.29 (m, 4H), 1.12 (s, 3H), 0.93 (t, *J* = 7.1 Hz , 3H); **^{13}C NMR** (126 MHz, $CDCl_3$) δ 200.0, 159.7, 127.4, 43.5, 35.8, 34.3, 33.7, 25.0, 17.6, 14.9; **HRMS (GC-EI)** *m/z* calculated for $C_{10}H_{16}O$ $[M]^+$: 152.119565; found 152.119700; The enantiomeric ratio was determined by GC, using chiral column: 30 m, Cyclosil B, at temperature:220/135 30 min iso 12/min 220 5min iso/350, Gas: 0.50 bar H_2, t_R (minor) = 23.20 min, t_R (major) = 23.87 min, er = 3:97; $[\boldsymbol{\alpha}]_{\boldsymbol{D}}^{\mathbf{25}}$ = –34.7 (*c* 0.075, MeOH);

[**NOTE**: The sense of absolute configuration was confirmed from the literature report[2b] of (–)-**9b** $[\boldsymbol{\alpha}]_{\boldsymbol{D}}^{\mathbf{23}}$ = –47.55 ±0.61 (*c* 1.13 mg/ml in MeOH) for er >99:1]

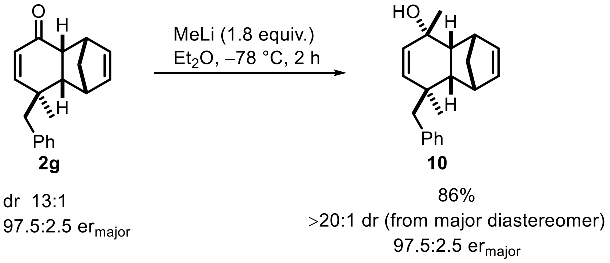

Procedure for the synthesis of (1*R*,4*S*,4a*R*,5*S*,8*S*,8a*S*)-8-benzyl-5,8-dimethyl-1,4,4a,5,8,8a-hexahydro-1,4-methanonaphthalen-5-ol 10: A flame-dried Schlenk flask equipped with a teflon

coated magnetic stirring bar was cooled under argon and charged with enantiopure compound **2g** (97.5:2.5 er_{major} and 13:1 dr) (1.0 equiv., 0.0794 mmol, 21 mg) in Et_2O (0.265 mL) at −78 °C. Then MeLi (1.6 M in Et_2O) (1.8 equiv., 0.143 mmol, 0.0894 mL) was added dropwise to the solution and was stirred for 2 h at the same temperature. After that the reaction mixture was slowly warmed up to 0 °C and quenched by careful addition of H_2O (2 mL) and diluted with Et_2O (5 mL). The organic layer was collected, dried over anhydrous $MgSO_4$ and concentrated under reduced pressure. The crude product was purified by preparative TLC Alox 25 mm, using 10% EtOAc in hexane as eluents to afford the desired product as yellow oil (19 mg, 86%, product contained ~5% unreacted starting material which was inseparable, obtained product as same diastereomeric ratio dr 16:1) [Note: The Methyllithium addition to enone carbonyl is highly diastereoselective dr >20:1 (from major dr)] **^{1}H NMR** (501 MHz, $CDCl_3$, spectra contained two diastereomers 16:1) δ 7.28–7.23 (m, 2H), 7.23–7.20 (m, 1H), 7.13–7.11 (m, 2H), 5.98 (dd, *J* = 5.6, 2.8 Hz, 1H), 5.94 (dd, *J* = 5.7, 2.9 Hz, 1H), 5.43 (dd, *J* = 10.2, 0.6 Hz, 1H), 4.99 (dd, *J* = 10.3, 1.0 Hz, 1H), 3.12 (m, 1H), 2.86–2.78 (m, 3H), 2.52–2.47 (m, 2H), 1.50 (s, 3H), 1.41 (br, 1H), 1.33 (dt, *J* = 8.1, 2.0 Hz, 1H), 1.29 (dtt, *J* = 7.5, 1.5, 0.8 Hz, 1H), 0.97 (s, 3H). **^{13}C NMR** (126 MHz, $CDCl_3$) δ 138.3, 135.8, 135.3, 135.2, 134.3, 131.0, 127.8, 126.2, 69.8, 52.3, 51.0, 48.5, 47.3, 47.3, 36.6, 33.9, 29.9, 25.4; **HRMS** (ESI) calculated for $C_{20}H_{24}ONa$ $[M+Na]^+$: 303.171934; found 303.171670; The enantiomeric ratio was determined by HPLC on a chiral stationary phase. **HPLC** Daicel Chiralcel AS-3, ^{i}PrOH/heptane = 3/97, 1 mL/min, 25 °C, 254 nm, t_R (minor) = 3.73 min, t_R (major) = 4.2 min, er = 2.5:97.5.

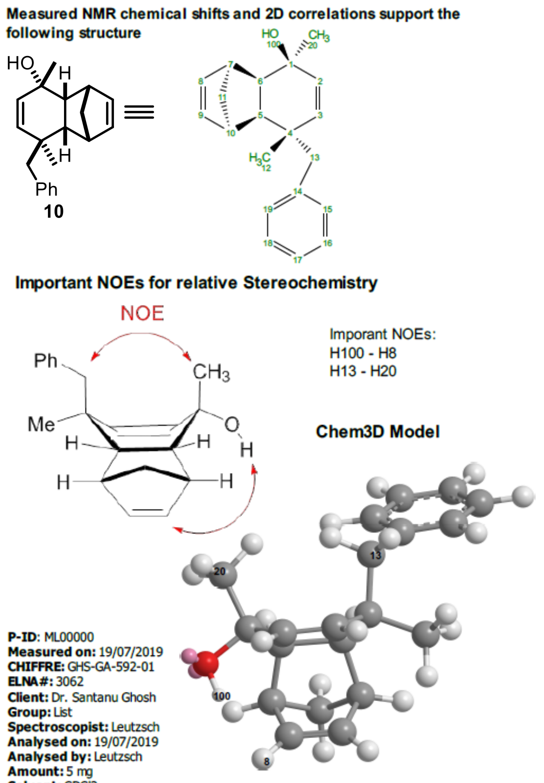

User Report
GHS-GA-592

Assignments						
Atom	Chemical Shift	J	COSY	HSQC	HMBC	NOESY
1 C	69.680				3, 5, 6, 20, 100	
2 C	134.117			2	20, 100	
H	5.428	0.60(6), 10.30(3)	3, 6	2	4, 6, 13, 20	3, 20, 100
3 C	135.182			3	12, 13', 13"	
H	4.985	10.30(2), 1.00(5)	2, 5	3	1, 4, 5, 12	2, 12, 13", 15, 19
4 C	36.476				2, 3, 12, 13', 13"	
5 C	48.345			5	3, 10, 11', 11", 12, 13', 13"	
H	2.487	9.80(6), 1.00(3)	3	5	1, 6, 10, 13	6, 11'
6 C	50.849			6	2, 5, 11', 11", 20, 100	
H	2.831	0.60(2), 9.80(5)	2	6	1, 7, 20	5, 7, 11', 12, 20
7 C	47.131			7	6, 8, 9, 11'	
H	3.119	2.80(8)	8, 10, 11', 11"	7		6, 8, 11', 11", 100
8 C	135.623			8	11'	
H	5.983	2.80(7), 5.60(9)	7, 9	8	7, 10, 11	7, 100
9 C	135.089			9	11'	
H	5.943	2.80(10), 5.60(8)	8, 10	9	7, 10, 11	10, 12
10 C	47.169			10	5, 8, 9, 11'	
H	2.837	2.80(9)	7, 9, 11', 11"	10	5	9, 11', 11", 12
11 C	49.402			11', 11"	8, 9	
H'	1.288		7, 10	11	5, 6, 7, 8, 9, 10	5, 6, 7, 10
H"	1.333		7, 10	11	5, 6	7, 10

Assignments						
Atom	Chemical Shift	J	COSY	HSQC	HMBC	NOESY
12 C	25.284			12	3, 13', 13"	
H3	0.972		13"	12	3, 4, 5, 13	3, 6, 9, 10, 13', 13", 15, 19
13 C	52.181			13', 13"	2, 5, 12, 15	
H'	2.499		13"	13	3, 4, 5, 12, 15, 19	12, 13", 15, 19, 20
H"	2.805		12, 13'	13	3, 4, 5, 12, 15, 19	3, 12, 13', 15, 19, 20
14 C	138.142				16, 18	
15 C	130.849			15	13', 13", 17, 19	
H	7.119			15	13, 17, 19	3, 12, 13', 13", 20
16 C	127.633			16	18	
H	7.251			16	14, 18	
17 C	126.078			17	15, 19	
H	7.211			17	15, 19	
18 C	127.633			18	16	
H	7.251			18	14, 16	
19 C	130.849			19	13', 13", 15, 17	
H	7.119			19	15, 17	3, 12, 13', 13", 20
20 C	33.778			20	2, 6	
H3	1.504			20	1, 2, 6	2, 6, 13', 13", 15, 19
100 O						
H	1.411				1, 2, 6	2, 7, 8

P-ID: ML00000
Measured on: 19/07/2019
CHIFFRE: GHS-GA-592-01
ELNA#: 3062
Client: Dr. Santanu Ghosh
Group: List
Spectroscopist: Leutzsch
Analysed on: 19/07/2019
Analysed by: Leutzsch
Amount: 5 mg
Solvent: CDCl3
Reference: solvent
Temperature: 298 K
Spectrometer: AV500as
Probe: 5 mm PABBO BB-1H/D Z-GRD Z119470/0004
Experiments: 1H-zg30, 13C-zgdc30, [13C, 1H]-ASAP_hsqcedetgpsp, [1H, 1H]-cosygpqf, [1H, 1H]-noesygpph, [13C, 1H]-hmbcgpl2ndqf

Table 10. Peak table for **10**: COSY, HSQC, HMBC, NOESY signals for the assignment of stereochemistry.

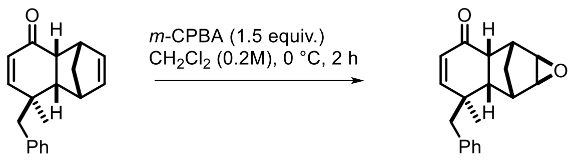

2g
13:1 dr
97.5:2.5 er_{major}

11
82%
>20:1 dr from major dr
98:2 er_{major}

Procedure for the synthesis of (1a*R*,2*R*,2a*S*,6*S*,6a*S*,7*S*,7a*S*)-6-benzyl-6-methyl-2,2a,6,6a,7,7a-hexahydro-2,7-methanonaphtho[2,3-b]oxiren-3(1aH)-one 11: A flame-dried Schlenkflask equipped with a teflon coated magnetic stirring bar was cooled under argon and charged with compound **2g** with (dr13:1, er_{major}: 97.5:2.5) (1.0 equiv., 0.076 mmol, 20 mg) in CH_2Cl_2 (0.38 mL) at 0 °C. Then a solution of *m*CPBA (1.5 equiv., 0.1135 mmol, 25 mg) in CH_2Cl_2 (0.2 mL) was added dropwise to the solution and stirring was continued for 2 h. After that the reaction mixture was quenched by using saturated $NaHCO_3$ aqueous solution (5 mL) and diluted with CH_2Cl_2 (5

mL). The organic layer was collected, dried over anhydrous $MgSO_4$ and concentrated under reduced pressure. The crude product was purified by preparative TLC Alox 25 mm, using 10% EtOAc in hexane as eluents and afforded the desired product as yellow oil (17 mg, 82% contained dr 13:1); [Note: The epoxidation of olefinic double bond is highly diastereoselective dr>20:1 (from major dr)] **^{1}H NMR** (500 MHz, $CDCl_3$) δ 7.23–7.16 (m, 3H), 6.97–6.95 (m, 2H), 6.41 (dd, J = 10.2, 1.6 Hz, 1H), 5.99 (d, J = 10.1 Hz, 1H), 2.95 (d, J = 3.4 Hz, 1H), 2.83–2.79 (m, 2H), 2.67–2.64 (m, 2H), 2.49 (d, J = 13.0 Hz, 1H), 2.38 (ddd, J = 9.5, 3.5, 1.6 Hz, 1H), 2.30 (dd, J = 9.5, 5.0 Hz, 1H), 1.39 (dt, J = 10.0, 1.9 Hz, 1H), 1.28 (s, 3H), 0.71 (d, J = 10.0 Hz, 1H); **^{13}C NMR** (126 MHz, $CDCl_3$) δ 200.1, 156.6, 136.1, 131.1, 130.5, 128.3, 127.1, 52.9, 50.7, 50.2, 47.9, 46.9, 43.5, 42.4, 38.2, 26.7, 25.8; **HRMS** (ESI) *m/z* calculated for $C_{19}H_{20}O_2Na$ $[M+Na]^+$: 303.135549; found 303.135650; for the diastereomeric mixture; The enantiomeric ratio was determined by HPLC on a chiral stationary phase. **HPLC** Daicel Chiralcel IA, ^{i}PrOH/heptane = 5/95, 1 mL/min, 25 °C, 254 nm, t_R (minor) = 16.01 min, t_R (major) = 20.88 min, er_{major} = 2:98.

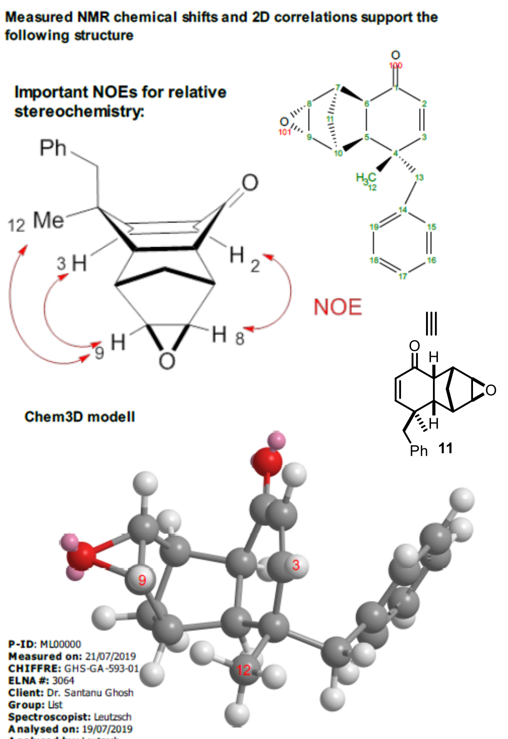

P-ID: ML00000
Measured on: 21/07/2019
CHIFFRE: GHS-GA-593-01
ELNA #: 3064
Client: Dr. Santanu Ghosh
Group: List
Spectroscopist: Leutzsch
Analysed on: 19/07/2019
Analysed by: Leutzsch
Amount: 4 mg
Solvent: CDCl3
Reference: solvent
Temperature: 298 K
Spectrometer: AV500as
Probe: 5 mm PABBO BB-1H/D Z-GRD Z119470/0004
Experiments: 1H-zg30, [13C, 1H]-ASAP_hsqcedetgpsp, 13C-zgdc30, [1H, 1H]-cosygpqf, [1H, 1H]-noesygpph, [13C, 1H]-hmbcgpl2ndqf

User Report
GHS-GA-593

Atom	Chemical Shift	J	COSY	HSQC	HMBC	NOESY
1 C	199.989				3, 5, 6	
2 C	130.351			2	6, 12	
H	6.058	10.10(3)		2	4, 6, 13	3, 8
3 C	156.447			3	5, 12, 13', 13''	
H	6.475	1.50(5), 10.10(2)	5	3	1, 4, 5, 12	2, 9, 12, 13', 13''
4 C	38.033				2, 3, 5, 12, 13', 13''	
5 C	47.763			5	3, 6, 7, 11', 11'', 12, 13', 13''	
H	2.447	9.50(6), 3.60(10), 1.50(3)	3, 6, 10	5	1, 3, 4, 6, 9, 10, 13	11', 12, 13', 13'', 15, 19
6 C	46.777			6	2, 5, 10, 11', 11''	
H	2.365	9.50(5)	5, 7	6	1, 2, 5, 7, 8, 10	7, 11', 15, 19
7 C	43.372			7	6, 8, 11'	
H	2.890		6, 11', 11''	7	5, 9, 10	6, 11', 11''
8 C	50.571			8	6, 9, 10, 11'	
H	2.864	0.60(11'), 3.50(9)	9	8	7, 9, 11	2, 9
9 C	50.092			9	5, 7, 8, 11'	
H	3.020	3.50(8), 0.60(11')	8	9	8, 10, 11	3, 8, 10, 12
10 C	42.225			10	5, 6, 7, 9, 11'	
H	2.709	3.60(5)	5, 11', 11''	10	6, 8	9, 11', 11''
11 C	26.519			11', 11''	8, 9	
H'	0.774	9.90(11''), 0.60(8), 0.60(9)	7, 10	11	5, 6, 7, 8, 9, 10	5, 6, 7, 10, 11''
H''	1.459	9.90(11')	7, 10	11	5, 6	7, 10, 11'
12 C	25.679			12	3, 13', 13''	
H3	1.345			12	2, 3, 4, 5, 13	3, 5, 9, 13', 13'', 15, 19
13 C	52.713			13', 13''	2, 5, 12, 19	
H'	2.561	13.00(13'')	13''	13	3, 4, 5, 12, 14, 15, 19	3, 5, 12, 15, 19
H''	2.728	13.00(13')	13'	13	3, 4, 5, 12, 14, 15, 19	3, 5, 12, 15, 19
14 C	135.914				13', 13'', 15, 16, 18, 19	
15 C	130.968			15	13', 13'', 17, 19	
H	7.032			15	14, 17, 19	5, 6, 12, 13', 13''
16 C	128.132			16	18	
H	7.244			16	14, 18	
17 C	126.985			17	15, 19	
H	7.244			17	15, 19	
18 C	128.132			18	16	
H	7.244			18	14, 16	
19 C	130.968			19	13', 13'', 15, 17	
H	7.032			19	13, 14, 15, 17	5, 6, 12, 13', 13''
100 O						
101 O						

Table 11. Peak table for **11:** COSY, HMBC, HSQC, NOESY signals for the assignment of stereochemistry.

Absolute Stereochemistry Assignment

The assignment of relative stereochemistry for the diastereomers **2g** and **2g′** was conducted by COSY, HMBC, HSQC, NOESY NMR analysis (see page **14-16**). The major and minor diastereomer for the catalyzed Diels–Alder reaction were assigned as the *endo*-product. The absolute configuration of **2g** was determined by converting to the corresponding Mosher's ester derivatives **17a** and **17b** as described below and the relative configuration of **2a-h** were assigned by analogy. The absolute configuration of the minor diastereomer was assigned accordingly.

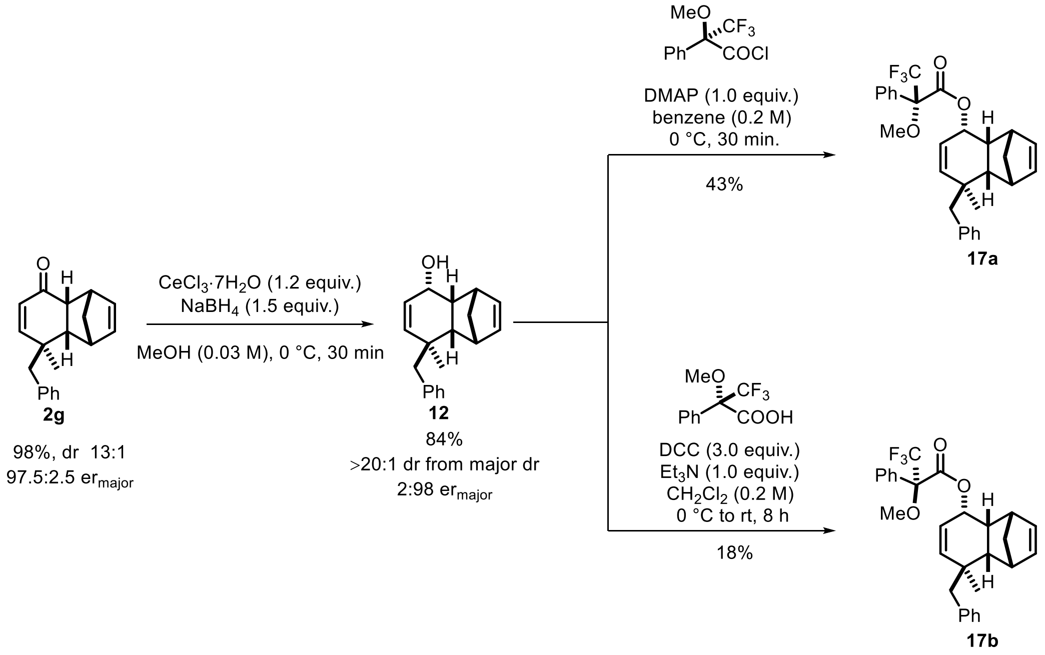

Procedure for the synthesis of (1*R*,4*S*,4a*R*,5*S*,8*S*,8a*S*)-8-benzyl-8-methyl-1,4,4a,5,8,8a-hexahydro-1,4-methanonaphthalen-5-ol 12: A Schlenk flask equipped with a teflon coated

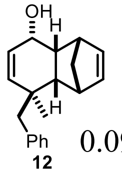
12

magnetic stirring bar was charged with compound **2g** (97.5:2.5 er$_{major}$ and dr 13:1) (1.0 equiv., 0.076 mmol, 20 mg) in MeOH (2.53 mL) at 0 °C. Then $CeCl_3.7H_2O$ (1.2 equiv., 0.091 mmol, 34 mg) was added and followed by the addition of $NaBH_4$ (1.5 equiv., 0.1135 mmol, 4.2 mg). Then the reaction mixture was allowed to stir for 30 minutes (TLC showed complete consumption of starting materials) and quenched by the addition of H_2O (5 mL), diluted with CH_2Cl_2 (10 mL). The organic layer was collected, dried over anhydrous $MgSO_4$ andconcentrated under reduced pressure. The crude product was purified by preparative TLC Alox

25 mm, using 10% EtOAc in hexane as eluent to afford the desired product as yellow oil (17 mg, 84%, dr 13:1). [Note: The reduction of enone carbonyl is highly diastereoselective dr >20:1 (from major dr)] ^{1}**H NMR** (501 MHz, $CDCl_3$) δ 7.25–7.19 (m, 3H), 7.11–7.09 (m, 2H), 5.85 (dd, *J* = 5.6, 2.8 Hz, 1H), 5.76 (dd, *J* = 5.7, 2.8 Hz, 1H), 4.96 (ddd, *J* = 10.1, 3.1, 1.2 Hz, 1H), 4.17 (d, *J* = 9.0 Hz, 1H), 3.01 (m, 1H), 2.94 (td, *J* = 9.2, 3.9 Hz, 1H), 2.86 (m, 1H), 2.70 (d, *J* = 12.8 Hz, 1H), 2.45 (ddd, *J* = 9.5, 3.5, 1.3 Hz, 1H), 2.39 (d, *J* = 12.9 Hz, 1H), 1.39 (br, 1H), 1.31 (m, 2H), 1.02 (s, 3H); ^{13}**C NMR** (126 MHz, $CDCl_3$) δ 138.4, 135.6, 135.3, 134.7, 131.6, 131.0, 127.8, 126.2, 67.1, 50.1, 49.4, 47.1, 46.9, 45.5, 44.7, 37.9, 25.5; **HRMS** (ESIpos) *m/z* calculated for $C_{19}H_{22}ONa$ $[M+Na]^+$: 289.156284; found 289.156160; The enantiomeric ratio was determined by HPLC on a chiral stationary phase. **HPLC** Daicel Chiralcel OJ-3, ^{i}PrOH/heptane = 3/97, 1 mL/min, 25 °C, 254 nm, t_R (minor) = 8.99 min, t_R (major) = 11.95 min, er = 2:98 (from the major diastereomer **2g**)

Procedure for the synthesis of (1*R*,4*S*,4a*R*,5*S*,8*S*,8a*S*)-8-benzyl-8-methyl-1,4,4a,5,8,8a-hexahydro-1,4-methanonaphthalen-5-yl (*R*)-3,3,3-trifluoro-2-methoxy-2-phenylpropanoate

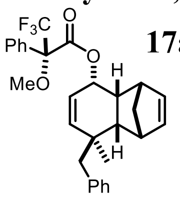

17a

17a: A flame dried Schlenk flask equipped with a teflon coated magnetic string bar was cooled under argon and was charged with compound **12** (er_{major} = 98:2, dr 13:1) (1.0 equiv., 0.038 mmol, 10 mg) in benzene (0.1 mL) at 0 °C. Afterwards DMAP (1.0 equiv., 0.038 mmol, 5 mg) and (*S*)-3,3,3-trifluoro-2-methoxy-2-phenylpropanoyl chloride (1.0 equiv., 0.038 mmol, 10 mg) were added to the solution, and the reaction mixture was stirred for 30 minutes. Then the reaction mixture was filtered through a short anhydrous $MgSO_4$ pad. The crude mixture was collected and purified by preparative TLC (Alox, 25 mm) using 10% EtOAc in hexane and afforded the desired product as colorless oil (43%, dr 14:1); ^{1}**H NMR** (501 MHz, $CDCl_3$) δ 7.58 (dd, *J* = 6.7, 3.1 Hz, 2H), 7.45–7.41 (m, 3H), 7.28–7.20 (m, 3H), 7.13–7.11 (m, 2H), 5.73 (dd, *J* = 5.6, 2.8 Hz, 1H), 5.64 (dd, *J* = 5.6, 2.8 Hz, 1H), 5.37 (dd, *J* = 10.3, 1.7 Hz, 1H), 5.31 (ddd, *J* = 9.5, 3.1, 1.9 Hz, 1H), 5.13 (ddd, *J* = 10.3, 3.0, 1.3 Hz, 1H), 3.60 (m, 3H), 3.08 (td, *J* = 9.4, 3.9 Hz, 1H), 2.84 (br, 1H), 2.71 (d, *J* = 12.9 Hz, 1H), 2.63 (br, 1H), 2.49–2.43 (m, signal merge, 2H), 1.26–1.23 (m, 2H), 1.05 (s, 3H); ^{13}**C NMR** (126 MHz, $CDCl_3$, 76.99 ppm for ^{13}C of $CDCl_3$) δ 166.1, 137.6, 136.7, 135.5, 134.8, 132.5, 130.9, 129.6, 128.4, 127.8 , 127.3, 126.3, 84.4, 73.5, 55.4, 50.4, 48.9, 47.0, 46.5, 46.0, 41.2, 37.7, 25.2 (two signals are overlapped); ^{19}**F NMR** (470 MHz, $CDCl_3$) δ −70.99 (s, CF_3).

Suggested structure

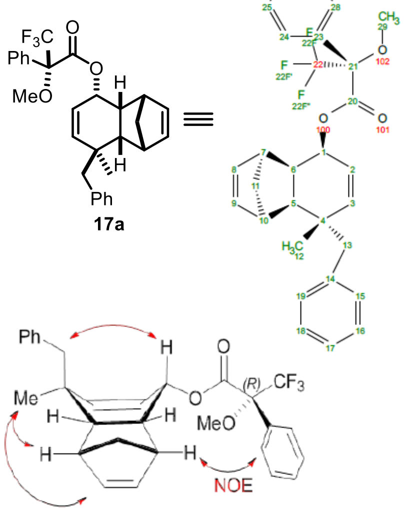

17a

User Report
GHS-GA-612-01

Assignments					
Atom	Chemical Shift	COSY	HSQC	HMBC	NOESY
1 C	73.497		1	3, 6	
H	5.314	2, 3, 6	1	2, 3, 6, 7, 20	6, 13', 13'', 15, 19, 29
2 C	126.296		2	1, 6, 12	
H	5.374	1, 3	2	4, 6, 13	8, 24, 28, 29
3 C	136.728		3	1, 5, 12, 13', 13''	
H	5.126	1, 2, 5	3	1, 4, 5, 12	9, 12, 15, 19
4 C	37.695			2, 3, 5, 12, 13', 13''	
5 C	46.501		5	3, 6, 11, 12, 13', 13''	
H	2.458	3, 6, 10	5	3, 4, 9, 10, 13	6, 10, 11
6 C	41.172		6	1, 2, 11	
H	3.080	1, 5, 7	6	1, 2, 5, 7, 8	1, 5, 7, 11, 15, 19
7 C	46.007		7	1, 6, 8, 9	
H	2.633	6, 8, 10, 11	7		6, 8, 11, 24, 28
8 C	135.529		8	6, 9, 11	
H	5.637	7, 9	8	7, 9, 10, 11	2, 7, 29
9 C	134.794		9	5, 8, 11	
H	5.734	8, 10	9	7, 8, 10, 11	3, 10, 12
10 C	46.965		10	5, 8, 9, 12	
H	2.839	5, 7, 9, 11	10		5, 9, 11, 12
11 C	48.947		11	8, 9	
H2	1.237	7, 10	11	5, 6, 8, 9	5, 6, 7, 10
12 C	25.214		12	3, 13', 13''	
H3	1.047		12	2, 3, 4, 5, 10, 13	3, 9, 10, 13'', 15, 19
13 C	50.351		13', 13''	2, 5, 12, 15	
H'	2.714	13''	13	3, 4, 5, 12, 14, 15, 19	1, 15, 19
H''	2.439	13'	13	3, 4, 5, 12, 14, 15, 19	1, 12, 15, 19
14 C	137.649			13', 13'', 16, 18	
15 C	130.892		15	13', 13'', 17	
H	7.121		15	13, 17, 19	1, 3, 6, 12, 13', 13''
16 C	127.766		16	18	
H	7.267		16	14, 18	

Assignments					
Atom	Chemical Shift	COSY	HSQC	HMBC	NOESY
17 C	126.296		17	15, 17	
H	7.233		17	15, 17	
18 C	127.766		18	16	
H	7.267		18	14, 16	
19 C	130.892		19	13', 13'', 15	
H	7.121		19		1, 3, 6, 12, 13', 13''
20 C	166.143			1	
21 C	84.365			29	
22 C					
22F F	-71.030				
22F' F	-71.030				
22F'' F	-71.030				
23 C	132.514				
24 C	127.276		24		
H	7.578		24	26	2, 7, 29
25 C	128.410		25		
H	7.418		25		
26 C	129.572		26	24, 28	
H	7.418		26		
27 C	128.410		27		
H	7.418		27		
28 C	127.276		28		
H	7.578		28	26	2, 7, 29
29 C	55.400		29		
H3	3.598		29	21	1, 2, 8, 24, 28
100 O					
101 O					
102 O					

Diasteromer agrees with GHS-GA-575
for comparison of Mosher ester, please see GHS-GA-615

P-ID: ML00000
Measured on: 04/07/2019
CHIFFRE: GHS-GA-612-01
ELNA #: 3005
Client: Dr. Santanu Ghosh
Group: List
Spectroscopist: SPECTROSCOPIST
Analysed on: 04/07/2019
Analysed by: Leutzsch
Amount: 4 mg
Solvent: CDCl3
Reference: TMS
Temperature: 298 K
Spectrometer: AV500as
Probe: 5 mm PABBO BB-1H/D Z-GRD Z119470/0004
Experiments: 1H-zg30, 13C-zgdc30, [13C, 1H]-ASAP_hsqcedetgpsp, [1H, 1H]-cosygpqf, [1H, 1H]-noesygpph, [13C, 1H]-hmbcgpl2ndqf, 19F-zgfhig30qn.ph

Table 12. Peak table for **17a**: COSY, HMBC, HSQC, NOESY signals for the assignment of stereochemistry.

Procedure for the synthesis of (1*R*,4*S*,4a*R*,5*S*,8*S*,8a*S*)-8-benzyl-8-methyl-1,4,4a,5,8,8a-hexahydro-1,4-methanonaphthalen-5-yl (*S*)-3,3,3-trifluoro-2-methoxy-2-phenylpropanoate 17b: A flame dried Schlenk flask equipped with a teflon coated magnetic stirring bar was cooled

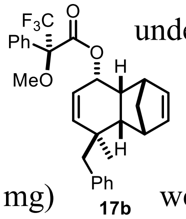

17b

under argon and charged with (*S*)-3,3,3-trifluoro-2-methoxy-2-phenylpropanoyl acid (1.0 equiv., 0.075 mmol, 18 mg) in CH_2Cl_2 (0.2 mL) at 0 °C. To it Et_3N (1.0 equiv., 0.038 mmol, 5 mg) and *N*,*N*'-Dicyclohexylcarbodiimid (3.0 equiv., 0.225 mmol, 46 mg) were added. After 5 minutes a solution of compound **12** (er_{major} = 98:2, dr 13:1) (1.0 equiv., 0.075 mmol, 20 mg) in CH_2Cl_2 (0.2 mL) was added to the solution, and the reaction mixture was stirred for 8 h. The crude mixture was collected and purified by preparative TLC (Alox, 25mm) using 10% EtOAc in hexane and afforded the desired product as colorless oil (18%, dr >20:1); **^{1}H NMR** (501 MHz, $CDCl_3$) δ 7.57–7.55 (m, 2H), 7.46–7.42 (m, 3H), 7.29–7.21 (m, 3H), 7.13–7.11 (m, 2H), 5.72 (dd, *J* = 5.7, 2.8 Hz, 1H), 5.60 (dd, *J* = 5.6, 2.8 Hz, 1H), 5.30–5.26 (m, 2H), 5.09

(ddd, J = 10.3, 3.2, 1.2 Hz, 1H), 3.56 (m, 3H), 3.07 (td, J = 9.6, 3.8 Hz, 1H), 2.85 (br, 1H), 2.75 (br, 1H), 2.71 (d, J = 12.9 Hz, 1H), 2.44 (dd, J = 3.4, 1.3 Hz, 1H), 2.44 (d, J = 13.0 Hz, 1H), 1.26 (br, signal merge with grease impurity, 2H), 1.04 (s, 3H); **^{13}C NMR** (126 MHz, $CDCl_3$) δ 166.4, 137.8, 136.6, 135.6, 135.0, 132.3, 131.1, 129.7, 128.6, 127.9, 127.7, 126.6, 126.4, 124.7, 73.7, 55.4, 50.5, 49.1, 47.1, 46.6, 46.5, 41.4, 37.9, 29.9, 25.4; **^{19}F NMR** (470 MHz, $CDCl_3$) δ −71.16 (s, CF_3); **HRMS** (ESI) calculated for $C_{29}H_{29}F_3O_3Na$ $[M+Na]^+$: 505.196099; found 505.196630.

Suggested structure

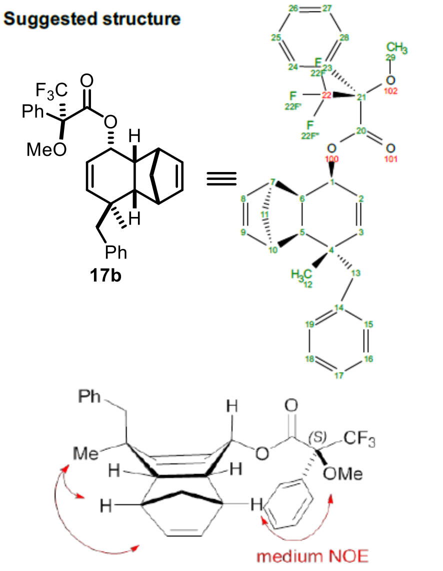

User Report
GHS-GA-615-01

Assignments					
Atom	Chemical Shift	COSY	HSQC	HMBC	NOESY
1 C	73.502		1	6	
H	5.276	6	1		15, 29
2 C	126.424		2	6	
H	5.283		2		8
3 C	136.449		3	5, 12, 13', 13''	
H	5.087	5	3	5	9, 12, 13', 13'', 15
4 C	37.690			5, 12, 13', 13''	
5 C	46.448		5	3, 6, 11, 12, 13', 13''	
H	2.463	3, 6, 10	5	3, 4, 9, 10, 13	6, 10, 11, 12
6 C	41.203		6	11	
H	3.065	1, 5, 7	6	1, 2, 5, 7, 8	5, 7, 11
7 C	46.292		7	6, 8, 9	
H	2.749	6, 8, 10, 11	7		6, 8, 11, 24, 28, 29
8 C	135.426		8	6, 11	
H	5.597	7, 9	8	7, 10, 11	2, 7, 29
9 C	134.875		9	5, 11	
H	5.720	8, 10	9	7, 10, 11	3, 10, 12
10 C	46.947		10	5, 8, 9	
H	2.845	5, 7, 9, 11	10		5, 9, 11, 12
11 C	48.939		11	8, 9	
H2	1.260	7, 10	11	5, 6, 8, 9	5, 6, 7, 10
12 C	25.236		12	13', 13''	
H3	1.039		12	3, 4, 5, 13	3, 5, 9, 10, 13', 13'', 15, 19
13 C	50.346		13', 13''	5, 12, 15, 19	
H'	2.713	13''	13	3, 4, 5, 12, 14, 15, 19	3, 12, 15
H''	2.436	13'	13	3, 4, 5, 12, 14, 15, 19	3, 12, 15
14 C	137.668			13', 13'', 16, 18	

Assignments					
Atom	Chemical Shift	COSY	HSQC	HMBC	NOESY
17 C	126.286		17	15	
H	7.229		17	15, 19	
18 C	127.770		18	16	
H	7.266		18	14, 16	
19 C	130.896		19	13', 13'', 15, 17	
H	7.120		19	13	12
20 C	166.216				
21 C	84.699			29	
22 C					
22F F	-71.197				
22F' F	-71.197				
22F'' F	-71.197				
23 C	132.171			25, 27	
24 C	127.505		24		
H	7.561		24	26	7, 29
25 C	128.448		25		
H	7.436		25	23	
26 C	129.577		26	24	
H	7.422		26		
27 C	128.448		27		
H	7.436		27	23	
28 C	127.505		28		
H	7.561		28		7, 29
29 C	55.264		29		

P-ID: ML00000
Measured on: 12/07/2019
CHIFFRE: GHS-GA-615-01
ELNA #: 3042
Client: Dr. Santanu Ghosh
Group: List
Spectroscopist: Leutzsch
Analysed on: 12/07/2019
Analysed by: Leutzsch
Amount: 4 mg
Solvent: CDCl3
Reference: solvent
Temperature: 298 K
Spectrometer: AV500as
Probe: 5 mm PABBO BB-1H/D Z-GRD Z119470/0004
Experiments: 1H-zg30, [1H, 1H]-cosygpqf, [1H, 1H]-noesygpph, [13C, 1H]-ASAP_hsqcedetgpsp, [13C, 1H]-hmbcgpl2ndqf, 13C-zgdc30, 1H-zg30, 19F-zgfhig30qn.ph

Table 13. Peak table for **17b**: COSY, HSQC, HMBC, NOESY signals for the assignment of stereochemistry.

Confirmation of Absolute Configuration

17b / 16b GHS-GA-615 S-Ester Atom	Chemical Shift	17a / 16a GHS-GA-612 R-Ester Atom	Chemical Shift	δS-δR (ppm)	δS-δR (hz)
1 C	73.502	1 C	73.497		
H	5.276	H	5.314	**-0.038**	**-19.0**
2 C	126.424	2 C	126.296		
H	5.283	H	5.374	**-0.091**	**-45.5**
3 C	136.449	3 C	136.728		
H	5.087	H	5.126	**-0.039**	**-19.5**
4 C	37.69	4 C	37.695		
5 C	46.448	5 C	46.501		
H	2.463	H	2.458	0.005	2.5
6 C	41.203	6 C	41.172		
H	3.065	H	3.08	-0.015	-7.5
7 C	46.292	7 C	46.007		
H	2.749	H	2.633	**0.116**	**58.0**
8 C	135.426	8 C	135.529		
H	5.597	H	5.637	-0.04	-20.0
9 C	134.875	9 C	134.794		
H	5.72	H	5.734	-0.014	-7.0
10 C	46.947	10 C	46.965	-0.018	-9.0
H	2.845	H	2.839	0.006	3.0
11 C	48.939	11 C	48.947		
H2	1.26	H2	1.237	0.023	11.5
12 C	25.236	12 C	25.214		
H3	1.039	H3	1.047	-0.008	-4.0
13 C	50.346	13 C	50.351		
H'	2.713	H'	2.714	-0.001	-0.5
H''	2.436	H''	2.439	-0.003	-1.5

For REF see: T. R. Hoye, C. S. Jeffrey, F. Shao, *Nat. Protoc.* **2007,** 2, 2451–2458.

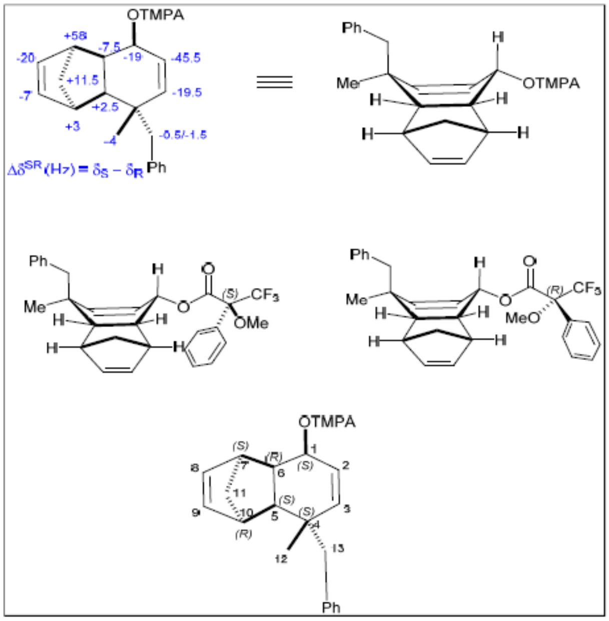

The following absolute configuration explains the observed mosher shifts best:

Due to the concave structure of the molecule (see drawing), H8 and H9 are on the same side as H2 and H3. Therefore, their $\delta\Delta^{SR}$ values are also negative.

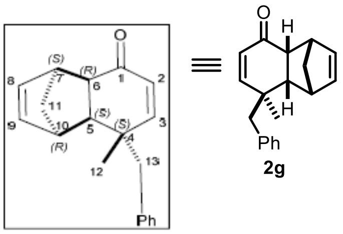

2g

[Note: In addition, the sense of absolute configuration of **9b** derived in two steps from the adduct **2b** (see page **24-25**) is also support the observed absolute stereochemistry of the Diels–Alder adduct]

(*S*)-4-methyl-4-propylcyclohex-2-en-1-one 9b: $[\alpha]_D^{25}$ = –34.7 (*c* 0.075 in MeOH for er = 97:3); Lit.:[2b] $[\alpha]_D^{23}$ = –47.55 ±0.61 (*c* 1.13 mg/ml in MeOH) for er > 99:1]; for **(*R*)-4-methyl-4-propylcyclohex-2-en-1-one 9b** Lit.:[2d] $[\alpha]_D^{13}$ = +4.96 (*c* 2.26 mg/mL in MeOH)]

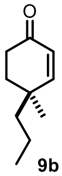

9b

The assignment of relative stereochemistry of product **2l′** was conducted by COSY, HMBC, HSQC, NOESY NMR analysis (see page **19-20**) and comparison of spectroscopic data with literature data.[10] The Diels–Alder adduct was assigned as the *endo*-addition product. The absolute stereochemical configuration of **2l′** was determined by comparing the specific rotation with reported value available in literature. The relative stereochemistry of **2i′** (major diastereomer) was determined by COSY, HMBC, HSQC, NOESY NMR analysis (see page **16-18**) accordingly the absolute stereochemistry of **2i′**, **2j′** and **2k′** were assigned.

methyl (1*S*,4*R*,4a*S*,5*R*,8a*R*)-5-methyl-8-oxo-1,4,4a,5,8,8a-hexahydro-1,4-methanonaphthalene-5-carboxylate 2l′: $[\alpha]_D^{20}$ = −99.5 (*c* 0.46, $CHCl_3$ for er = 92:8). Lit.:[10] $[\alpha]_D^{27}$ = −74.3 (*c* = 2.22, $CHCl_3$, for er = 87:13).

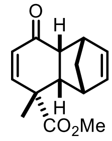

2l'

Computational Studies

All calculations presented in this paper were carried out with a development version of the ORCA suite of programs base on version 4.2.[12] Molecular geometries were optimized in the gas-phase using the PBE functional[13] in conjunction with the D3 version of Grimme's dispersion correction with Becke-Johnson damping function,[14] using the resolution of identity approximation. The def2-SVP basis set was used for all atoms with matching auxiliary basis.[15] Analytic frequency calculations were performed to verify the nature of all stationary points (minima and transition states) and to calculate free energies and enthalpies at 193 K by using the rigid-rotor harmonic oscillator (RRHO) approximation, as implemented in ORCA. Single-point energies considering solvation effects (toluene) were also carried out via the conductor like polarizable continuum model (C-PCM)[16] at M06-2X/def2-TZVP level.[17] Accurate enantio- and diastereoselectivites were computed by computing single-point gas phase energies at the DLPNO-CCSD(T)/def2-TZVP[18] level. NormalPNO settings and tighten TCutPairs threshold (10^{-5}) were used. Solvation, entropy and thermal corrections were kept at DFT level. This protocol is denoted as (DLPNO-CCSD(T) + C-PCM(toluene))//PBE-D3/def2-SVP and was already successfully used for studying closely related systems.[19]

To further assess the performance of the computational protocol used in the geometry optimizations (PBE-D3/def2-SVP) for the systems studied in this work, we computed the

geometry of the stereodetermining **TS-*ent*-2a** using various computational protocols: (i) TPSSh[20]-D3/def2-SVP; (ii) PBE-D3/def2-TZVP(-f); (iii) (C-PCM-PBE-D3/def2-SVP). All computational protocols led to very similar geometries at can be seen in Table 11 (root-mean-square-deviation, RMSD, smaller than 0.16Å).

Conformational sampling: The initial conformation sampling was done using the XTB code (version 6.1).[21] The TSs conformer/rotamer ensembles were generated through the default MTD-GC procedure at the semi-empirical tight-binding based quantum chemistry method GFN2-xTB[22] implemented in CREST 2.6.[23] This procedure executes extensive RMSD based meta-dynamic searches between 400-500 K with a length that is unique to each molecule (based on the molecular flexibility). At the end an extra genetic crossing step is done. Default settings were used (canonical NVT ensemble with a Berendsen thermostat[24] at a heat transfer time of 0.5 ps. Covalent bonds are constrained with the SHAKE algorithm for MD time step dτ of 5 fs). Keeping the forming C···C bond distances fixed, a total of 442 **(TS-2a)**, 343 (**TS-2a′**), 985 (**TS-2j′**), and 540 (**TS-2j**) conformers were found. The structures were further refined at PBE-D3/def2-SVP level, leading to a total of 177 low-energy TS conformers.

Table 14. Comparison between the optimized **TS-*ent*-2a** geometries computed at the reference level PBE-D3/def2-SVP *vs* the other computational protocols.[a]

**Method**	TPSSh-D3/def2-SVP	PBE-D3/def2-TZVP(-f)	(C-PCM)-PBE-D3/ def2-SVP
	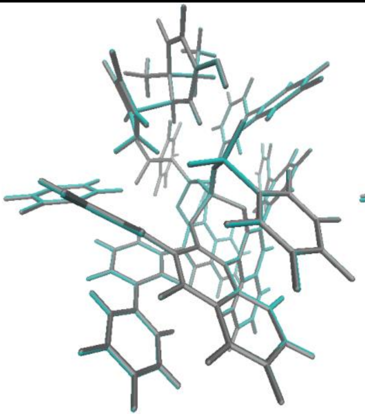	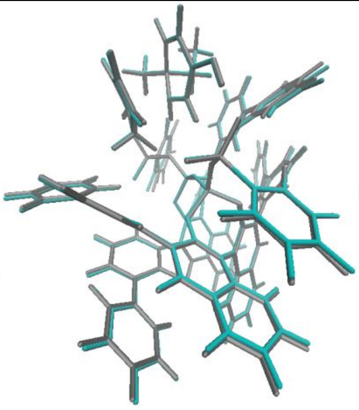	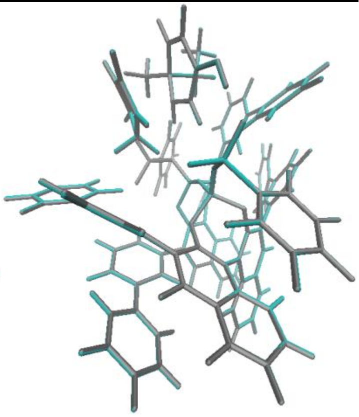
**RMSD (Å)**	0.146	0.159	0.035

[a]The superimposed structures of the reference (gray) and the test (cyan).

Molecular Electrostatic Potentials (MEPs): MEPs were computed separately for the cation (protonated dienone) and the anion at the geometry they have in the TS. An isodensity surface of 0.002 au was used to map the MEPs in a range −0.1 (red) to +0.1 (blue). It can be seen that the counteranion at the TS exhibits an open pocket were the negative charge (red region of MEPs in **Figure 1**) is highly delocalized between the nitrogen atoms and the oxygens of the $–SO_2C_6F_5$ groups. The regions of the activated dienone expected to be attracted favorably (dark blue region of MEPs in **Figure 1**) by the negative regions of the counteranion are the proton and the four unsaturated carbons able to form the new σ-bonds with cyclopentadiene.

Local Energy Decomposition (LED) Analysis: LED permits to quantify the most important chemical terms stabilizing the stereodetermining TSs. This allows to unravel the factors determining the selectivity. The theory of LED scheme has been described in recent publications,[25] and hence, we only describe herein the main features of this analysis. The DLPNO-CCSD(T) binding energy between the CIP and the CP can be partitioned as

$$\Delta E = \Delta E_{\text{geo-prep}} + \Delta E_{\text{int}} \tag{1}$$

where $\Delta E_{\text{geo-prep}}$ is the geometric preparation energy required to deform the fragments from their equilibrium structure into the geometry they have in the TS; while ΔE_{int} is the interaction energy between the distorted fragments.

Under the DLPNO-CCSD(T) framework, the occupied HF orbitals of the TS are localized into the fragment where they mostly belong. By exploiting this localization, the HF contribution to the interaction energy can be decomposed as:

$$\Delta E_{\text{int}}^{\text{HF}} = \Delta E_{\text{el-prep}}^{\text{HF}} + E_{\text{elstat}} + E_{\text{exch}} \tag{2}$$

E_{elstat} and E_{exch} are the permanent and induced electrostatic and exchange interaction between the electron density of the deformed fragments. E_{exch} is always attractive while the sign of E_{elstat} depends on the system. The electronic preparation $\Delta E_{\text{el-prep}}^{\text{HF}}$ is always repulsive and represents the energy required to distort the electronic structure of the fragments from their unperturbed state to the one they have in the interacting system. Thus, it comprises the so-called “Pauli repulsion” as well as polarization effects.

Finally, the correlation contribution to the interaction energy term can be expressed as the sum of the non-dispersive ($\Delta E_{\text{no-disp}}$) and dispersive (E_{disp}) correlation contributions, which includes the London dispersion. Thus, the overall binding energy between the CIP and the CP is given as:

$$\Delta E = \Delta E_{\text{geo-prep}} + \Delta E^{\text{HF}}_{\text{el-prep}} + E_{\text{elstat}} + E_{\text{exch}} + E_{\text{disp}} + \Delta E_{\text{no-disp}} \quad (3)$$

Reaction mechanism of dienone 1j with cyclopentadiene catalyzed by (*S*,*S*)-6d: To provide an insight into the asymmetric cycloaddition mechanism we have computed the reaction profile for methoxy dienone (**1j**), cyclopentadiene (CP) and catalyst **(*S*,*S*)-6d** at the M06-2X/def2-TZVP + C-PCM-(toluene)//PBE-D3/def2-SVP level of theory (see **Figure 1**). The catalyst activation is an exergonic process (ΔG = −8.7 kcal·mol^{-1}) that occurs via the protonation of **1j**. The resulting protonated ketone forms a strongly directional H-bond with an oxygen atom in the $-SO_2C_6F_5$ group of the anion (**CIP-1j** in **Figure 1**). Successively, **CIP-1j** forms a reactant complex (**RC-1j**) with CP, which afterwards forms product complex **P-2j′** by an *endo* attack of the substrate. In the stereodetermining TS structure **TS-2j′**, the cyclopentadiene interacts with the catalyst through a network of non-conventional C−H···O and C−H···N hydrogen bonds. Finally, after the stereodetermining step, product **2j′** is released upon exchange with **1j**, thus forming **CIP-1j** and concomitantly restoring the catalytic cycle. It is noteworthy that the H-bond established as the early molecular recognition between the activated substrate and the counteranion in **CIP-1j** is conserved along the whole reaction mechanism.

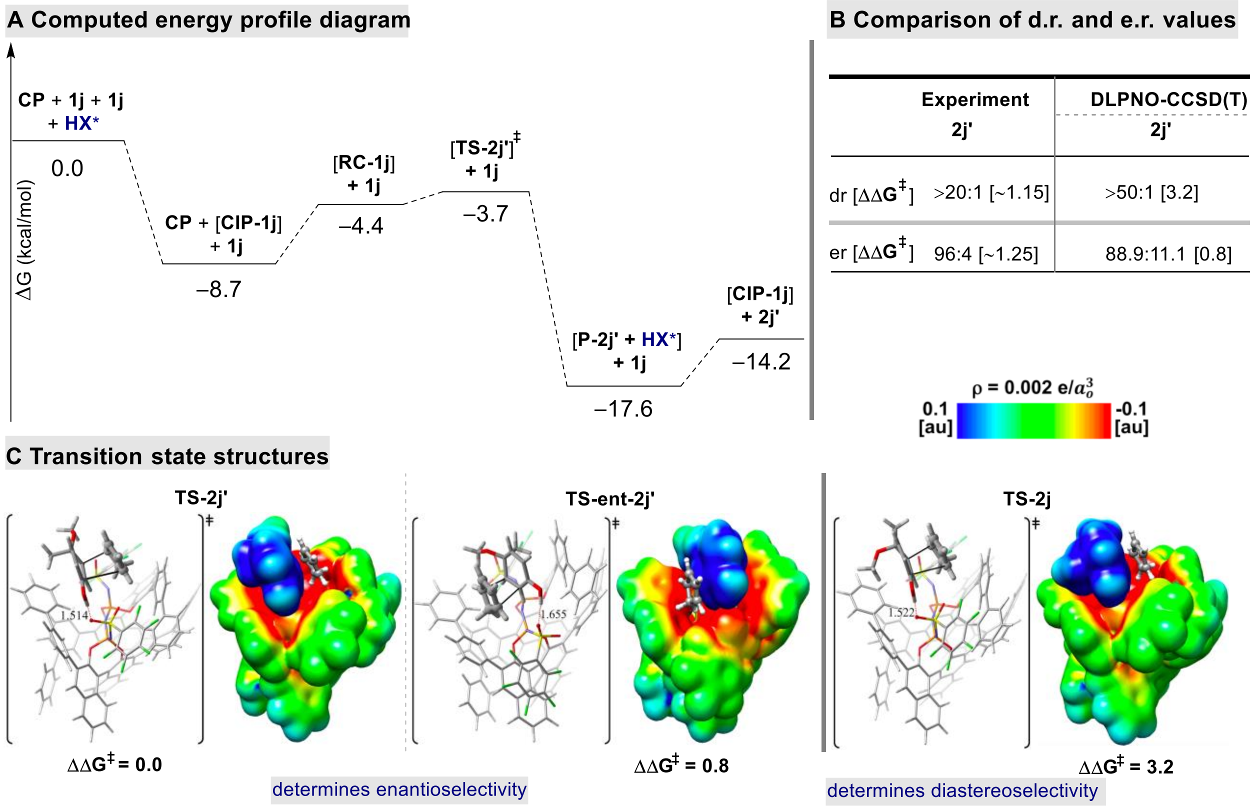

Figure 1. Computational Studies. (**A**) Proposed reaction mechanism calculated at M06-2X/def2-TZVP+C-PCM-(toluene)//PBE-D3/def2-SVP level. (**B**) Experimental and computational dr and er values together with $\Delta\Delta G^{\ddagger}$ at −80 ºC (in kcal·mol^{-1}). TS single-point energies at DLPNO-CCSD(T)/def2-TZVP+C-PCM(toluene)//PBE-D3/def2-SVP level. (**C**) Enantiomeric and diastereomeric TS structures with corresponding MEPs.

Accurate dr and er values (**Figure 1**) were calculated as the relative free energies of the TSs (according to the Curtin-Hammett principle)[26] at DLPNO-CCSD(T)/def2-TZVP+ C-PCM-(toluene)//PBE-D3/def2-SVP level. For the major diastereomer and enantiomer **2j′**, the experimentally determined dr is 20:1 ($\Delta\Delta G^{\neq}$ = 1.15 kcal·mol^{-1}) and er is 96:4 ($\Delta\Delta G^{\neq}$ = 1.25 kcal·mol^{-1}) which are in good agreement with the computed dr and er values of 50:1 ($\Delta\Delta G^{\neq}$ = 3.2 kcal·mol^{-1}) and 88.9:11.1 ($\Delta\Delta G^{\neq}$ = 0.8 kcal·mol^{-1}), respectively.

Origin of the Stereoselectivity: To shed light into the reverse and notably different diastereoselectivity of **2a** (dr 5:1) and **2j′** (dr > 20:1), we used the LED analysis of the DLPNO-CCSD(T) interaction energy between the CIP and CP at the TS geometries (see **Table 14**).

Calculated energies of **TS-2a** and **TS-2a′** (ΔΔE = 0.4 kcal·mol^{-1}) indicate that CP favorably attacks the less congested face of the substrate to form **2a**, which corresponds to that with the small group closer to the π-system of CP (see **Figure 2B**). In contrast, the exclusive formation of **2j′** is evident from the stability of the **TS-2j′** over **TS-2j** (ΔΔE = 6.1 kcal·mol^{-1}). This preference arises from the lower steric repulsion between the π-system of CP and the methoxy oxygen ($\Delta E^{\mathrm{HF}}_{\mathrm{el\text{-}prep}}$ (**TS-2j′**) = 362.7 kcal·mol^{-1}) compared to that induced by the methylene group ($\Delta E^{\mathrm{HF}}_{\mathrm{el\text{-}prep}}$ (**TS-2j**) = 369.7 kcal·mol^{-1}). This can be seen qualitatively from the non-covalent (NCI) index[16] depicted in **Figure 2B**. All TSs are highly asymmetric and show an attractive interaction between the two carbon atoms that will form the first σ-bond (bluish circle, **Figure 2**). A steric interaction between both rings (extended green surfaces) is also present. Note that for **TS-2j** this surface extends beyond the parallel aligned rings evidencing the aforementioned steric repulsion between the π-system of CP and the methylene group (see red arrow in **TS-2j**).

Table 15. DLPNO-CCSD(T)/LED results (in kcal·mol^{-1}) for the interaction of dienone **1a** and **1j** with cyclopentadiene in the enantiodetermining TSs for the corresponding Diels-Alder reactions. The difference between both TSs for each term is also reported.

TS	2a	2a′	(2a′ – 2a)	2j′	2j	(2j – 2j′)
ΔE	-3.1	-2.7	0.4	-11.2	-5.1	6.1
$\Delta E_{\mathrm{geo\text{-}prep}}$	21.8	22.3	0.5	10.0	15.6	5.6
ΔE_{int}	-24.9	-25.0	-0.1	-21.2	-20.7	0.5
$\Delta E^{\mathrm{HF}}_{\mathrm{el\text{-}prep}}$	572.5	560.7	-11.8	362.7	369.7	7.0
E_{elstat}	-474.4	-463.5	10.9	-286.4	-289.6	-3.2
E_{exch}	-105.2	-104.0	1.2	-78.7	-81.4	-2.7
E_{disp}	-22.9	-22.8	0.1	-19.6	-20.4	-0.8
$\Delta E_{\mathrm{no\text{-}disp}}$	5.0	4.5	-0.5	0.7	1.1	0.4

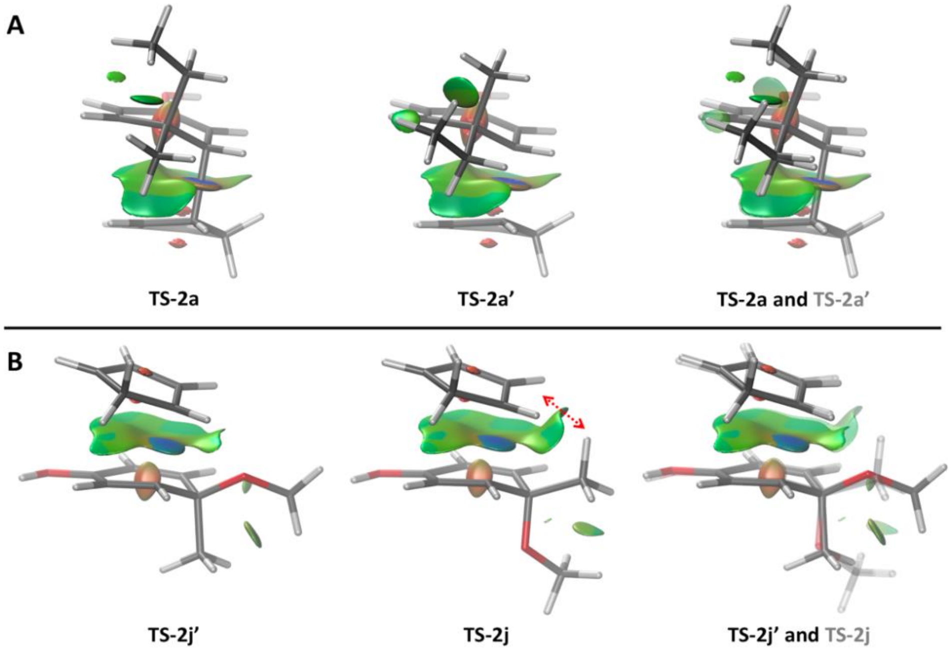

Figure 2. Contour plots of the reduced density gradient isosurfaces (cutoff of 0.07 a.u.) for (**A**) **TS-2a**, **TS-2a′** and superimposed TS structures, and for (**B**) **TS-2j′**, **TS-2j** and superimposed TS structures. The surface color code is blue for strong attractive, green for weak interactions, and red for strong repulsive interactions. Catalyst structure was removed for the sake of clarity.

References and Notes:

[1] R. B. Moffett, *Org. Synth*. **1952**, *32*, 41.

[2] a) A. Bokka, J. X. Mao, J. Hartung, S. R. Martinez, J. A. Simanis, K. Nam, J. Jeon, X. Shen, *Org. Lett.* **2018**, *20*, 5158–5162; b) B. M. Trost, R. N. Bream, J. Xu, *Angew. Chem. Int. Ed.* **2006**, *45*, 3109–3112; c) L. C. da Silva Filho, V. Lacerda Júnior, M. G. Constantino, G. V. J. da Silva, P. R. Invernize, *Beilstein J. Org. Chem.* **2005,** *1*, No. 14. doi:10.1186/1860-5397-1-14 d) G. Otani, S-I. Yamada, *Chem. Pharm. Bull.***1973**, *21*, 2125–2129; e) S. E. Denmark, K. L. Habermas, G. A. Hite, *Helv. Chim. Acta.* **1988,** *71*, 168–194; f) C. Sabot, B. Commare, M-A. Duceppe, S. Nahi, K. C. Guérard, S. Canesi, *Synlett,* **2008**, *20*, 3226–3230.

[3] Y. Han, S. Breitler, S. Zheng, E. J. Corey, *Org. Lett.* **2016**, *18*, 6172–6175.

[4] a) K. B. Chai, P. Sampson, *J. Org. Chem.* **1993,** *58*, 6807–6813; b) H. Hopf, J. Kämpen, P. Bubenitschek, P. G. Jones, *Eur. J. Org. Chem.* **2002**, 1708–1721.

[5] K. Ohkata, Y. Tamura, B. B. Shetuni, R. Takagi, W. Miyanaga, S. Kojima, L. Paquette, *J. Am. Chem. Soc.* **2004,** *126*, 16783–16792.

[6] a) T. Akiyama, H. Morita, J. Itoh, K. Fuchibe, *Org. Lett.* **2005**, *7*, 2583–2585; b) M. Terada, *Synthesis.* **2010**, *2010*, 1929–1982.

[7] a) P. García-García, F. Lay, P. García-García, C. Rabalakos, B. List, *Angew. Chem. Int. Ed.* **2009**, *48*, 4363–4366; b) S. Prevost, N. Dupre´, M. Leutzsch, Q. Wang, V. Wakchaure, B. List, *Angew. Chem. Int. Ed.* **2014**, *53*, 8770–8773.

[8] J. H. Kim, I. Čorić, S. Vellalath, B. List, *Angew. Chem. Int. Ed.* **2013**, *52*, 4474–4477.
[9] a) P.S.J. Kaib, L. Schreyer, S. Lee, R. Properzi, B. List, *Angew. Chem. Int. Ed.* **2016**, *55*, 13200–13203; b) S. Lee, P. S. J. Kaib, B. List, *J. Am. Chem. Soc.* **2017**, *139*, 2156–2159; c) S. Lee, H. Y. Bae, B. List, *Angew.Chem. Int. Ed.* **2018**, *57*, 12162–12166.
[10] R. Takagi, T. Nishi, *Org. Biomol. Chem.* **2015**, *13*, 11039–11045.
[11] Product **2e** was synthesized by following a reverse addition strategy. First cyclopentadiene was added and cooled to –95 °C. After 10 minutes at this temperature, starting material (was added through the wall of the reaction vial and stirred for the specific reaction time
[12] F. Neese, Wiley Interdiscip. Rev.: *Comput. Mol. Sci.* **2011**, *2*, 73–78.
[13] Y. Zhang, W. Yang, *Phys. Rev. Lett.* **1998**, *80*, 890–890.
[14] a) S. Grimme, J. Antony, S. Ehrlich, H. Krieg, *J. Chem. Phys.* **2010**, *132*, 154104; b) S. Grimme, S. Ehrlich, L. Goerigk, *J. Comput. Chem.* **2011**, *32*, 1456–1465.
[15] a) A. Hellweg, C. Hättig, S. Höfener, W. Klopper, *Theor. Chem. Acc.* **2007**, *117*, 587–597; b) F. Weigend, *Phys. Chem. Chem. Phys.* **2006**, *8*, 1057–1065; c) F. Weigend, *J. Comput. Chem.* **2007**, *29*, 167–175; d) F. Weigend, R. Ahlrichs, *Chem. Phys.* **2005**, *7*, 3297–3305.
[16] V. Barone, M. Cossi, *J. Phys. Chem. A* **1998**, 102, 1995–2001.
[17] Y. Zhao, D. G. Truhlar, *Theor. Chem. Acc.* **2008**, *120*, 215–241.
[18] C. Riplinger, P. Pinski, U. Becker, E. F. Valeev, F. Neese, *J. Chem. Phys.* **2016**, *144*, 024109.
[19] T. Gatzenmeier, M. Turberg, D. Yepes, Y. Xie, F. Neese, G. Bistoni, B. List, *J. Am. Chem. Soc.* **2018**, *140*, 12671–12676.
[20] a) J. M. Tao, J. P. Perdew, V. N. Staroverov, G. E. Scuseria, *Phys. Rev. Lett.* **2003**,*91*, 146401; b) V. N. Staroverov, G. E. Scuseria, J. M. Tao, J. P. Perdew, *J. Chem. Phys.* **2003**, *119*, 12129–12137.
[21] xtb v6.1. Ccontact xtb@thch.uni-bonn.de for access the program, University Bonn, **2019**.
[22] C. Bannwarth, S. Ehlert, S. Grimme, *J. Chem. Theory. Comput.* **2019**, *15*, 1652–1671.
[23] S. Grimme, C. Bannwarth, S. Dohm, A. Hansen, J. Pisarek, P. Pracht, J. Seibert, F. Neese, *Angew. Chem. Int. Ed.* **2017**, *56*, 14763–14769.
[24] H. C. J. Berendsen, *Simulating the Physical World: Hierachical Modeling from Quantum Mechanics to Fluid Dynamics*; Cambridge University Press: Cambridge, **2007**.
[25] a) W. B. Schneider, G. Bistoni, M. Sparta, M. Saitow, C. Riplinger, A. A. Auer, F. Neese, *J. Chem. Theory. Comput.* **2016**, *12*, 4778–4792; b) G. Bistoni, A. A. Auer, F. Neese, *Chem.: Eur. J.* **2017**, *23*, 865–873; c) A. Altun, F. Neese, G. Bistoni, *Beilstein J. Org. Chem.* **2018**, *14*, 919–929; d) Q. Lu, F. Neese, G. Bistoni, *Angew. Chem. Int. Ed.* **2018**, *57*, 4760–4764; e) A. Altun, F. Neese, G. Bistoni, *J. Chem. Theory. Comput.* **2019**, *15*, 215–228.
[26] P. I. Pollak, D. Y. Curtin, *J. Am. Chem. Soc.* **1950**, *72*, 961–965.
[27] E. R. Johnson, S. Keinan, P. Mori-Sanchez, J. Contreras-Garcia, A. J. Cohen, W. T. Yang, *J. Am. Chem. Soc.* **2010**, *132*, 6498–6506.

NMR traces

^{1}H NMR, ^{13}C NMR, ^{19}F and ^{31}P Spectra 6c

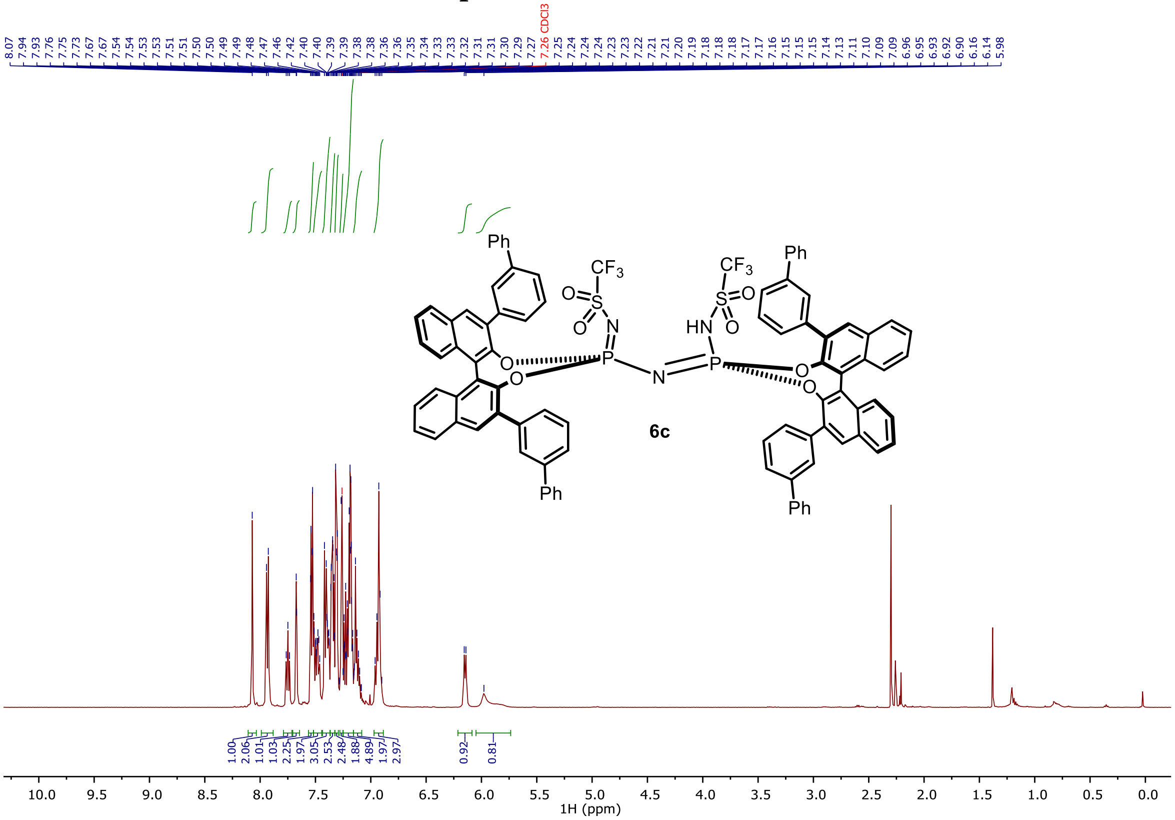

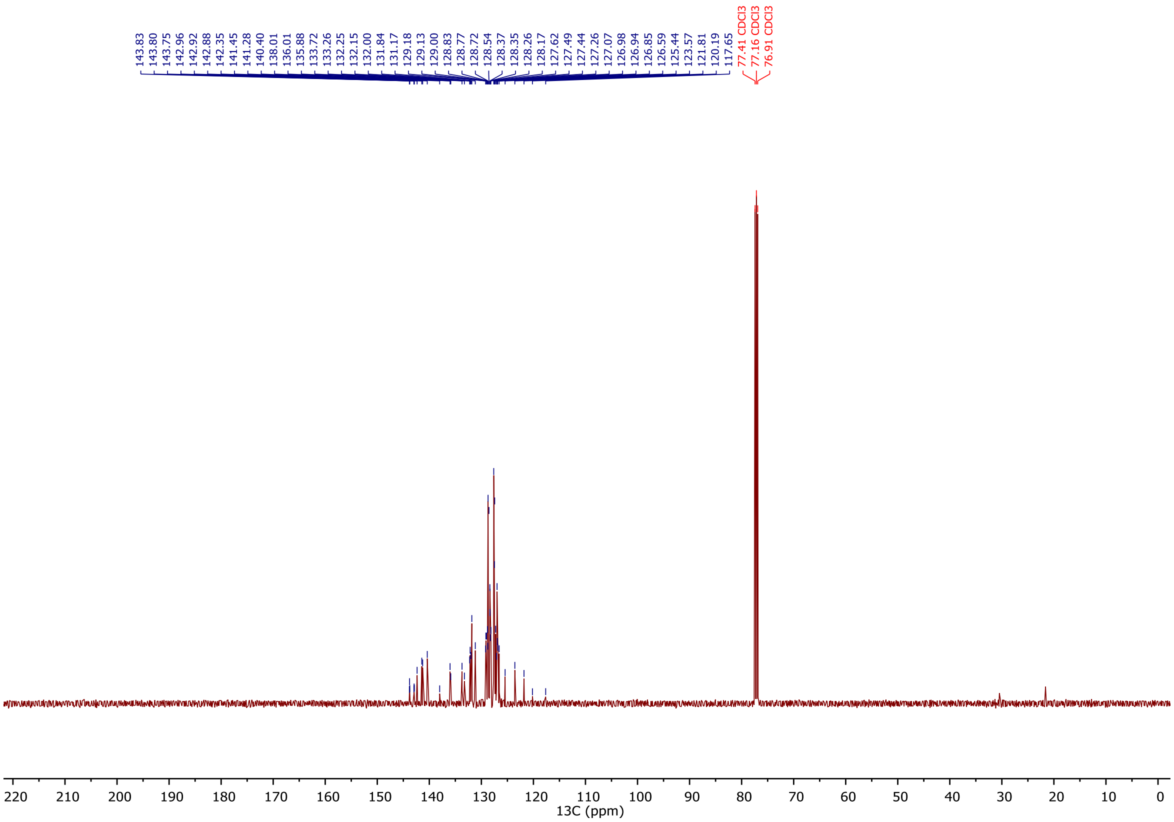

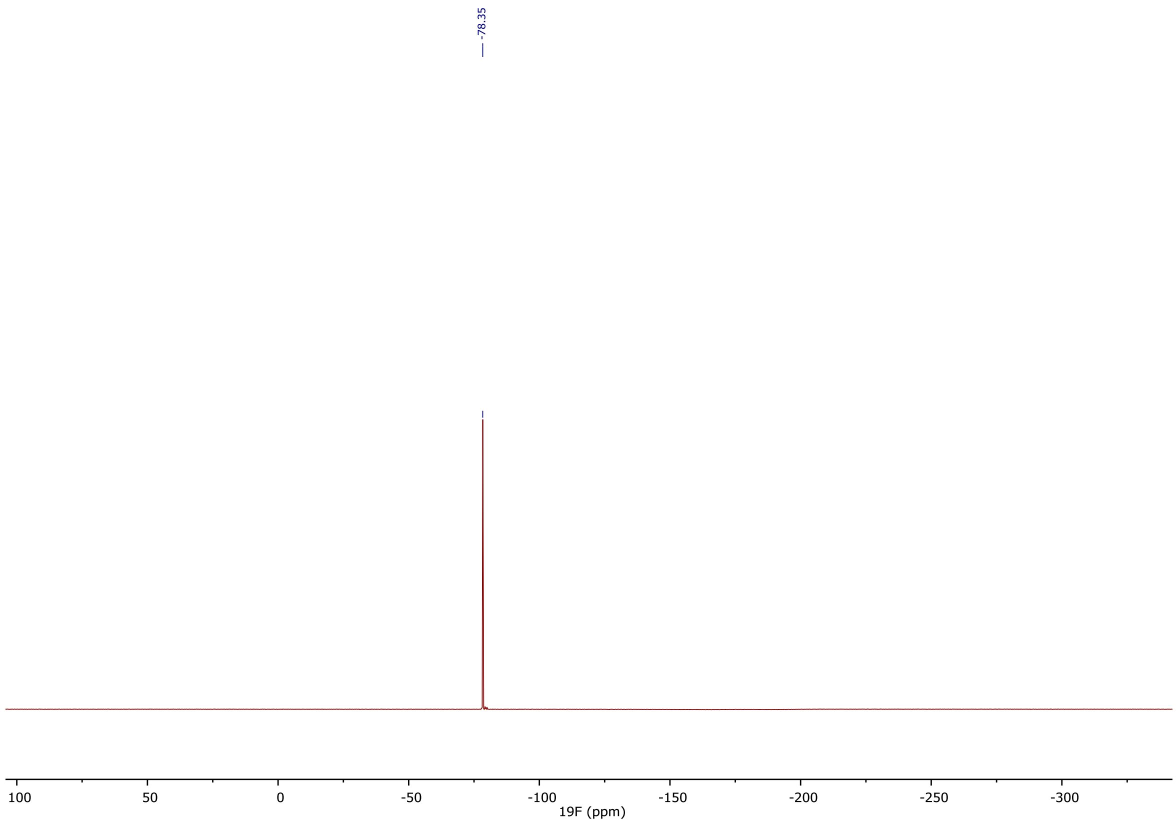

-78.35
100
50
0
-50
-100
-150
-200
-250
-300
19F (ppm)

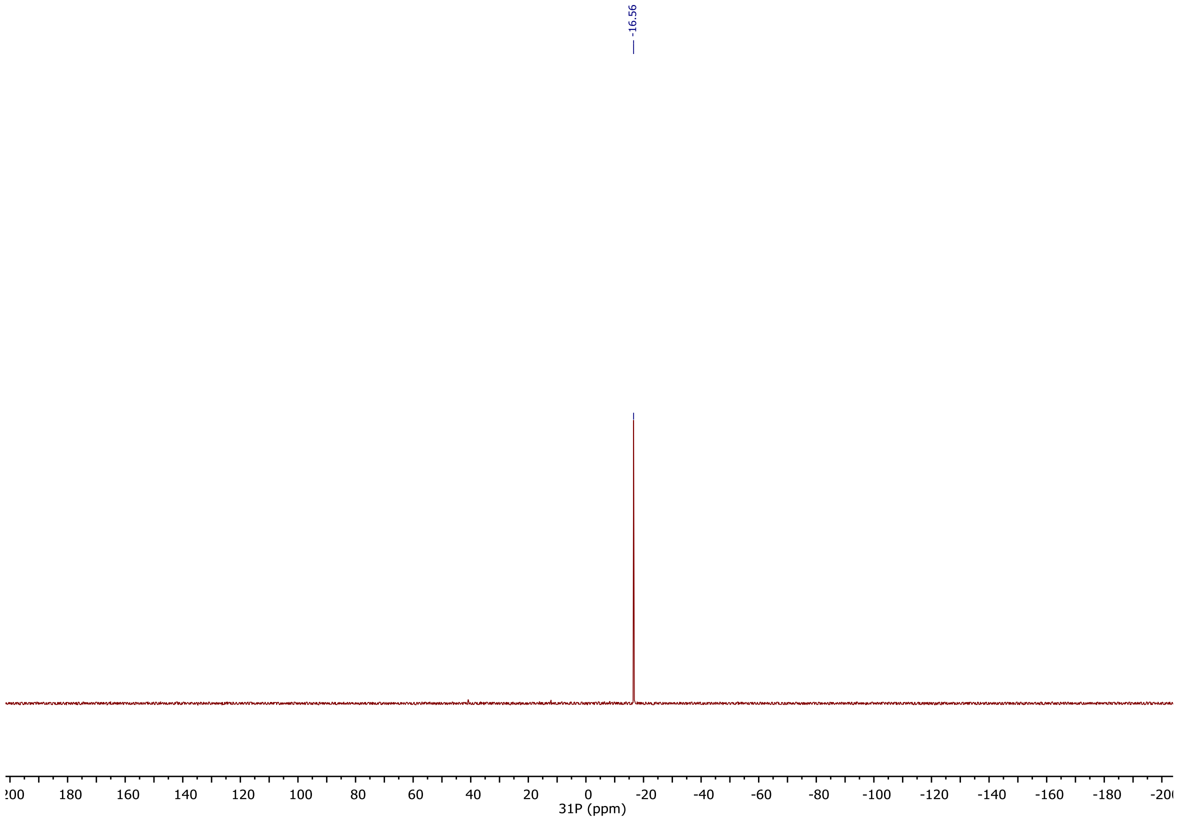

-16.56
180
160
140
120
100
80
60
40
20
0
-20
-40
-60
-80
-100
-120
-140
-160
-180
31P (ppm)

^{1}H NMR, ^{13}C NMR, ^{19}F and [31]P Spectra 6d

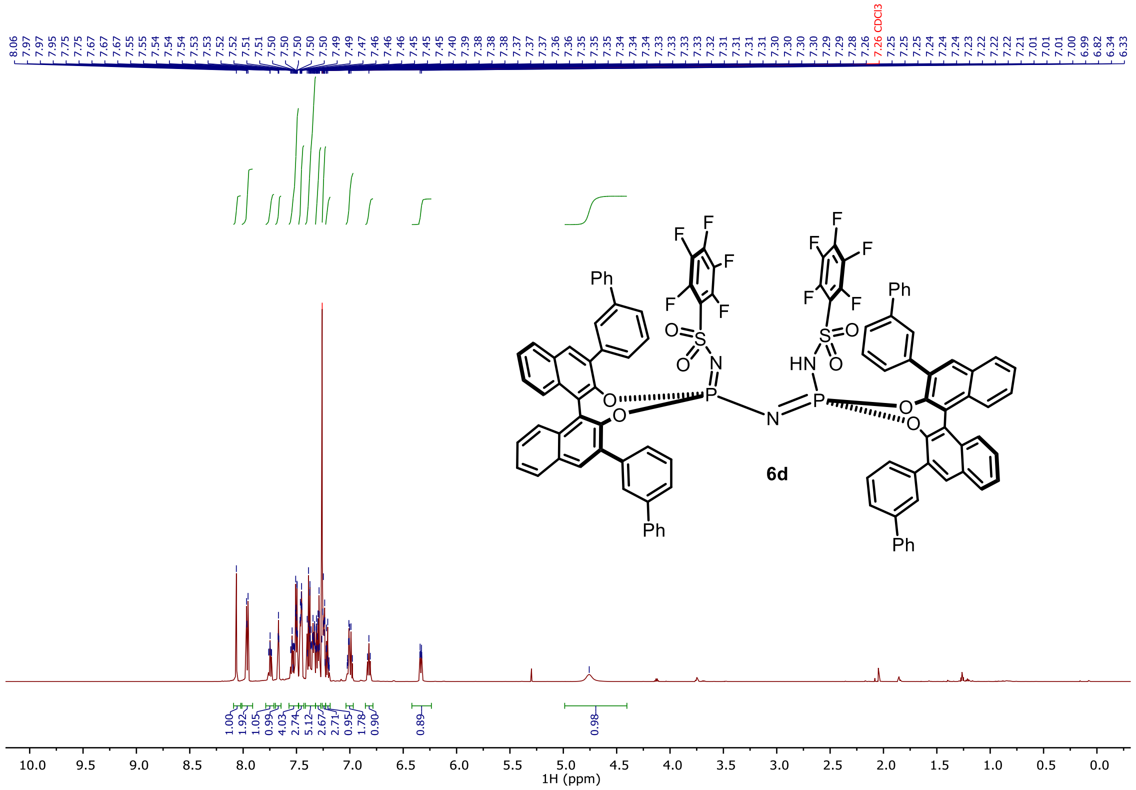

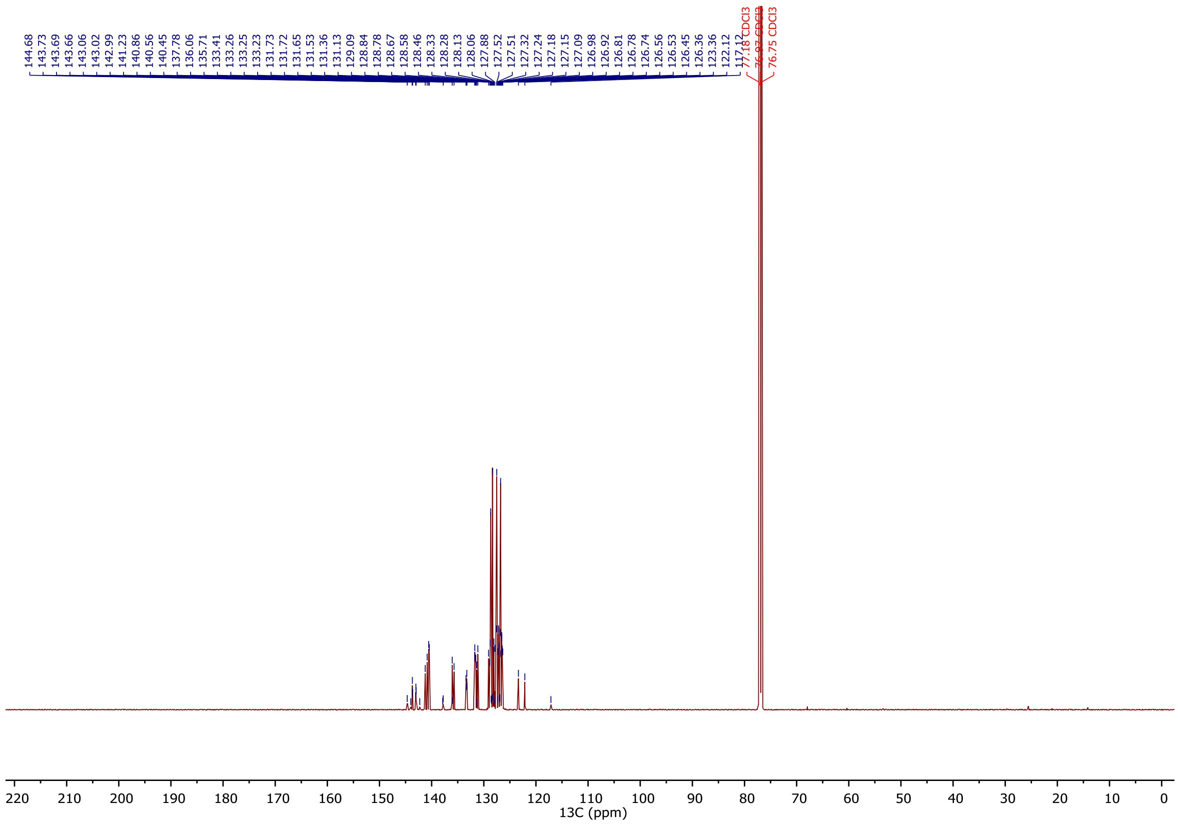

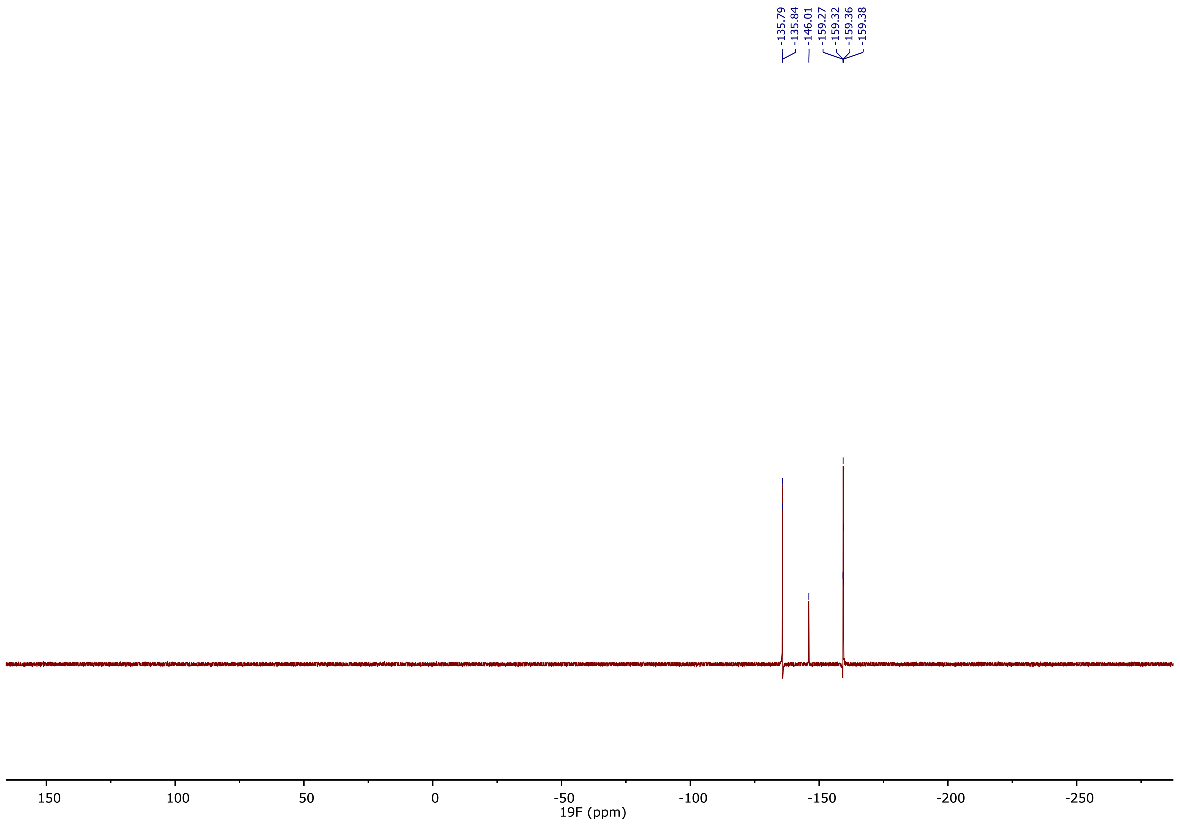
-135.79
-135.84
-146.01
-159.27
-159.32
-159.36
-159.38
150
100
50
0
-50
-100
-150
-200
-250
19F (ppm)

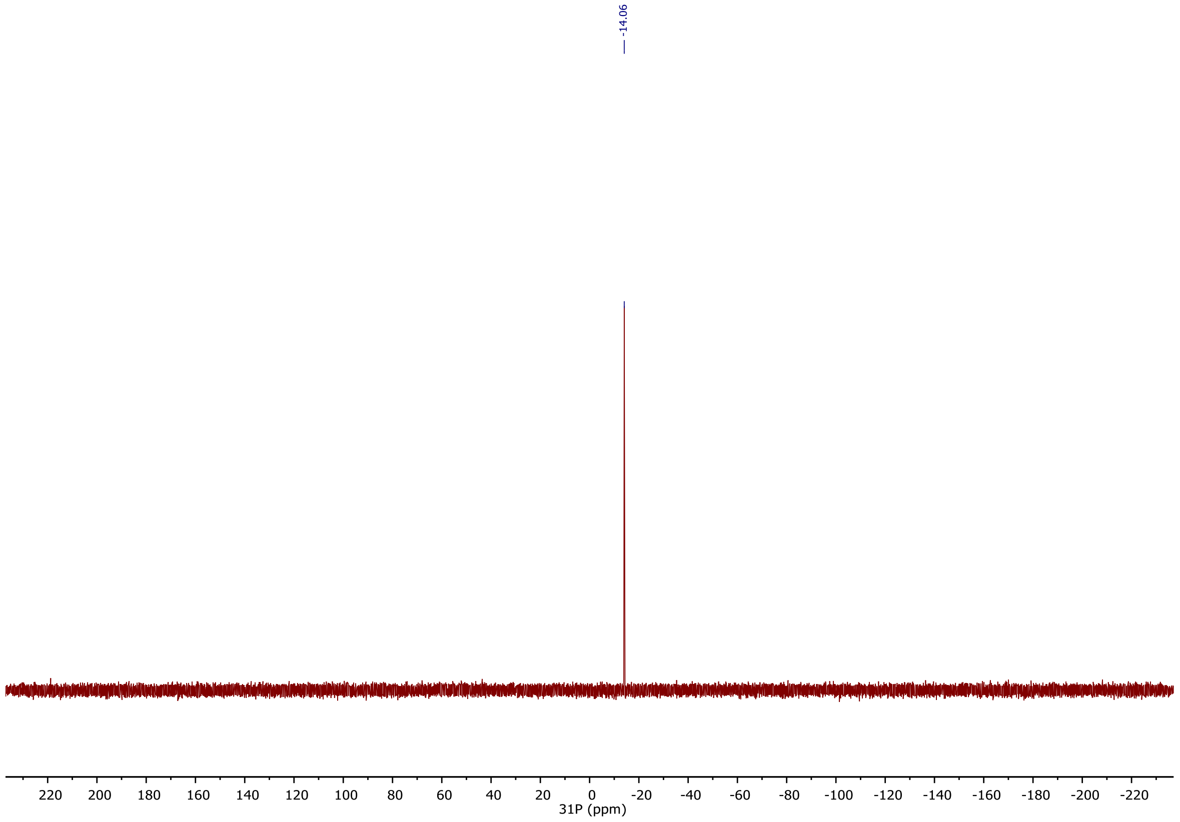
-14.06
220
200
180
160
140
120
100
80
60
40
20
0
-20
-40
-60
-80
-100
-120
-140
-160
-180
-200
-220
31P (ppm)

^{1}H NMR and ^{13}C NMR Spectra (±)-9g

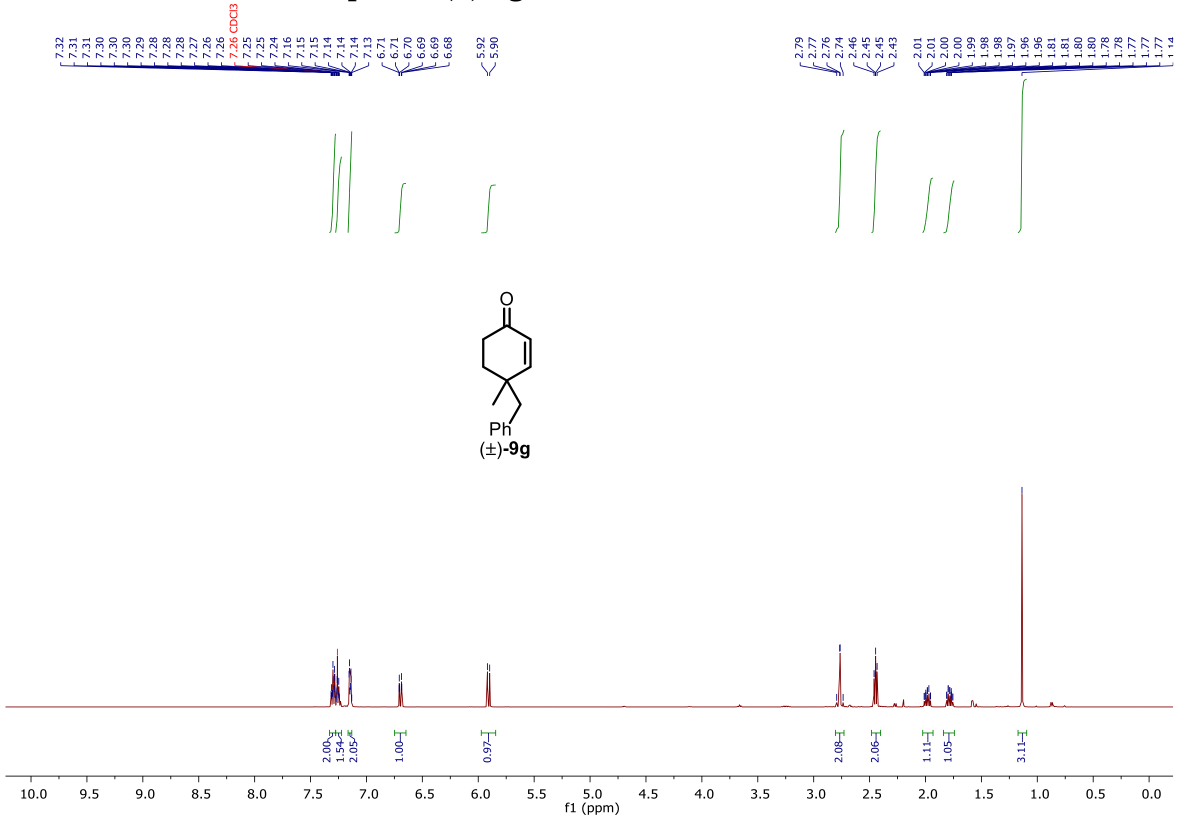

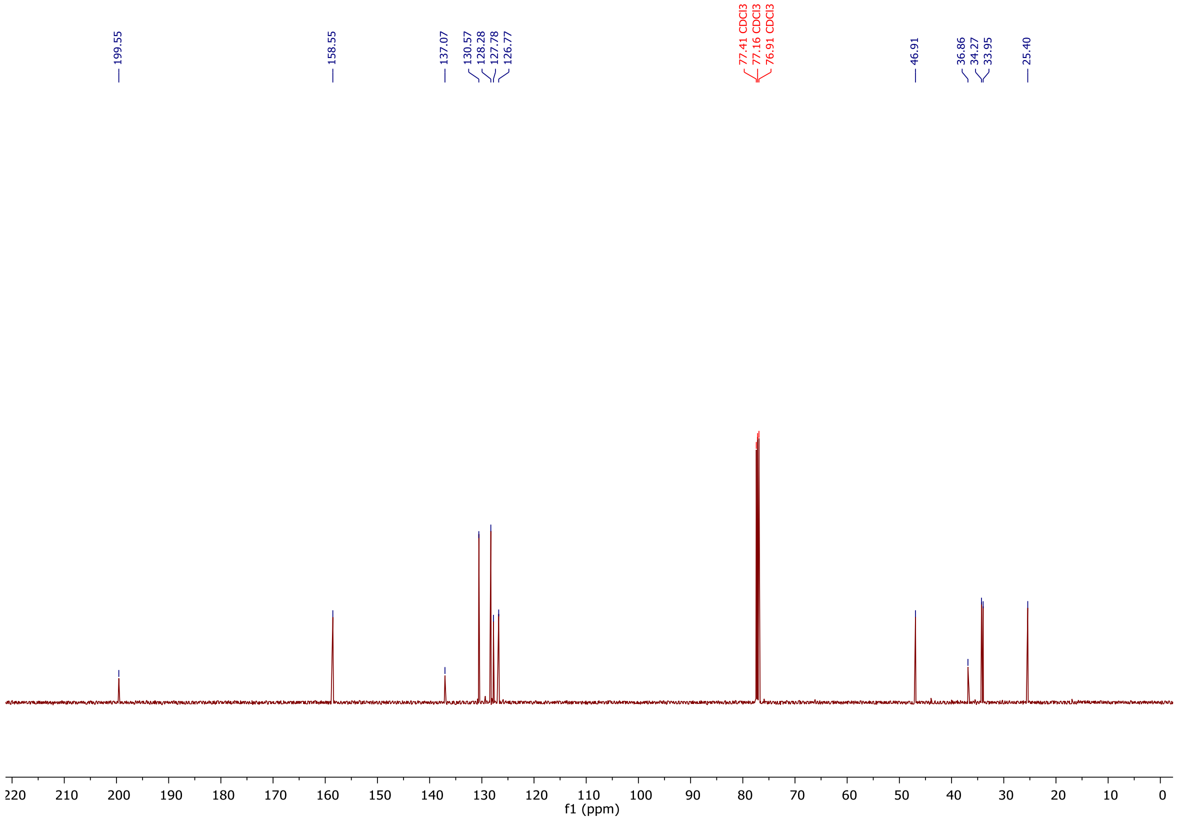

^{1}H NMR and ^{13}C NMR Spectra (±)-9h

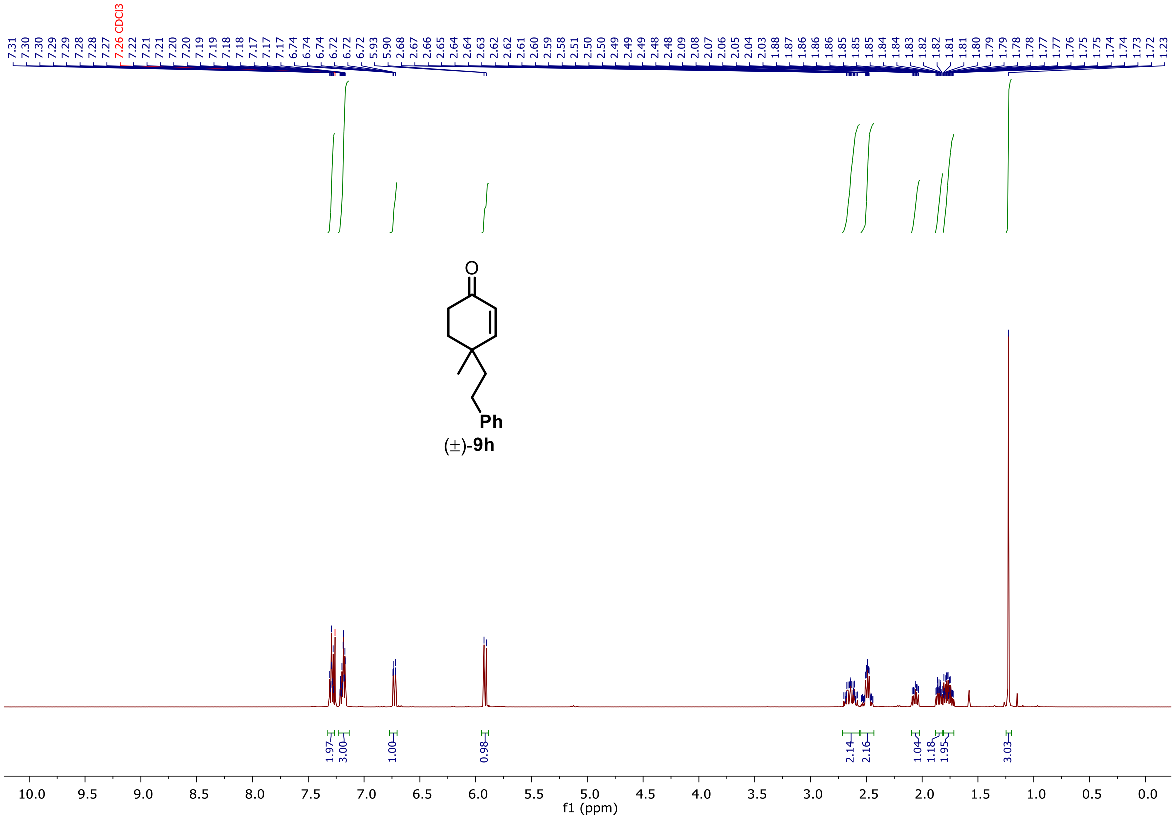

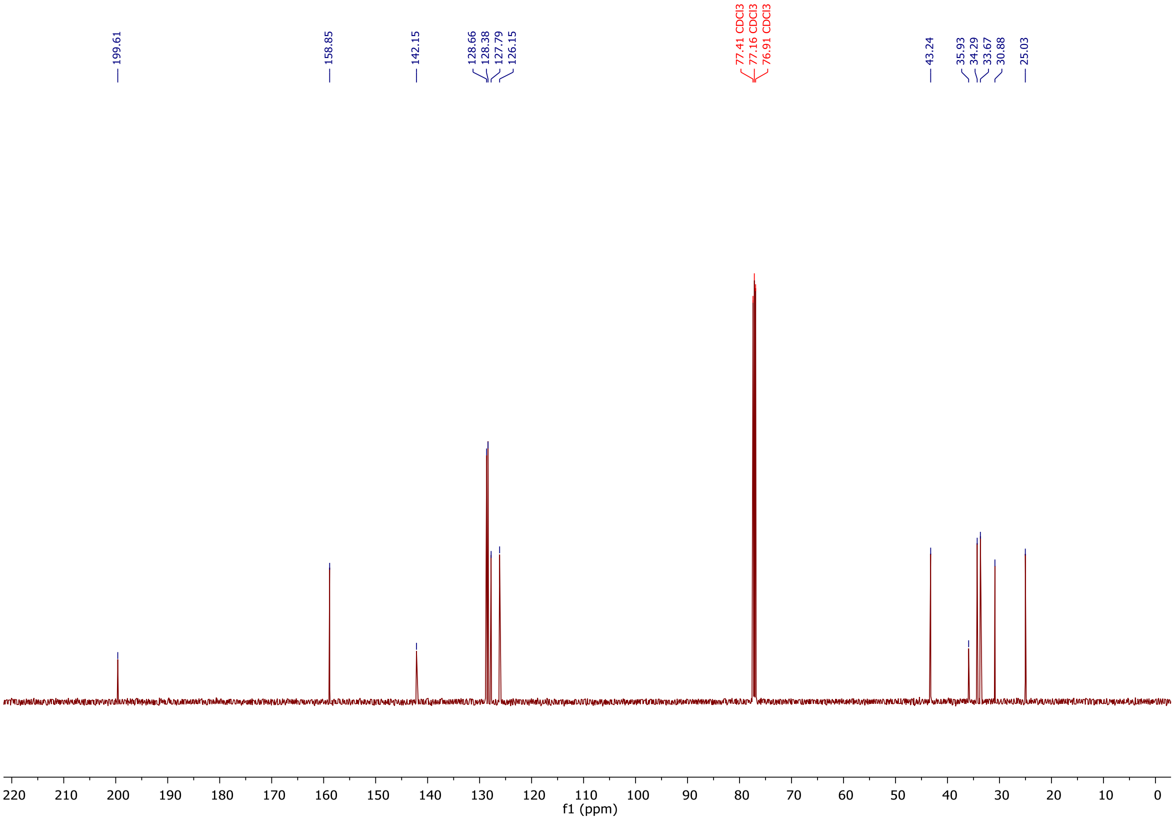

^{1}H NMR and ^{13}C NMR Spectra 1b

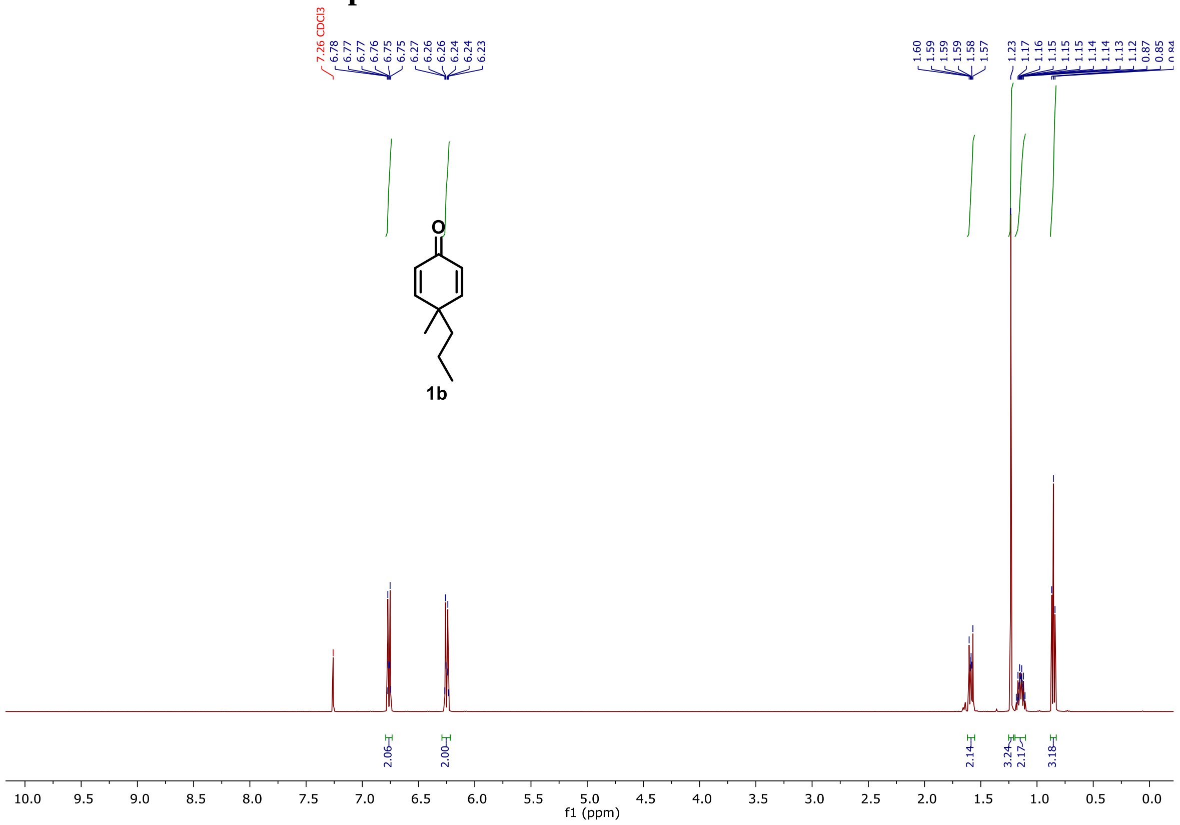

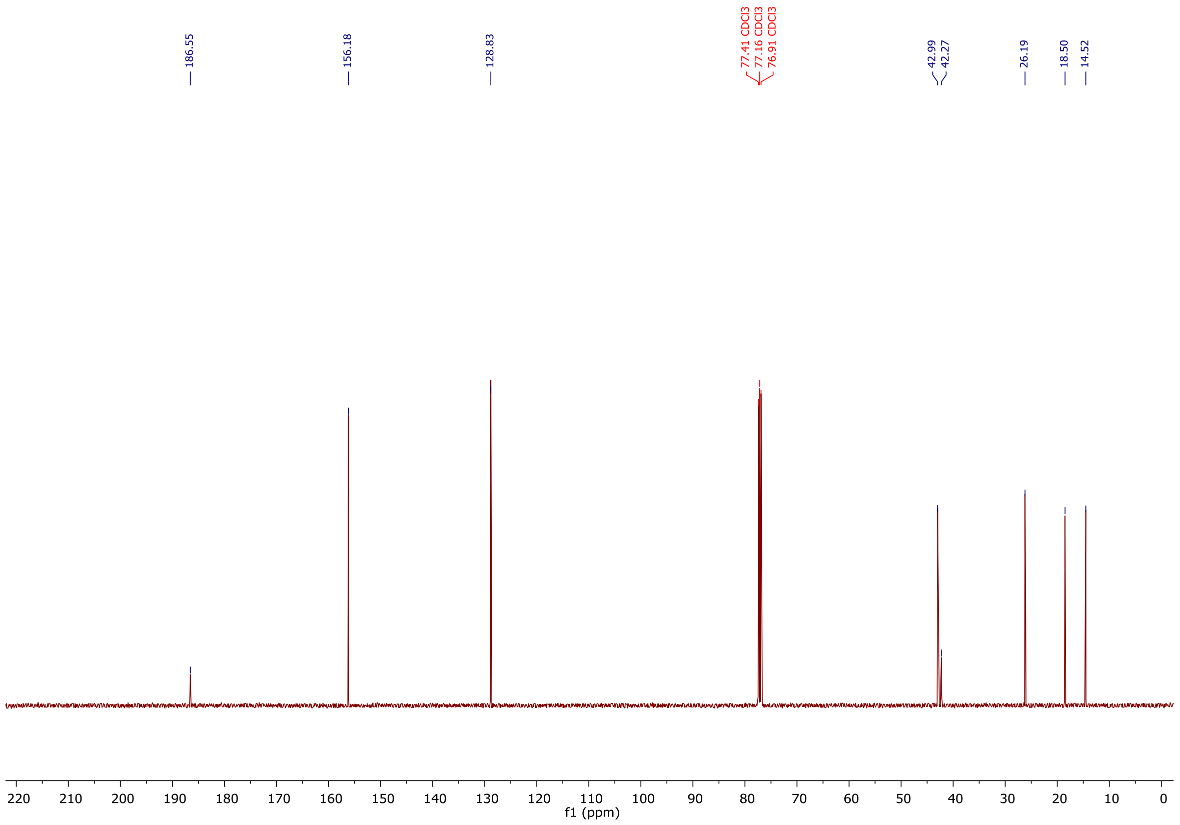

^{1}H NMR and ^{13}C NMR Spectra 1d

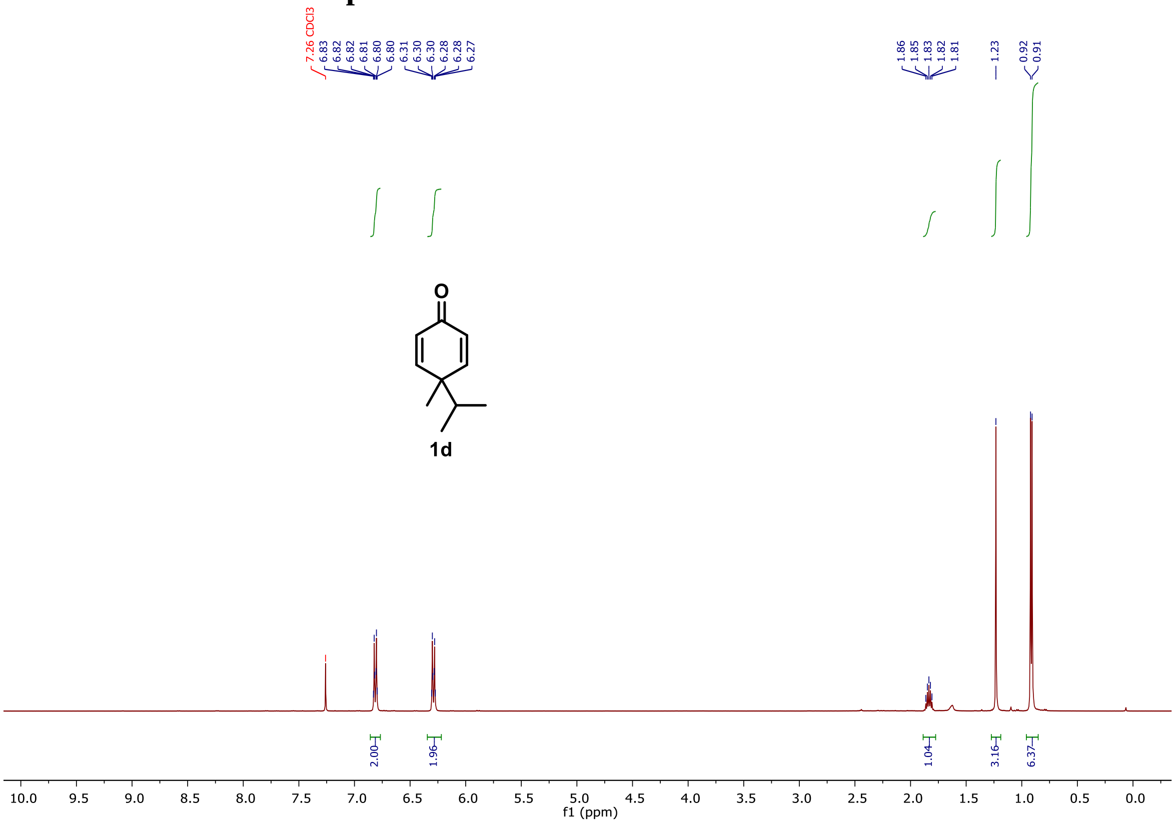

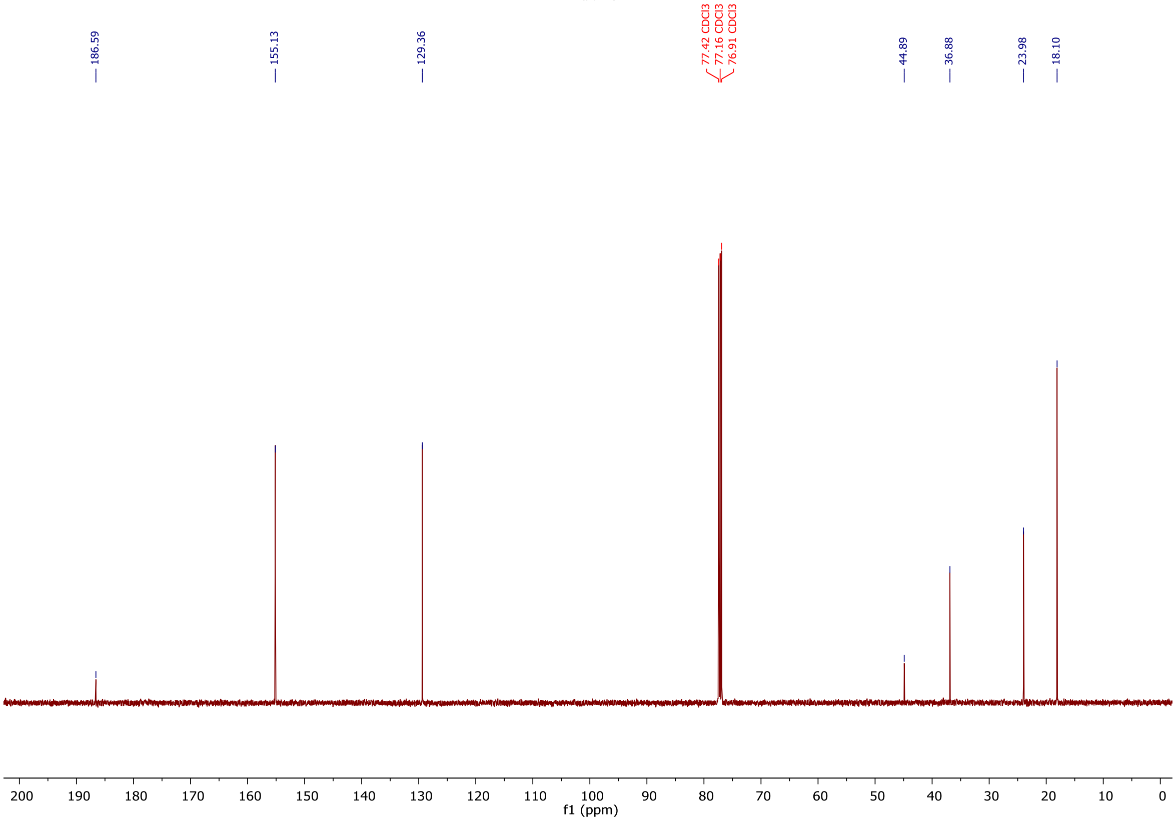

^{1}H NMR and ^{13}C NMR Spectra 1g

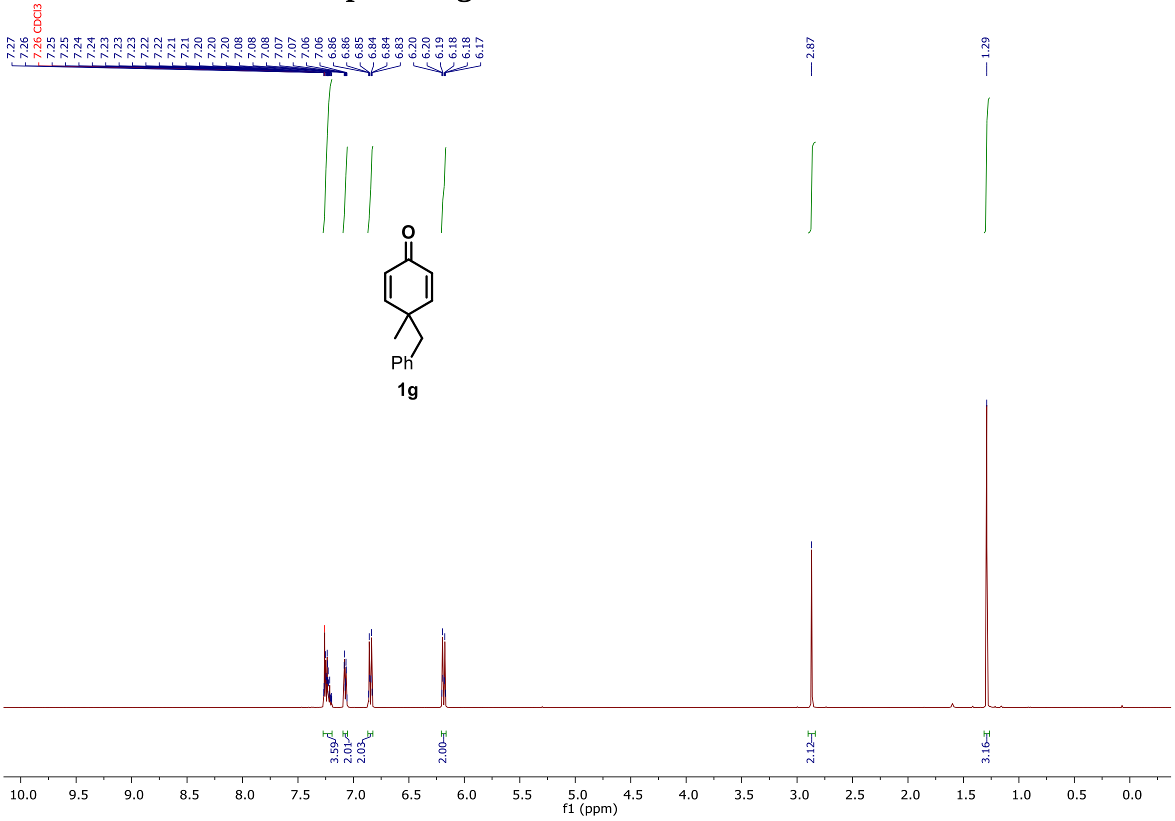

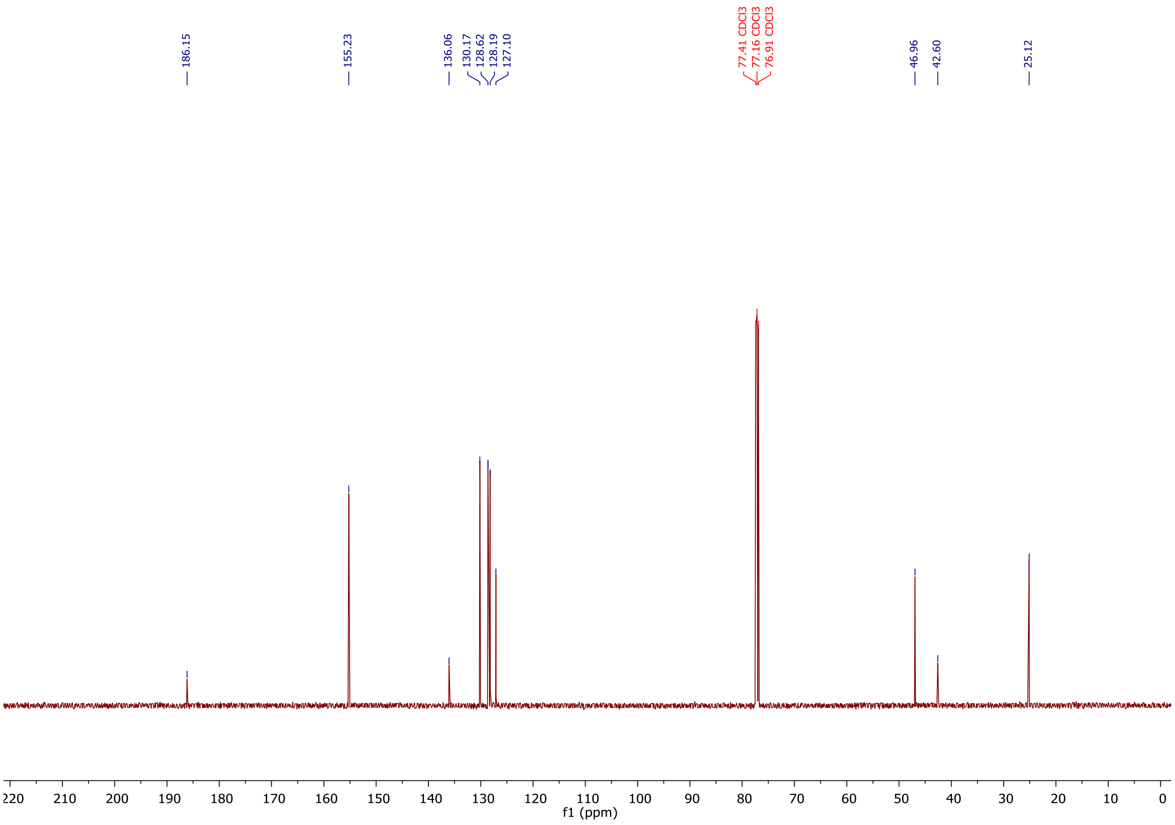

^{1}H NMR and ^{13}C NMR Spectra 1h

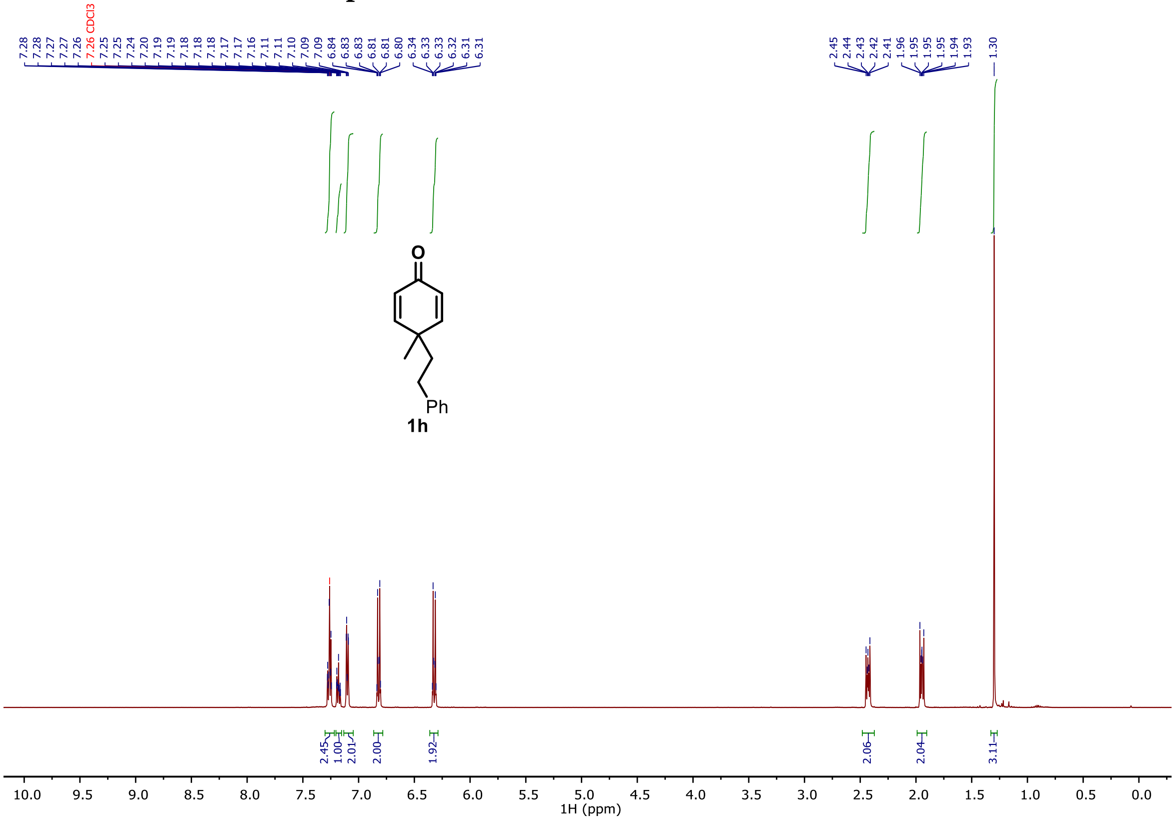

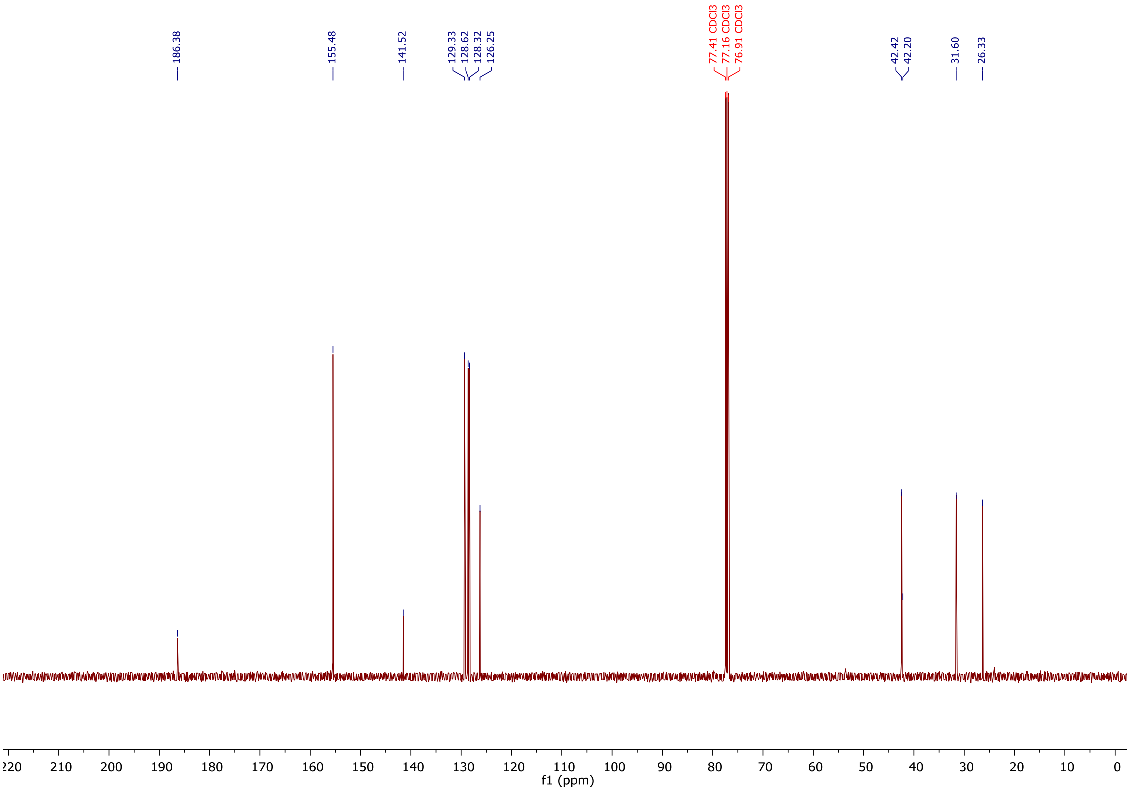

^{1}H NMR and ^{13}C NMR Spectra 2a

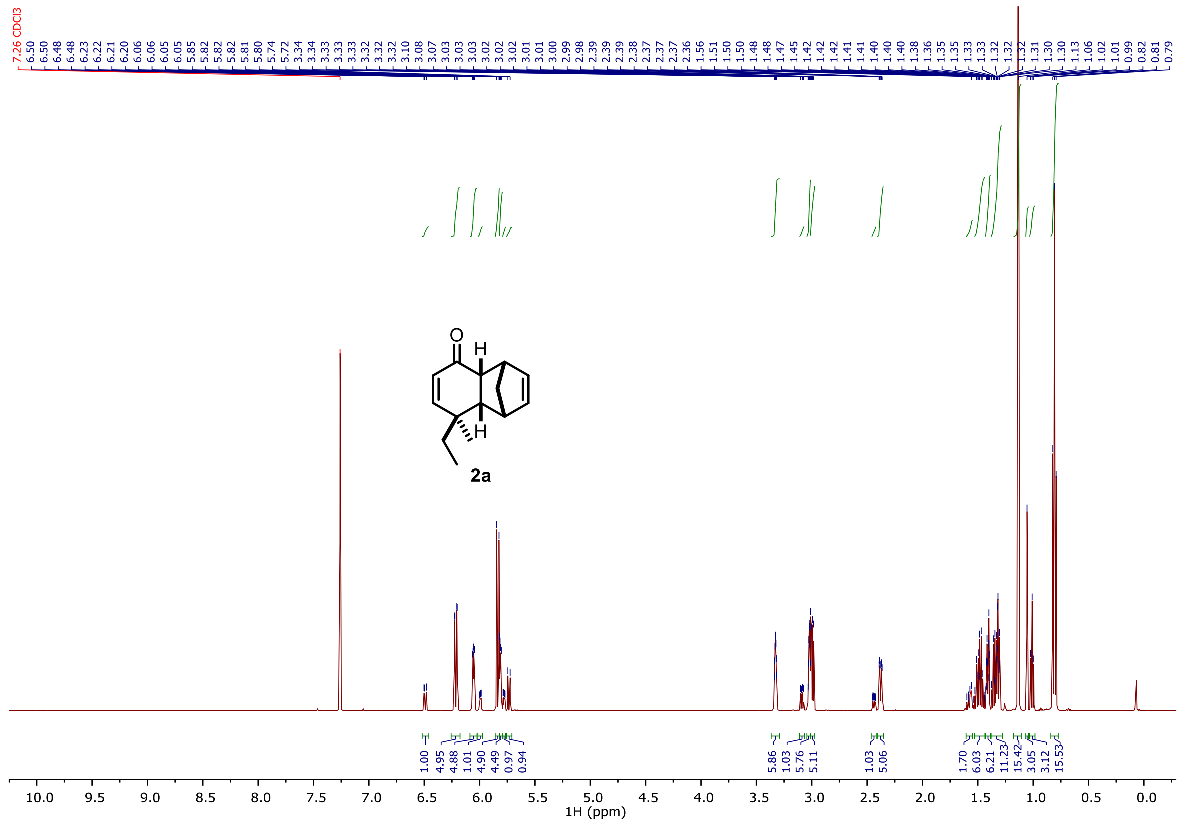

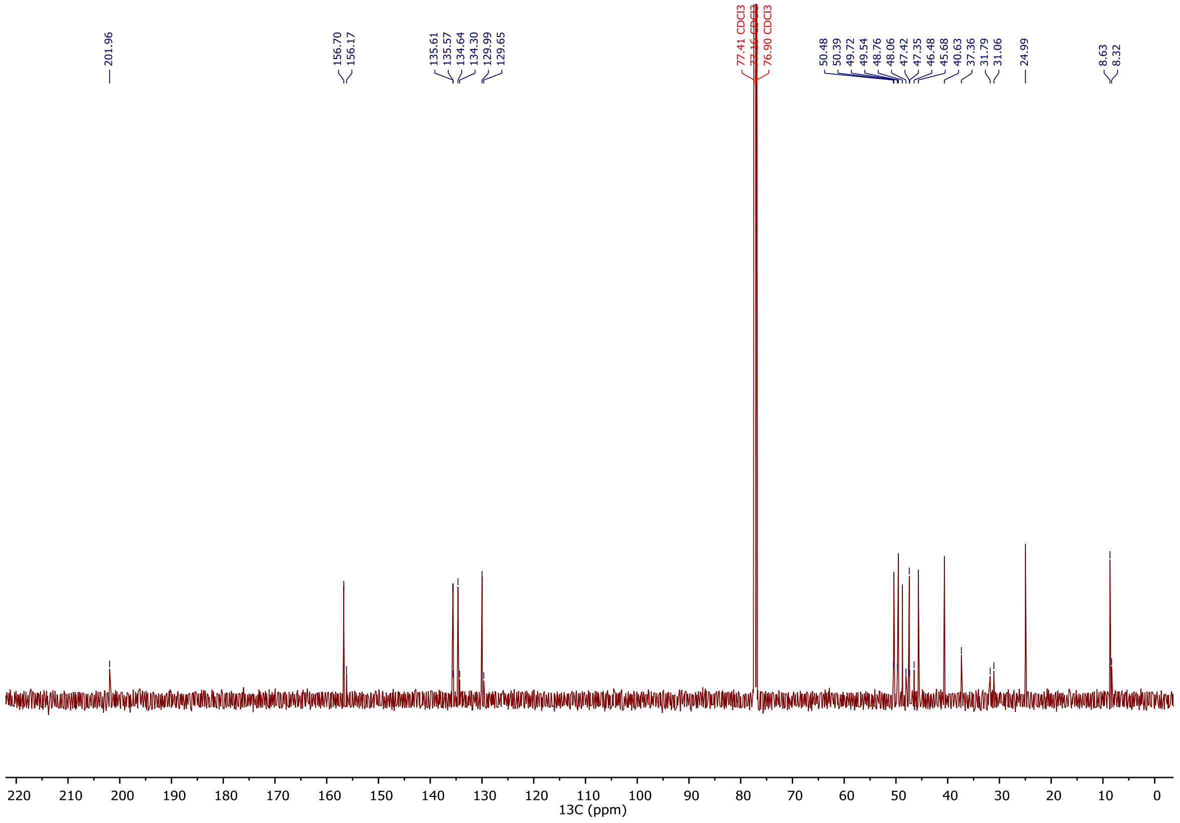

^{1}H NMR and ^{13}C NMR Spectra 2b

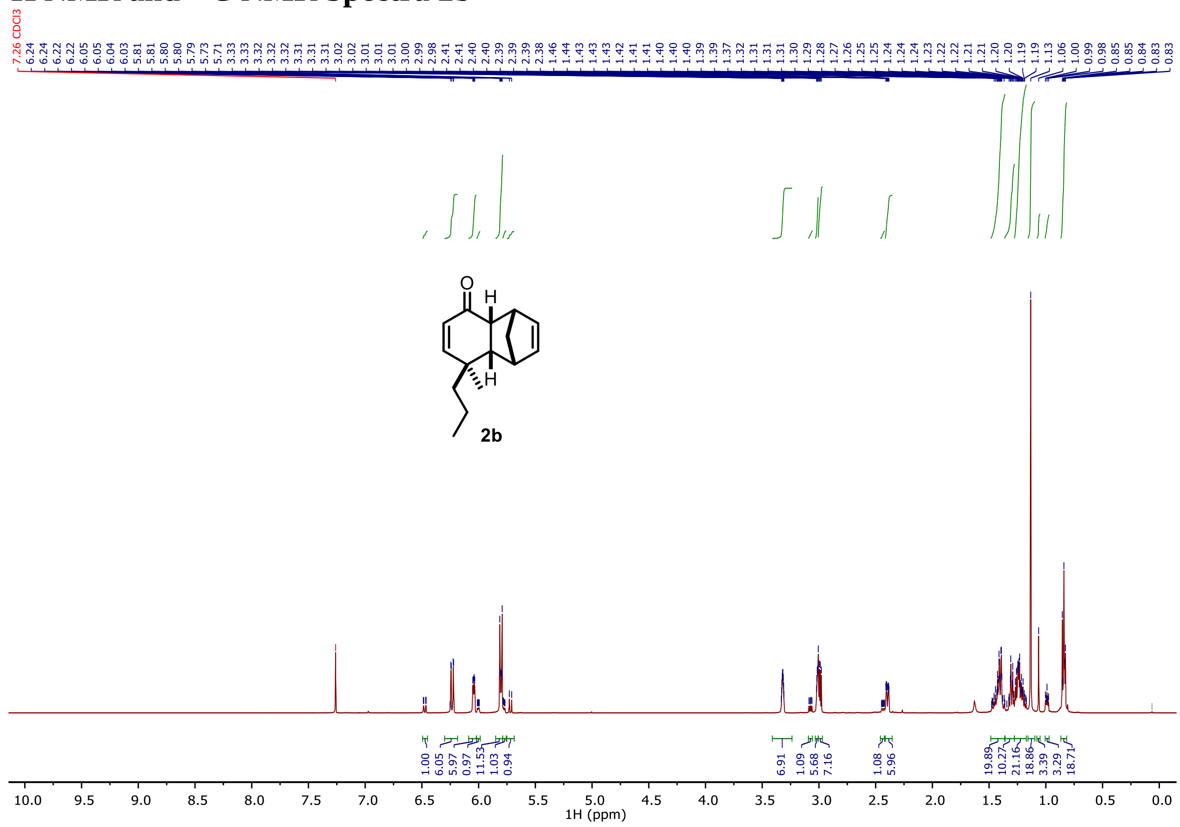

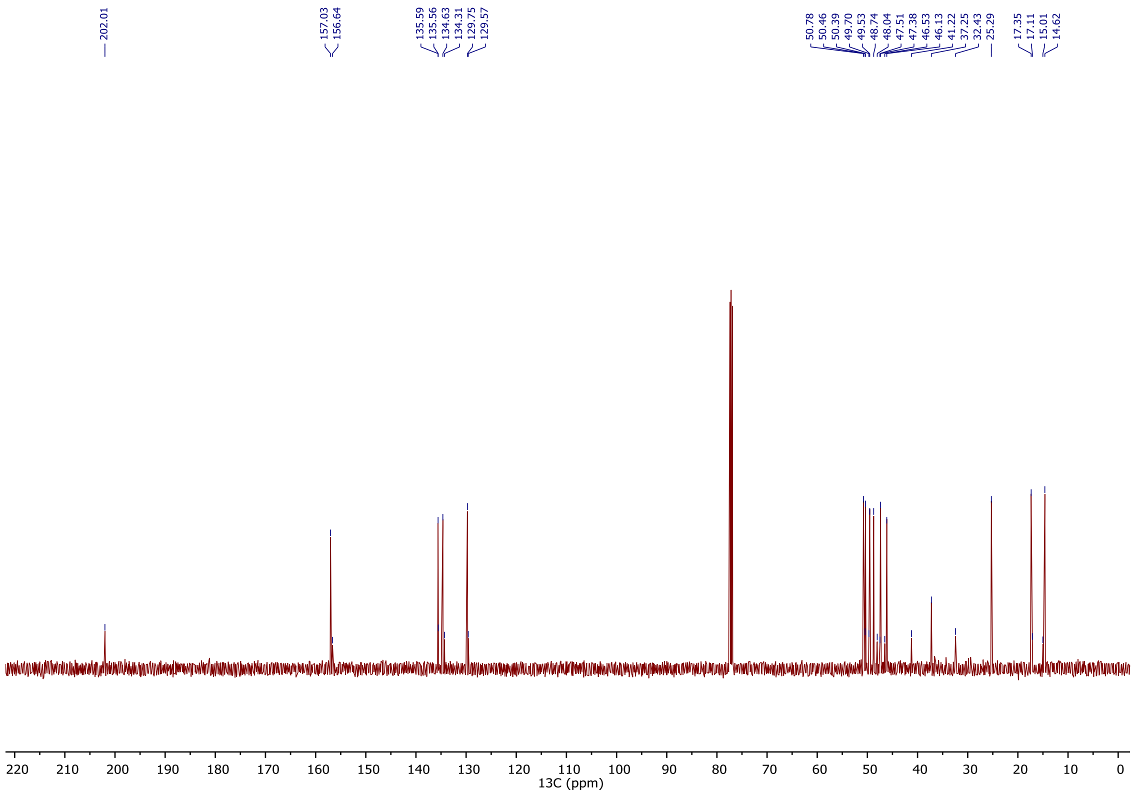

^{1}H NMR and ^{13}C NMR Spectra 2c

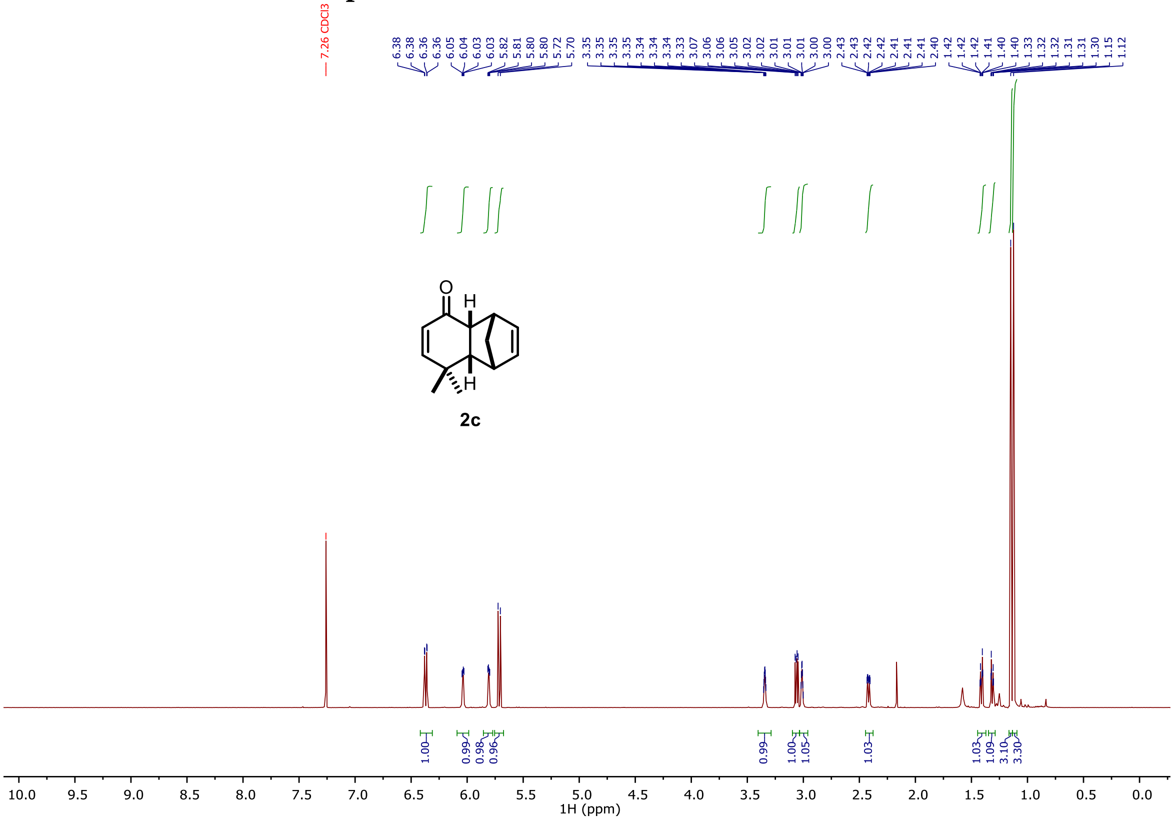

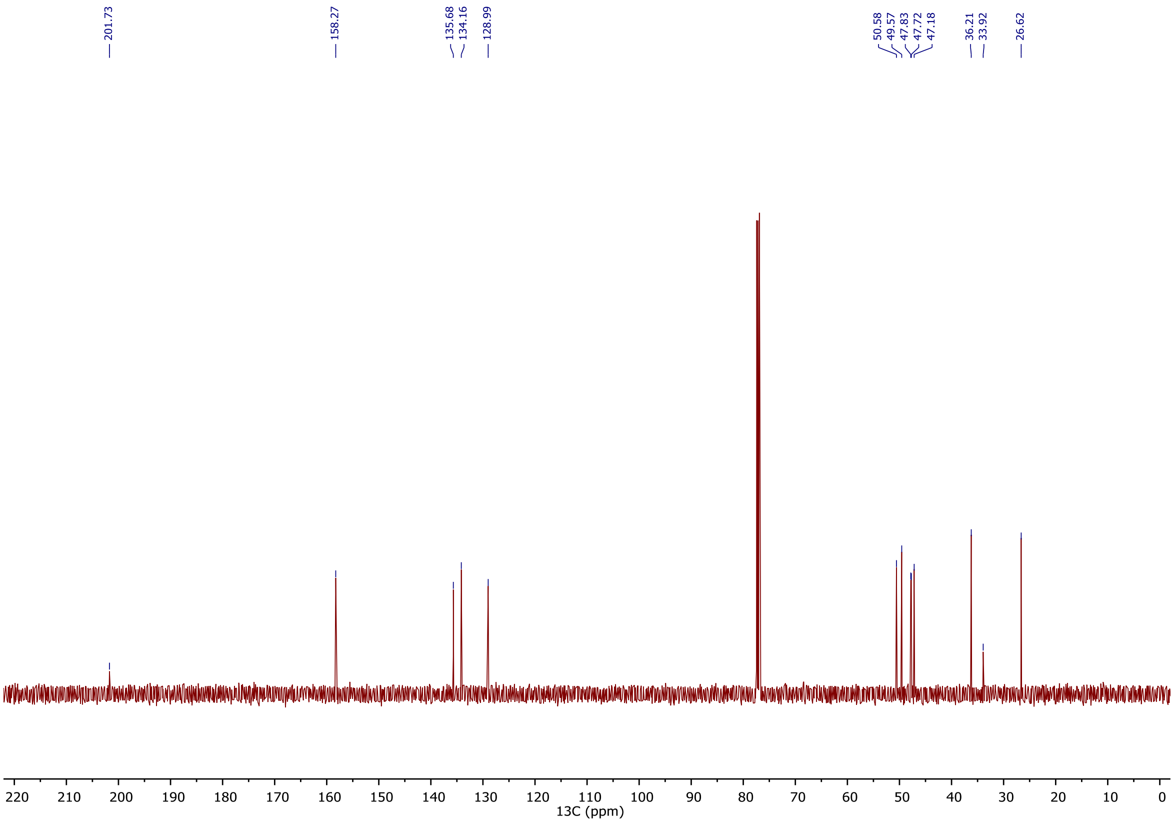

^{1}H NMR and ^{13}C NMR Spectra 2d

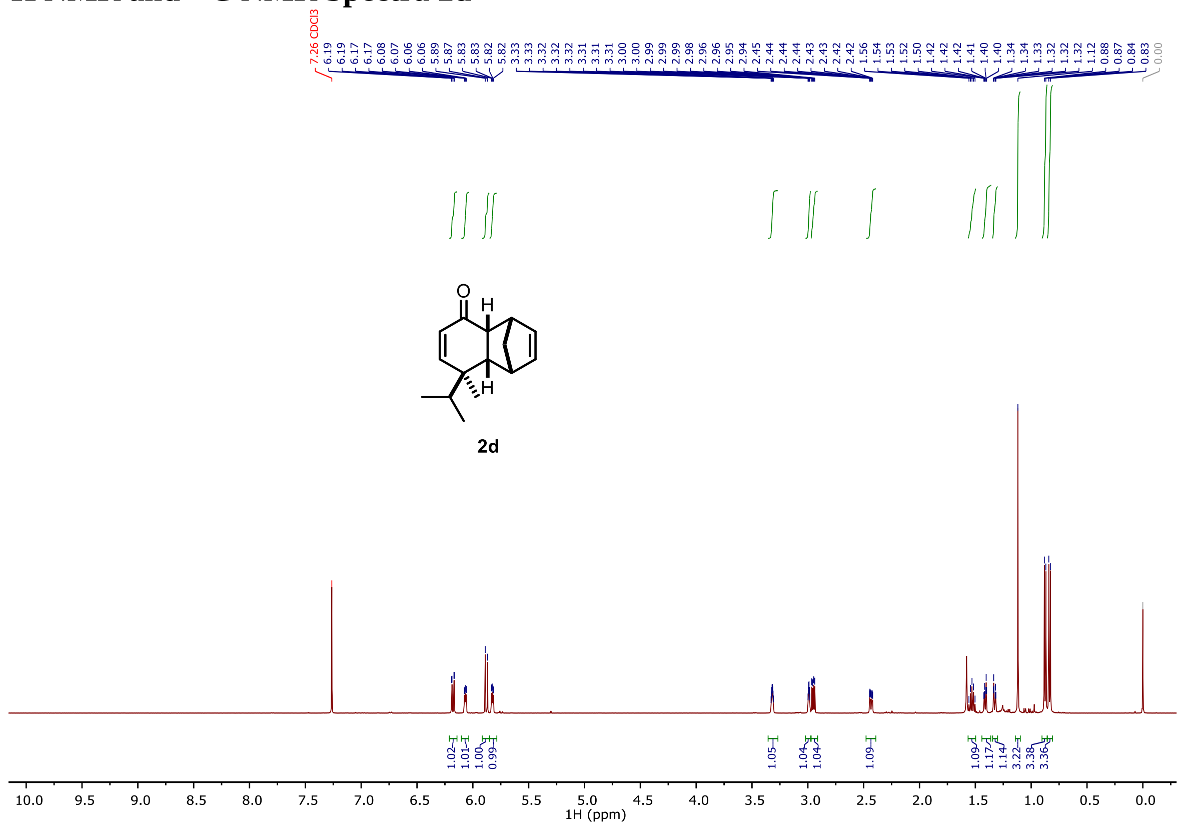

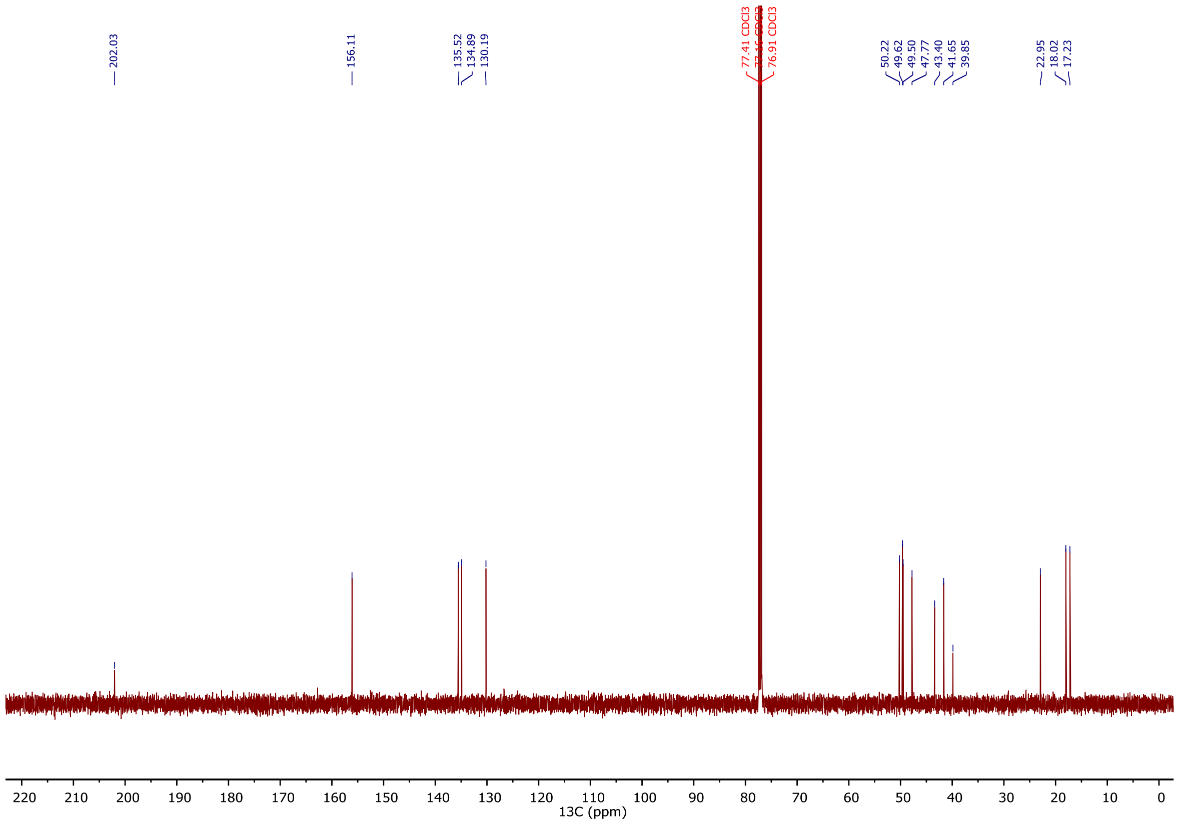

^{1}H NMR and ^{13}C NMR Spectra 2e

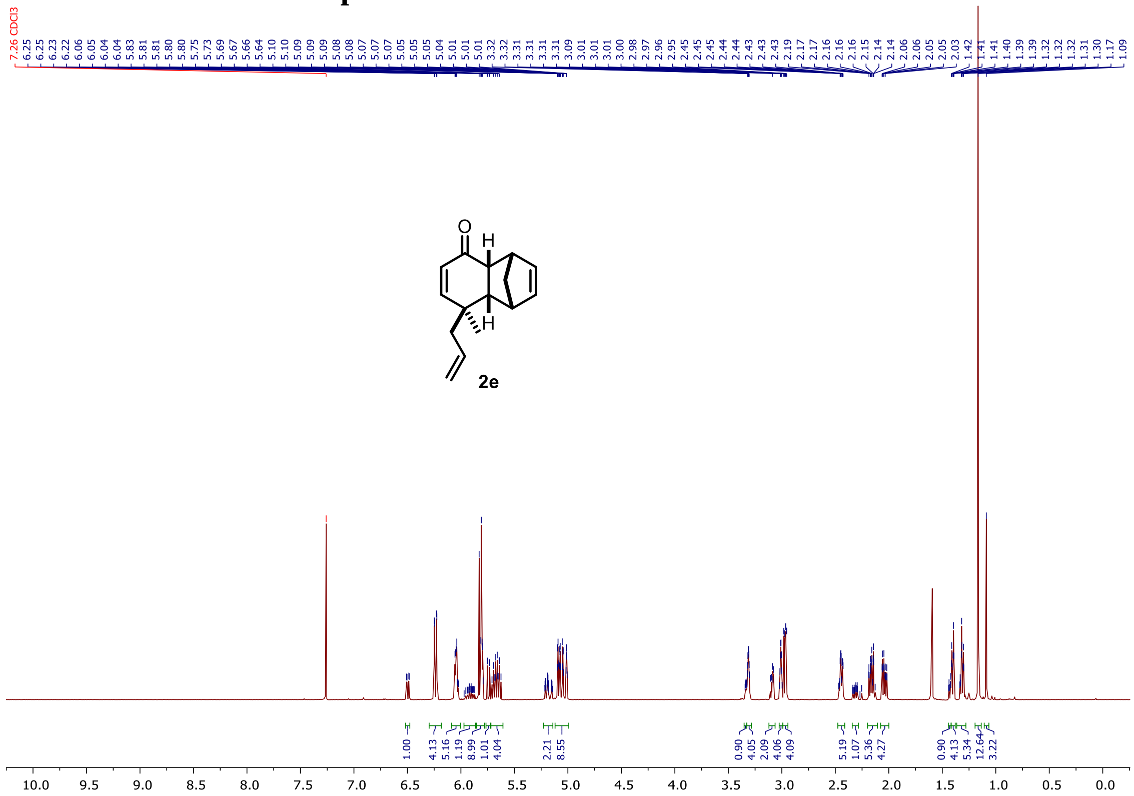

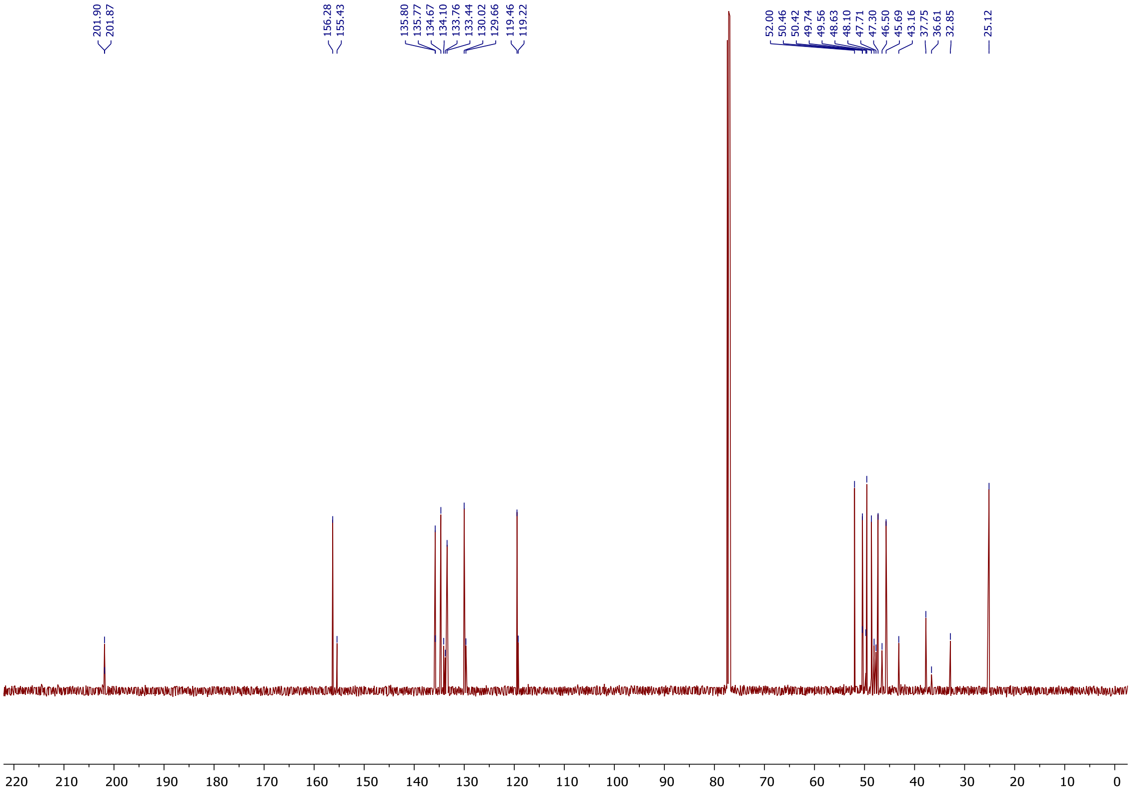

^{1}H NMR and ^{13}C NMR Spectra 2f

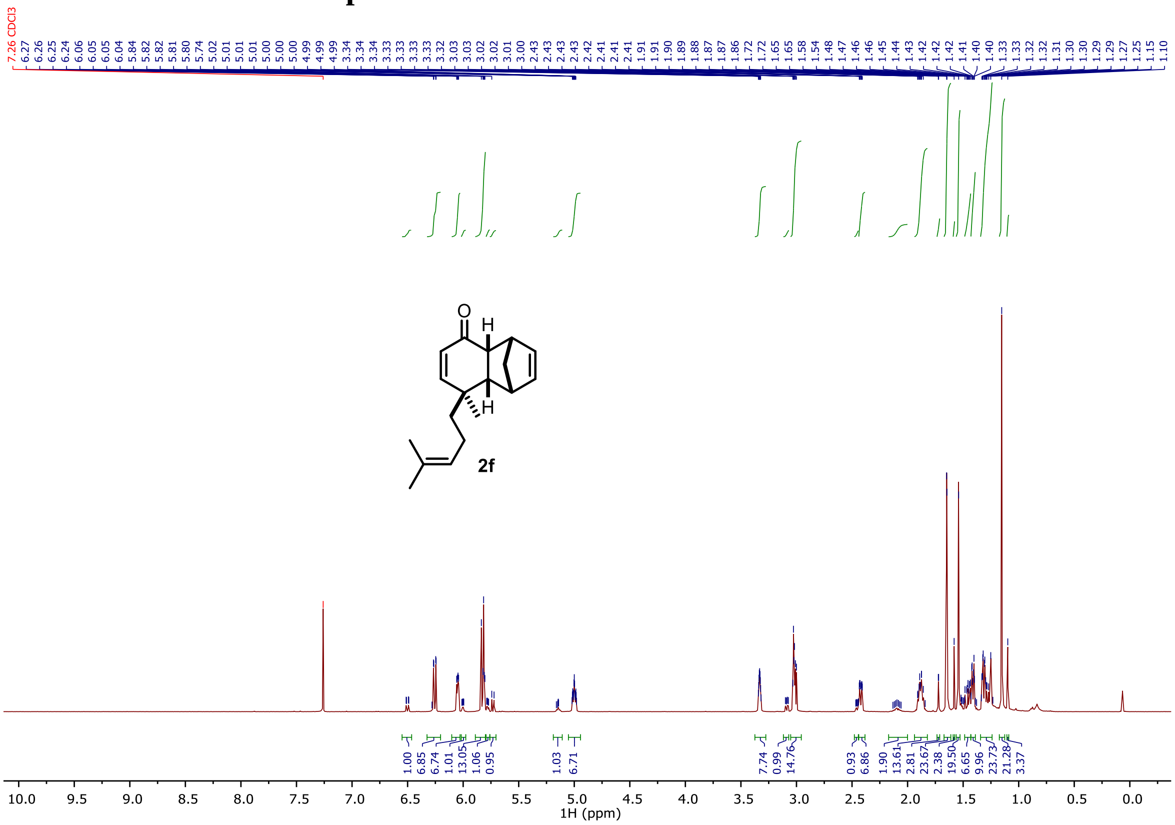

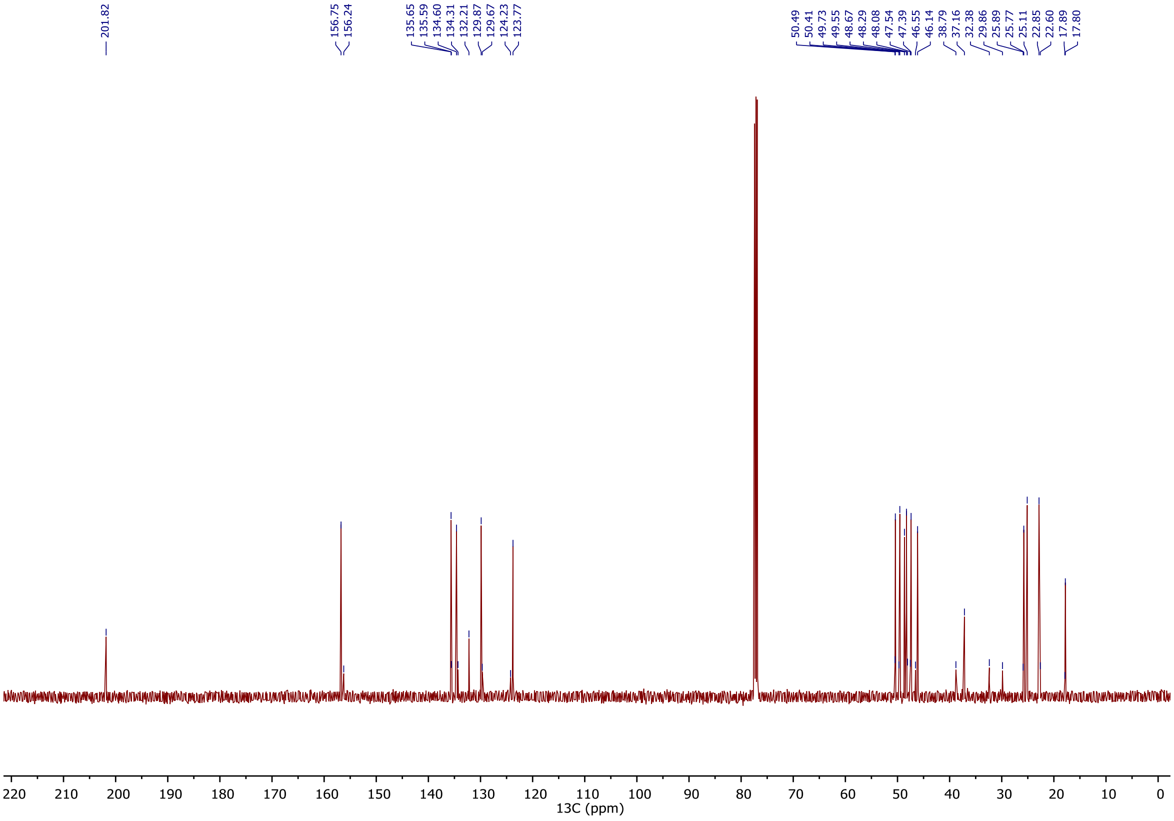

^{1}H NMR and ^{13}C NMR Spectra 2g

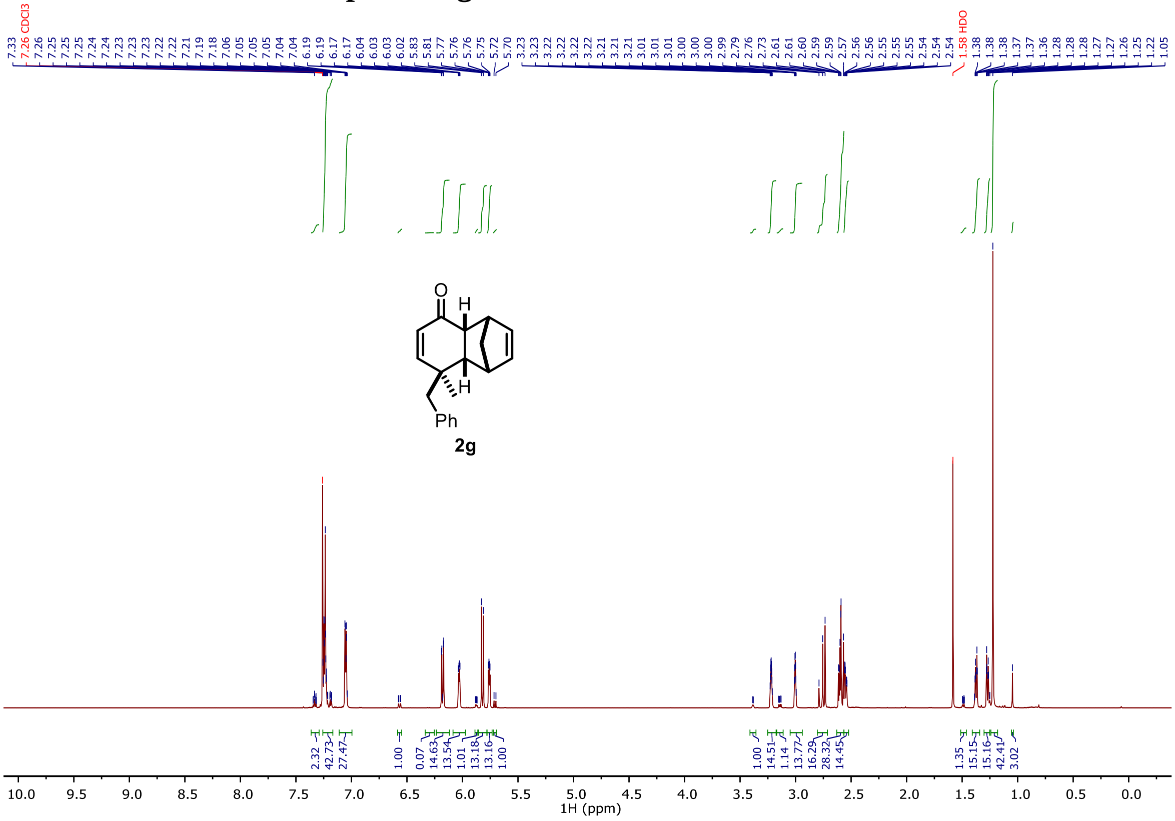

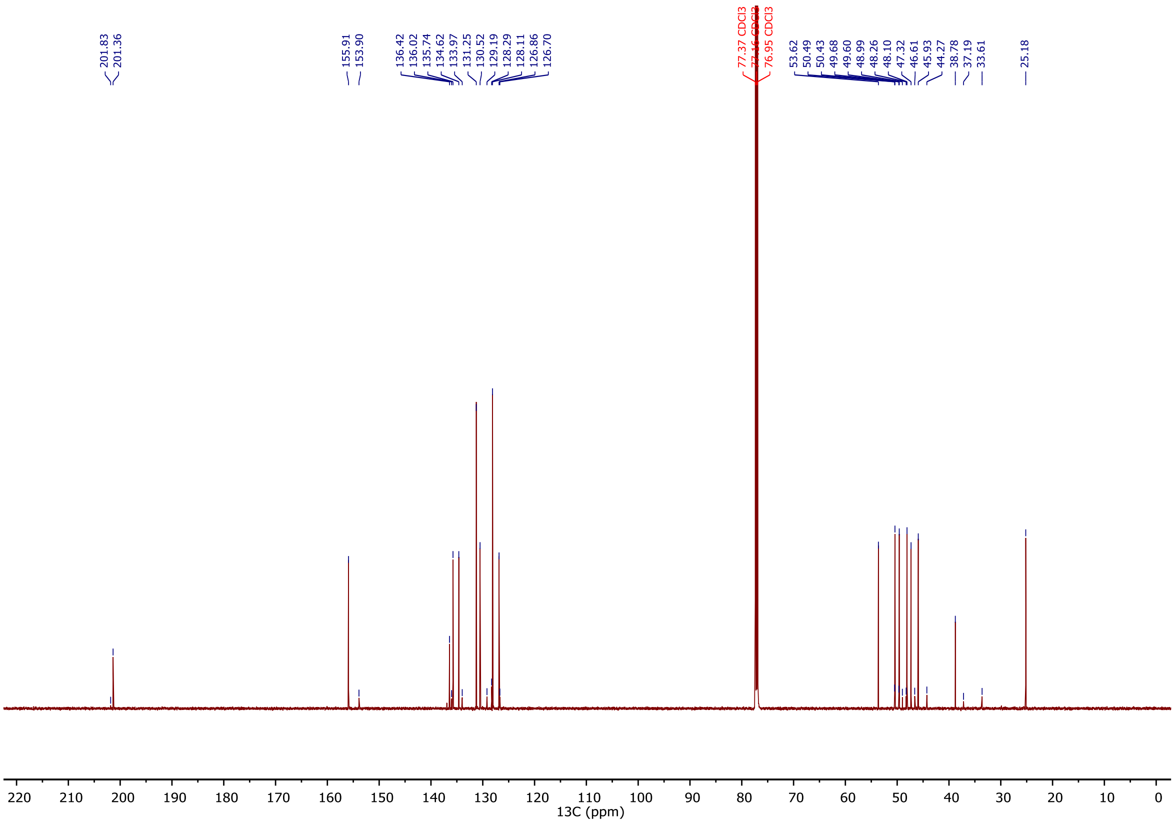

COSY spectra 2g

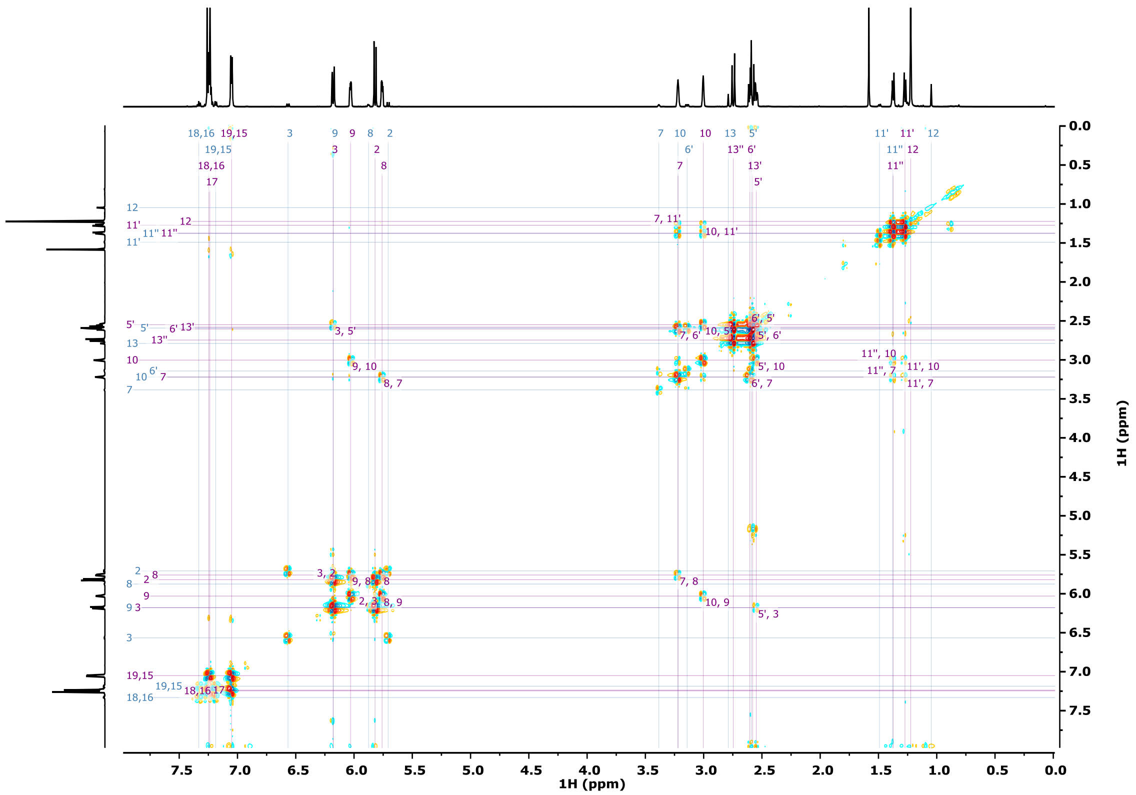

HSQC spectra 2g

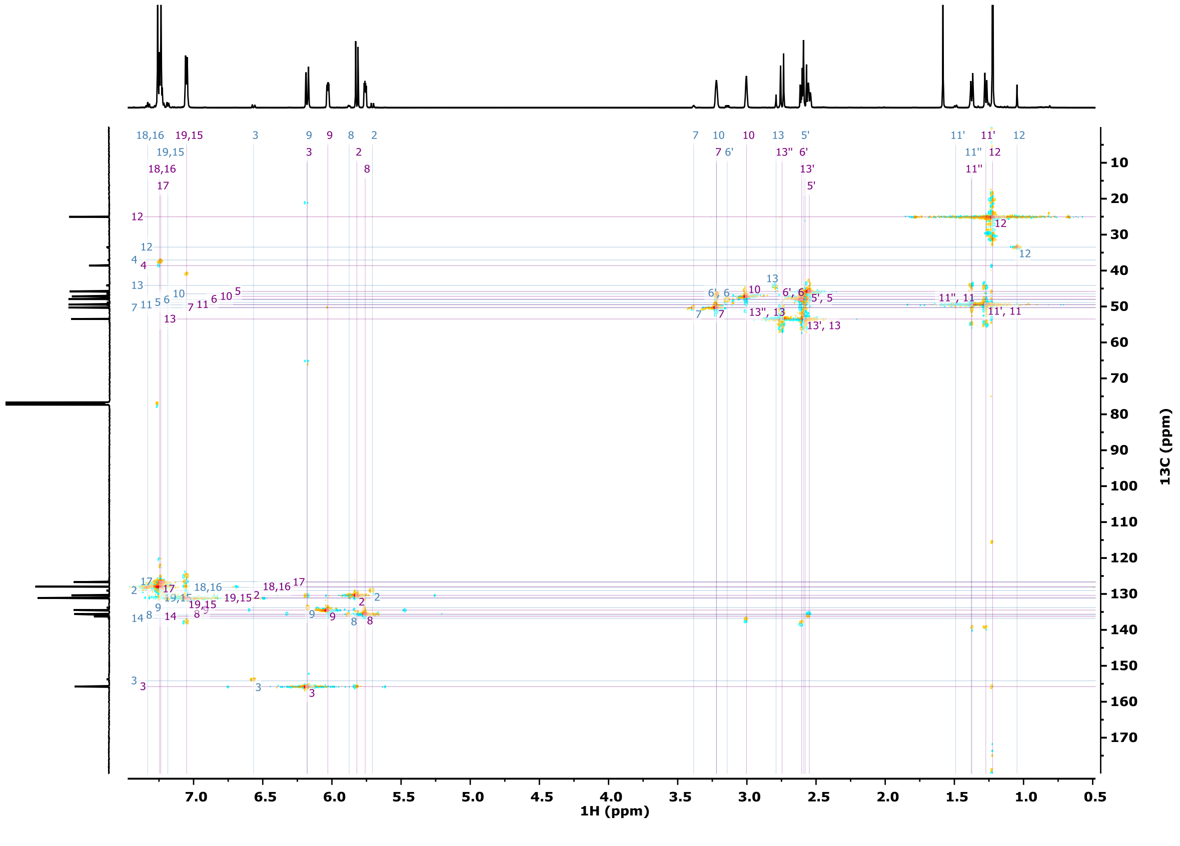

HMBC spectra 2g

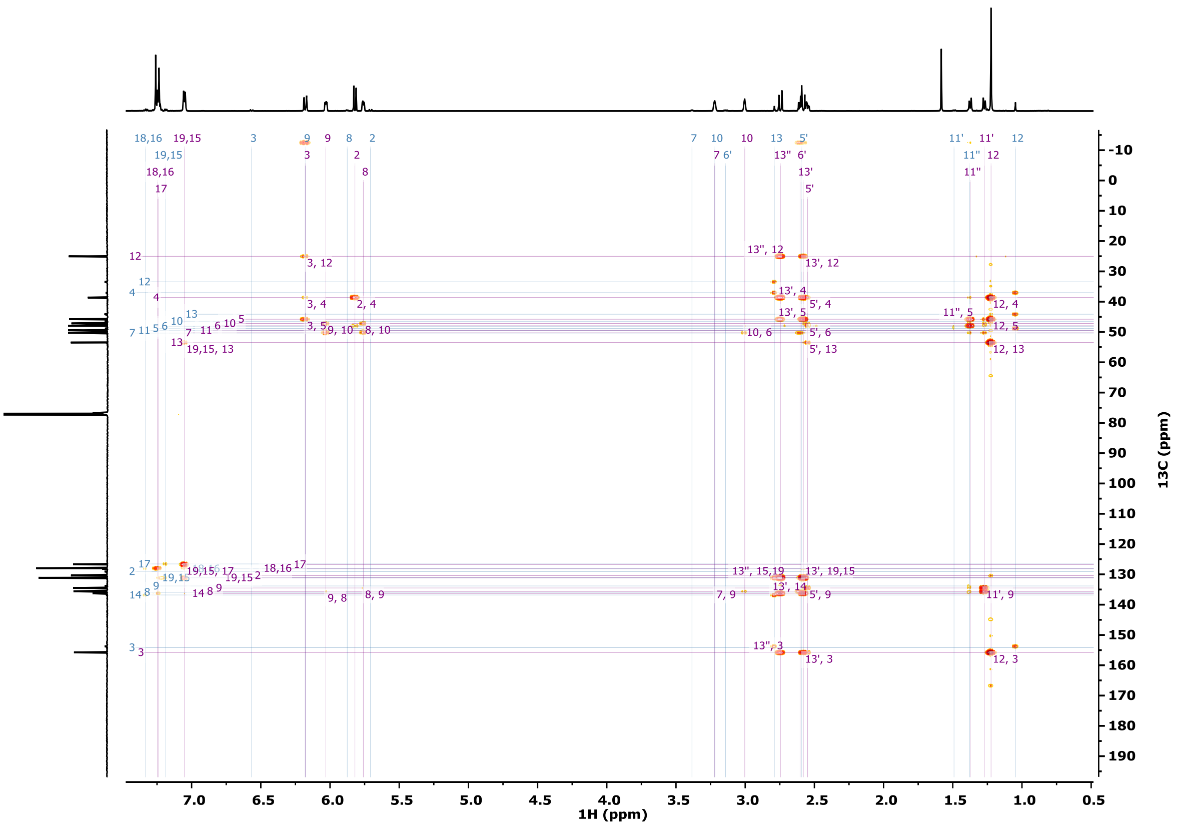

NOESY spectra 2g

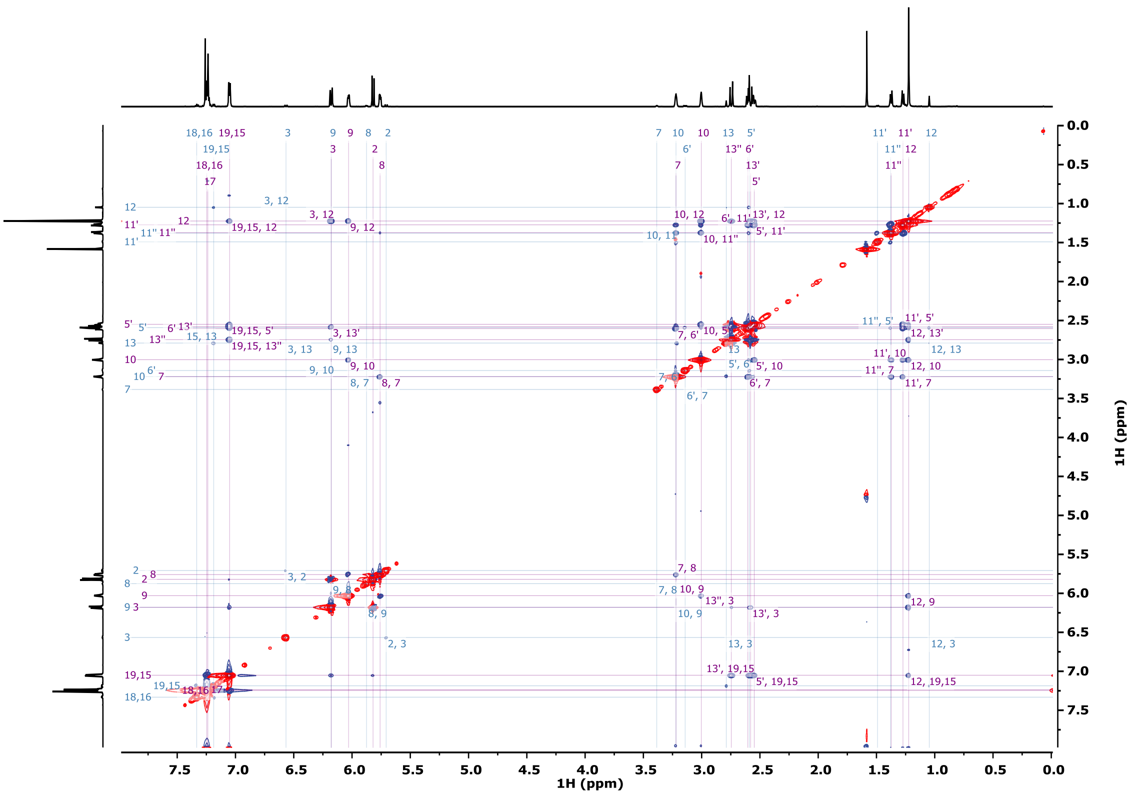

^{1}H NMR and ^{13}C NMR Spectra 2h

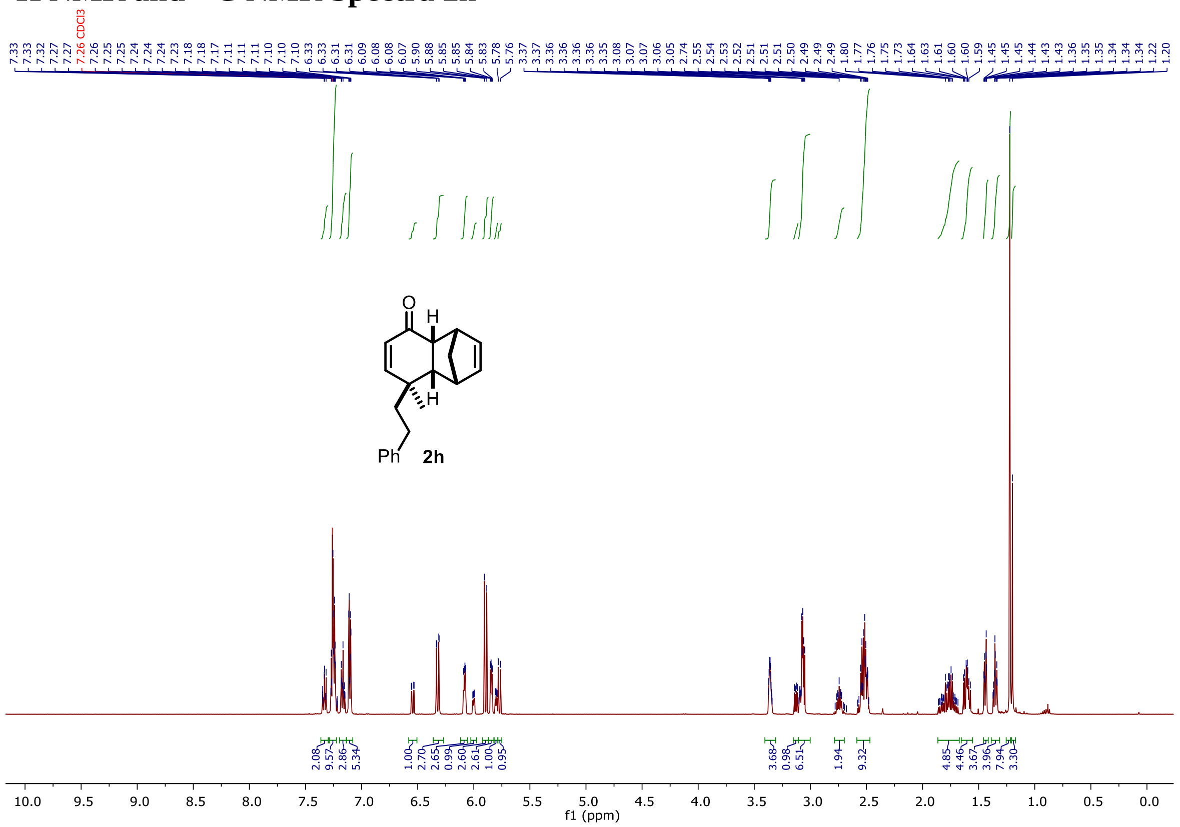

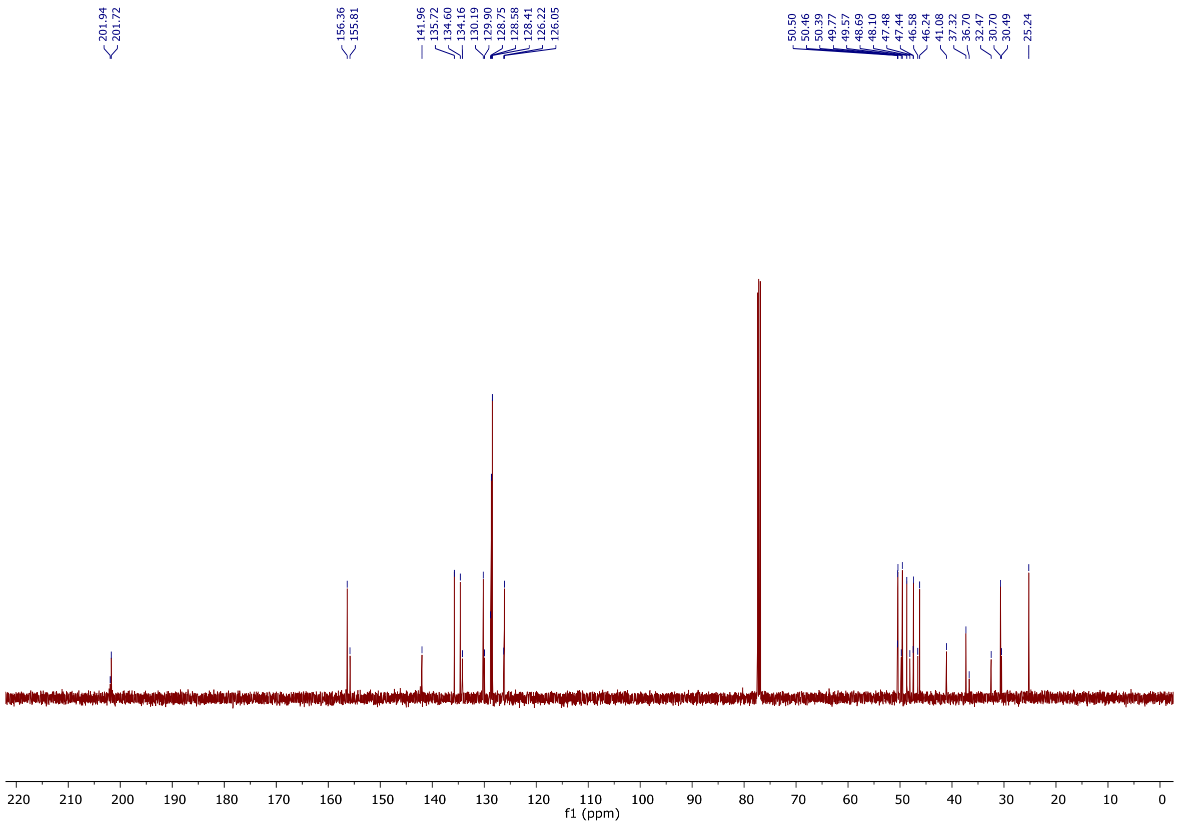

^{1}H NMR and ^{13}C NMR Spectra 2i′

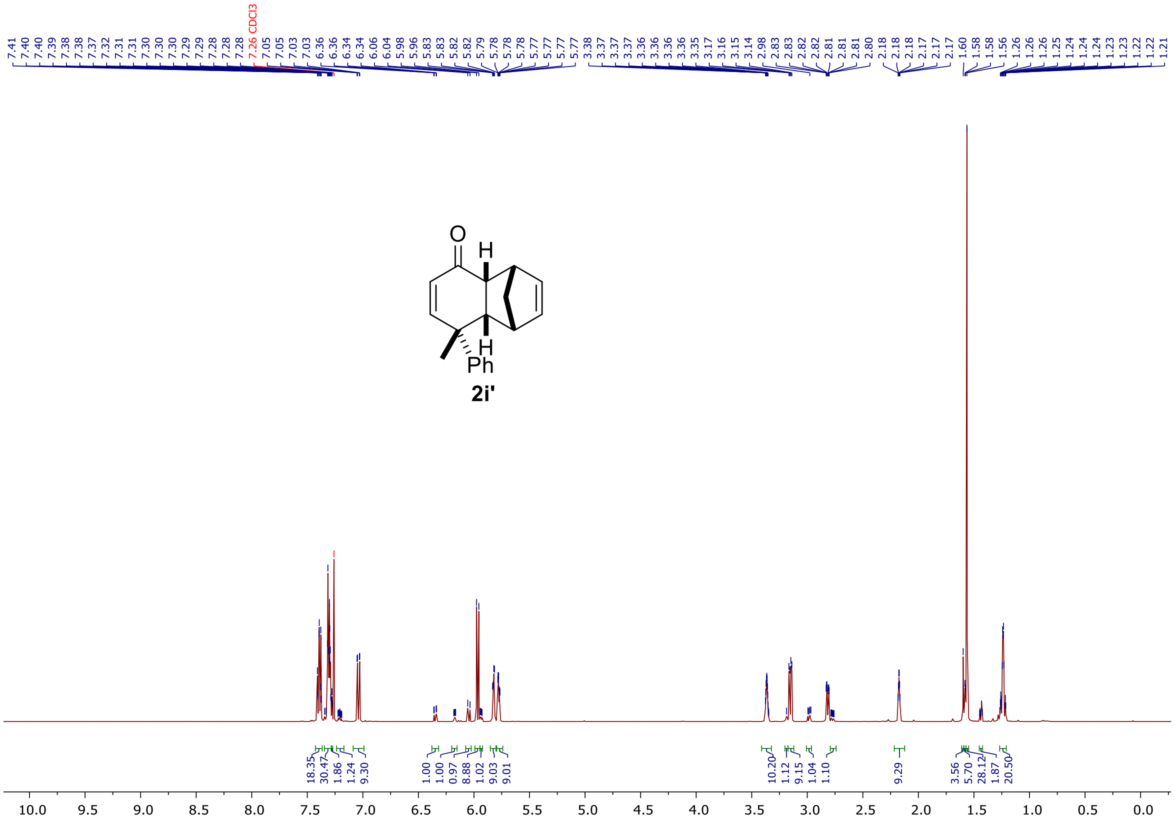

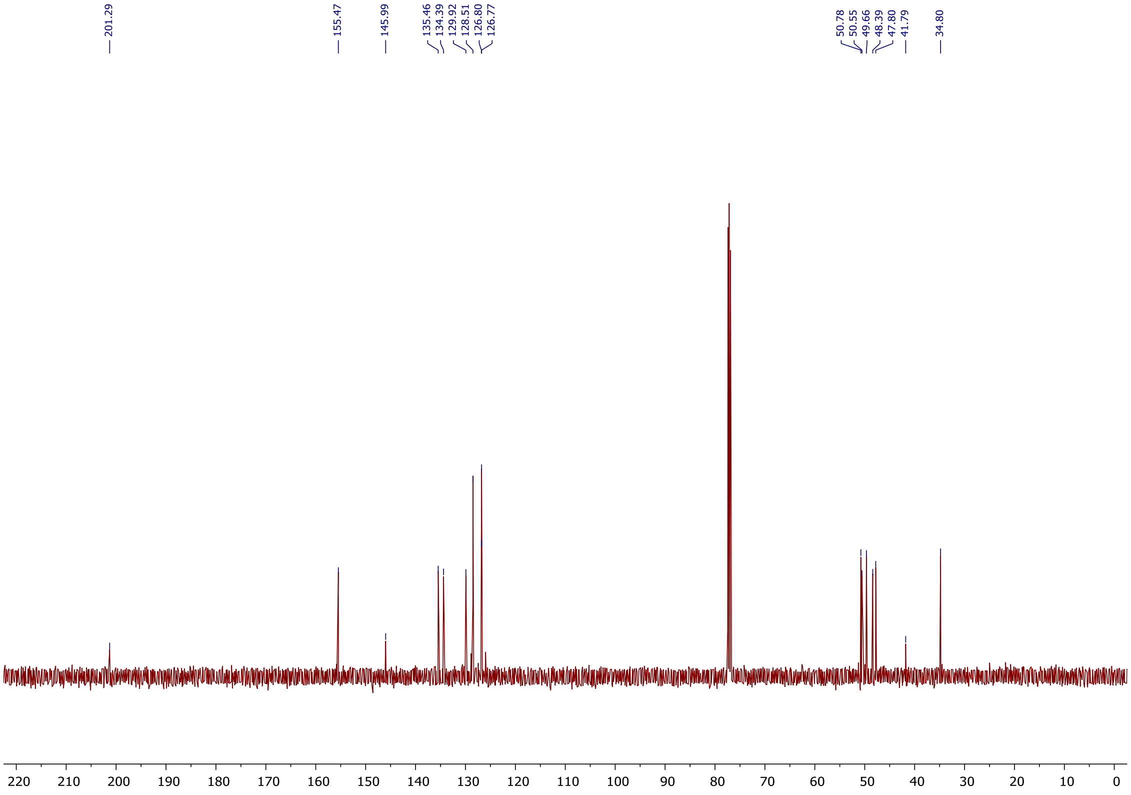

COSY spectra 2i′

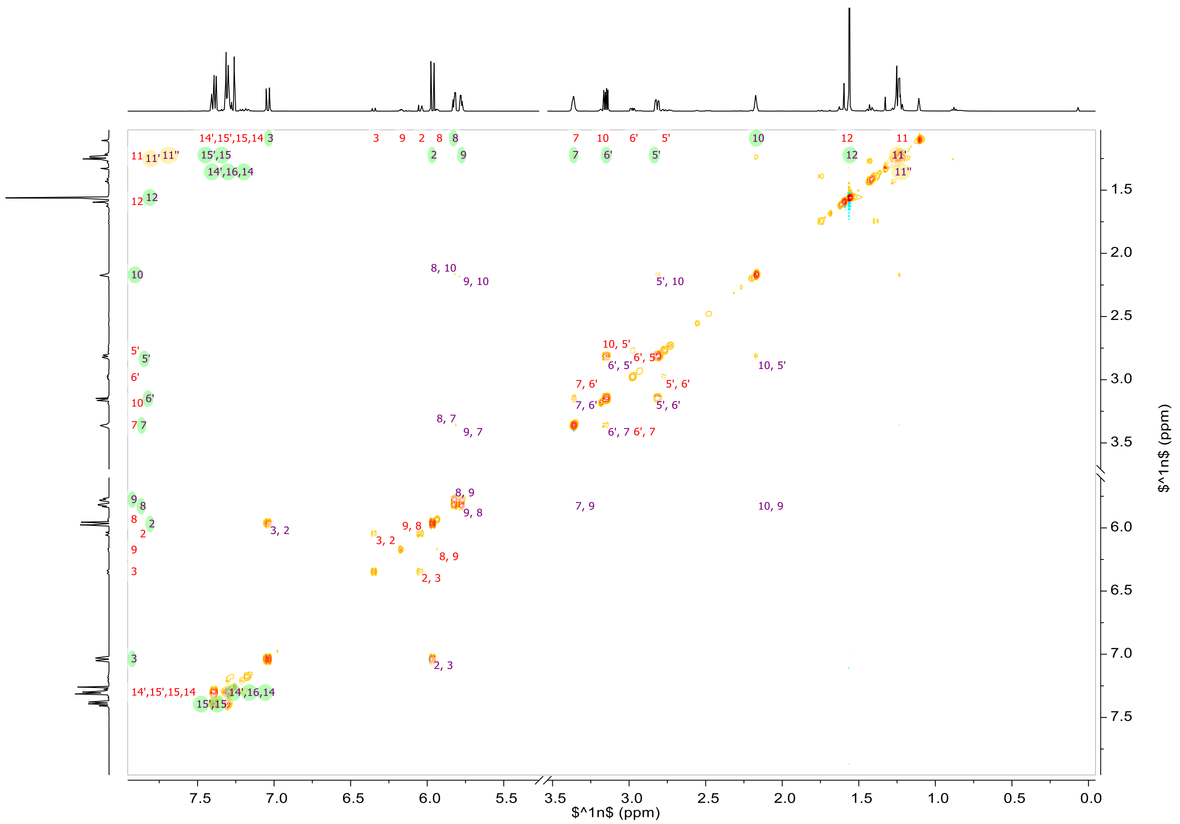

HSQC Spectra 2i′

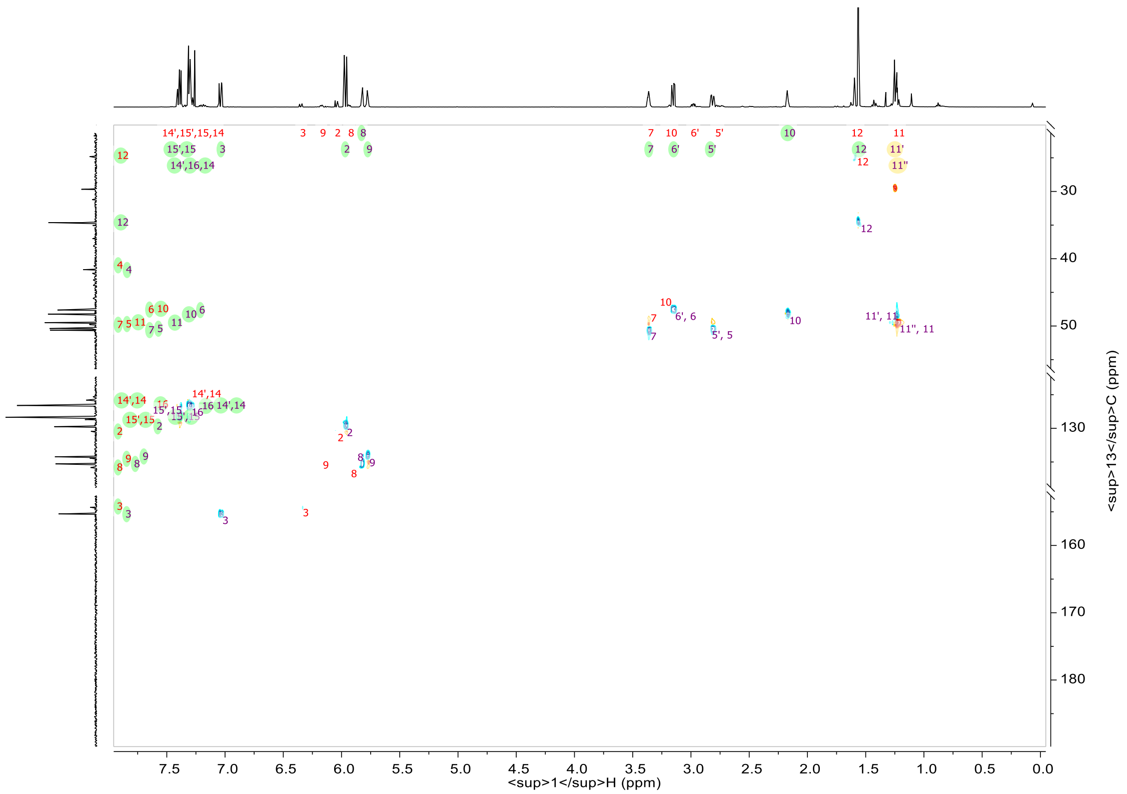

HMBC Spectra 2i′

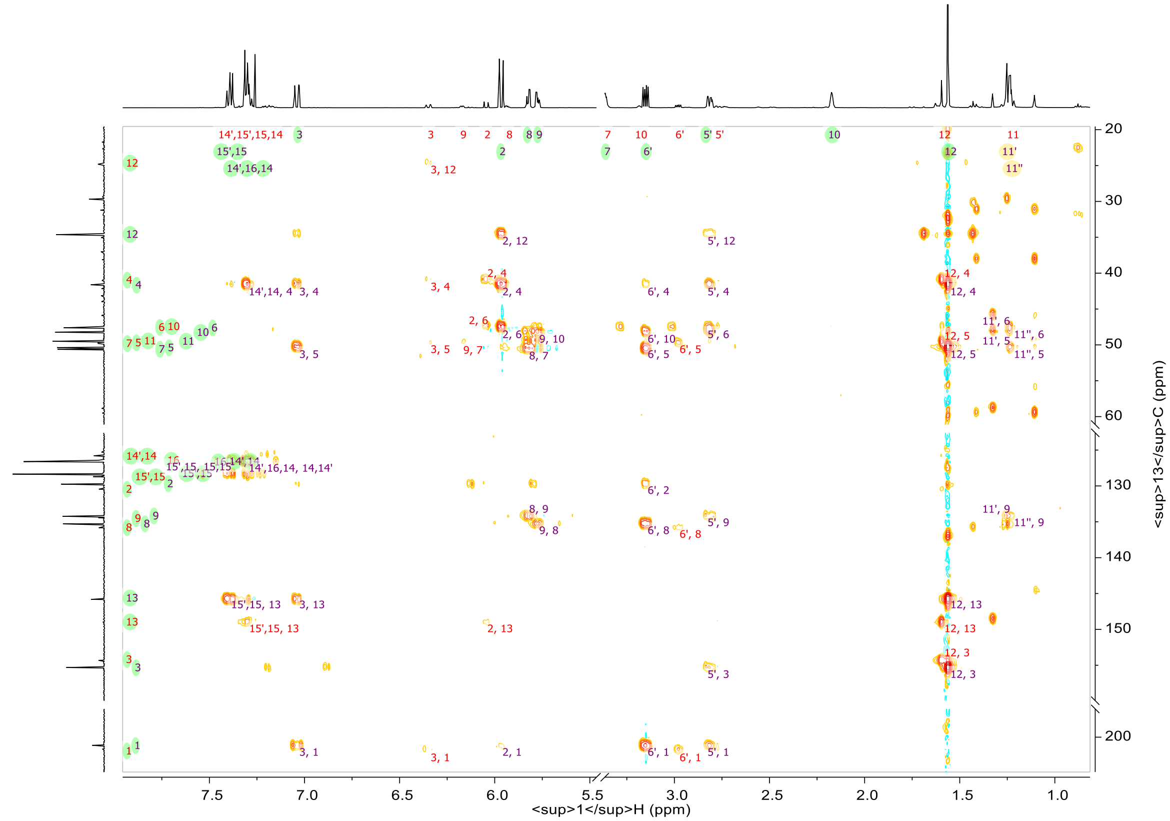

NOESY Spectra 2i′

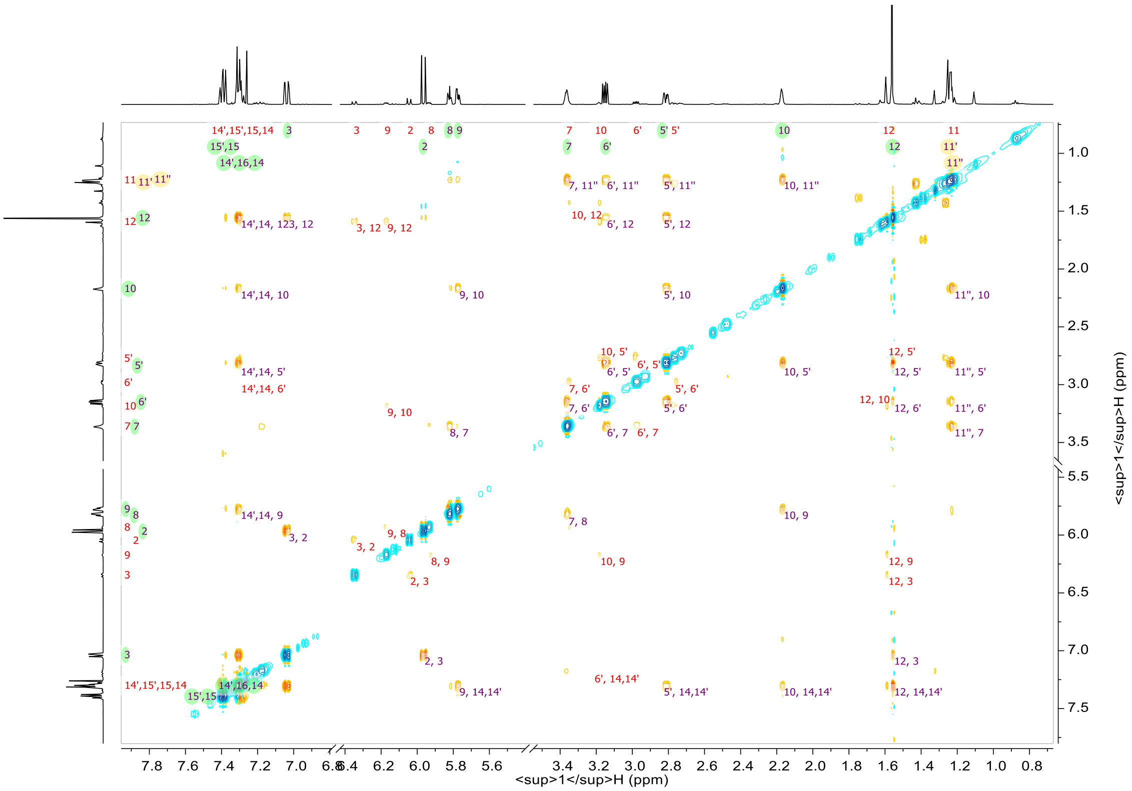

^{1}H NMR and ^{13}C NMR Spectra 2j′

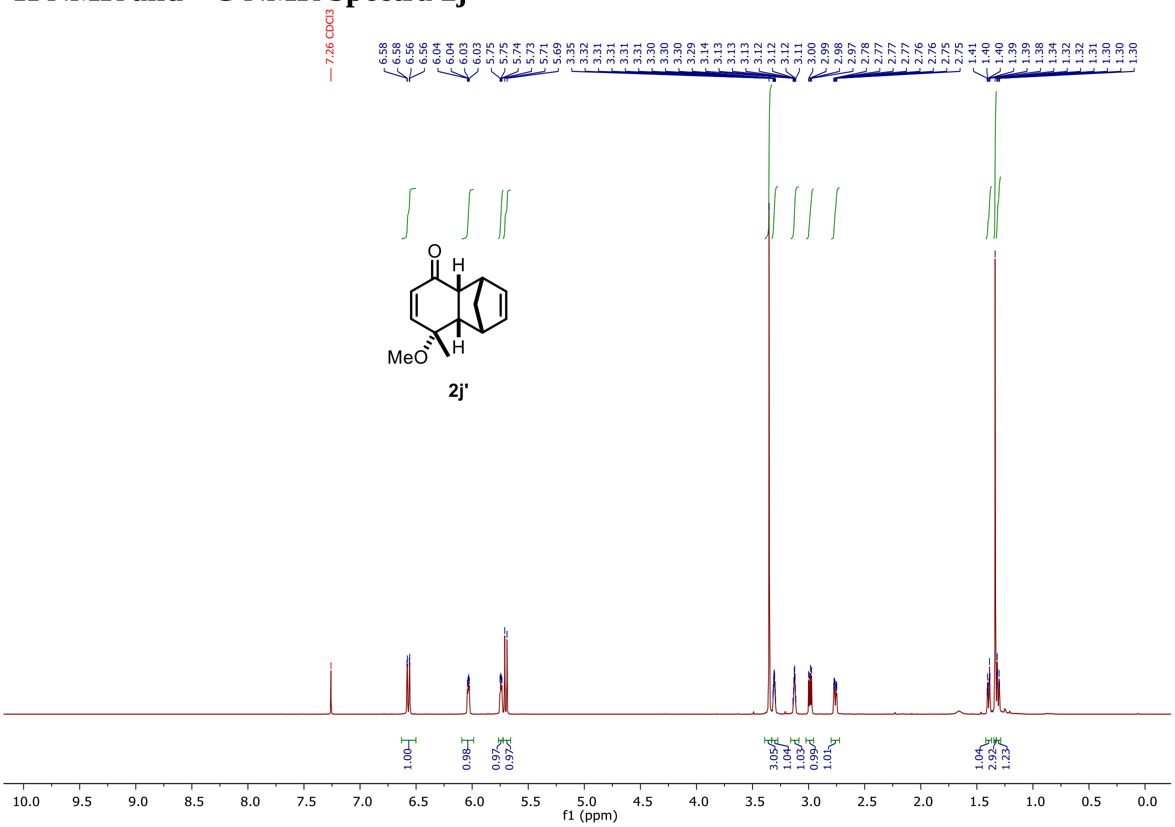

2j'

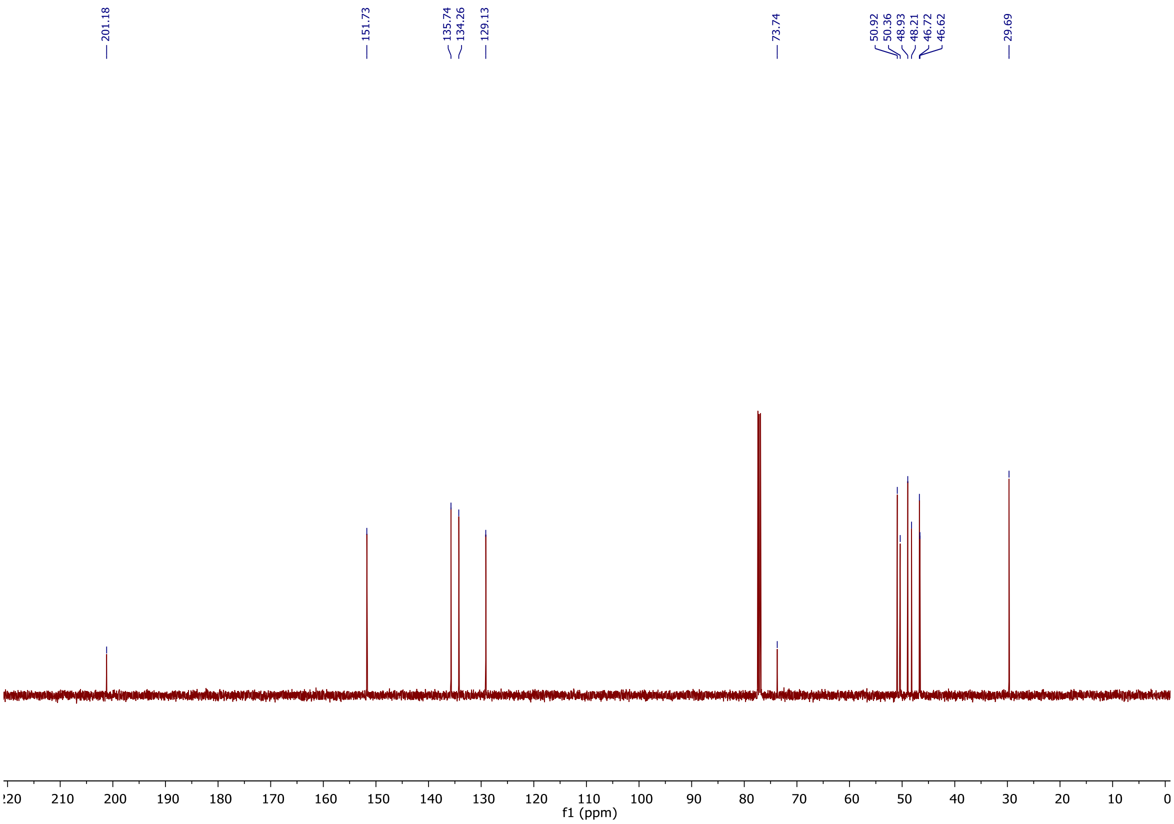

COSY spectra 2j′

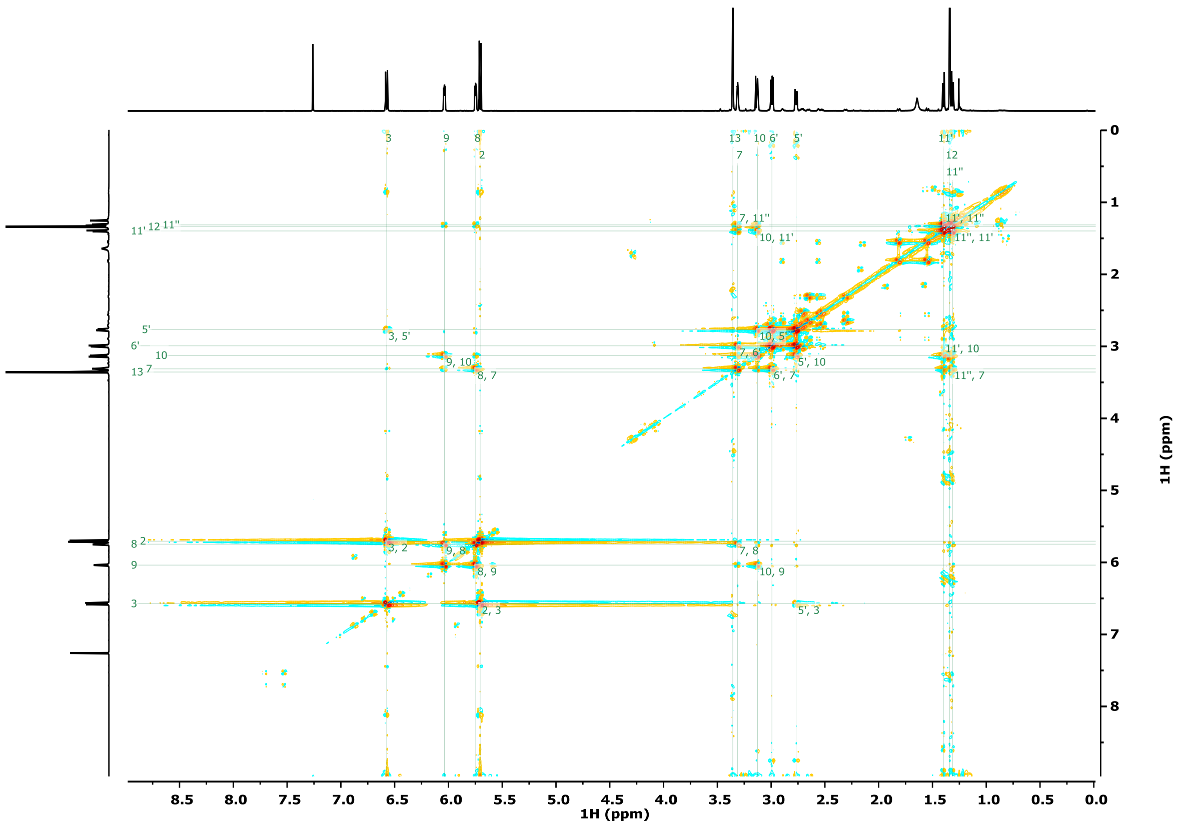

HSQC Spectra 2j′

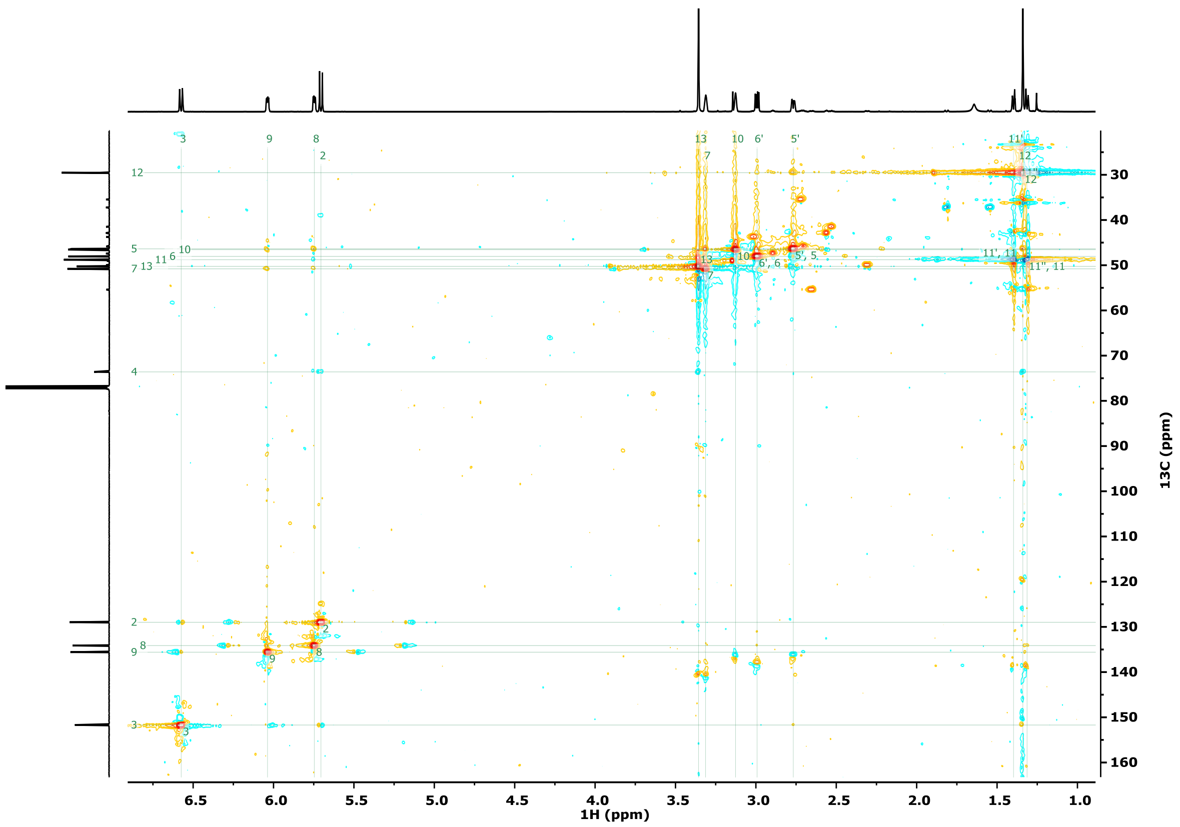

HMBC Spectra 2j′

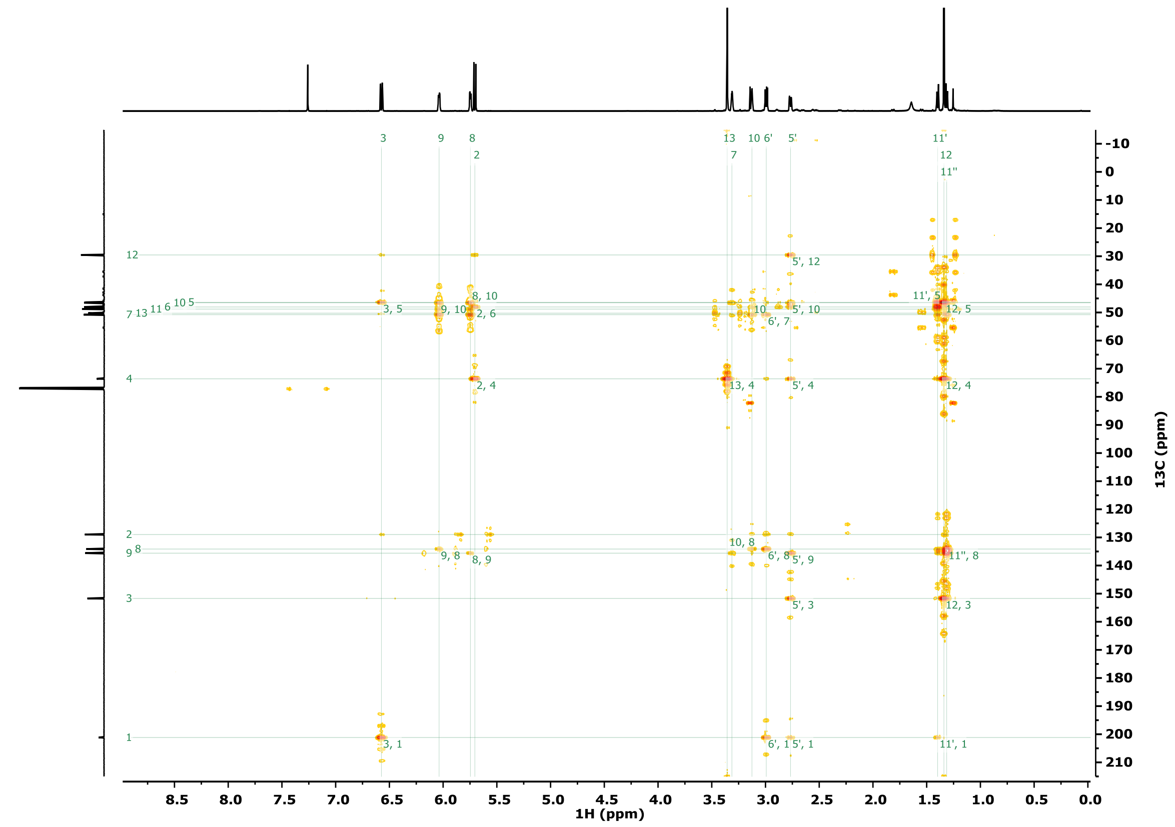

NOESY Spectra 2j′

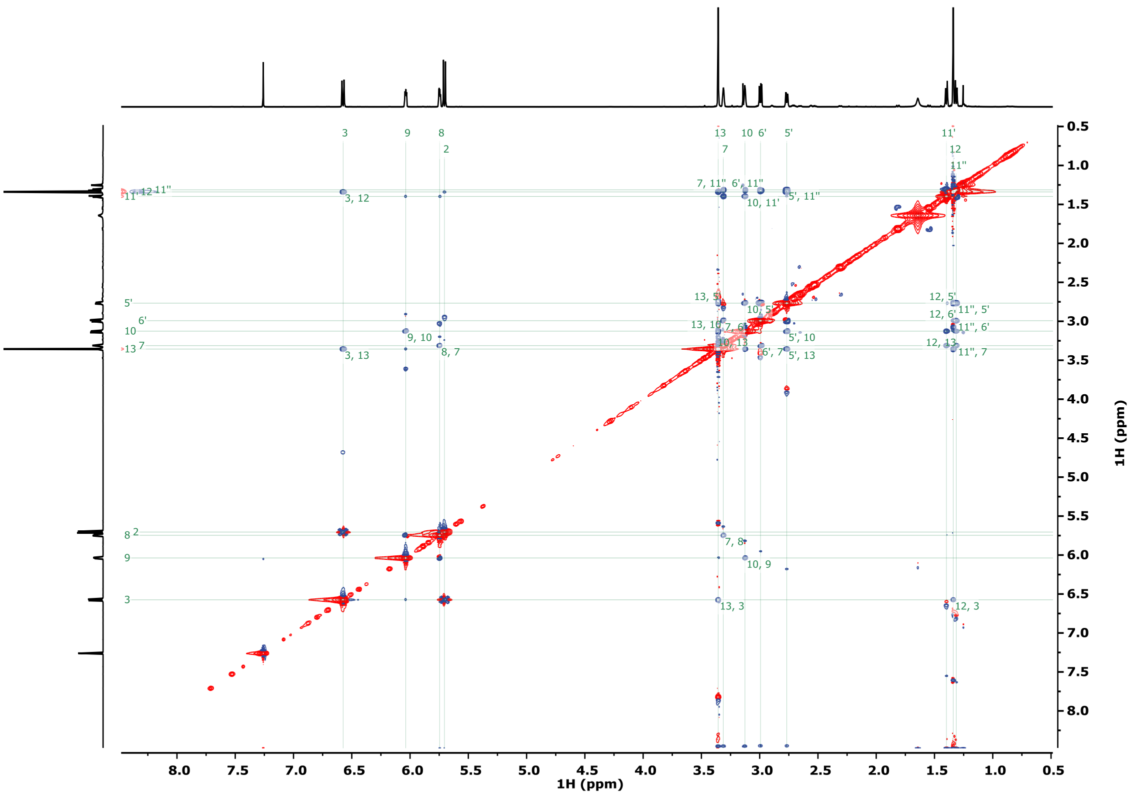

^{1}H NMR and ^{13}C NMR Spectra 2k′

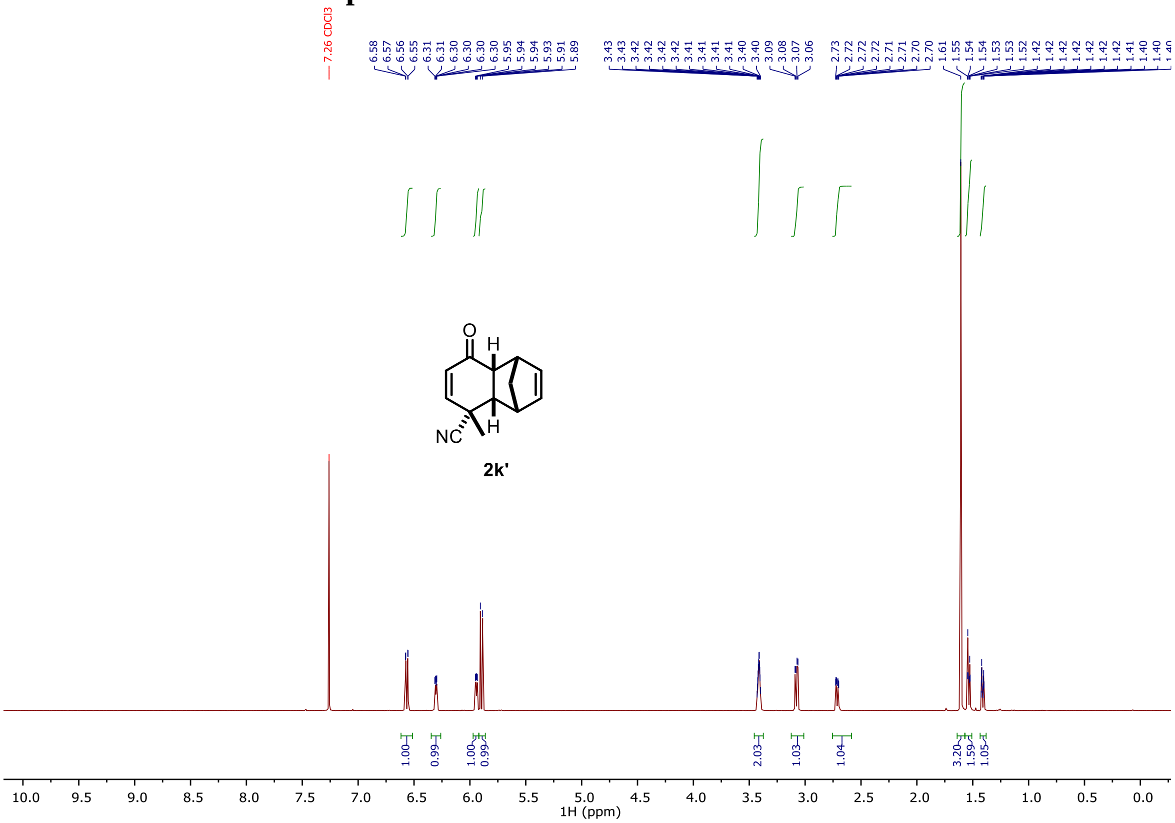

2k'

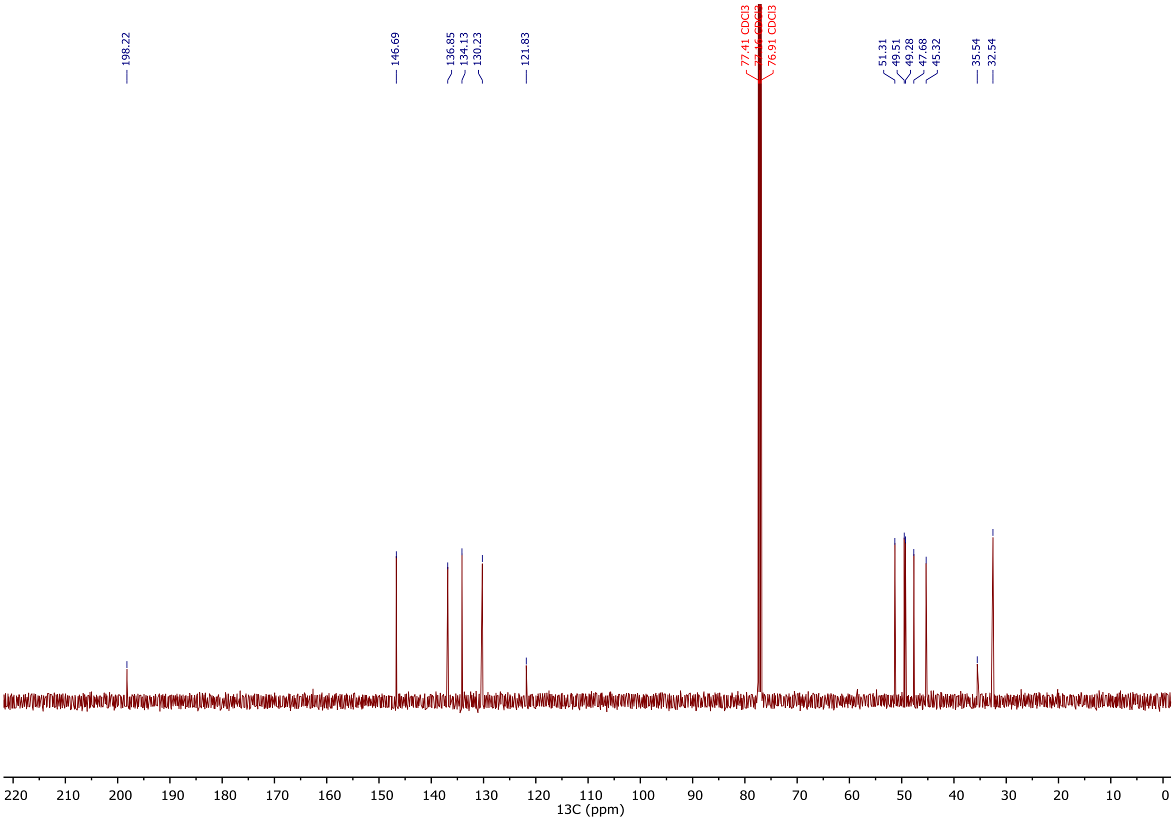

^{1}H NMR and ^{13}C NMR Spectra 2l′

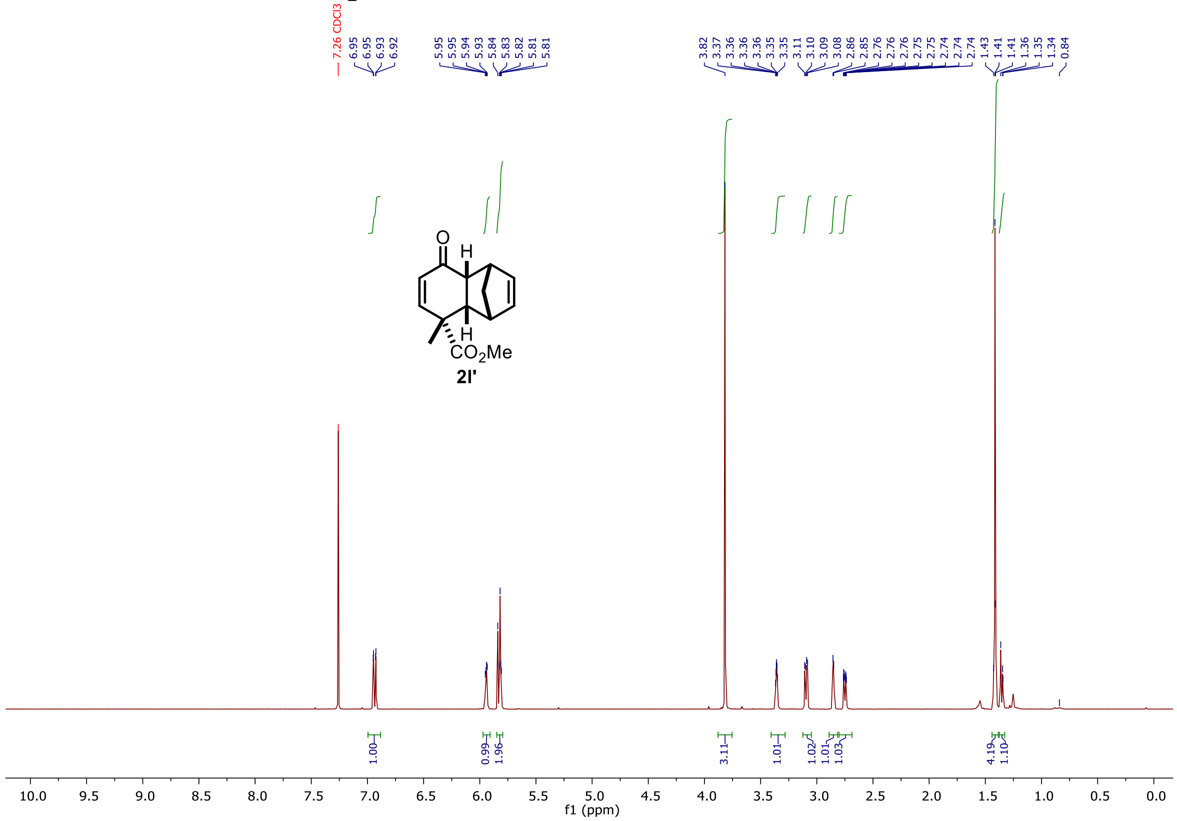

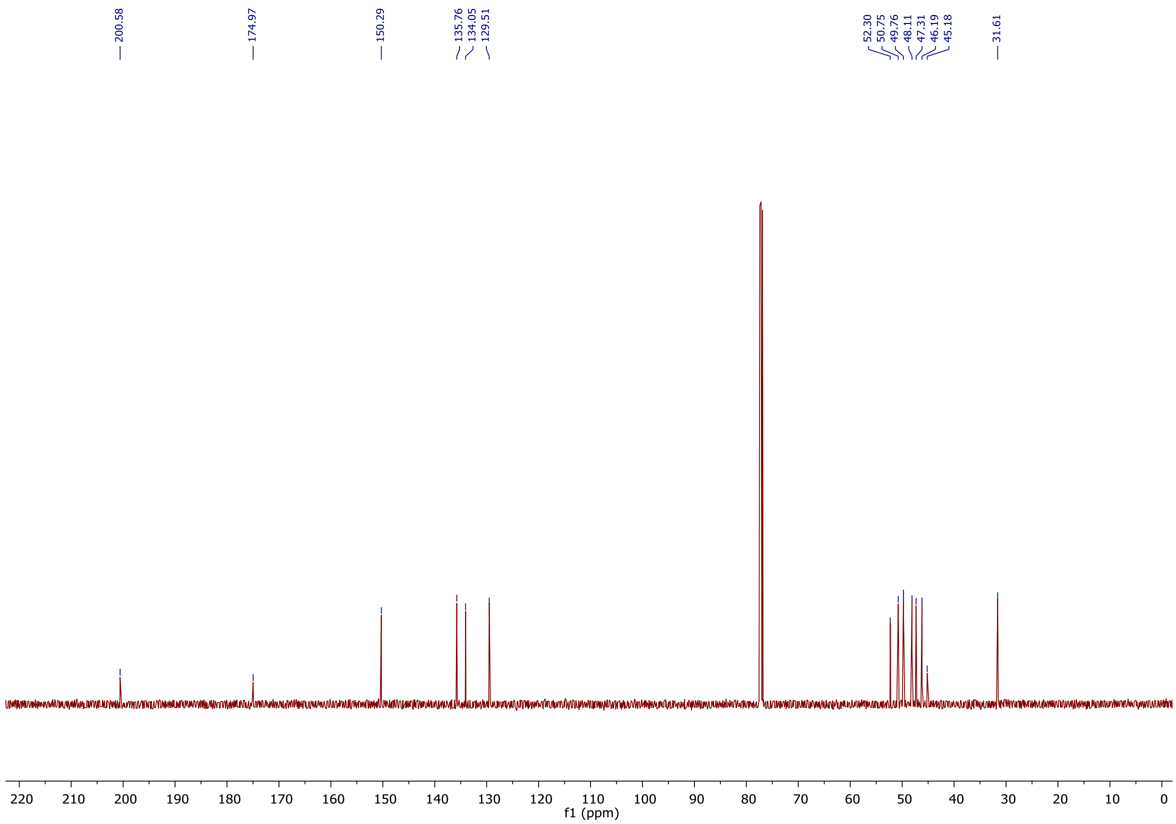

COSY spectra 2l′

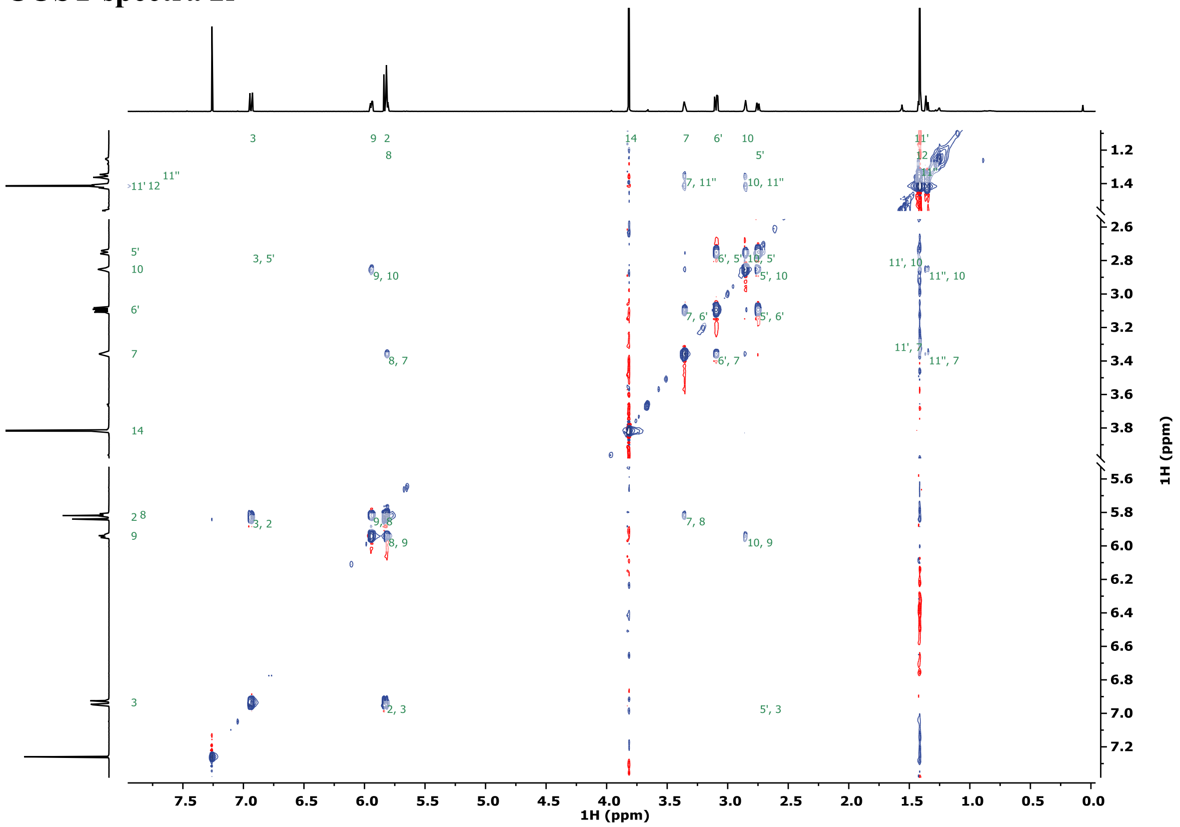

HSQC spectra 2l′

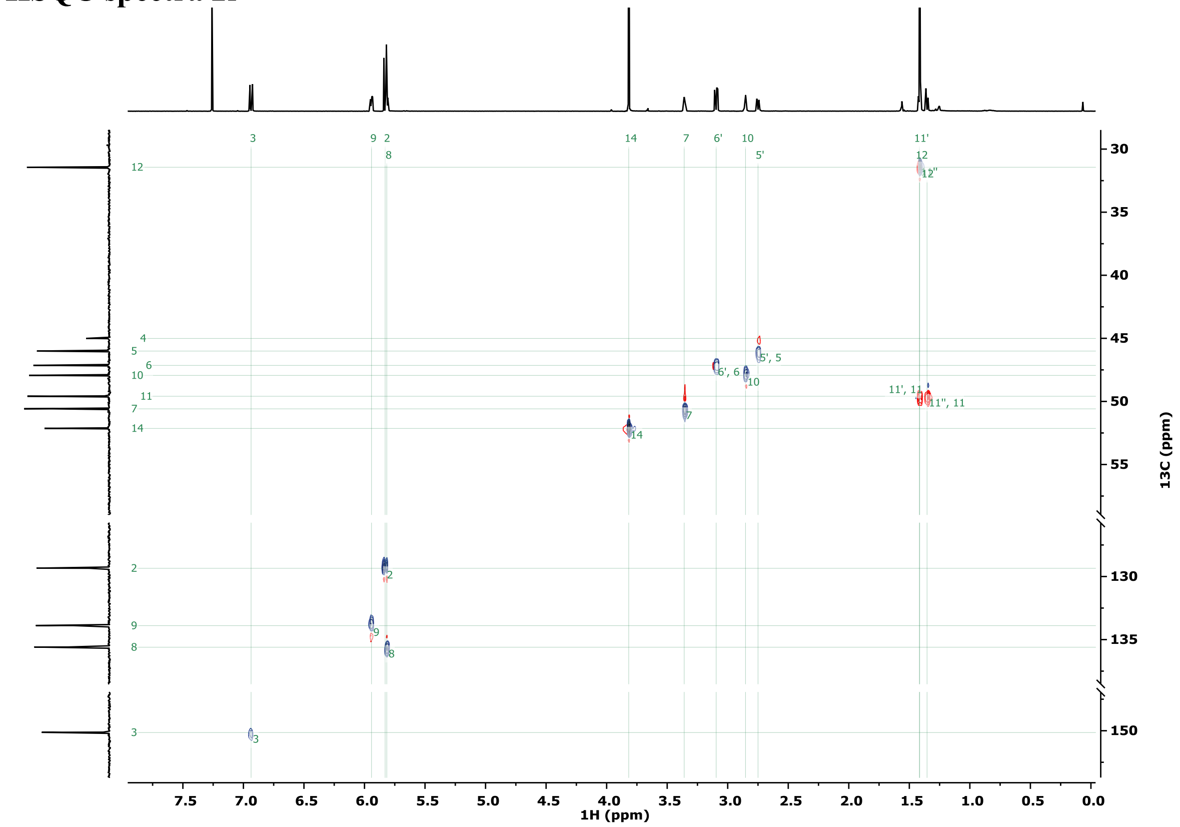

HMBC spectra 2l′

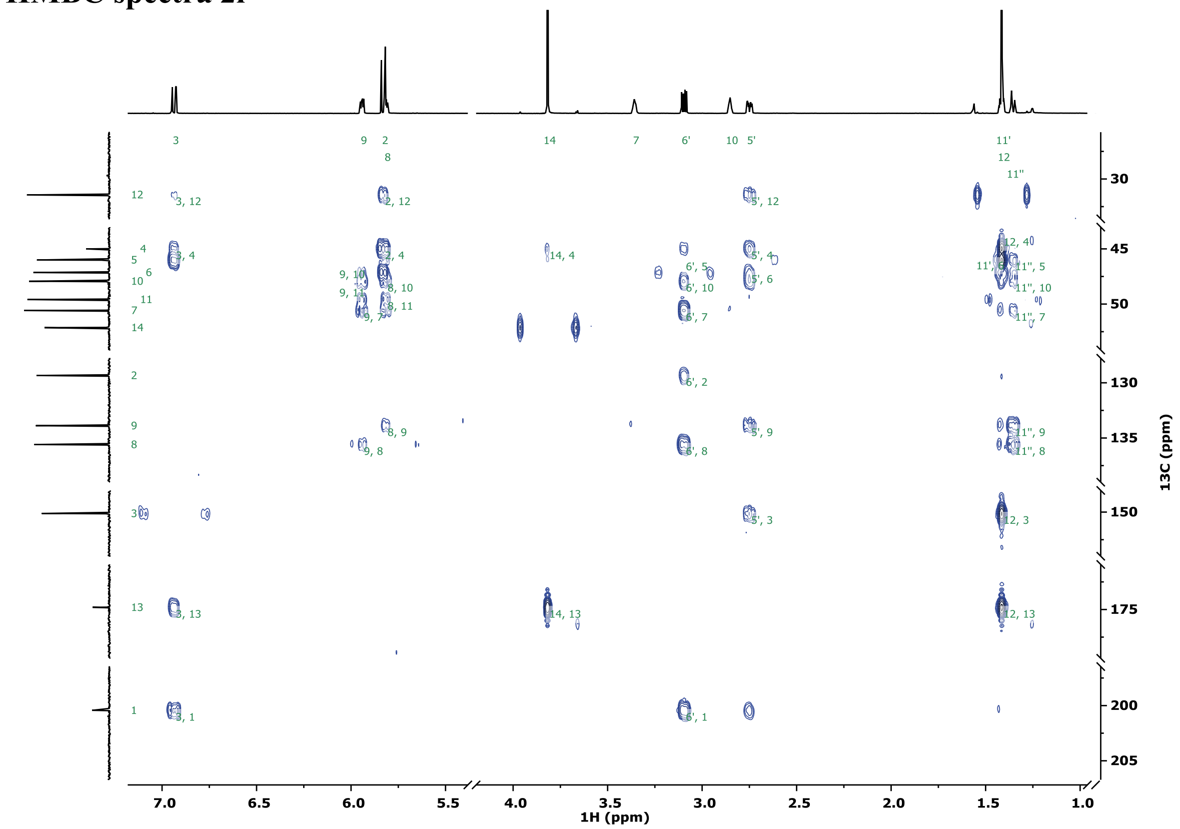

NOESY spectra 2l′

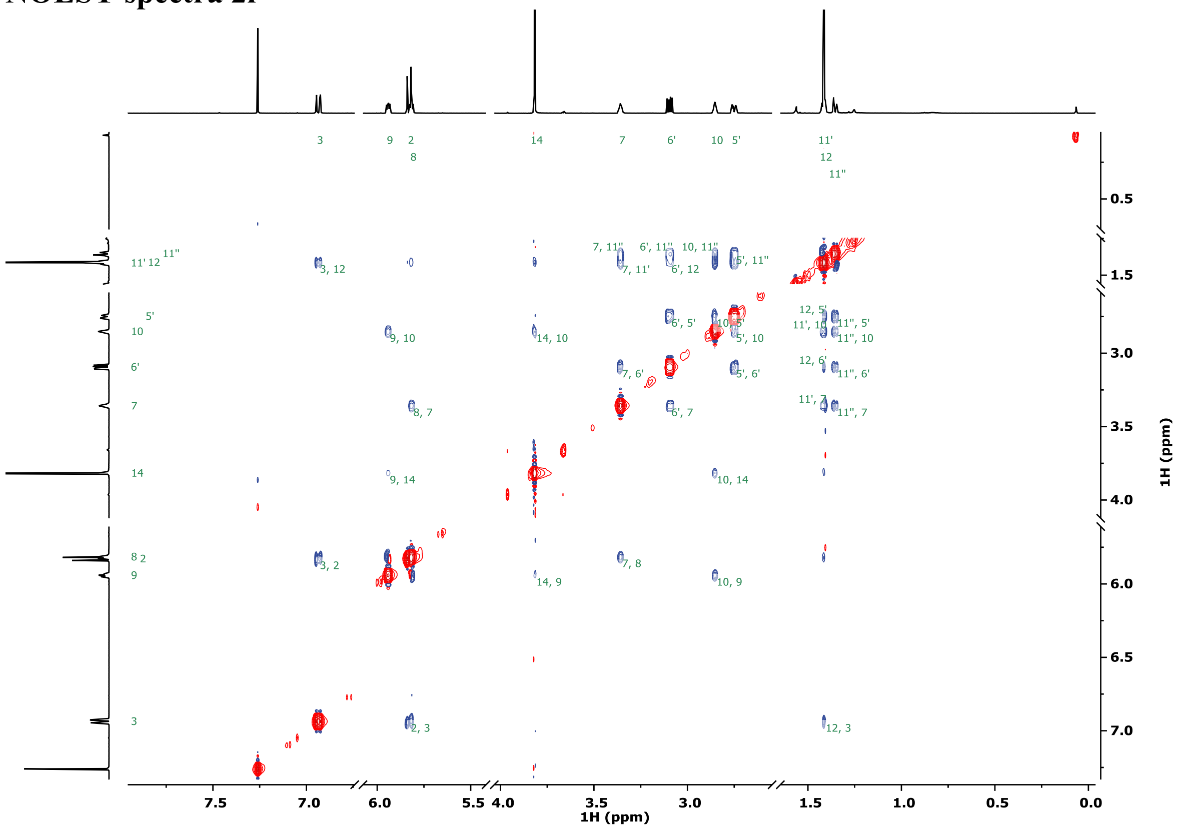

^{1}H NMR and ^{13}C NMR Spectra 2jj and 2jj′

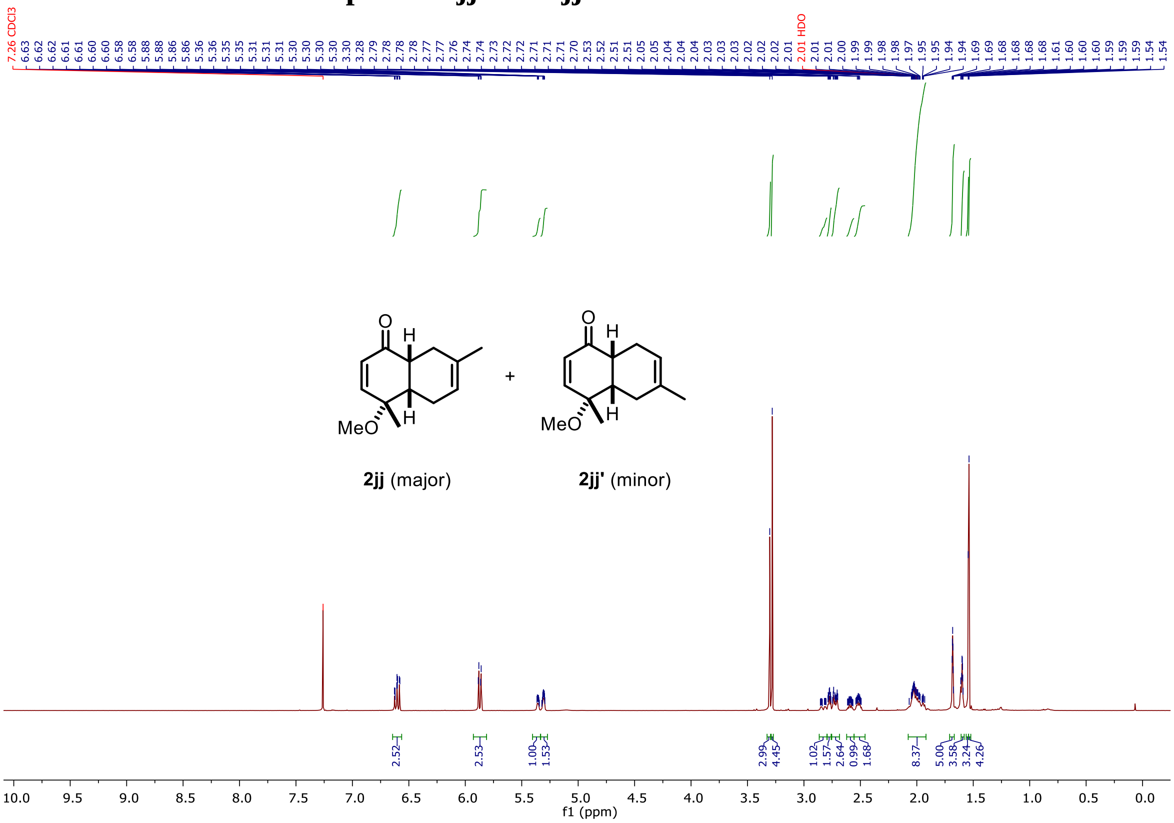

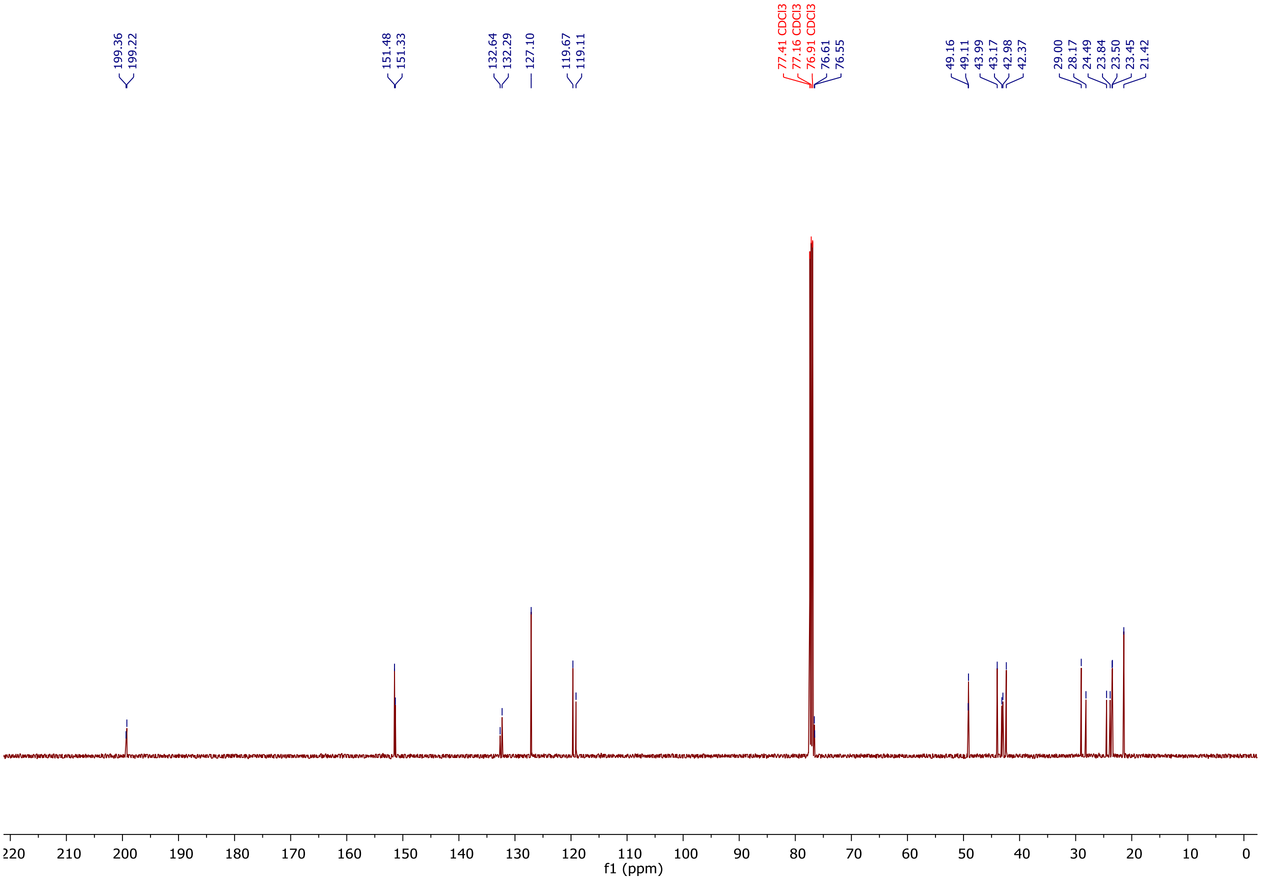

NOESY Spectrum

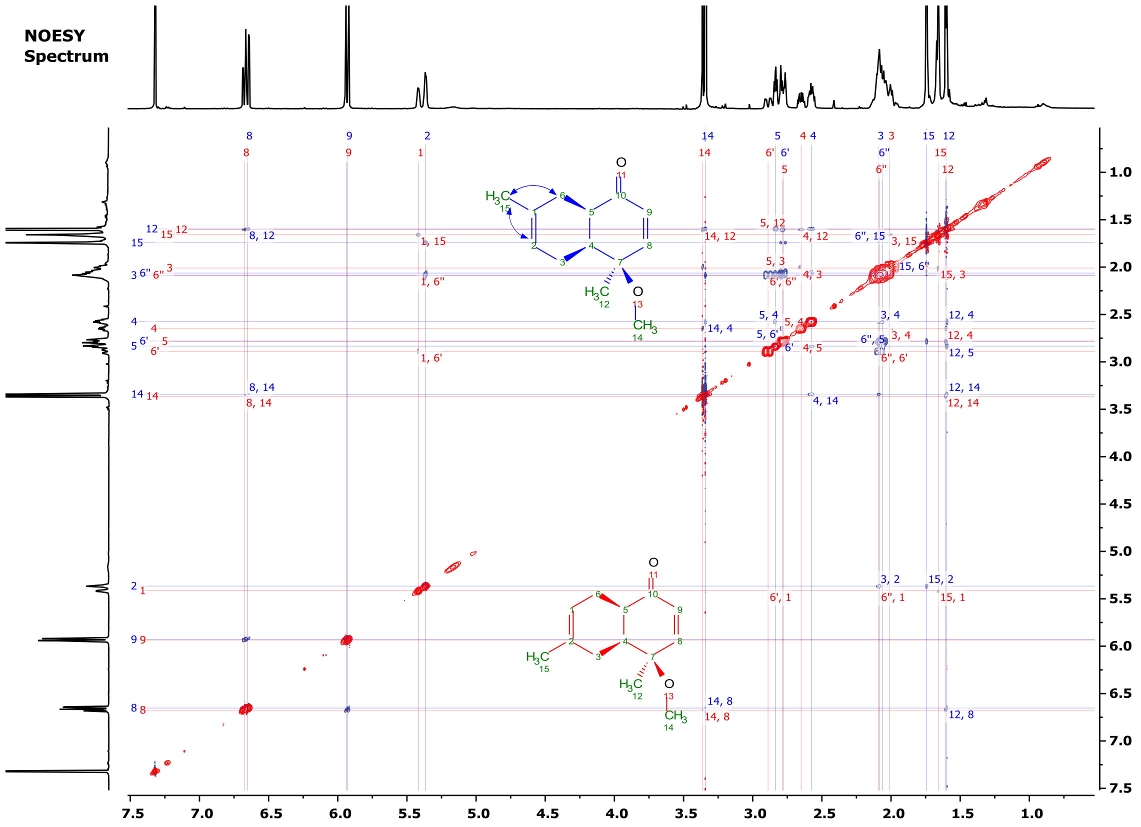

HSQC Spectrum

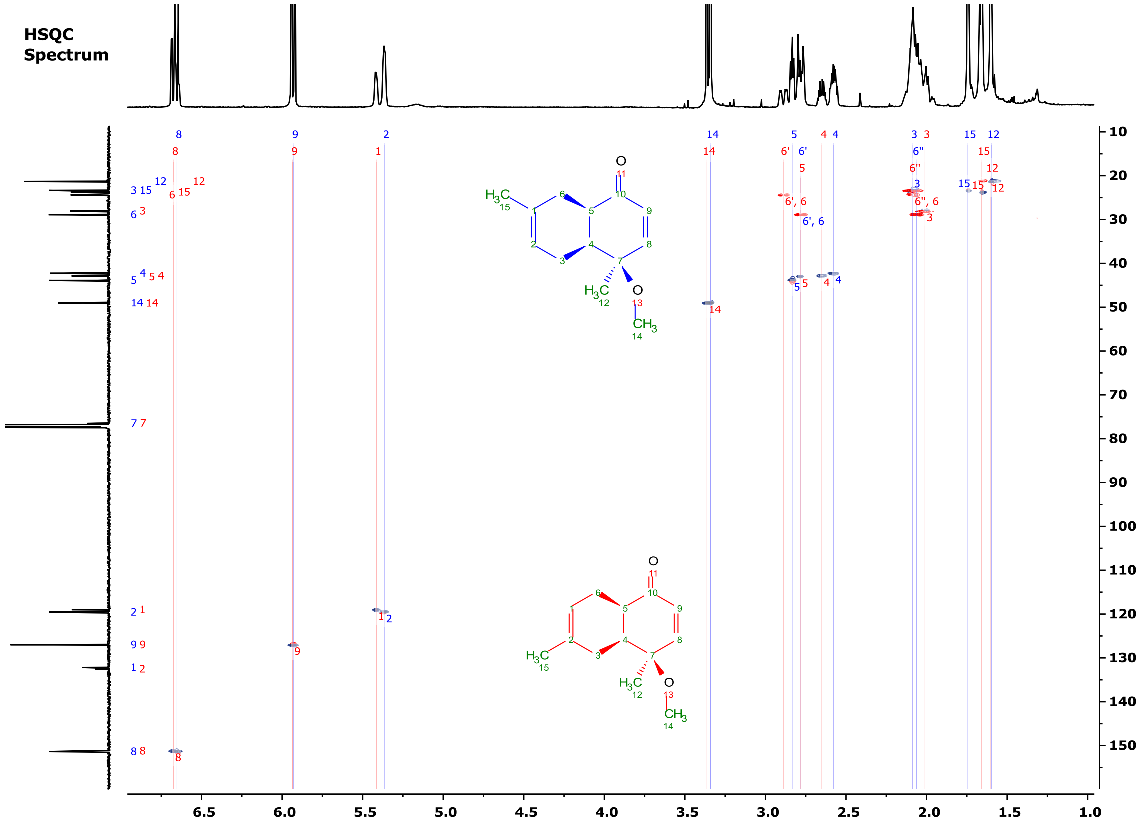

HMBC Spectrum

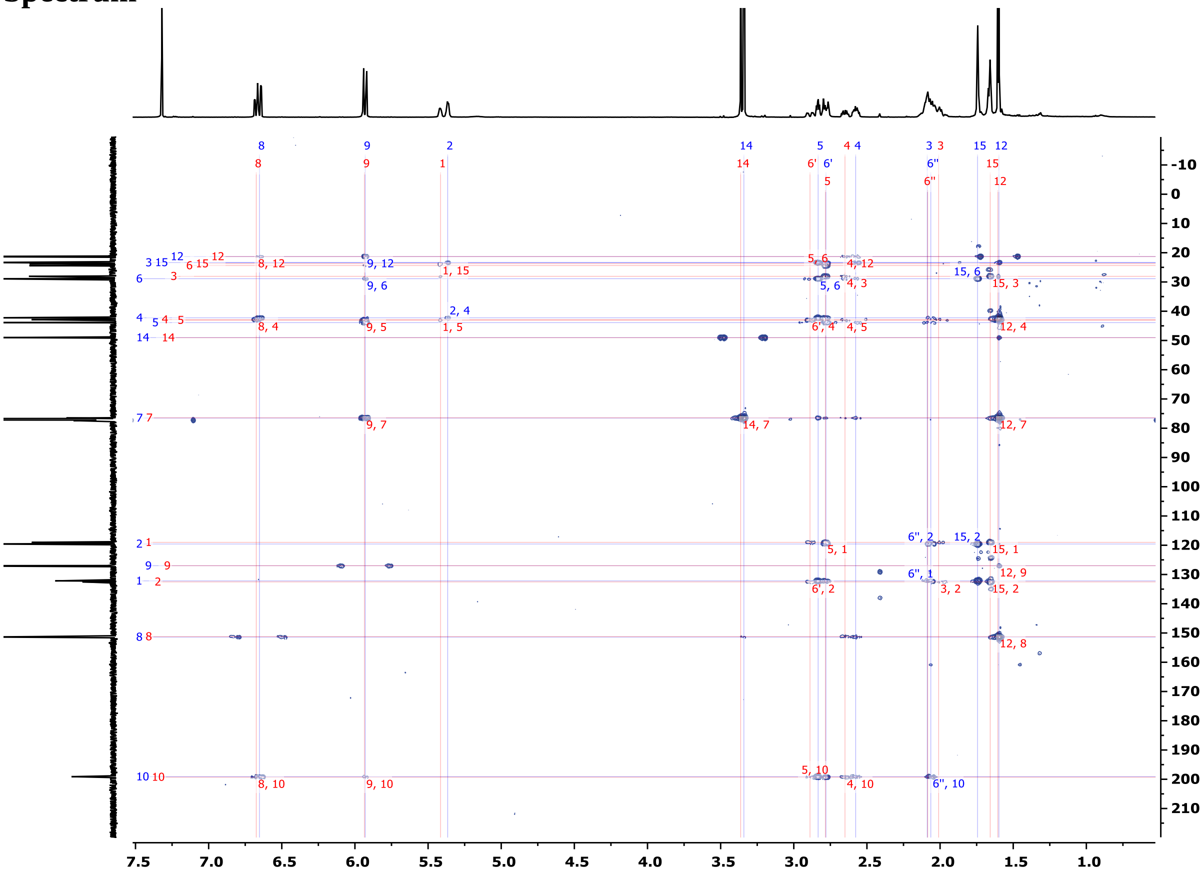

^{1}H NMR and ^{13}C NMR Spectra 7

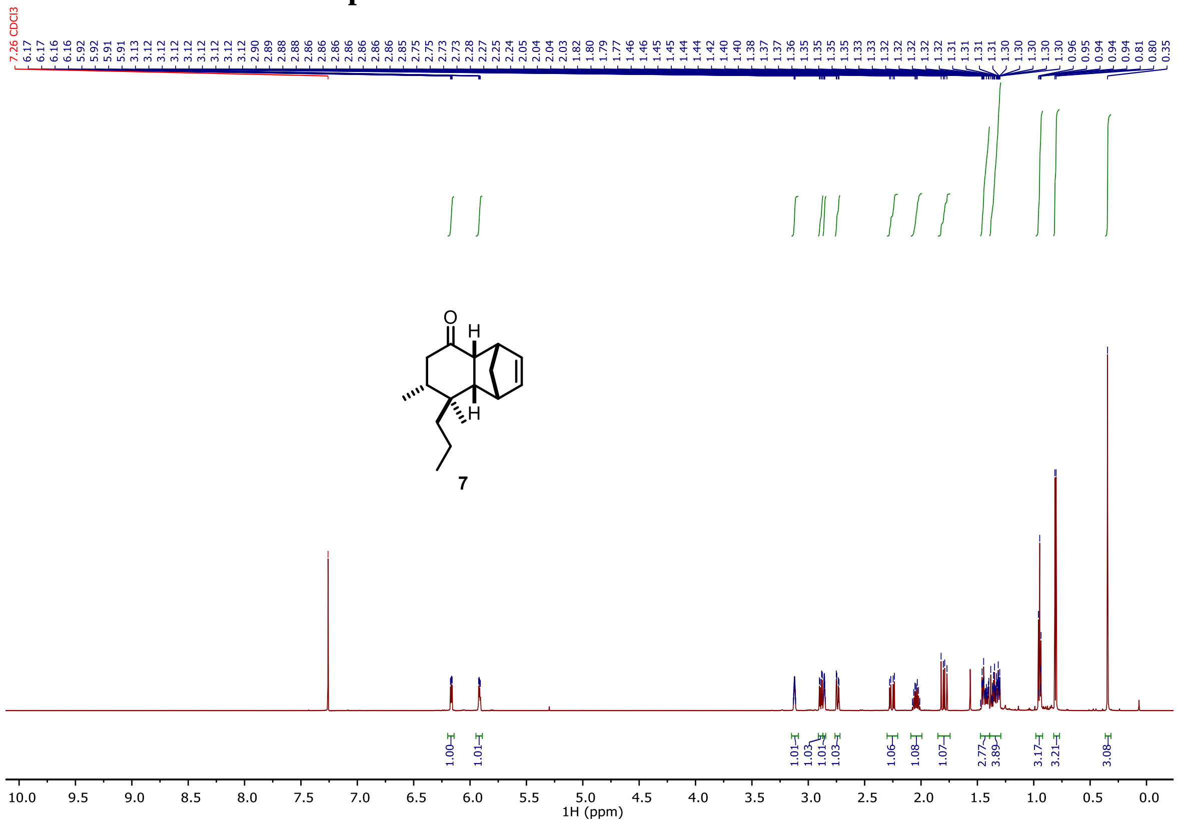

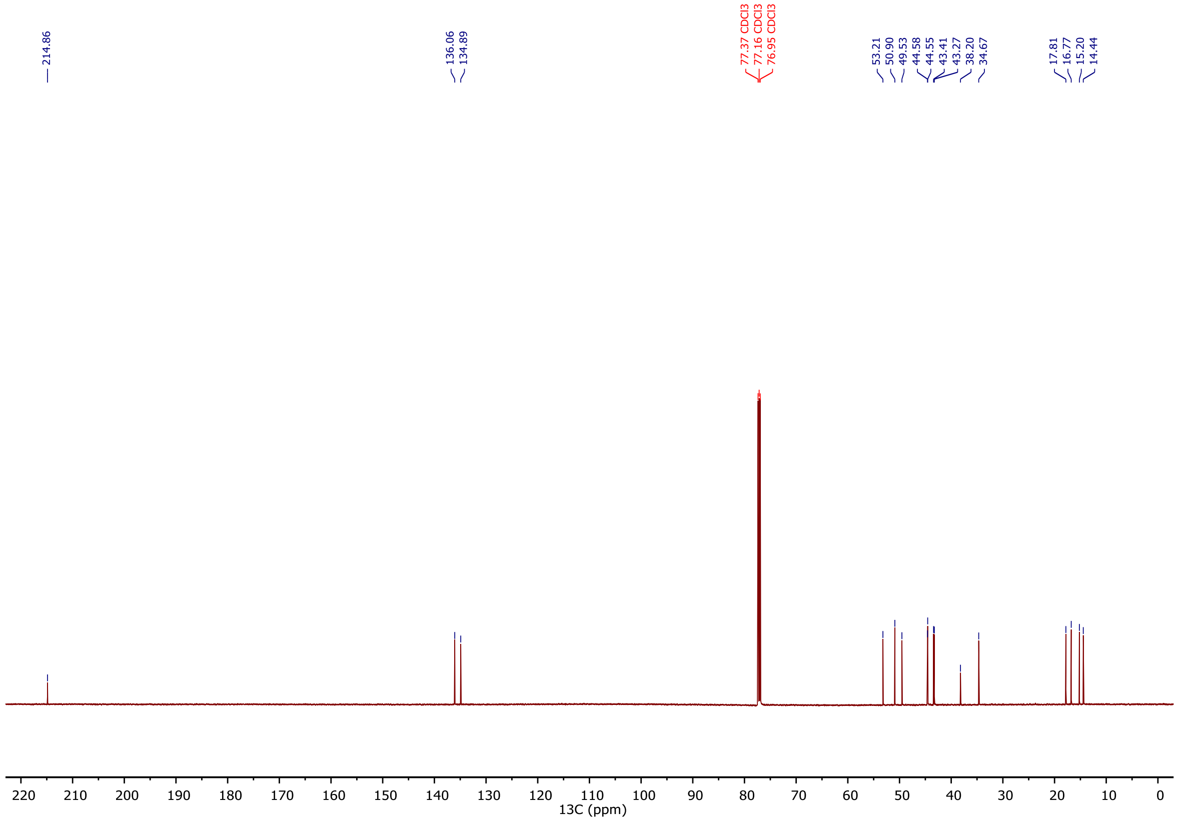

COSY spectra 7

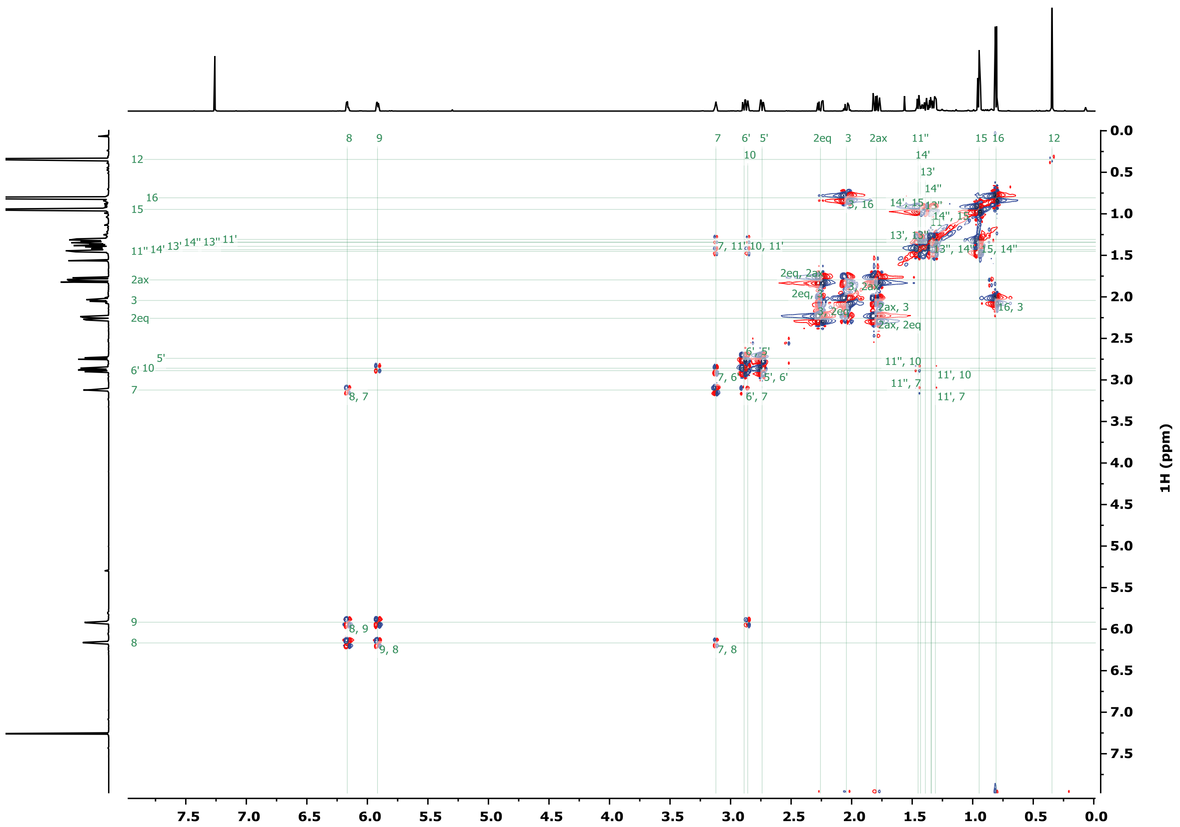

HSQC spectra 7

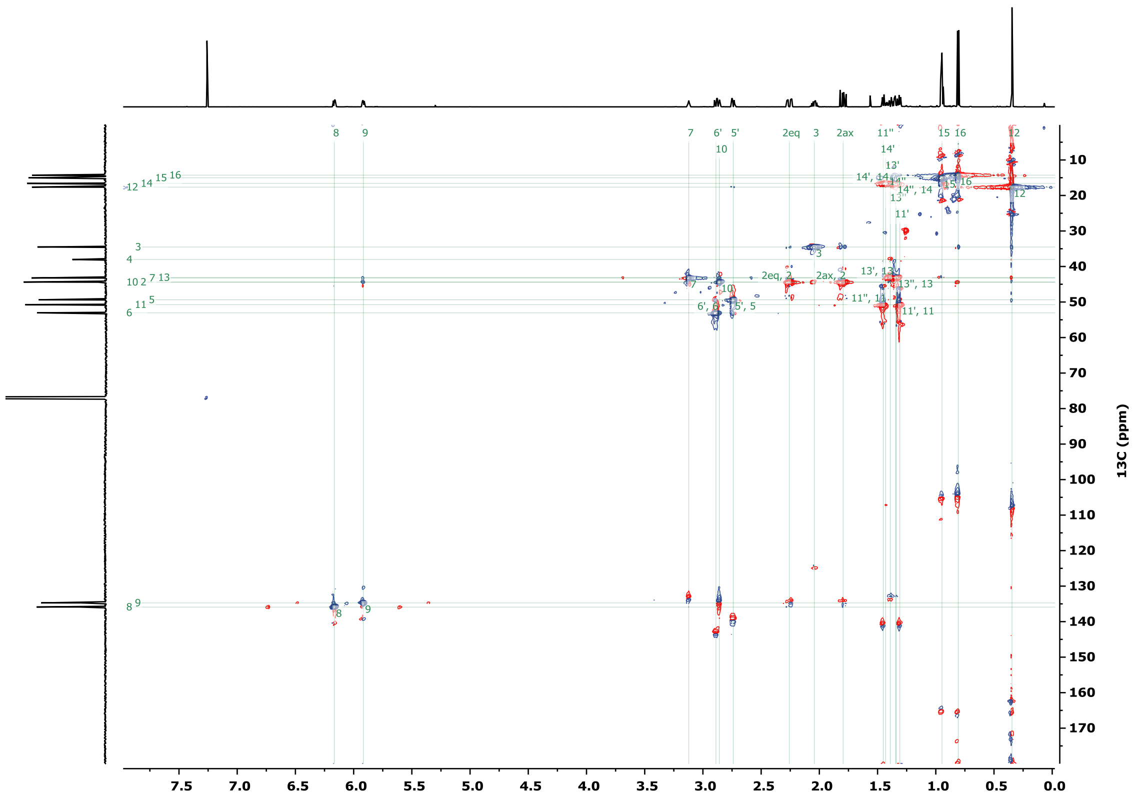

HMBC spectra 7

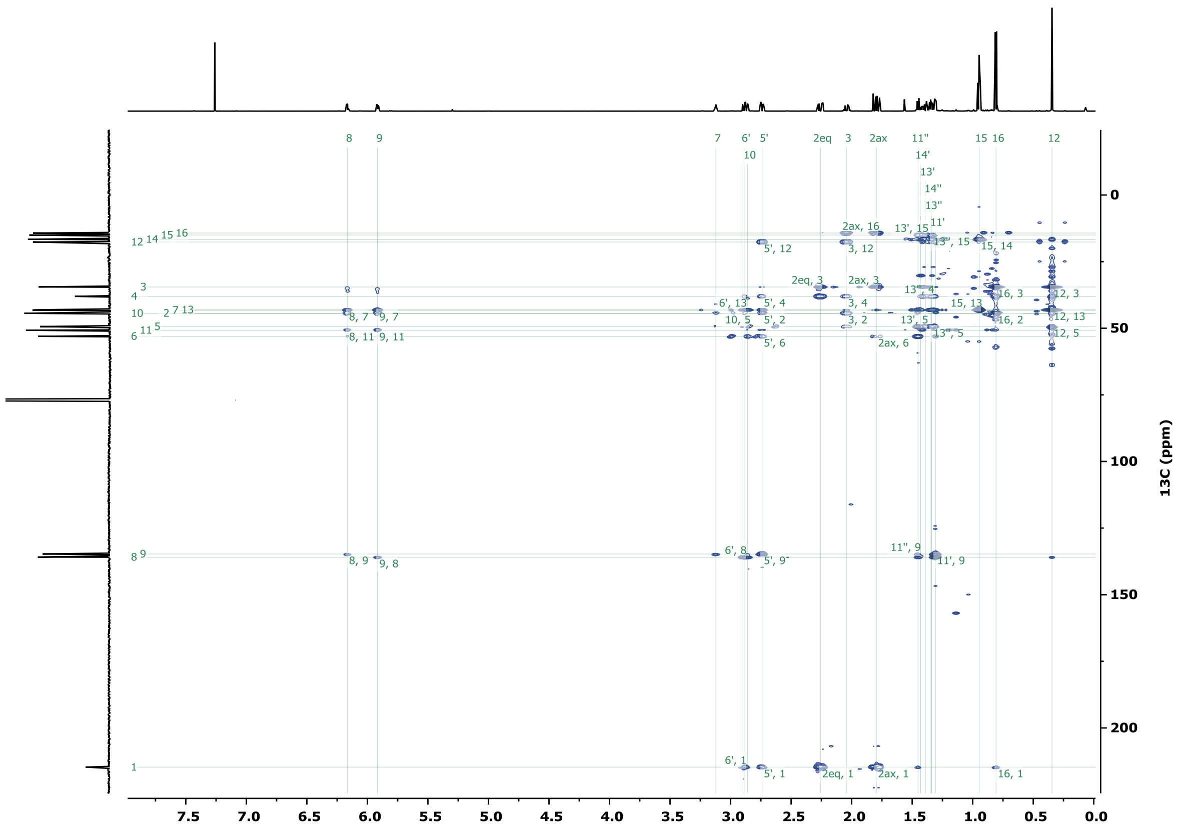

NOESY spectra 7

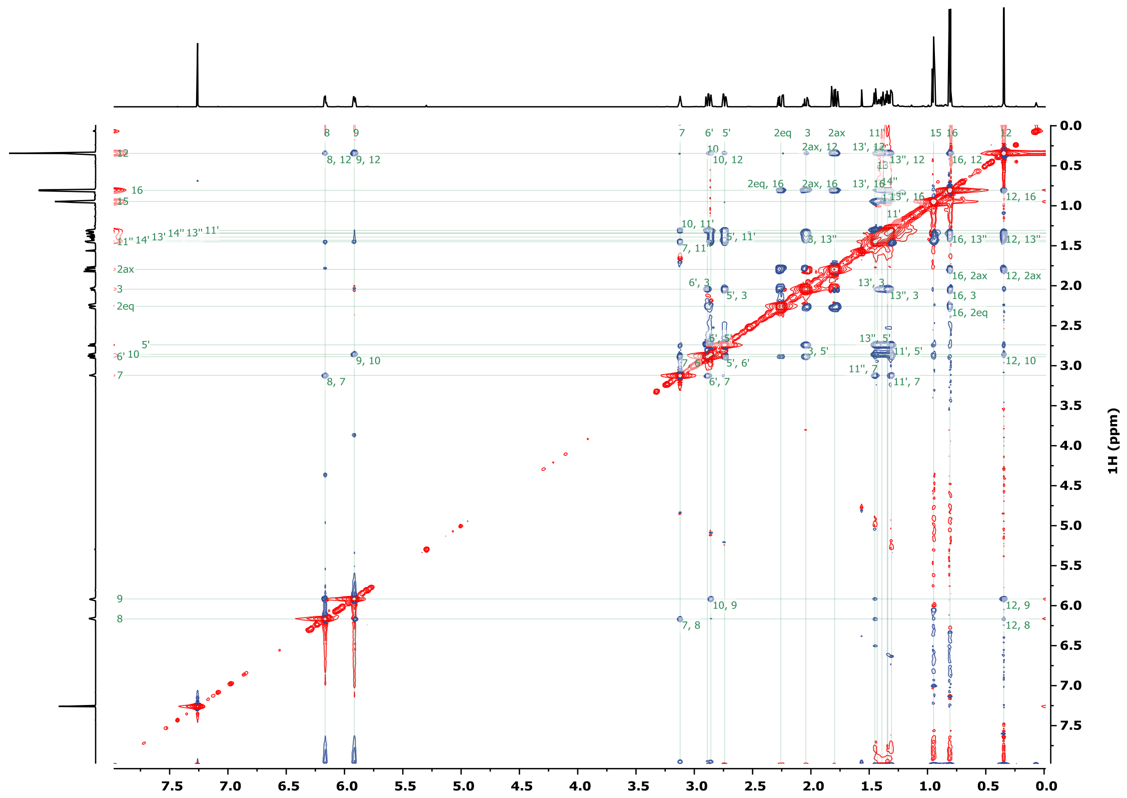

^{1}H NMR and ^{13}C NMR Spectra 8

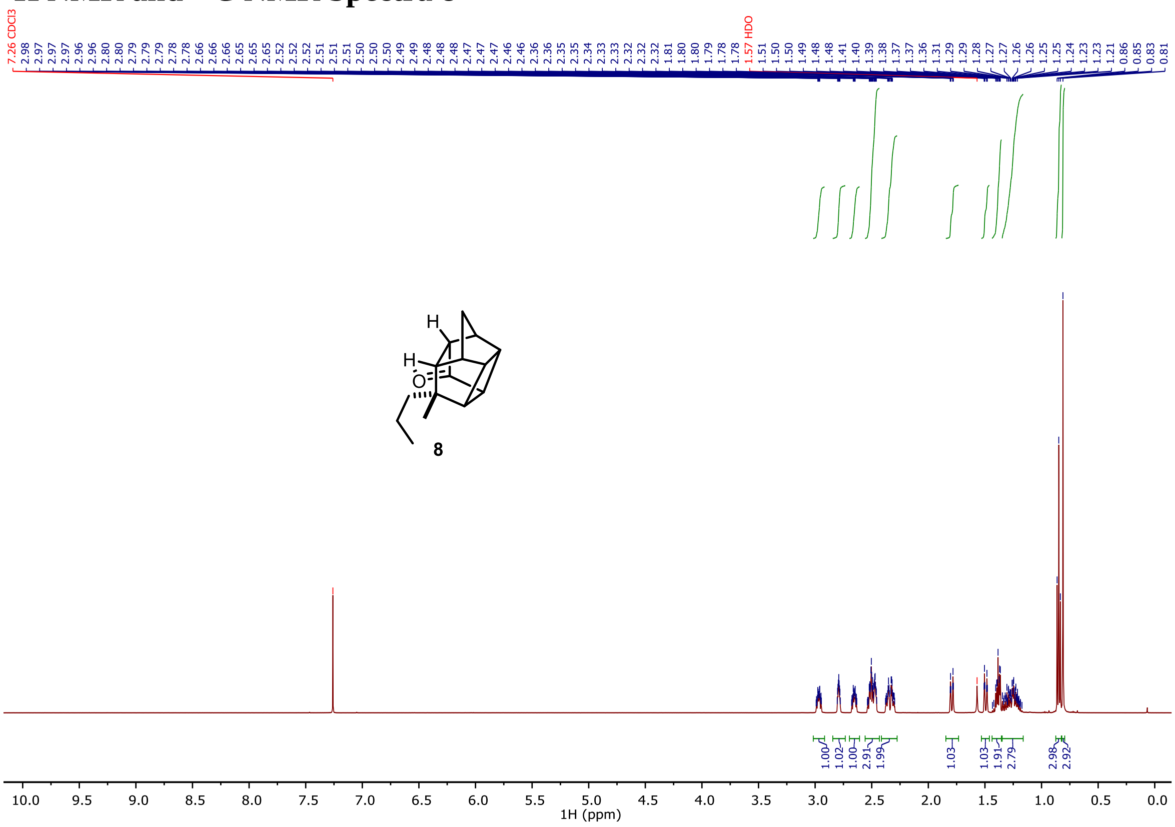

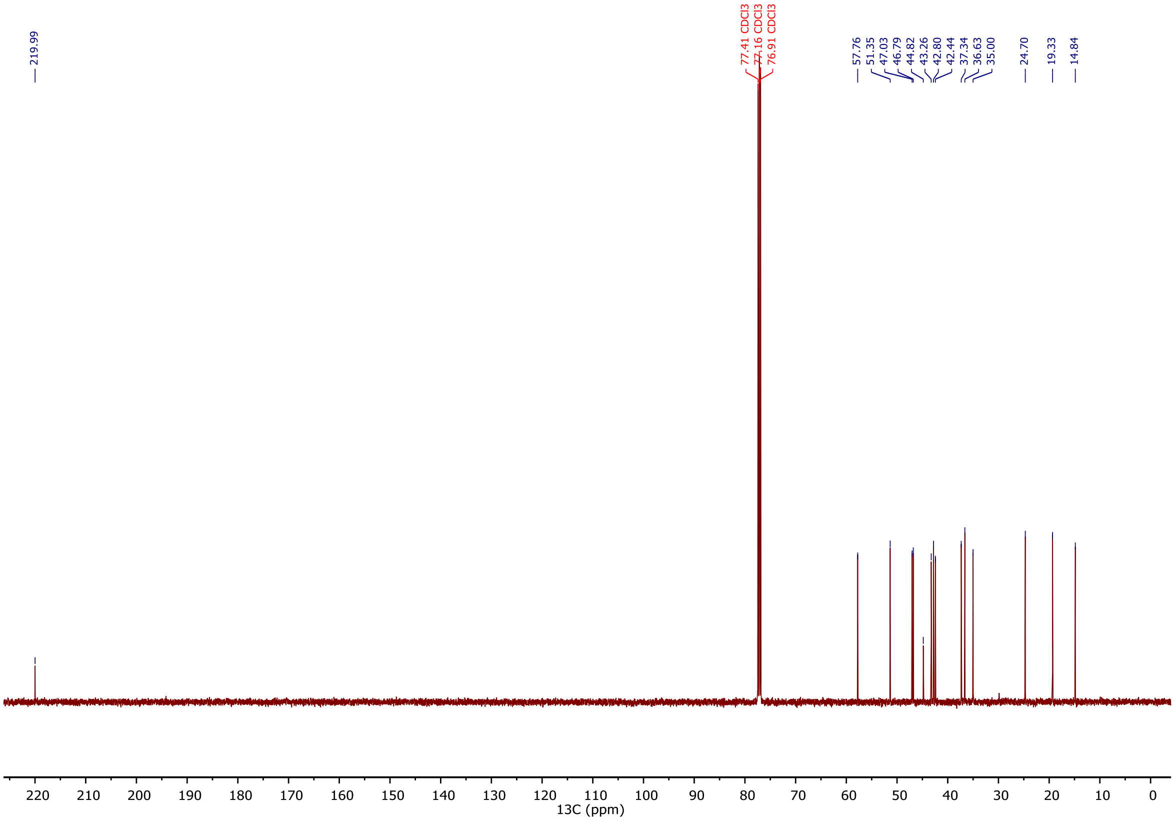

^{1}H NMR and ^{13}C NMR Spectra 16

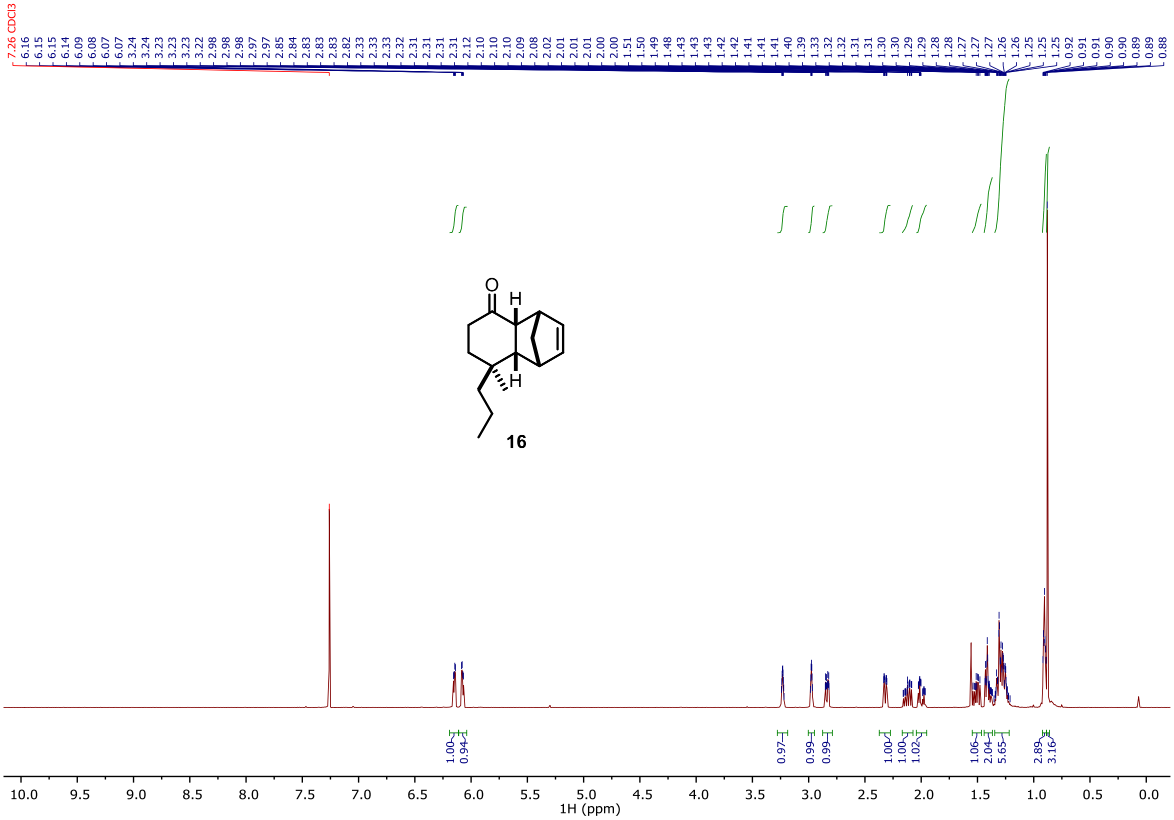

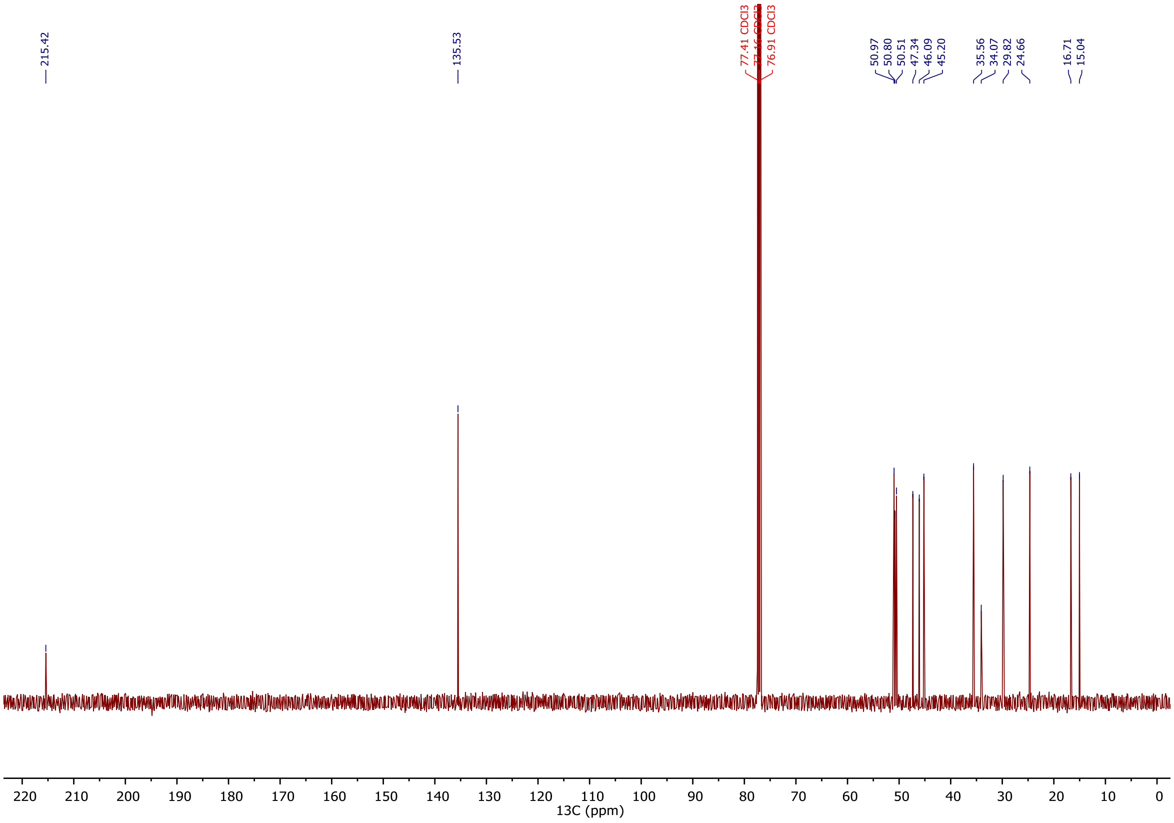

^{1}H NMR and ^{13}C NMR Spectra 9b

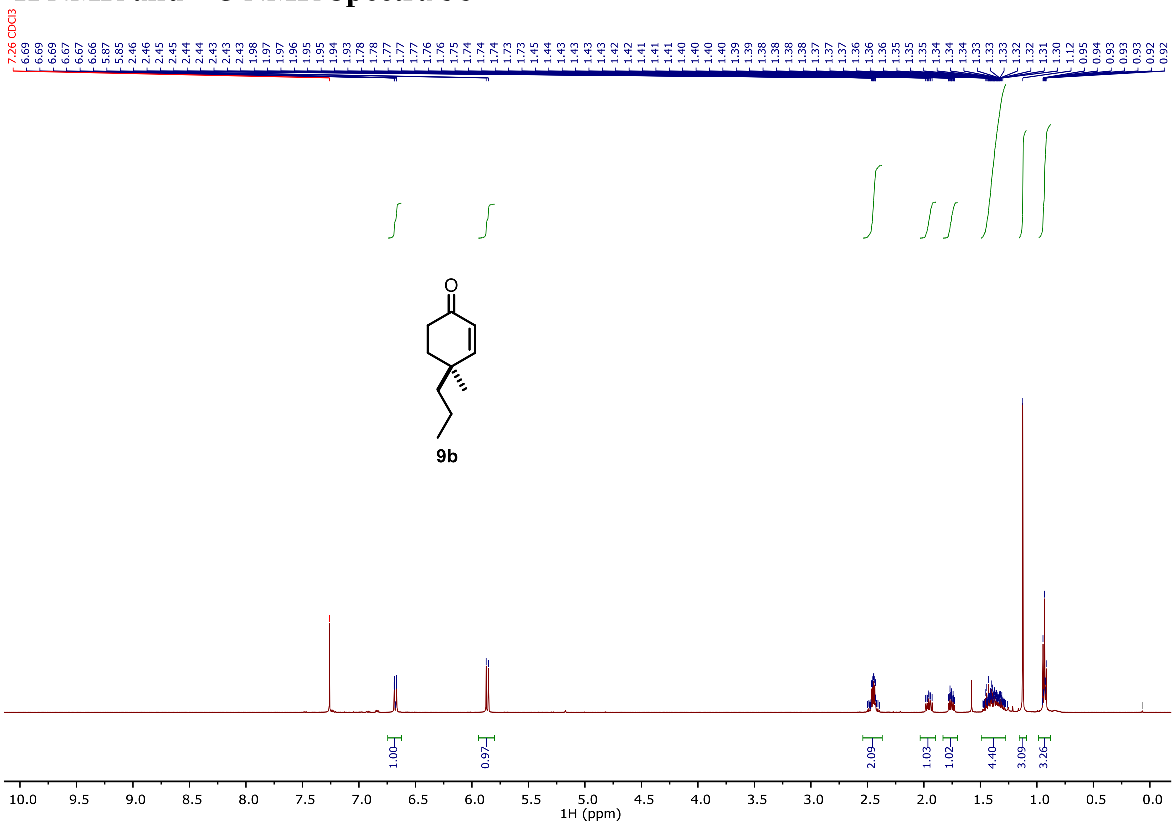

9b

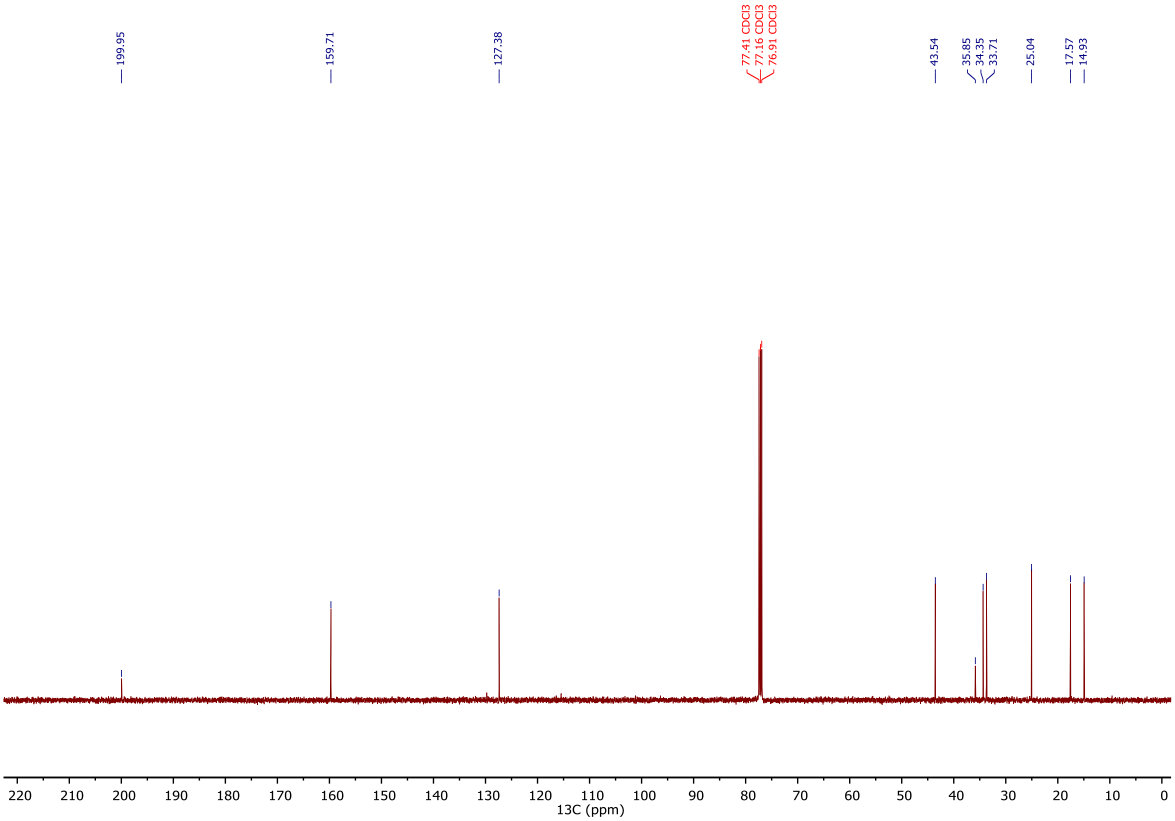

^{1}H NMR and ^{13}C NMR Spectra 10

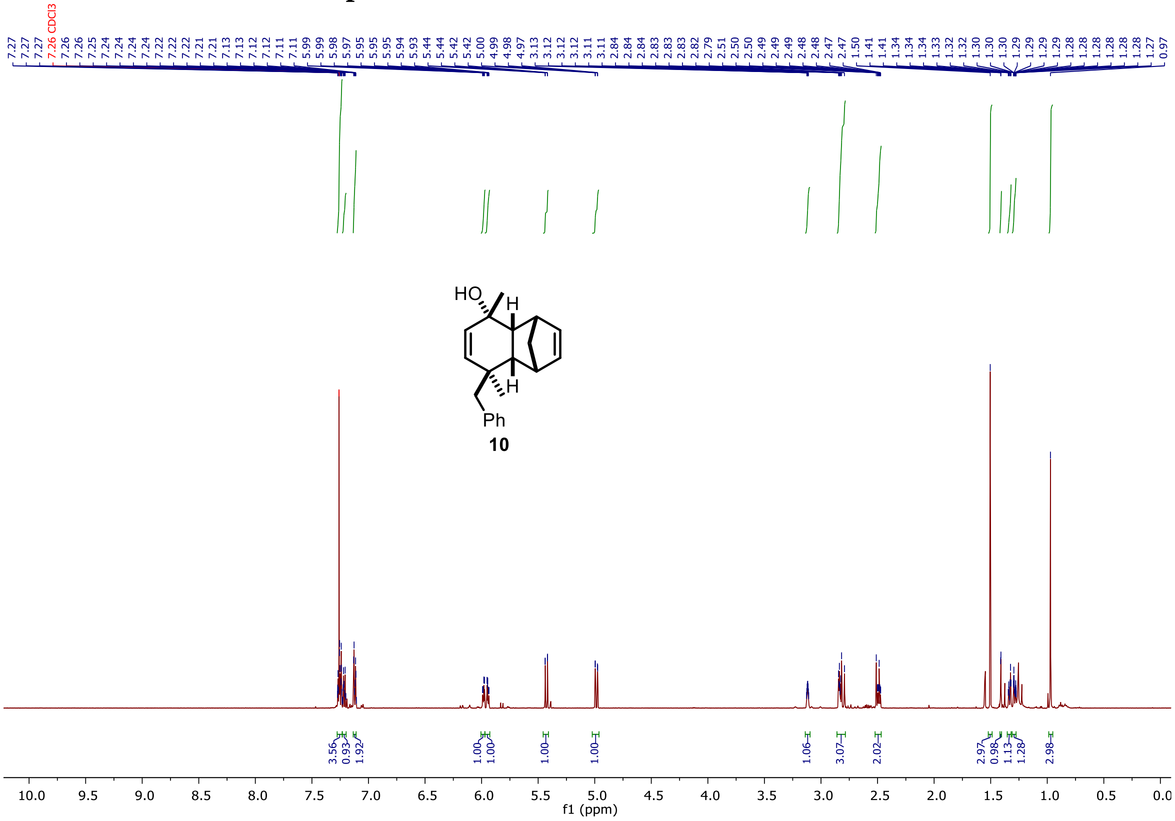

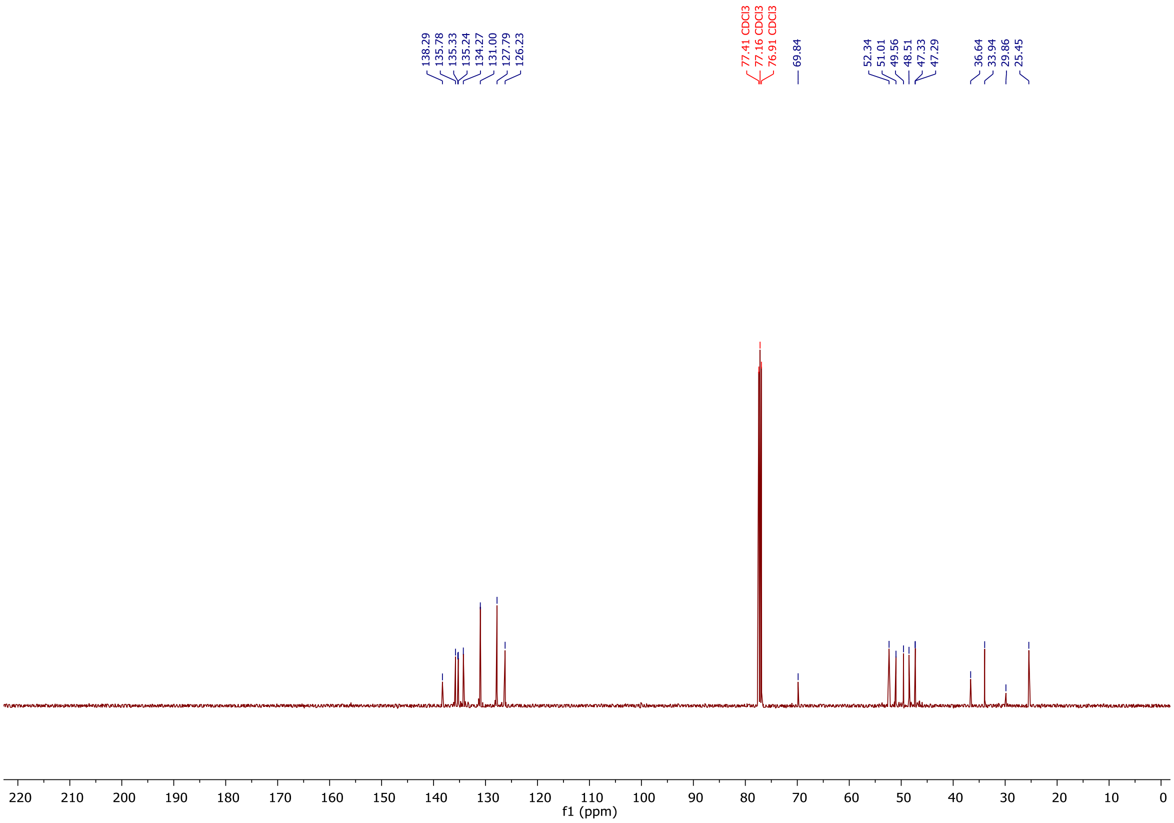

COSY spectra 10

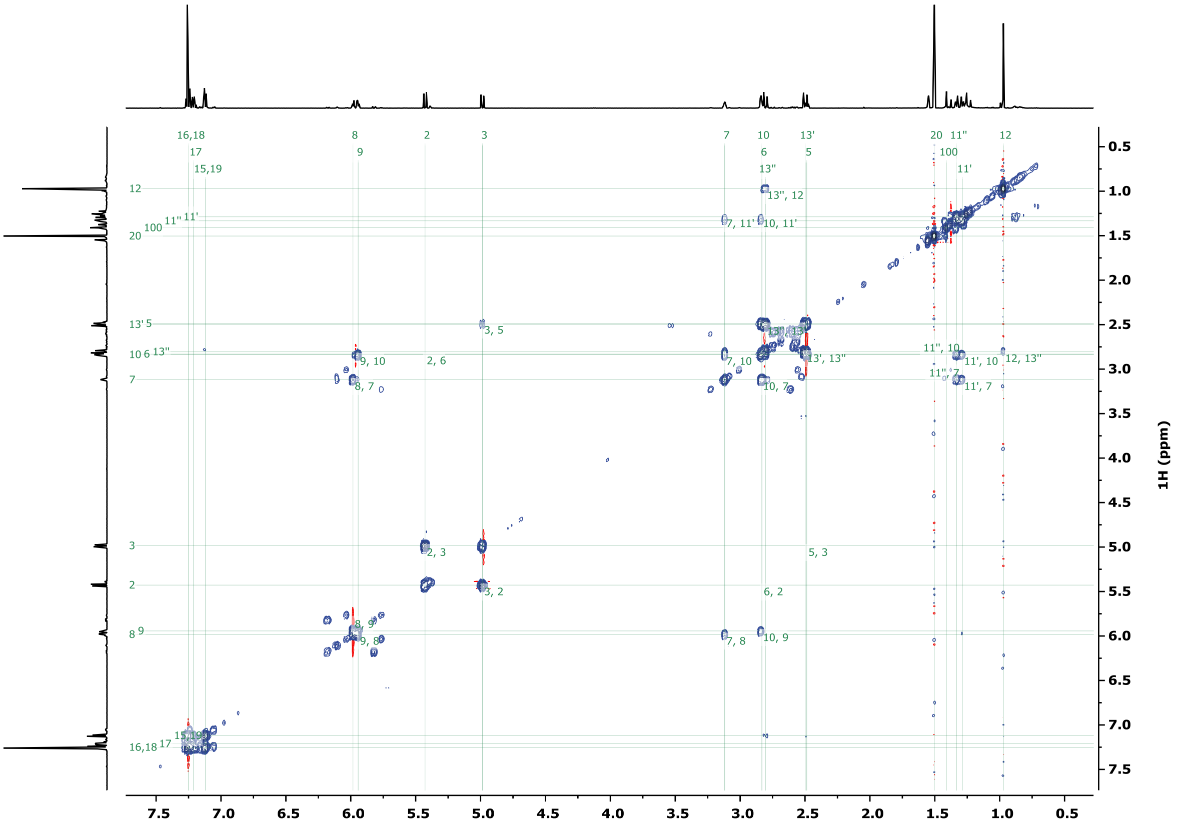

HSQC spectra 10

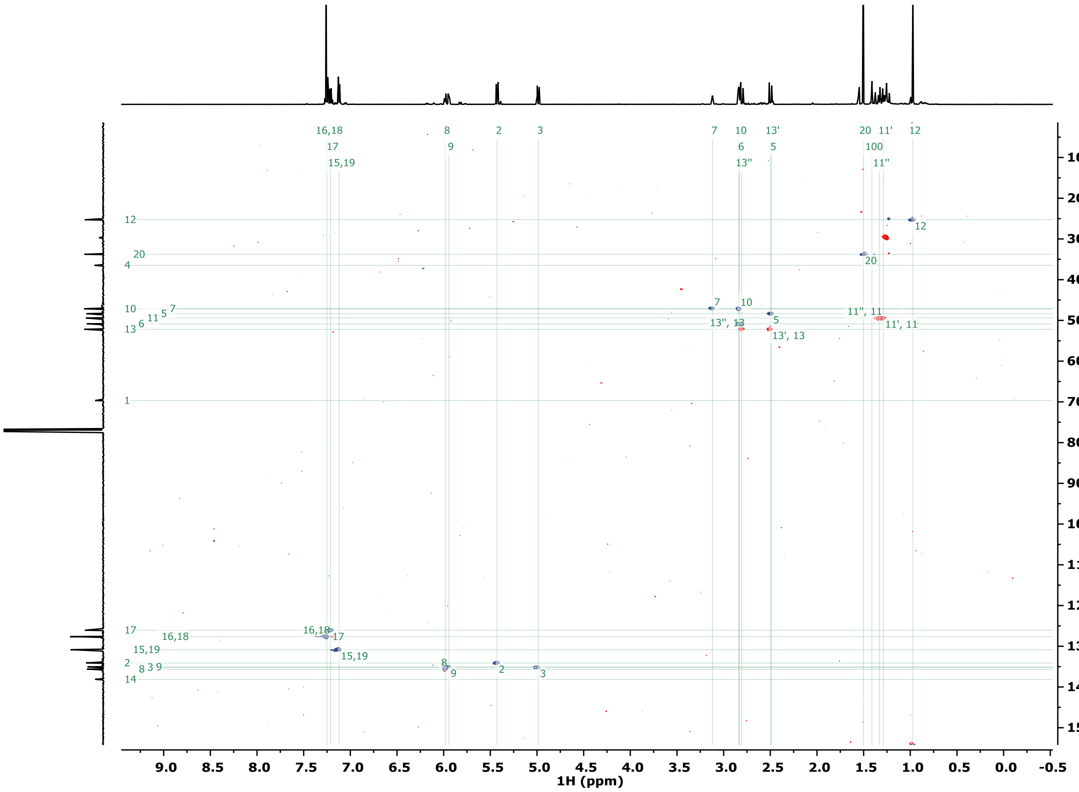

HMBC spectra 10

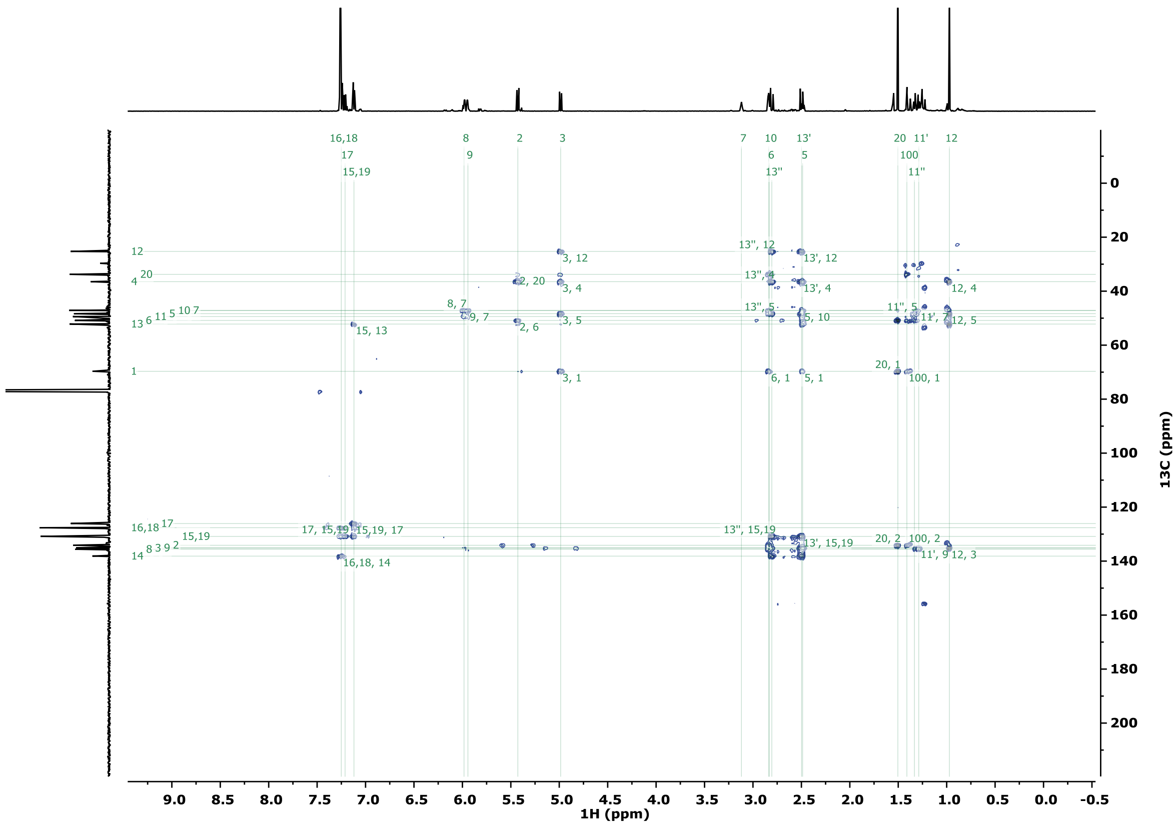

NOESY spectra 10

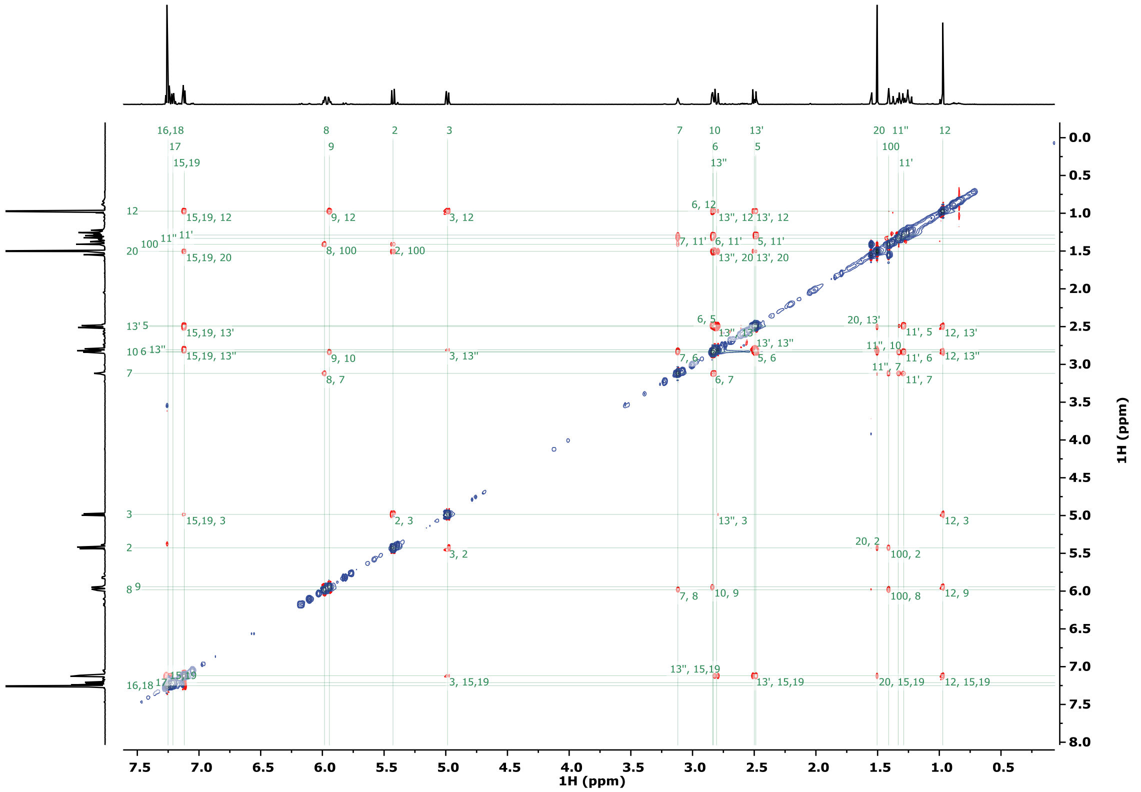

^{1}H NMR and ^{13}C NMR Spectra 11

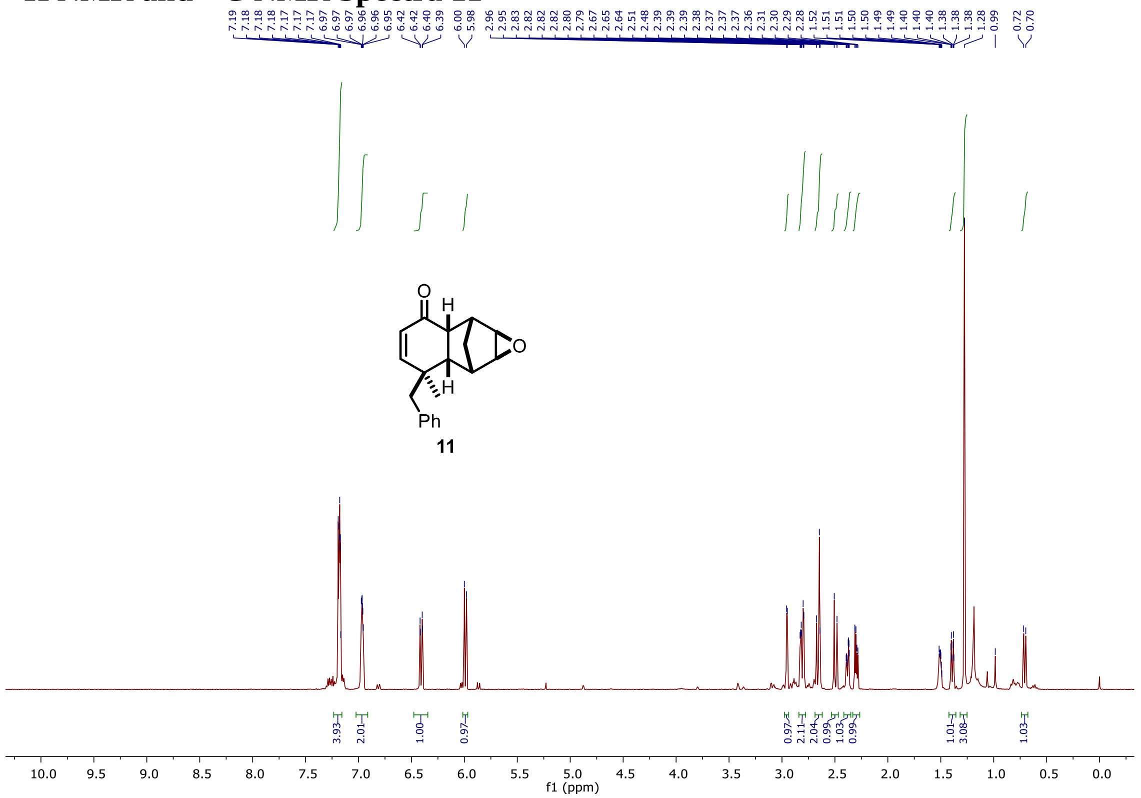

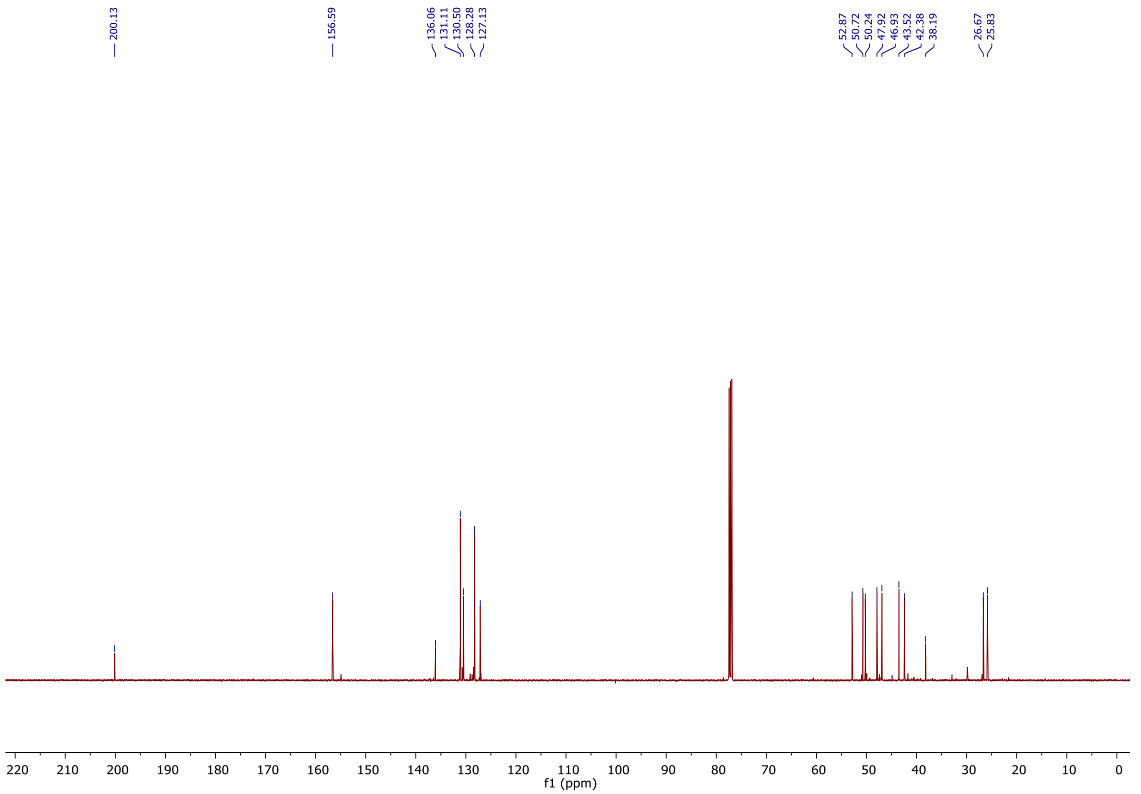

COSY spectra 11

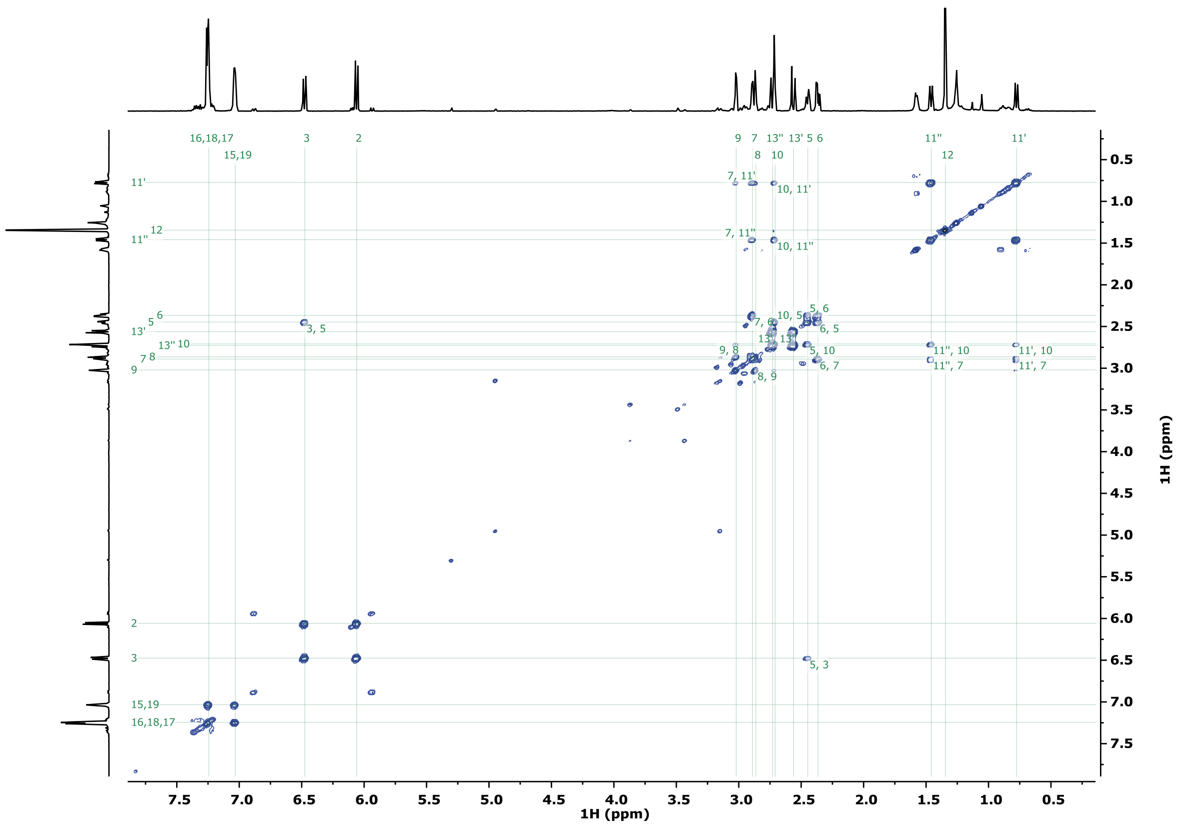

HMBC spectra 11

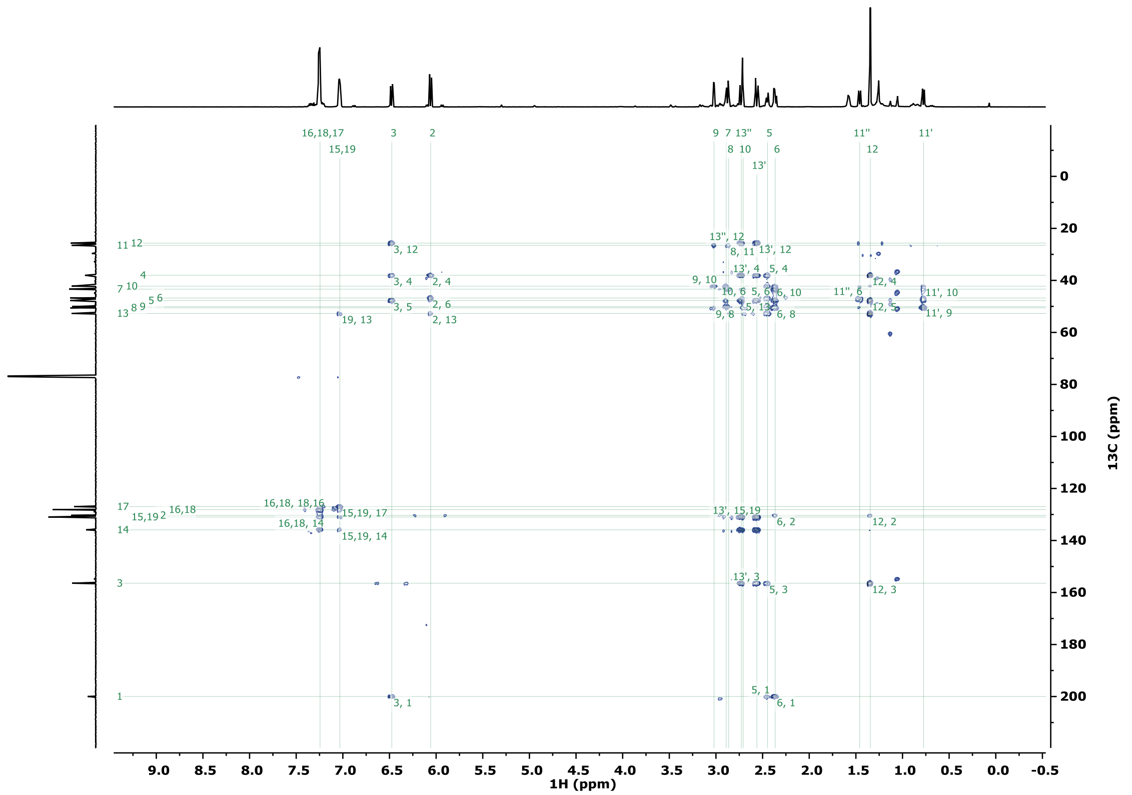

HSQC spectra 11

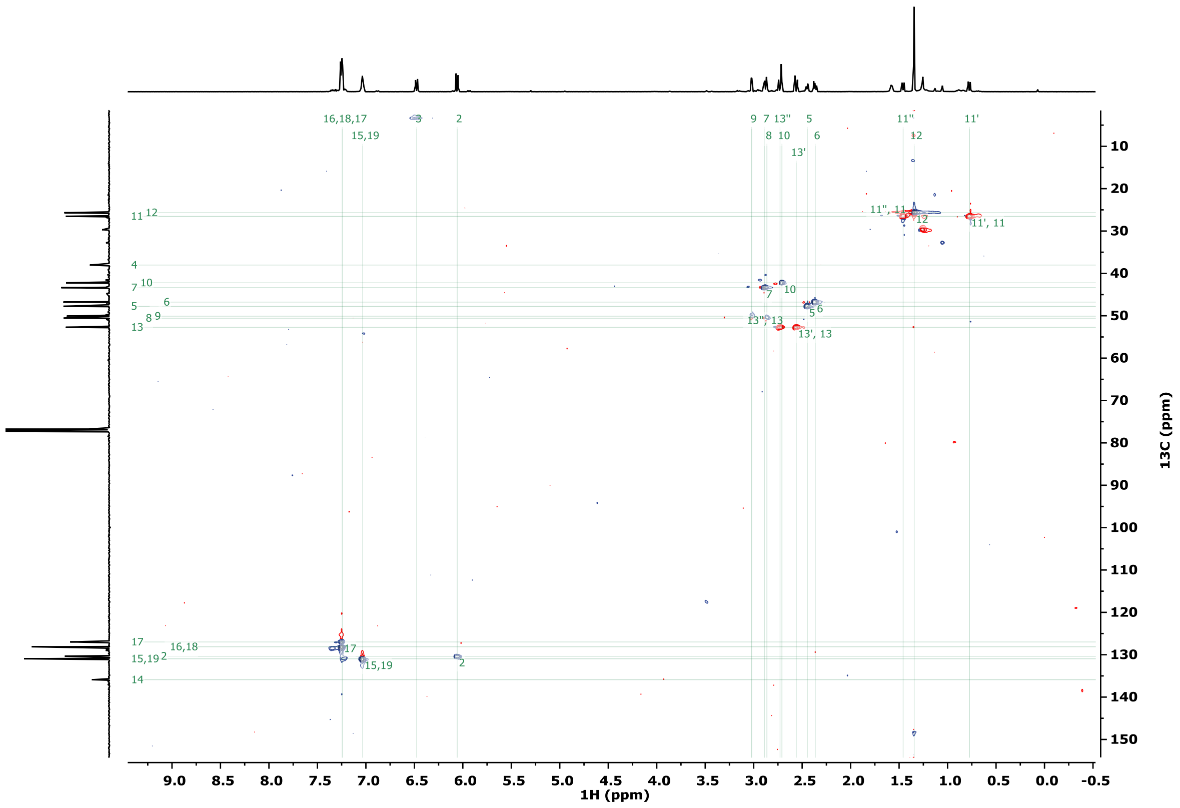

NOESY spectra 11

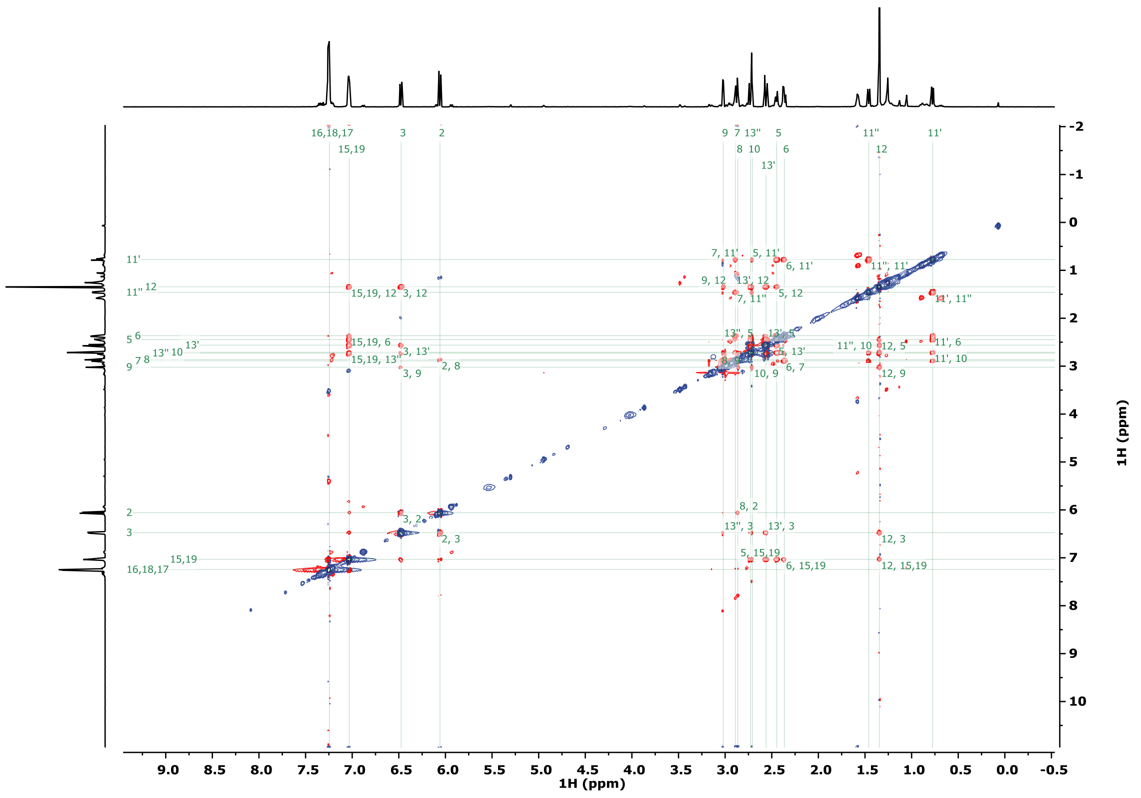

^{1}H NMR and ^{13}C NMR Spectra 12

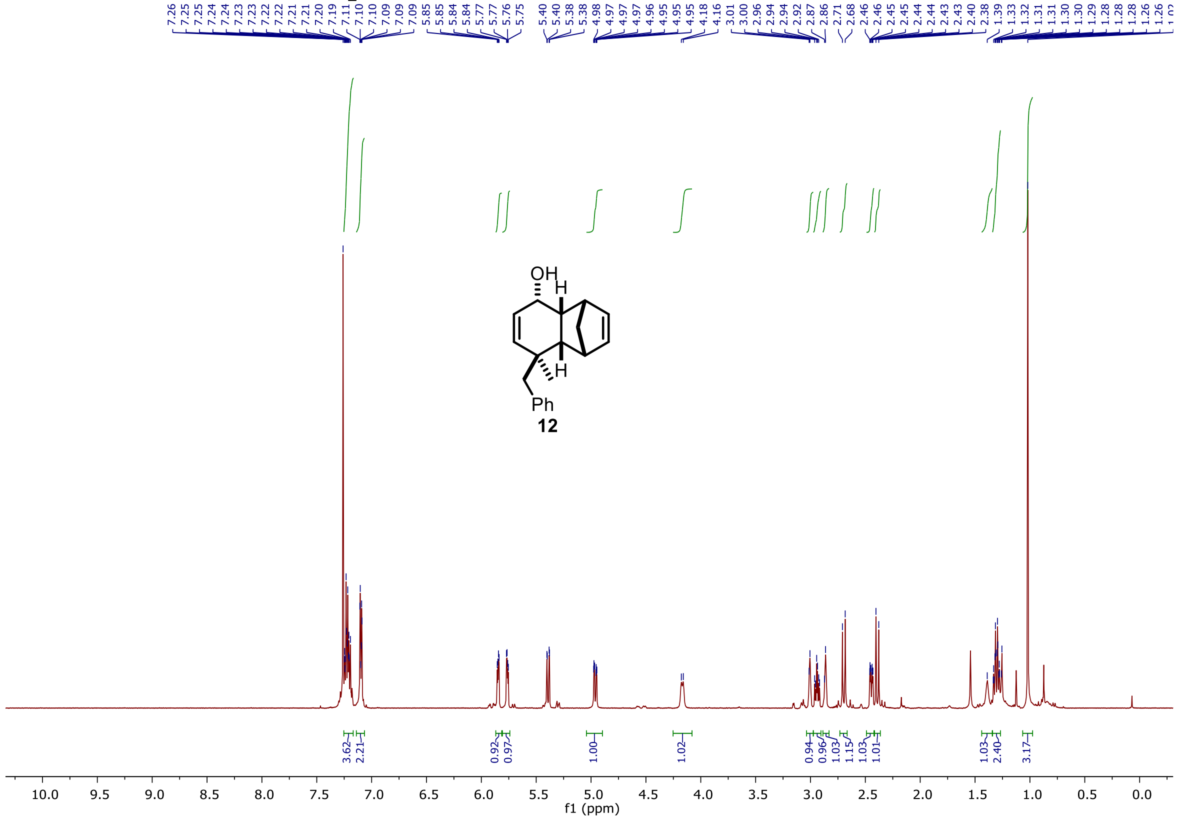

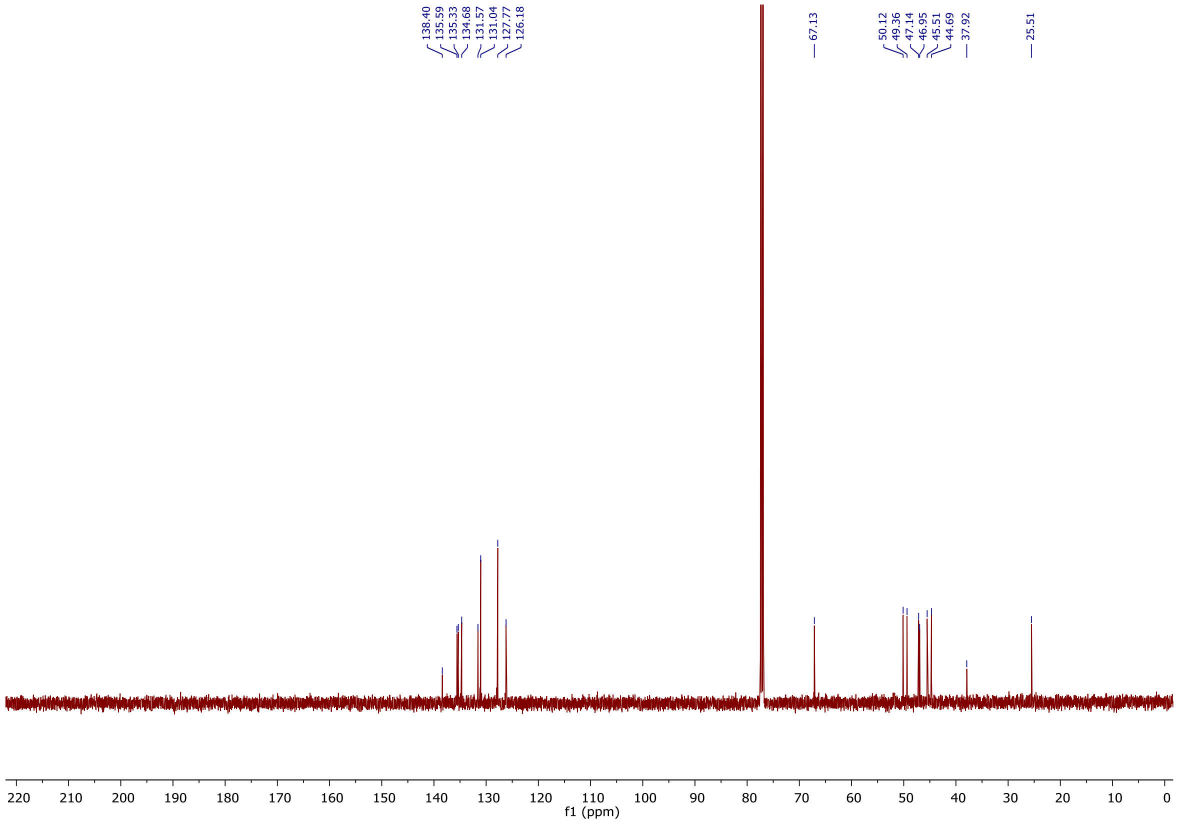

^{1}H NMR, ^{13}C NMR and ^{19}F NMR Spectra 17a

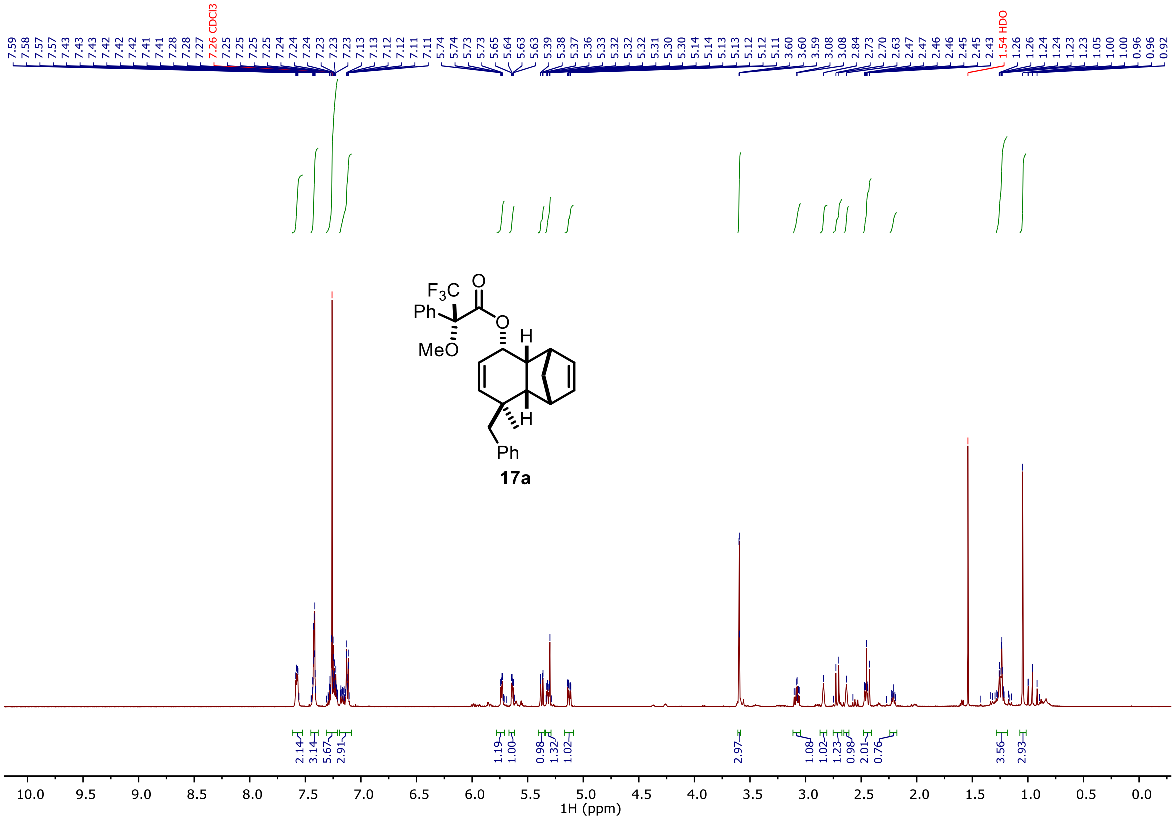

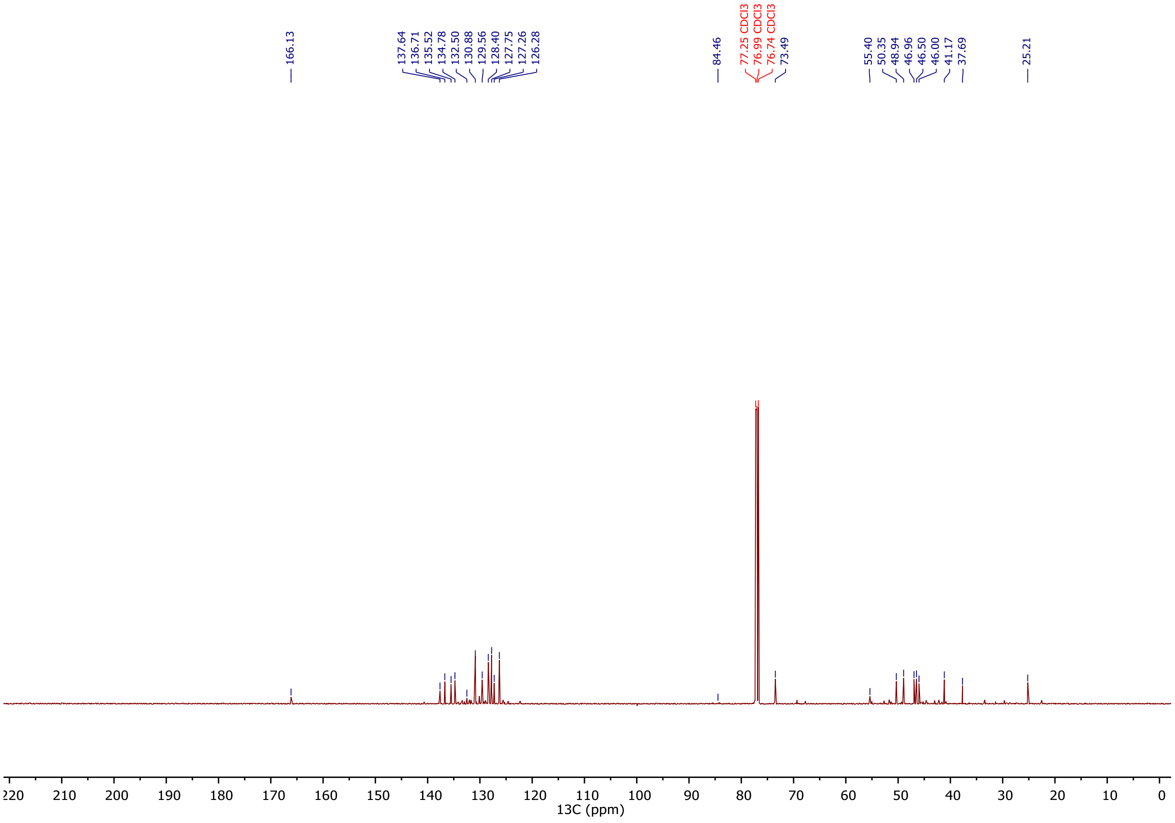

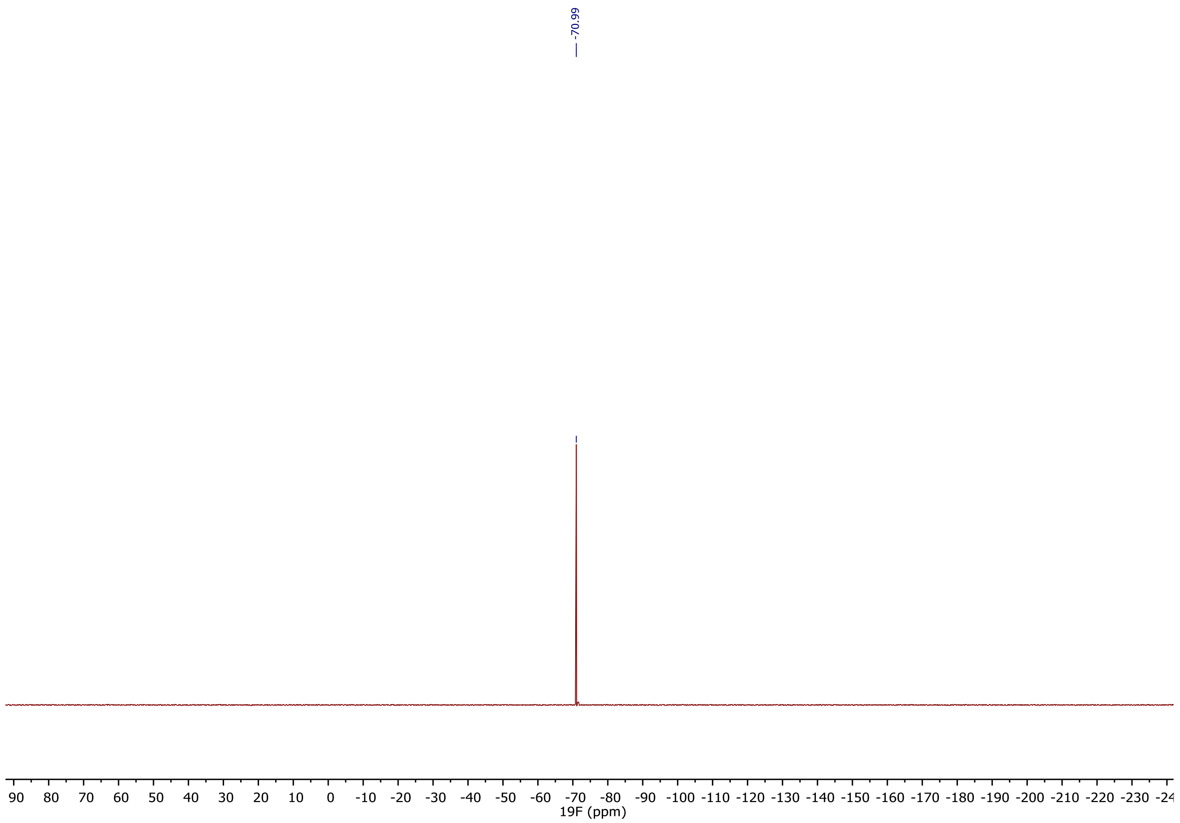

COSY spectra 17a

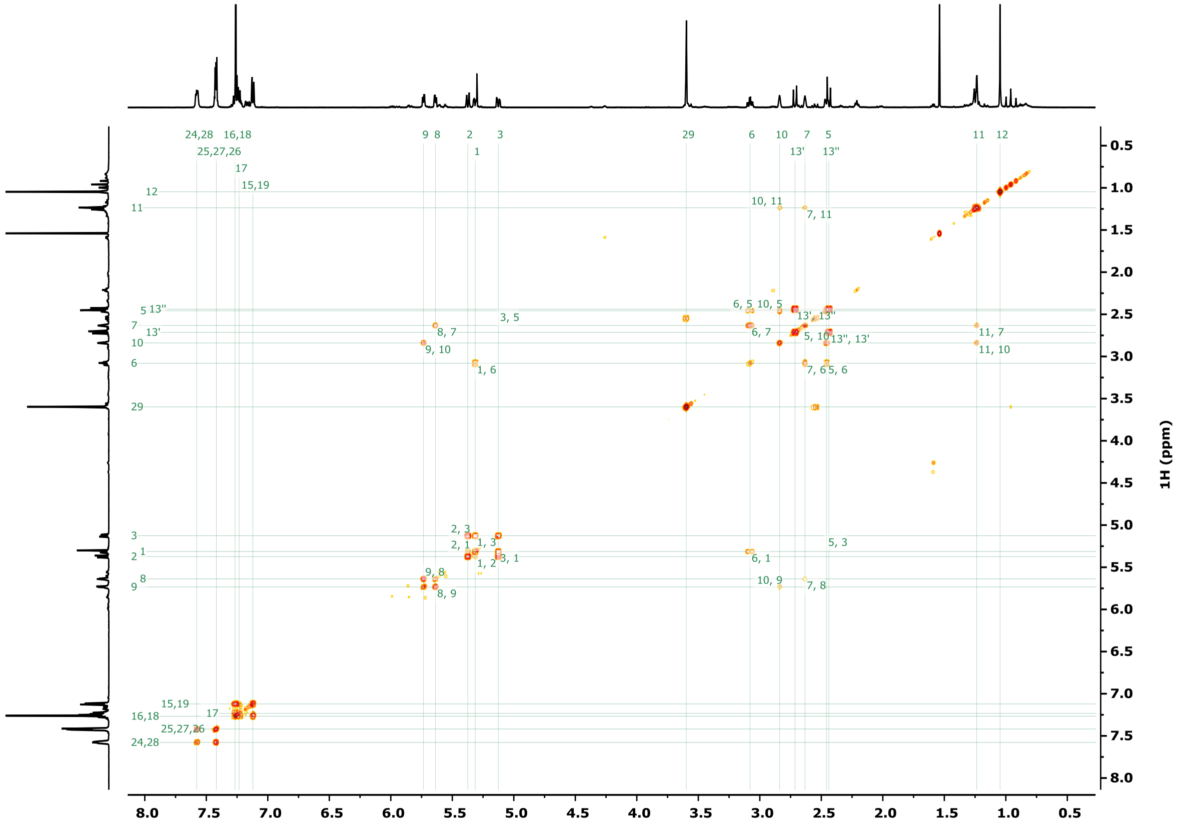

HSQC spectra 17a

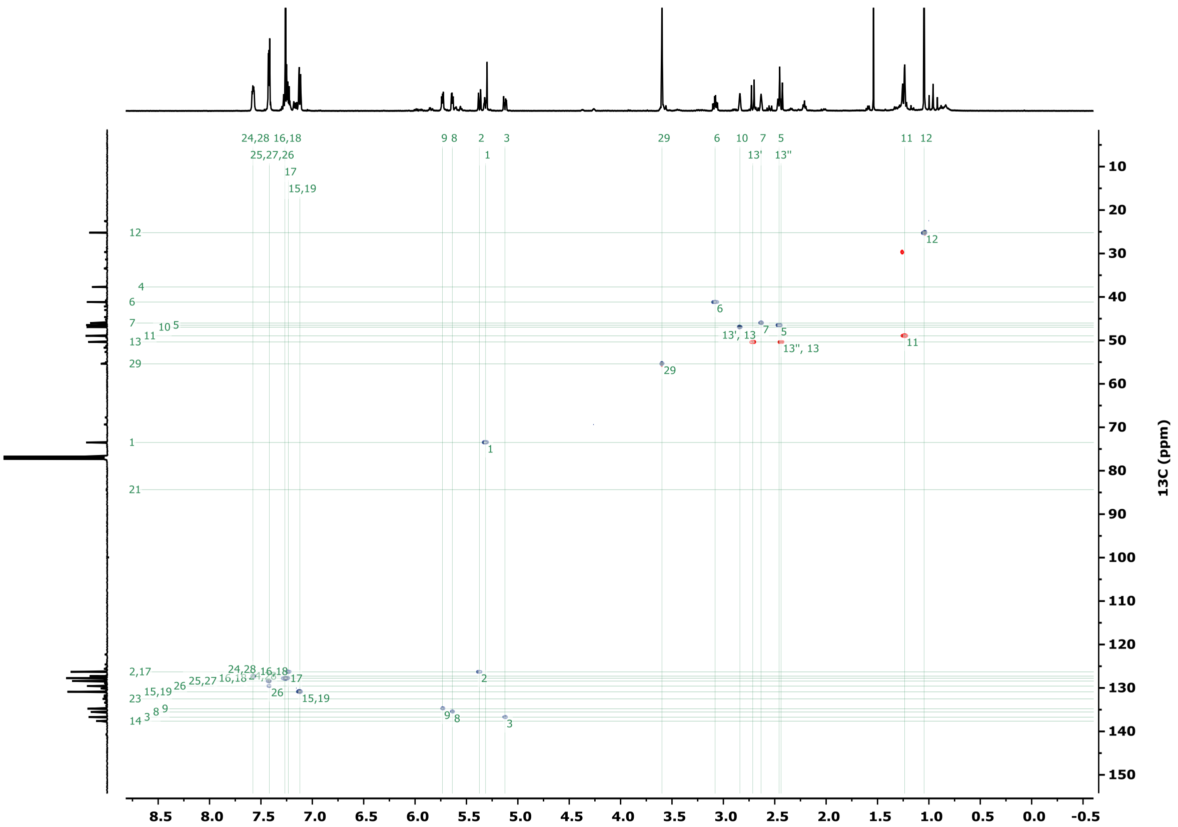

HMBC spectra 17a

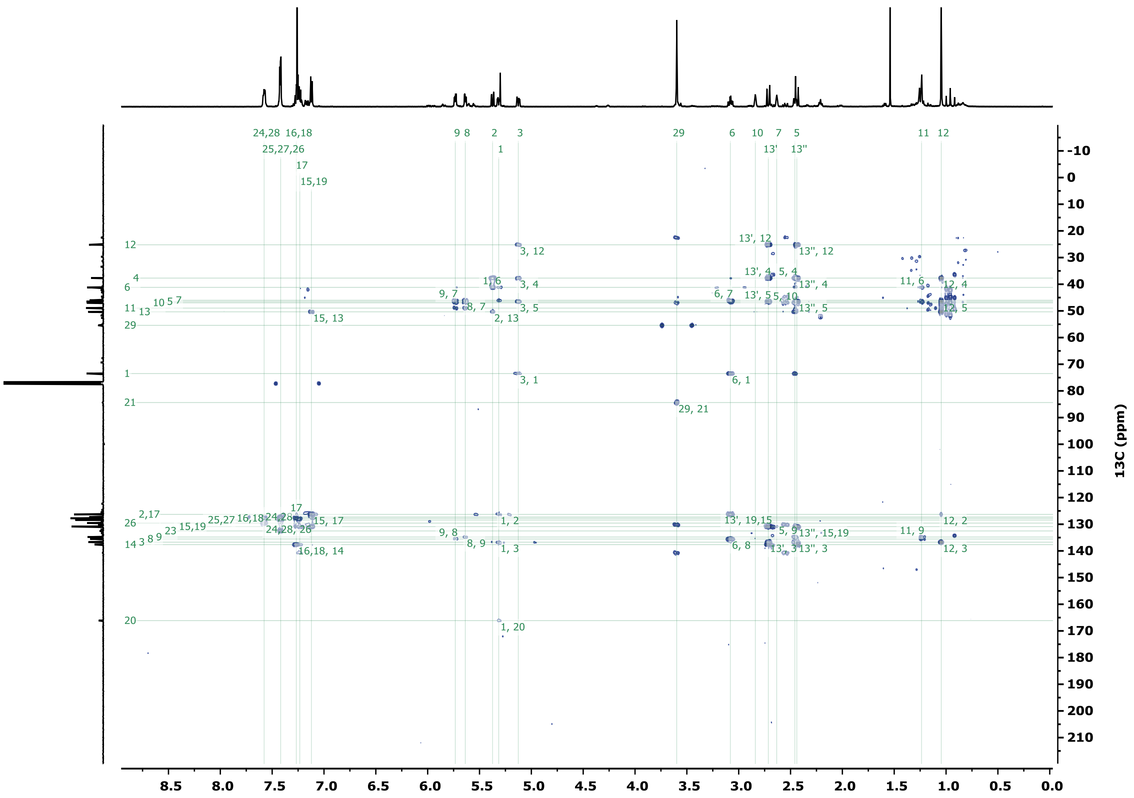

NOSEY spectra 17a

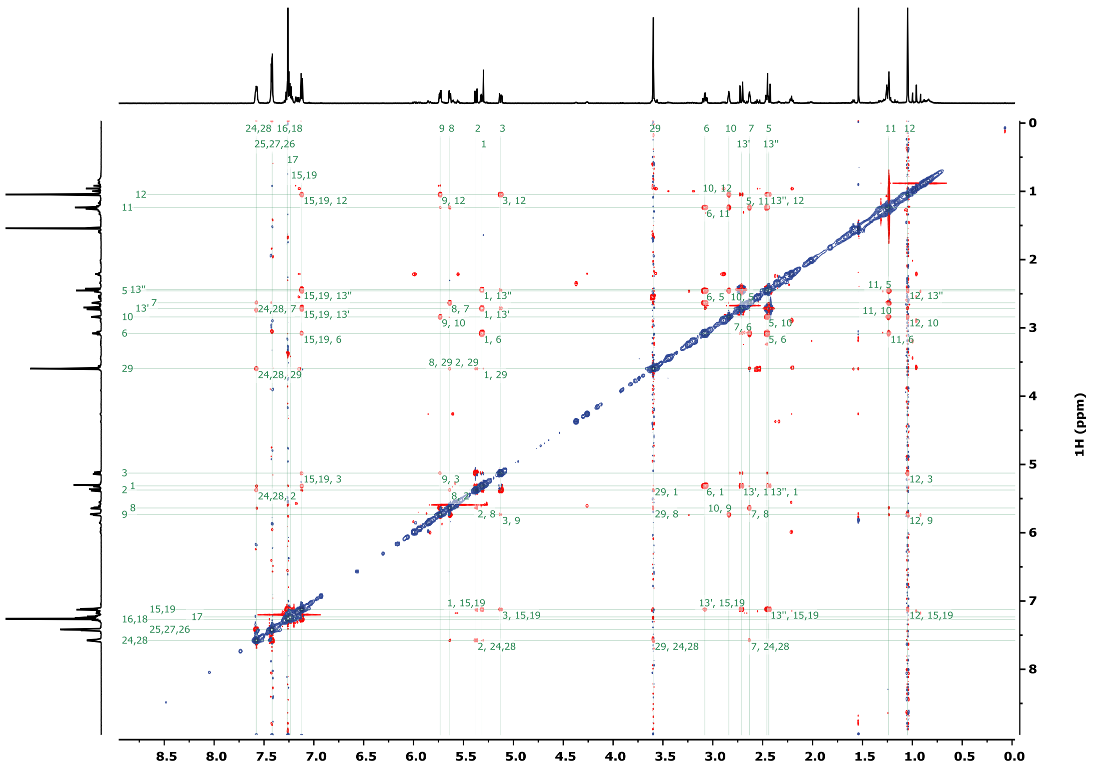

^{1}H NMR, ^{13}C NMR and ^{19}F NMR Spectra 17b

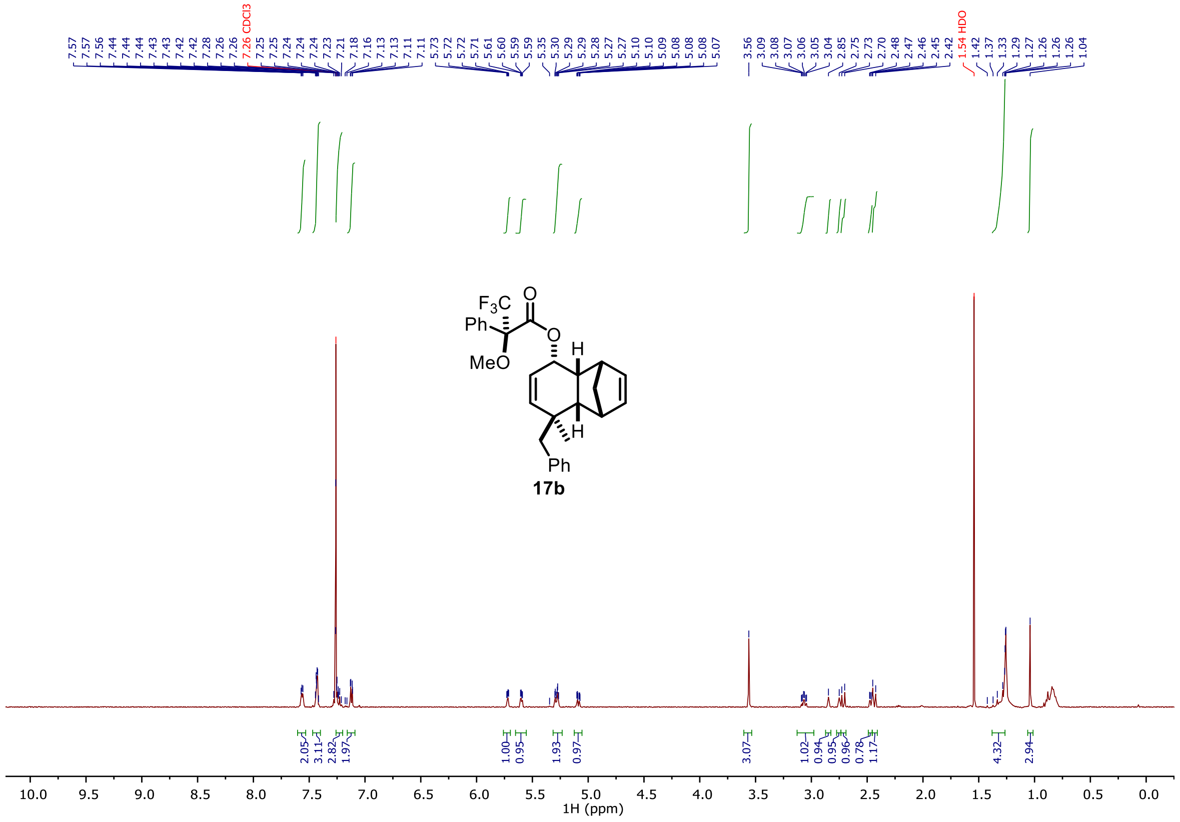

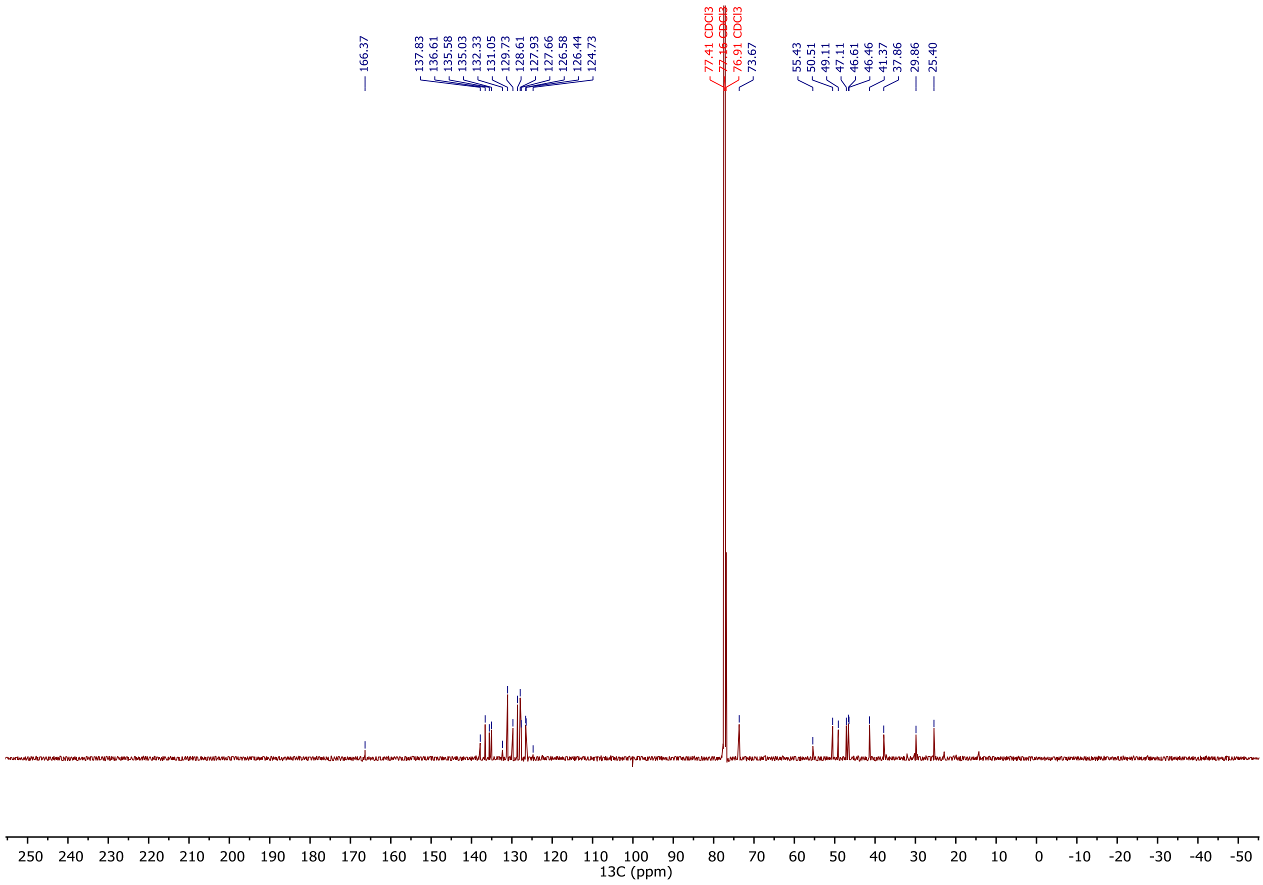
166.37
137.83
136.61
135.58
135.03
132.33
131.05
129.73
128.61
127.93
127.66
126.58
126.44
124.73
77.41 CDCl3
77.16 CDCl3
76.91 CDCl3
73.67
55.43
50.51
49.11
47.11
46.61
46.46
41.37
37.86
29.86
25.40
250 240 230 220 210 200 190 180 170 160 150 140 130 120 110 100 90 80 70 60 50 40 30 20 10 0 -10 -20 -30 -40 -50
13C (ppm)

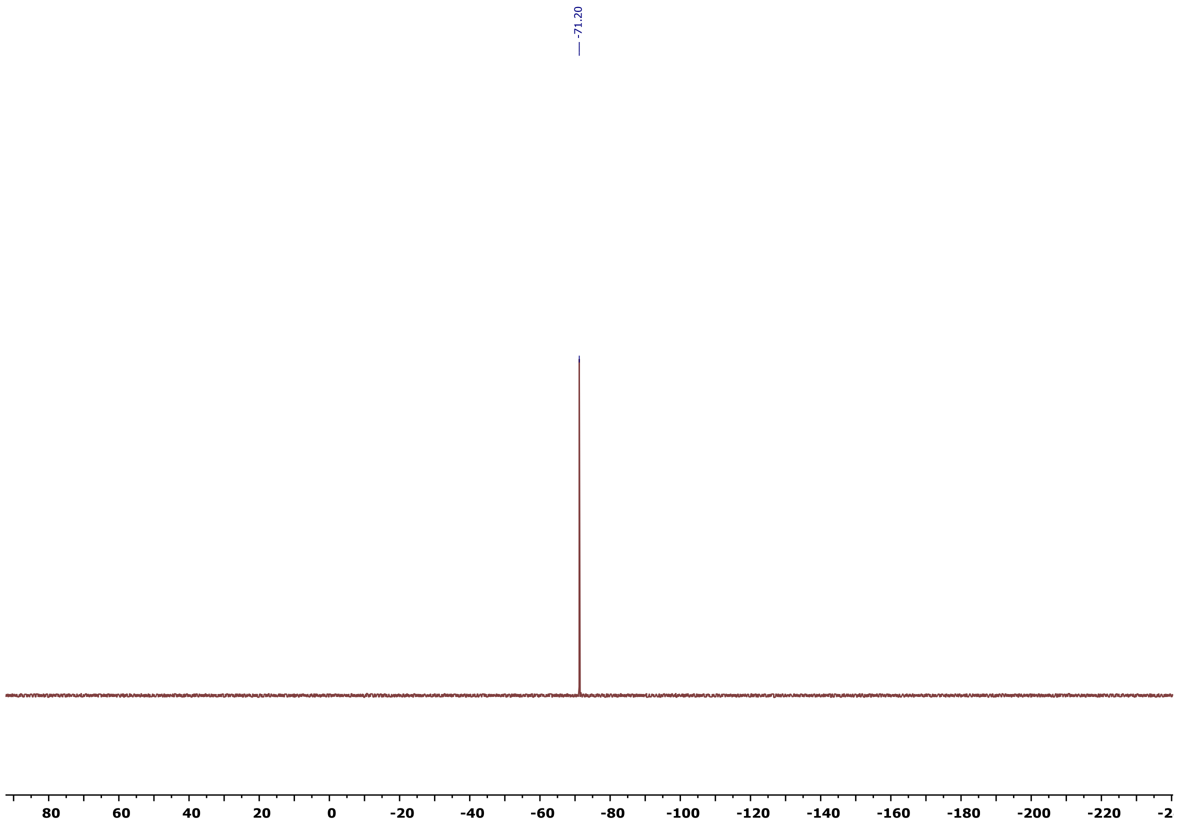
-71.20
80 60 40 20 0 -20 -40 -60 -80 -100 -120 -140 -160 -180 -200 -220 -2

COSY spectra 17b

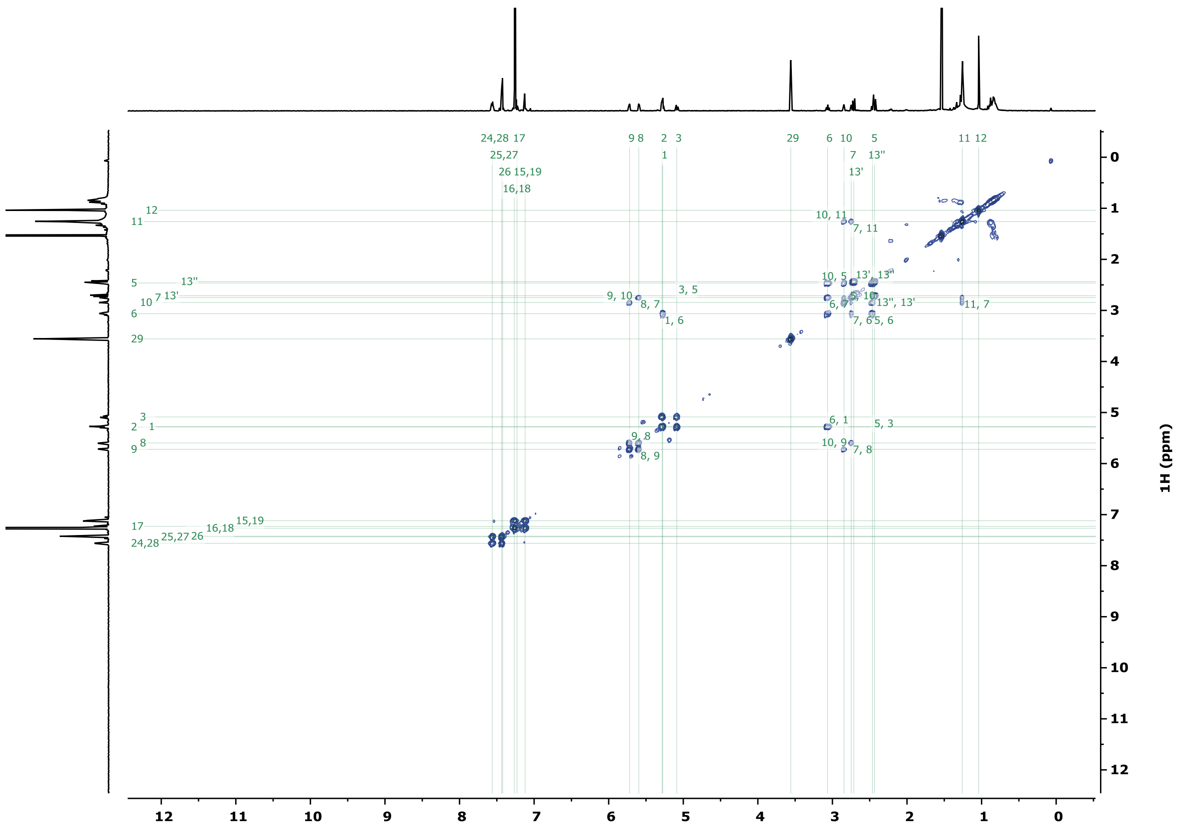

HSQC spectra 17b

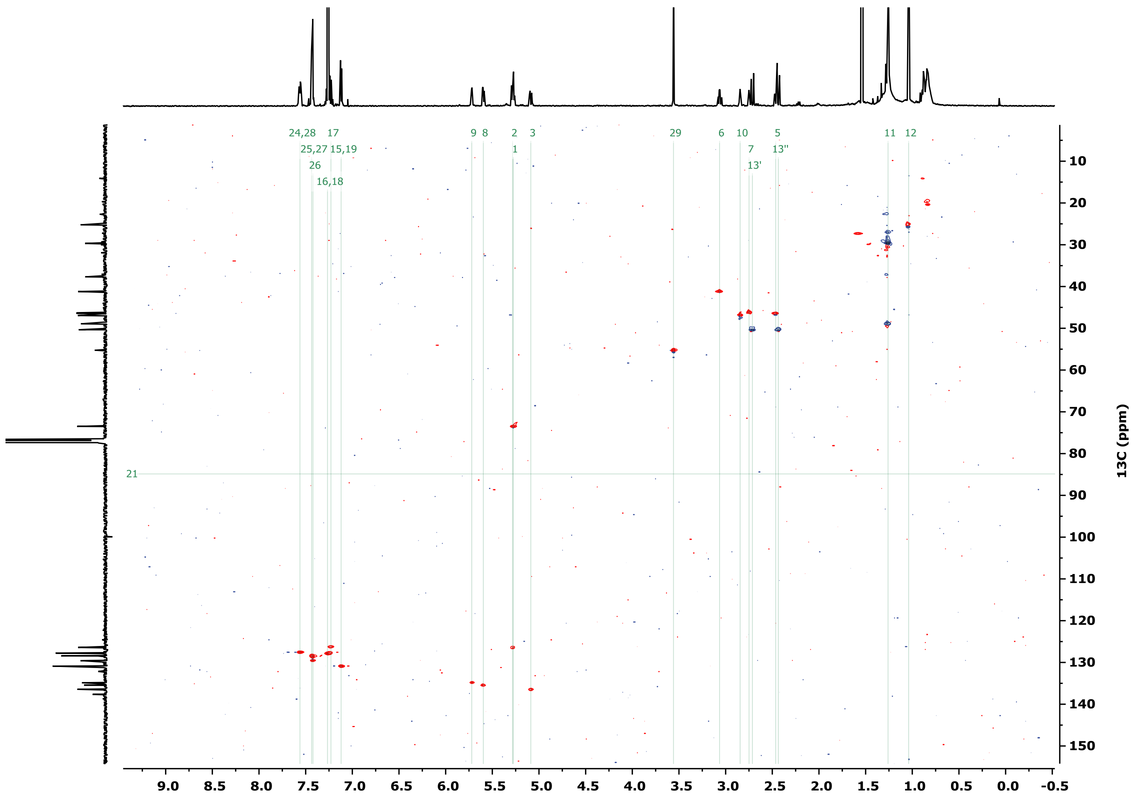

HMBC spectra 17b

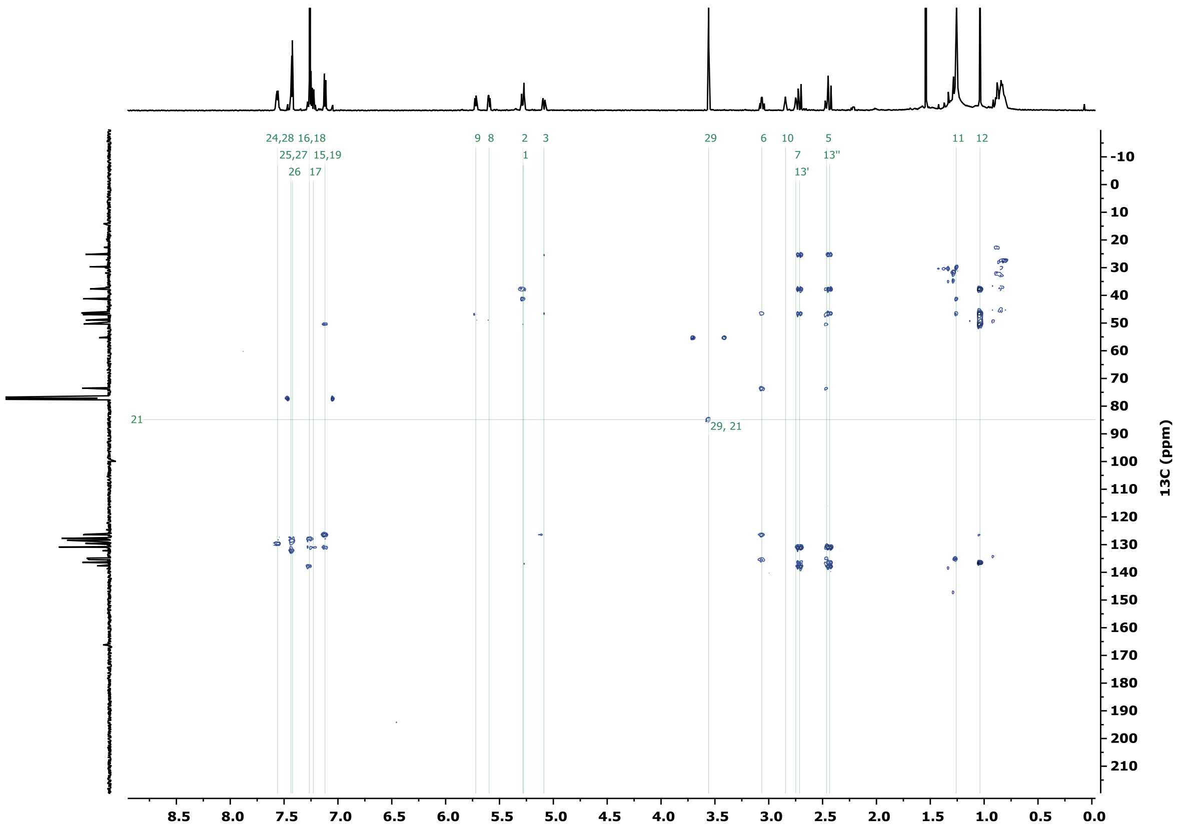

NOESY spectra 17b

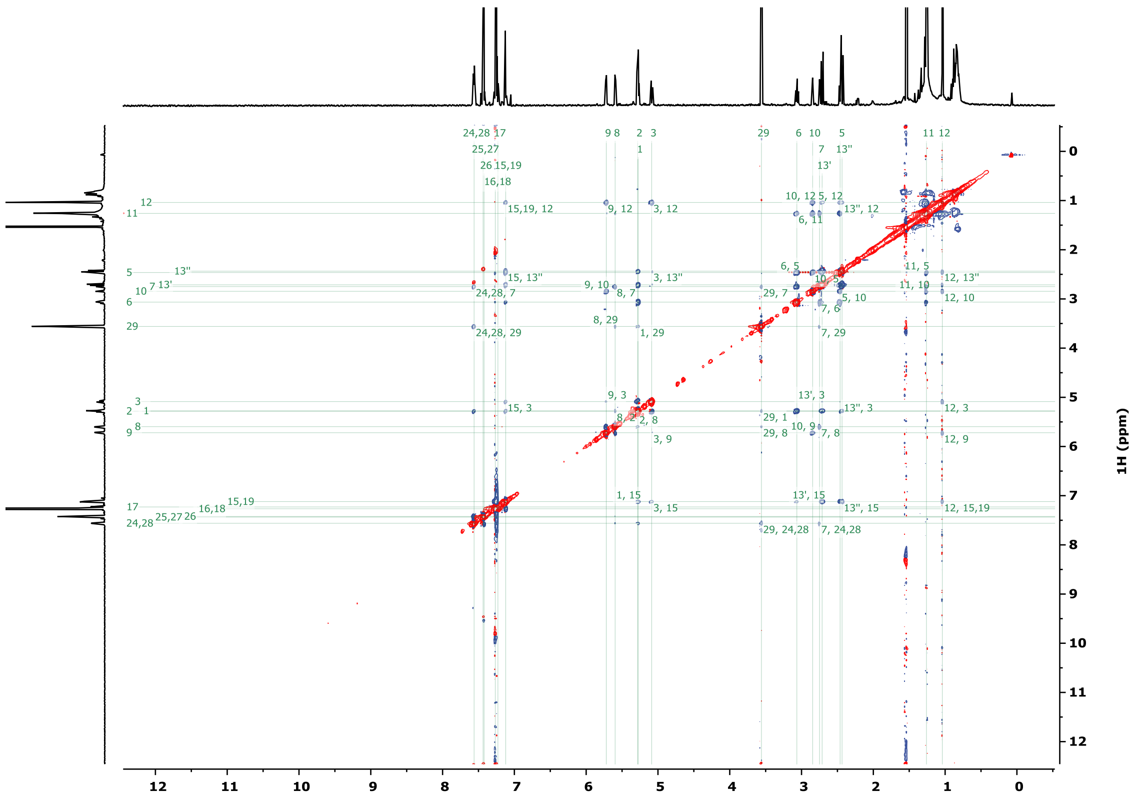

HPLC & GC Traces

HPLC Trace of Racemic 2a and 2a':

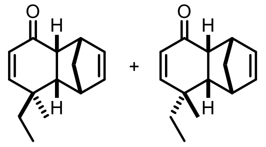

2a **2a'**

Datafile Name:GHS-GA-370.lcd
Sample Name:GHS-GA-370
Sample ID:GHS-GA-370

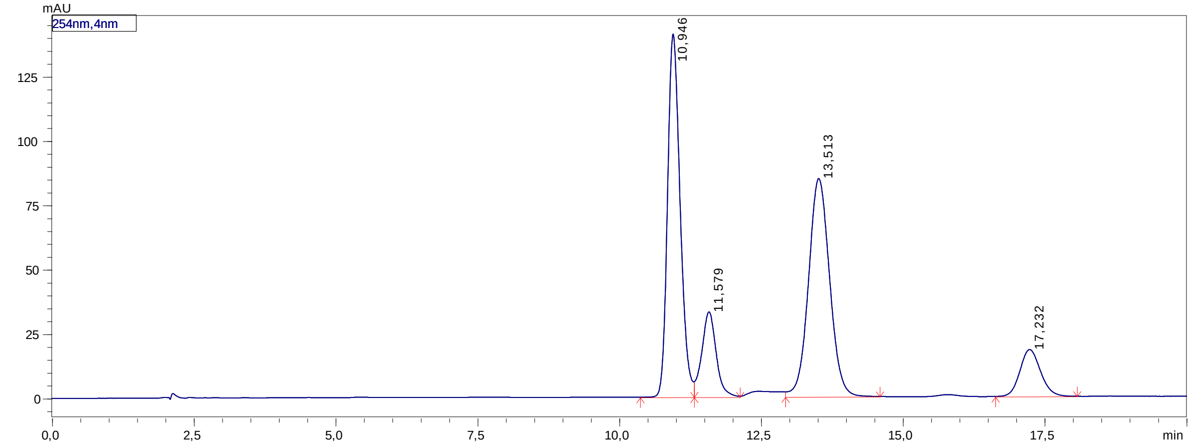

Peak#	Ret. Time	Area%	Name
1	10.946	40.219	E1/D1
2	11.579	10.781	E1/D2
3	13.513	40.514	E2/D1
4	17.232	8.487	E2/D2
Total		100.00	

HPLC Trace of Enantiopure 2a and 2a':

Datafile Name:DAT-SC-646.lcd

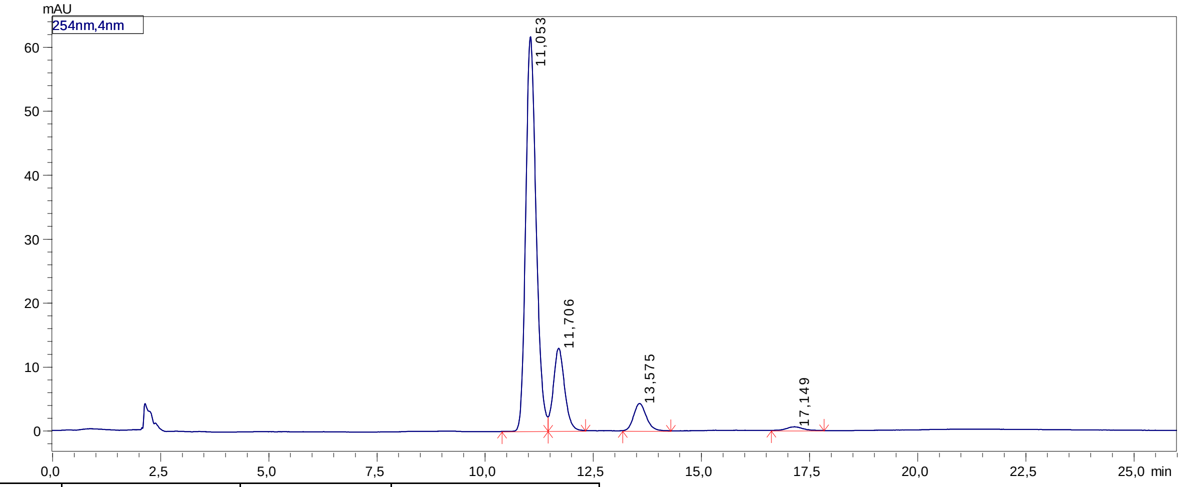

Peak#	Ret. Time	Area%	Name
1	11.053	75.642	E1/D1
2	11.706	17.006	E1/D2
3	13.575	6.296	E2/D1
4	17.149	1.056	E2/D2
Total		100.00	

D1: 92:8 er(major)
D2: 94:6 er (minor)

HPLC Trace of Racemic 2b and 2b':

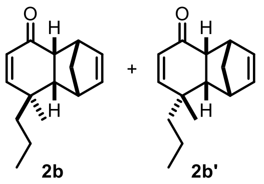

Datafile Name:GHS-GA-293r.lcd
Sample Name:GHS-GA-293r
Sample ID:GHS-GA-293r

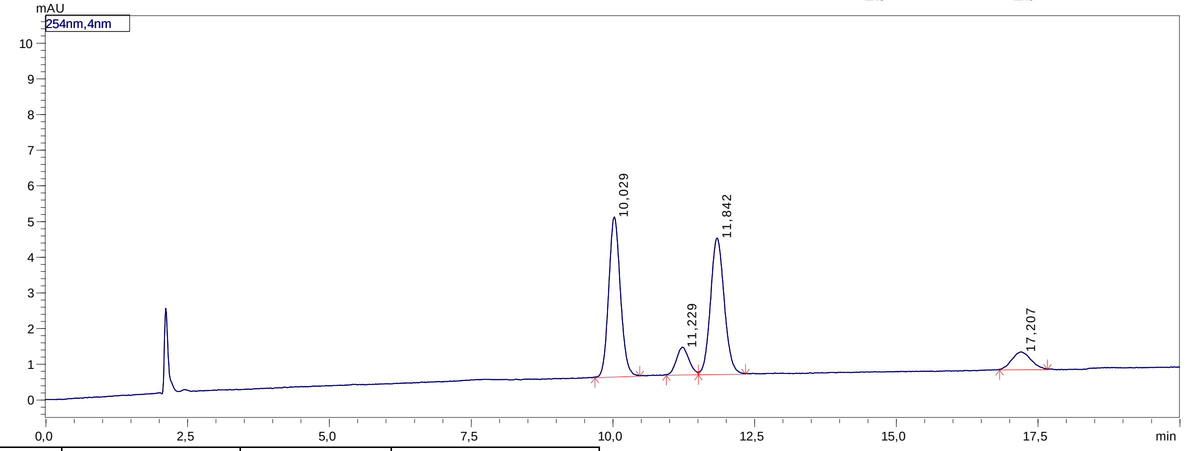

Peak#	Ret. Time	Area%	Name
1	10.029	42.501	E1/D1
2	11.229	7.725	E1/D2
3	11.842	42.468	E2/D1
4	17.207	7.306	E2/D2
Total		100.00	

HPLC Trace of Enantiopure 2b and 2b':

Datafile Name:DAT-SC-631.lcd

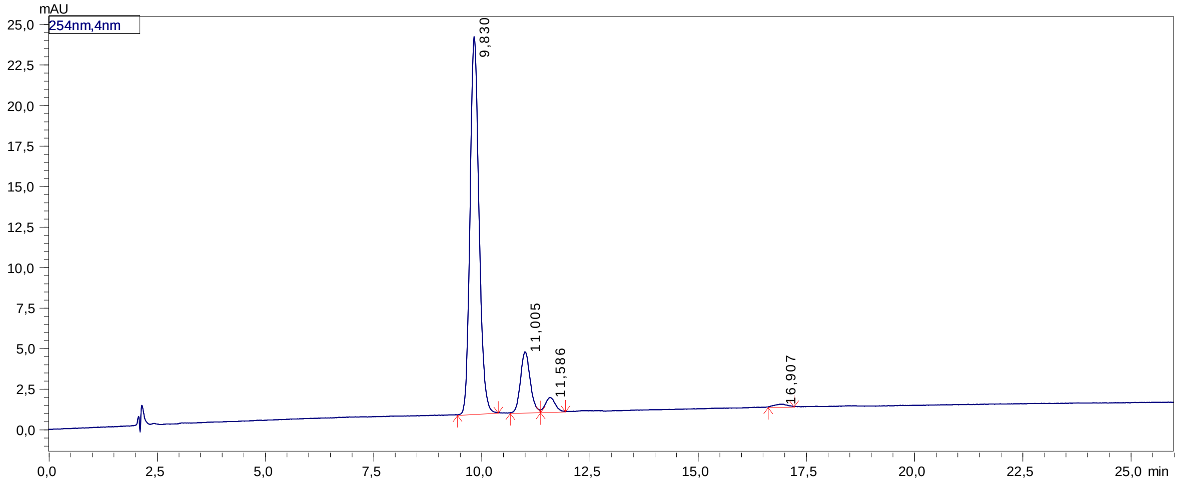

Peak#	Ret. Time	Area%	Name
1	9.830	81.755	E1/D1
2	11.005	14.152	E1/D2
3	11.586	3.342	E2/D1
4	16.907	0.751	E2/D1
Total		100.00	

D1: 96:4 er (major)
D2: 95:5 er (minor)

HPLC Trace of Racemic 2c:

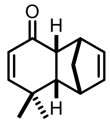

2c

Datafile Name:GHS-GA-446.lcd
Sample Name:GHS-GA-446
Sample ID:GHS-GA-446

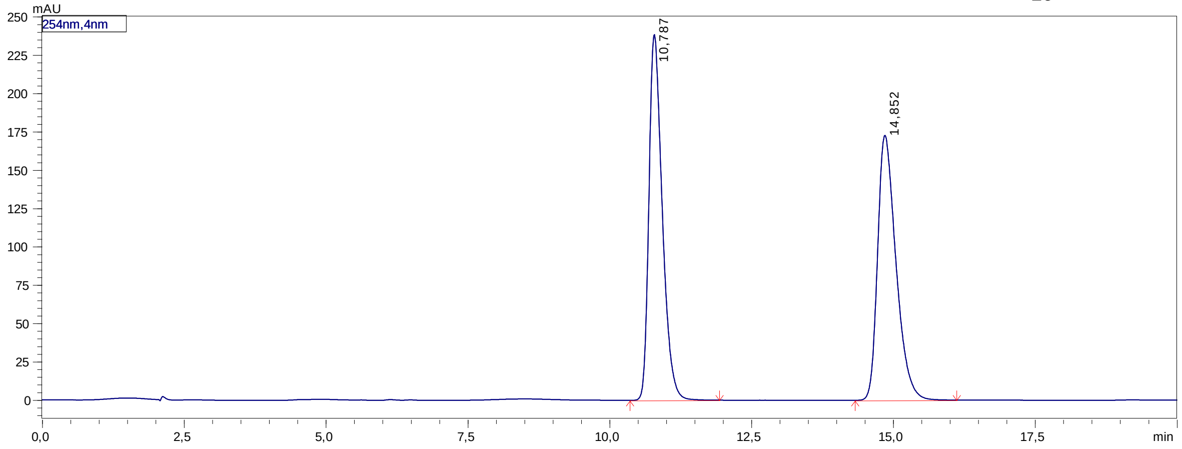

Peak#	Ret. Time	Area%
1	10.787	49.829
2	14.852	50.171
Total		100.00

HPLC Trace of Enantiopure 2c:

Datafile Name:DAT-SC-641.lcd

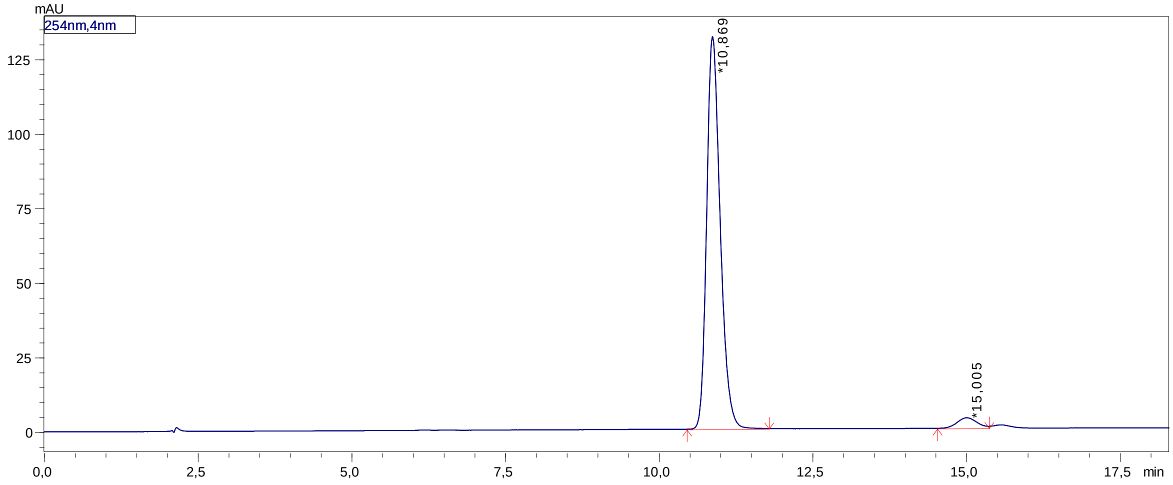

Peak#	Ret. Time	Area%
1	10.869	96.215
2	15.005	3.785
Total		100.00

HPLC Trace of Racemic 2d:

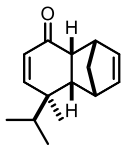

2d

Datafile Name:GHS-GA-416-IC3.lcd
Sample Name:GHS-GA-416
Sample ID:GHS-GA-416

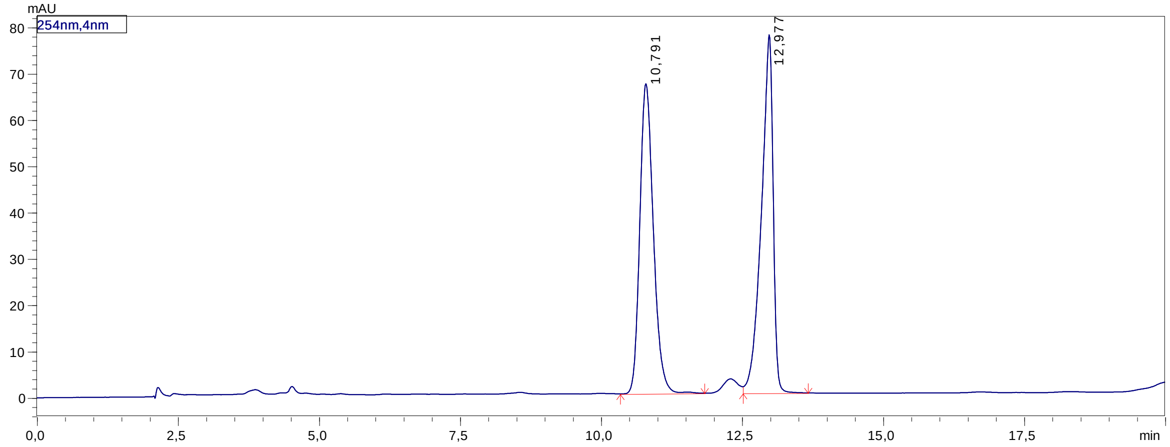

Peak#	Ret. Time	Area%
1	10.791	49.999
2	12.977	50.001
Total		100.00

HPLC Trace of Enantiopure 2d:

Datafile Name:DAT-SC-647.lcd

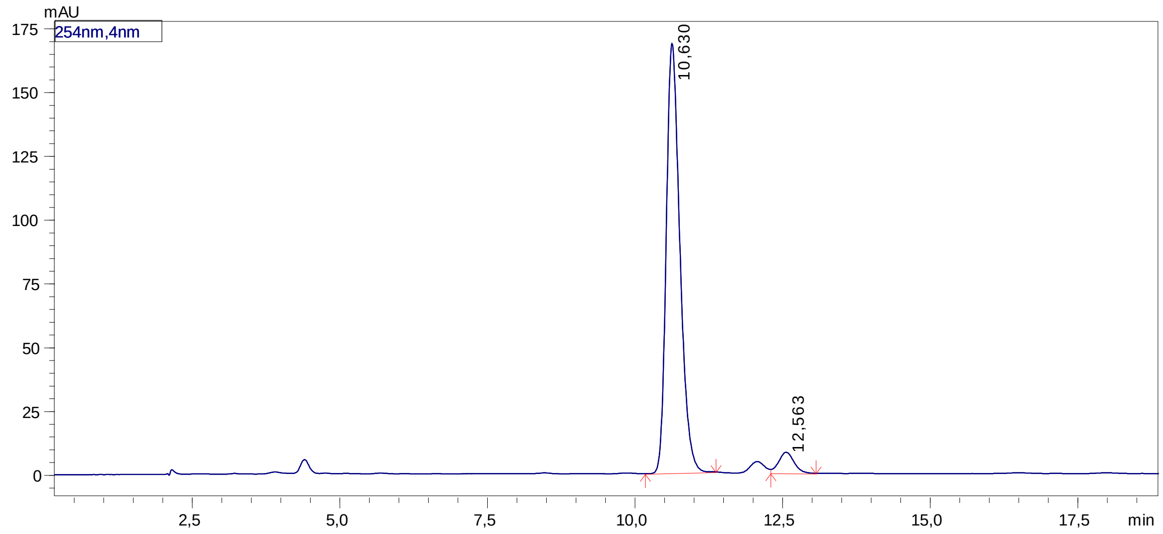

Peak#	Ret. Time	Area%
1	10.630	94.995
2	12.563	5.005
Total		100.00

HPLC Trace of Racemic 2e and 2e':

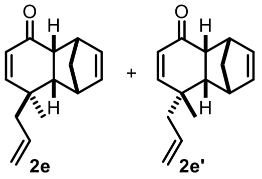

Gerät : Method Scout

Operator : Ke
Sample Name : GHS-GA-487-01
Vial # : 31
Injection Volume : 2 uL
Data File Name : GHS-GA-487-01-04.lcd
Method File Name : Gosh-chiral.lcm

Data Acquired: 16.04.2019 11:17:49

2 µL GHS-GA-487-01 (in 1 mL ACN)
150 mm Chiralpak AS-3R, 4.6 mm i.D.
Acetonitril / Wasser-Gradient:
50% - 10' - 90% B
1.0 ml/min, 22.0 MPa, 298 K
220 nm

Säulenauswahlventil
<<Oven>>
Valve L : 0
Valve R : 5

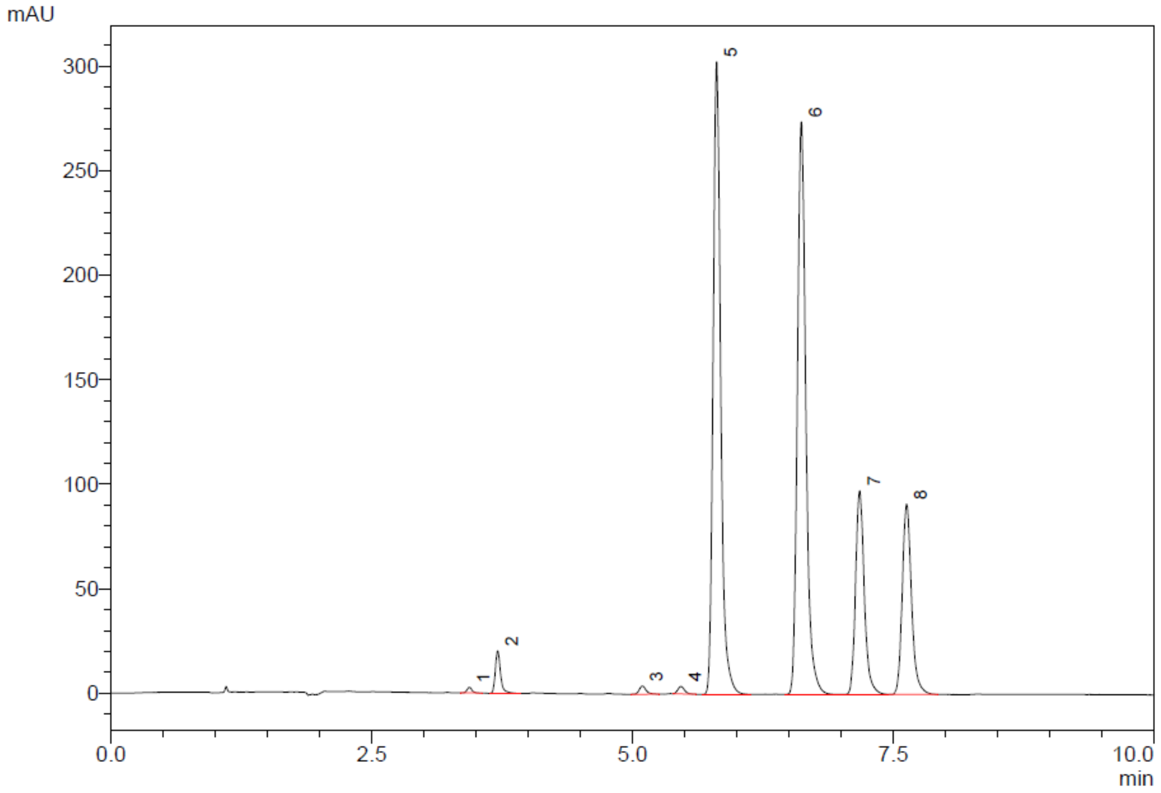

1 220nm,4nm

PDA Ch1 220nm

Peak #	Ret. Time	Area %	Name
1	3.44	0.21	
2	3.71	1.64	
3	5.09	0.41	
4	5.46	0.36	
5	5.81	35.44	E1/D1
6	6.62	35.80	E2/D1
7	7.18	13.13	E1/D2
8	7.63	13.00	E2/D2
Total		100.00	

HPLC Trace of Enantiopure 2e and 2e':

Gerät : Method Scout

Operator : St

Data Acquired: 21.05.2019 09:44:26

Sample Name : DAT-SC-661-01
Vial # : 2
Injection Volume : 1 uL
Data File Name : DAT-SC-661-01-01.lcd
Method File Name : Das-chiral.lcm

Säulenauswahlventil
<<Oven>>
Valve R : 5

1 µL DAT-SC-661-01 (in 1mL ACN)
150 mm Chiralcel AS-3R 4.6 mm i.D.
Acetonitril/Wasser - Gradient:
50 % B - 10` - 90% B
1.0 ml/min, 22.0 MPa, 298 K
220 nm

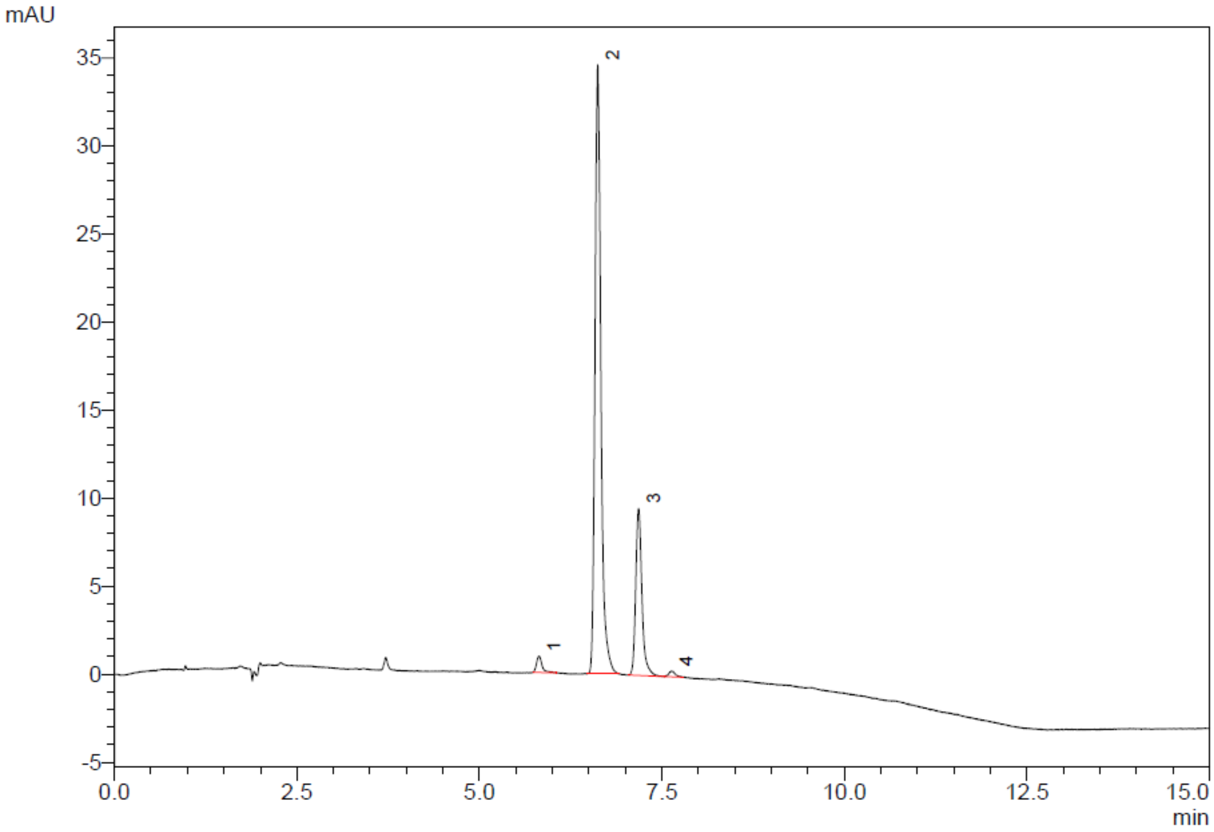

1 220nm,4nm

PDA Ch1 220nm

Peak #	Ret. Time	Area %	Name	
1	5.82	1.85	1D/1E	ee = 95.2 %
2	6.62	75.67	1D/2E	
3	7.18	21.69	2D/1E	ee = 93.0 %
4	7.63	0.79	2D/2E	
Total		100.00		

HPLC Trace of Racemic 2f and 2f':

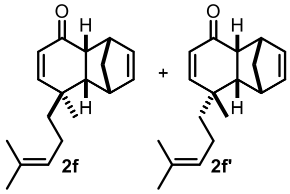

Datafile Name:GHS-GA-609_IC-3_5%_1mL002.lcd
Sample Name:GHS-GA-609
Sample ID:GHS-GA-609

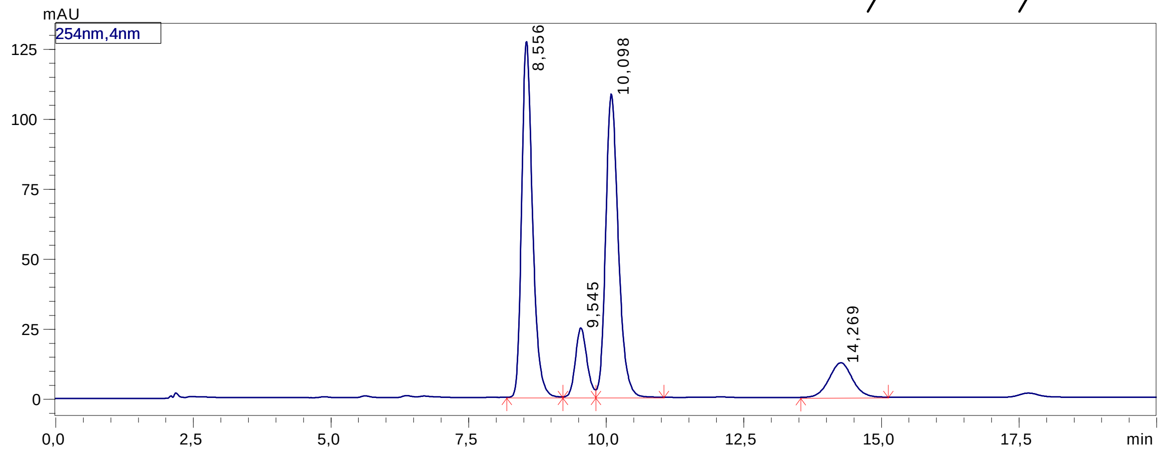

Peak#	Ret. Time	Area%	Name
1	8.556	41.464	E1/D1
2	9.545	8.603	E1/D2
3	10.098	41.298	E2/D1
4	14.269	8.635	E2/D2
Total		100.00	Name

HPLC Trace of Enantiopure 2f and 2f':

Datafile Name:GHS-GA-608_IC-3_5%_1mL002.lcd
Sample Name:GHS-GA-608
Sample ID:GHS-GA-608

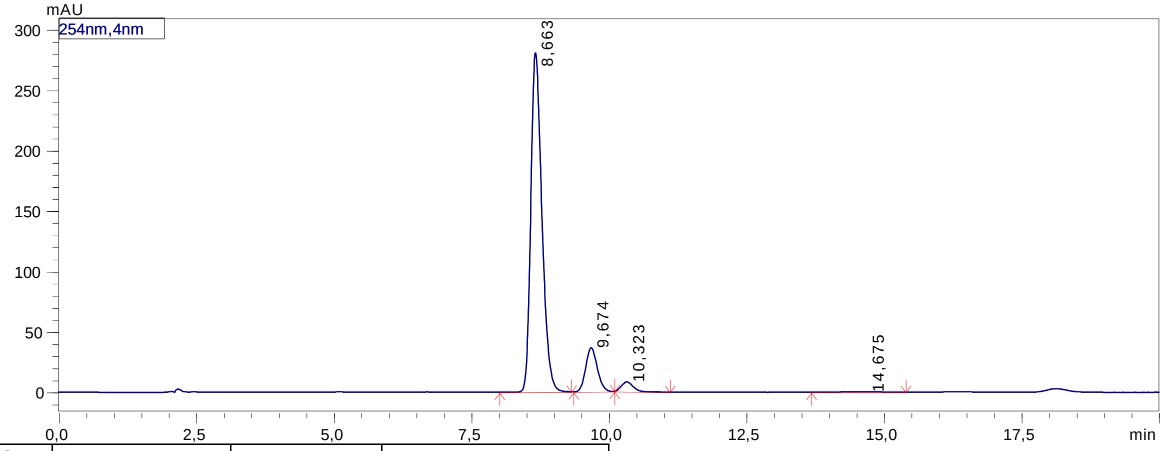

Peak#	Ret. Time	Area%	Name
1	8.663	85.596	E1/D1
2	9.674	11.489	E1/D2
3	10.323	2.523	E2/D1
4	14.676	0.391	E2/D2
Total		100.00	

D1: 97:3 er (major)
D2: 96.5:3.5 er (minor)

HPLC Trace of Racemic 2g:

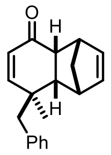

2g

Datafile Name:GHS-GA-376 OJ-3 95 5.lcd
Sample Name:GHS-GA-376
Sample ID:GHS-GA-376

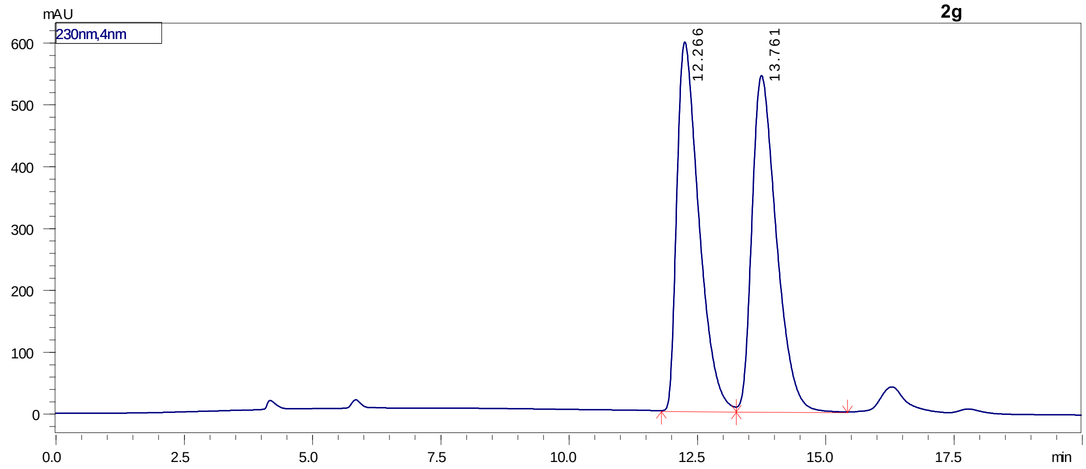

Peak#	Ret. Time	Area%
1	12.266	49.832
2	13.761	50.168
Total		100.00

HPLC Trace of Enantiopure 2g:

Datafile Name:GHS-GA-575OJ-3 95- 5_0.5.lcd
Sample Name:GHS-GA-575
Sample ID:GHS-GA-575

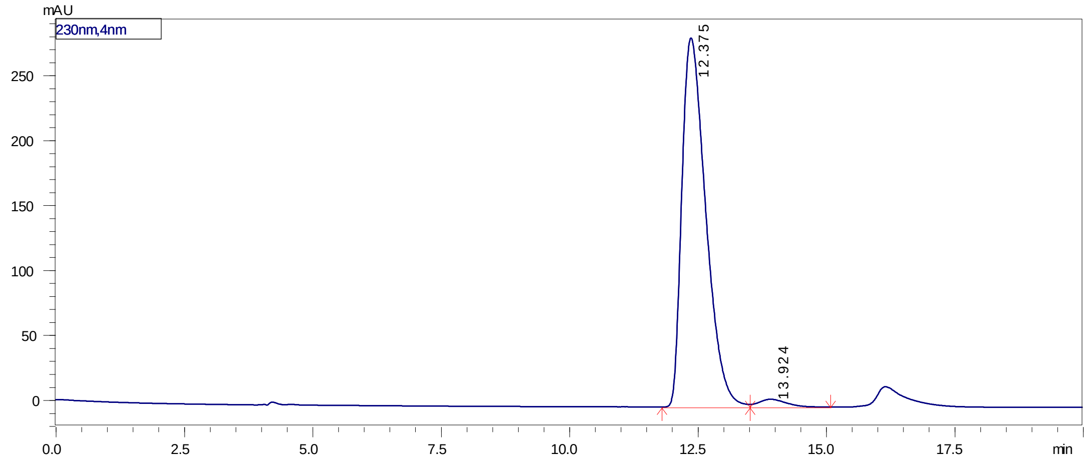

Peak#	Ret. Time	Area%
1	12.375	97.622
2	13.924	2.378
Total		100.00

HPLC Trace of Racemic 2h and 2h':

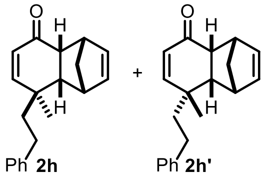

Datafile Name:GHS-GA-485.lcd
Sample Name:GHS-GA-485
Sample ID:GHS-GA-485

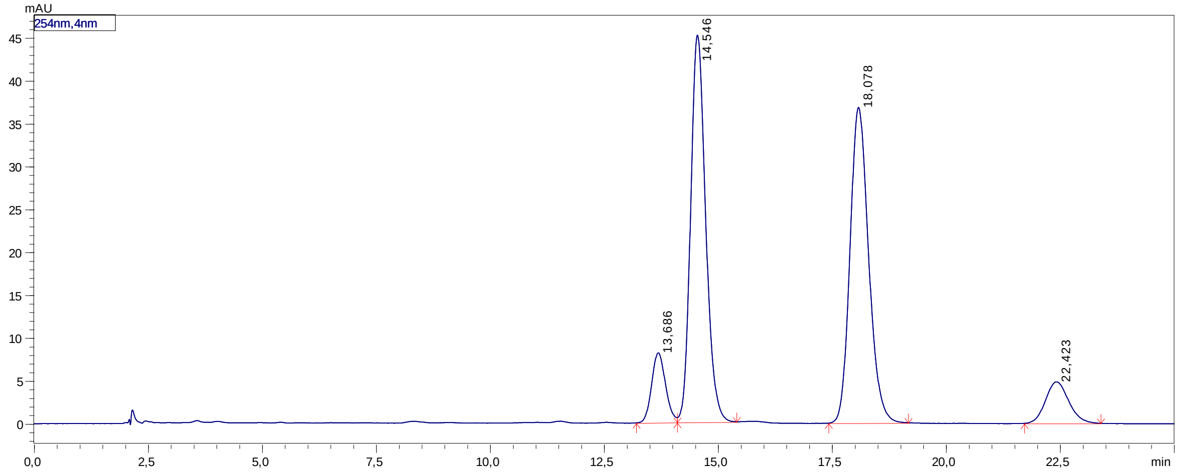

Peak#	Ret. Time	Area%	
1	13.686	7.104	D2/A1
2	14.546	42.749	D1/A1
3	18.078	43.102	D1/A2
4	22.423	7.046	D2/A2
Total		100.00	

HPLC Trace of Enantiopure 2h and 2h':

Datafile Name:DAT-SC-650-CC.lcd

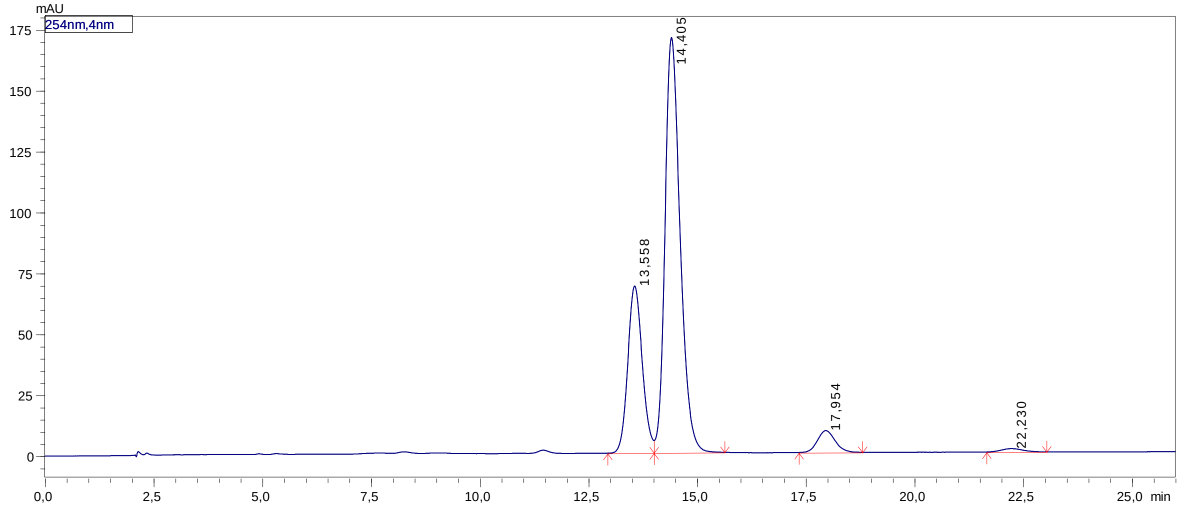

Peak#	Ret. Time	Area%	
1	13.558	26.443	D2/A1
2	14.405	68.612	D1/A1
3	17.954	4.171	D1/A2
4	22.230	0.773	D2/A2
Total		100.00	

D1: 94:6 er (major)
D2: 97:3 er (minor)

HPLC Trace of Racemic 2i and 2i':

Agilent

2D-LC Results Report

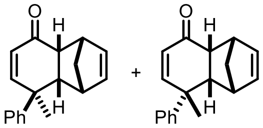

2i 2i'

Data File:	D:\Data\Kundendaten\Ghosh\GHS-GA-371-01006.D
Sample Name:	GHS-GA-371-01
Description:	2 µl GHS-GA-371-01 (in 2-Propanol)<br>50 mm Eclipse Plus C18 1.8 µm, 4.6 mm i.D.<br>Methanol / Wasser-Gradient:<br>60% - 5' - 90% B<br>1.0 ml/min, 44.8 MPa, 308 K<br>UV 220 nm<br><br>Heart Cut peak based 3', 150 mAu<br><br>2. Dimension:<br>150 mm Chiralcel OJ-3R, 4.6 mm i.D.,<br>Acetonitril / Wasser = 70:30<br>1.0 ml/min, 19.9 MPa, 298 K<br>UV, 220 nm

Instrument:	2D-LC ohne FS	Location:	P1-E-01
Injection date:	11-Mar-19, 15:32:37	Injection volume:	2.00
Acq. method:	Gosh-HC.M	Acq. operator:	SYSTEM

¹D chromatogram(s)

achiral

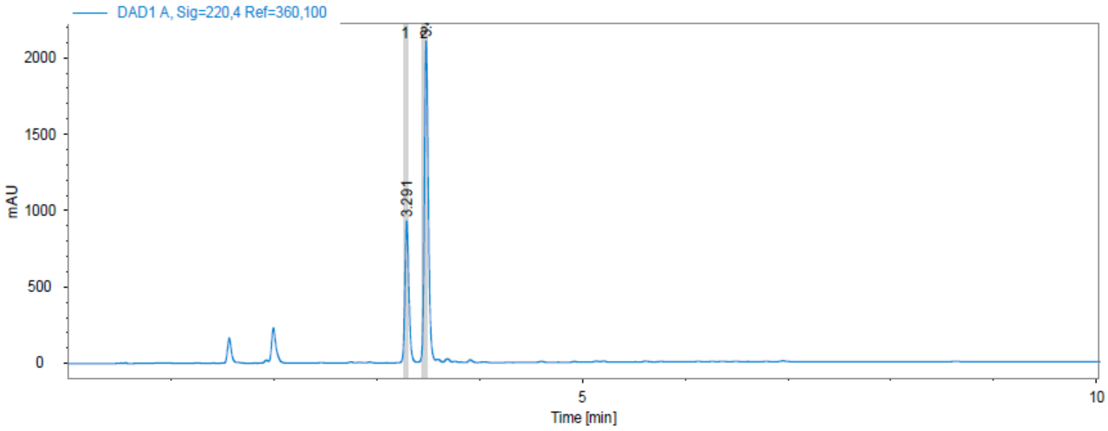

Sampling table (¹D)

Cut group	Cut #	¹D Cut start [min]	¹D Ret. time [min]	¹D Duration [min]	Trigger	²D Run start [min]
	1	3.26	3.291	0.04	Peak	3.31
	2	3.44	3.480	0.04	Peak	12.61

Component table

Signal: DAD2 A, Sig=220,4 Ref=360,100

chiral

Component	¹D Sampling range [min]	Ret.Time ²D [min]	Area	Area%	
1	3.26 - 3.30	3.578	4977.514	20.179	1st diastereomer
2	3.26 - 3.30	4.335	4913.223	19.919	
3	3.44 - 3.48	4.893	7401.426	30.006	2nd diastereomer
4	3.44 - 3.48	5.687	7374.206	29.896	

Agilent

2D-LC Results Report

Cut# : 1

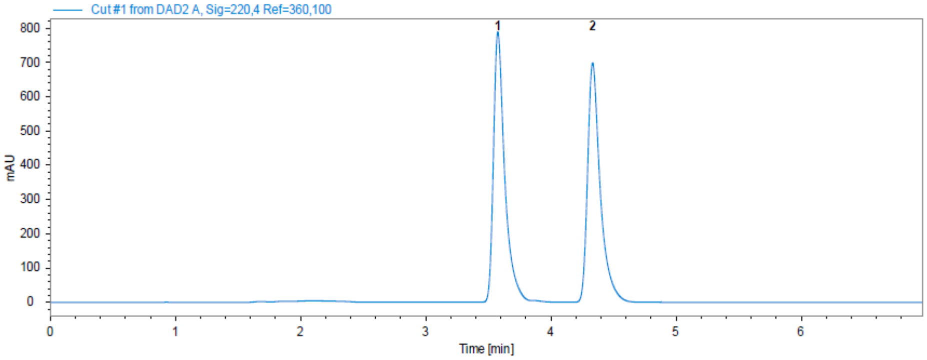

Signal: DAD2 A, Sig=220,4 Ref=360,100

Compound	Cut	Ret.Time	Area	Width	Height	Symmetry
1	1	3.578	4977.514<br>**4977.514**	0.094	785.928	0.592
2	1	4.335	4913.223<br>**4913.223**	0.104	698.627	0.617

Agilent

2D-LC Results Report

Cut# : 2

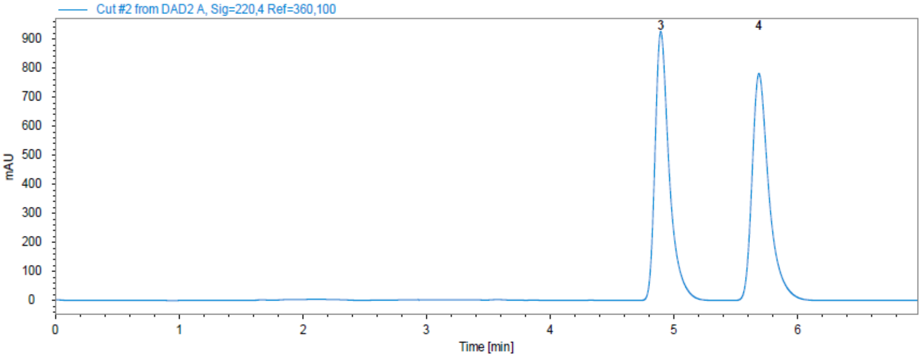

Signal: DAD2 A, Sig=220,4 Ref=360,100

Compound	Cut	Ret.Time	Area	Width	Height	Symmetry
3	2	4.893	7401.426<br>**7401.426**	0.119	925.171	0.627
4	2	5.687	7374.206<br>**7374.206**	0.142	781.043	0.646

HPLC Trace of Enantiopure 2i and 2i':

Agilent

2D-LC Results Report

**Data File:**	D:\Data\Kundendaten\Das\DAT-SC-640-01003.D
**Sample Name:**	DAT-SC-640-01
**Description:**	2 µl DAT-SC-640-01 (in 2-Propanol)<br>50 mm Eclipse Plus C18 1.8 µm, 4.6 mm i.D.<br>Methanol / Wasser-Gradient:<br>60% - 5' - 90% B<br>1.0 ml/min, 45.3 MPa, 308 K<br>UV 220 nm<br><br>Heart cut time based 3.26 min + 3.45 min<br><br>2. Dimension:<br>150 mm Chiralcel OJ-3R, 4.6 mm i.D.,<br>Acetonitril / Wasser = 70:30<br>1.0 ml/min, 20.0 MPa, 298 K<br>UV, 220 nm

**Instrument:**	2D-LC ohne FS	**Location:**	P1-D-02
**Injection date:**	13-Mar-19, 11:56:09	**Injection volume:**	2.00
**Acq. method:**	Das.M	**Acq. operator:**	SYSTEM

¹D chromatogram(s)

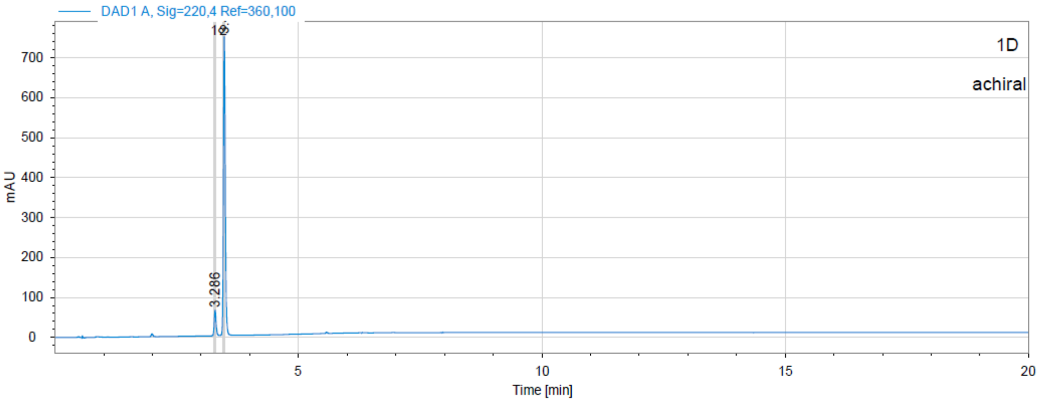

Sampling table (¹D)

Cut group	Cut #	¹D Cut start [min]	¹D Ret. time [min]	¹D Duration [min]	Trigger	²D Run start [min]	
	1	3.26	3.286	0.04	Time	3.31	1. Diastereomer
	2	3.45	3.476	0.04	Time	12.61	2. Diastereomer

Component table

Signal: DAD2 A, Sig=220,4 Ref=360,100

Component	¹D Sampling range [min]	Ret.Time ²D [min]	Area	Area%		
1	3.26 - 3.30	3.577	638.972	8.606	E1/D1	ee = 88.3%
2	3.26 - 3.30	4.333	39.569	0.533	E2/D1	
3	3.45 - 3.49	4.895	6374.210	85.849	E1/D2	ee = 89.0%
4	3.45 - 3.49	5.692	372.163	5.012	E2/D2	

2D-LC Results Report

Cut# : 1

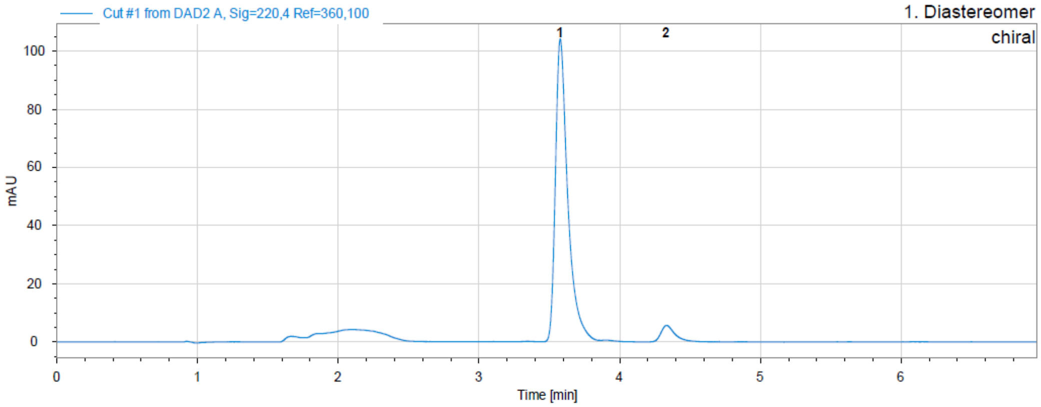

Signal: DAD2 A, Sig=220,4 Ref=360,100

Compound	Cut	Ret.Time	Area	Width	Height	Symmetry	
1	1	3.577	638.972<br>**638.972**	0.091	104.103	0.582	1. Enantiomer
2	1	4.333	39.569<br>**39.569**	0.100	5.653	0.602	2. Enantiomer

2D-LC Results Report

Cut# : 2

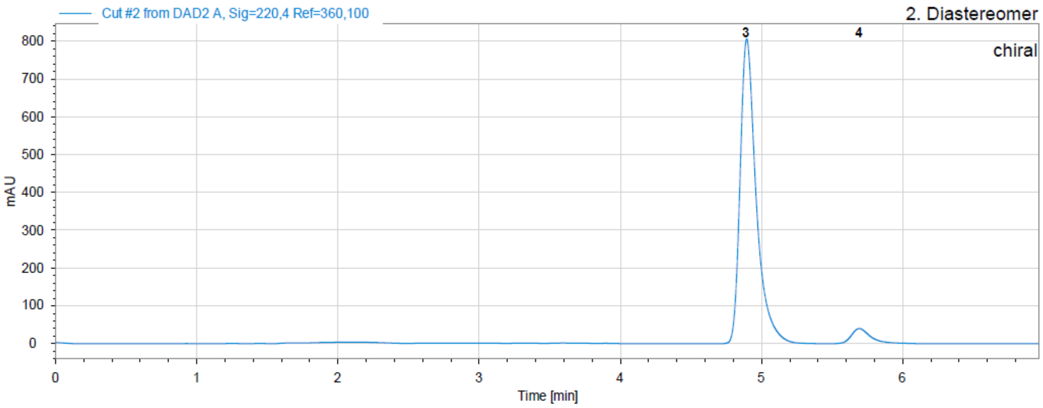

Signal: DAD2 A, Sig=220,4 Ref=360,100

Compound	Cut	Ret.Time	Area	Width	Height	Symmetry	
3	2	4.895	6374.210<br>**6374.210**	0.119	805.911	0.631	1. Enantiomer
4	2	5.692	372.163<br>**372.163**	0.140	39.702	0.654	2. Enantiomer

HPLC Trace of Racemic 2j':

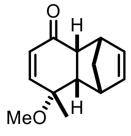

2j'

Datafile Name:GHS-GA-435.lcd
Sample Name:GHS-GA-435
Sample ID:GHS-GA-435

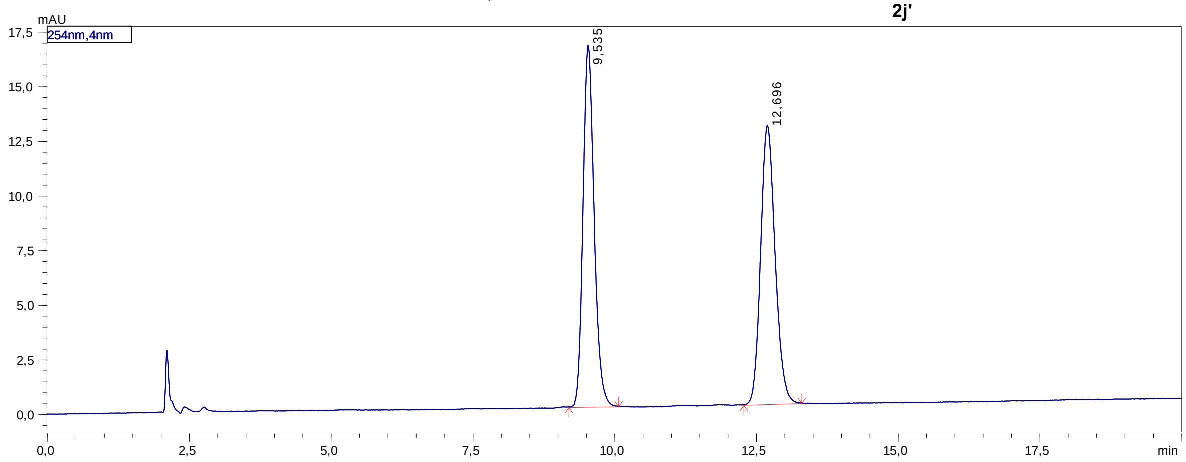

Peak#	Ret. Time	Area%
1	9.535	49.956
2	12.696	50.044
Total		100.00

HPLC Trace of Enantiopure 2j':

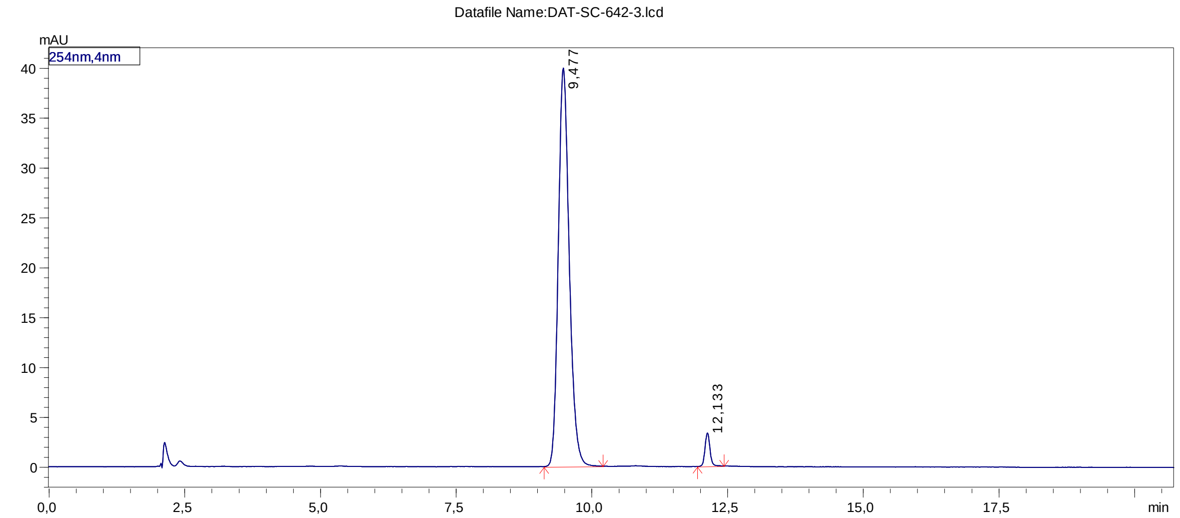

Peak#	Ret. Time	Area%
1	9.477	96.317
2	12.133	3.683
Total		100.00

HPLC Trace of Racemic 2k':

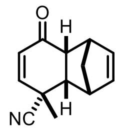

2k'

Datafile Name:GHS-GA-463.lcd
Sample Name:GHS-GA-462
Sample ID:GHS-GA-462

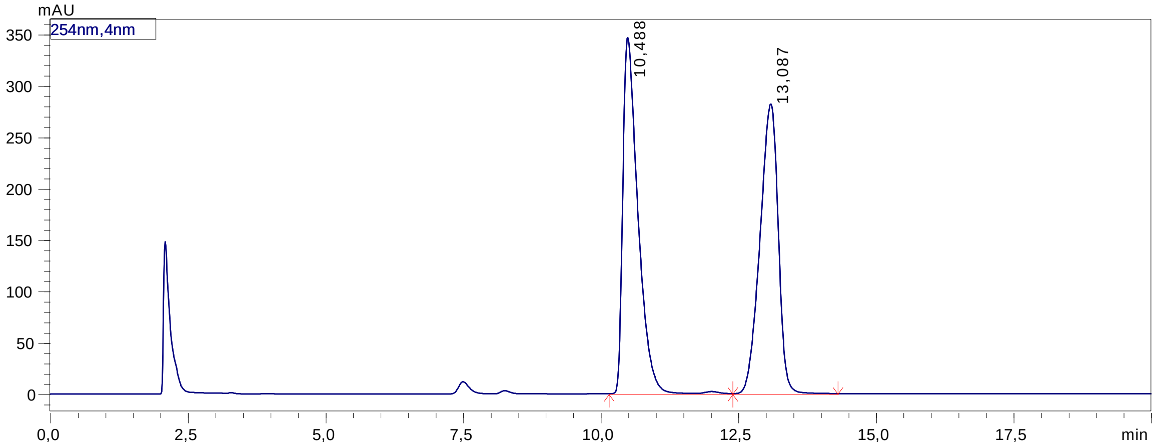

Peak#	Ret. Time	Area%
1	10.488	50.543
2	13.087	49.457
Total		100.00

HPLC Trace of Enantiopure 2k':

Datafile Name:GHS-GA-606_IC-3_10%_1mL002.lcd
Sample Name:GHS-GA-606
Sample ID:GHS-GA-606

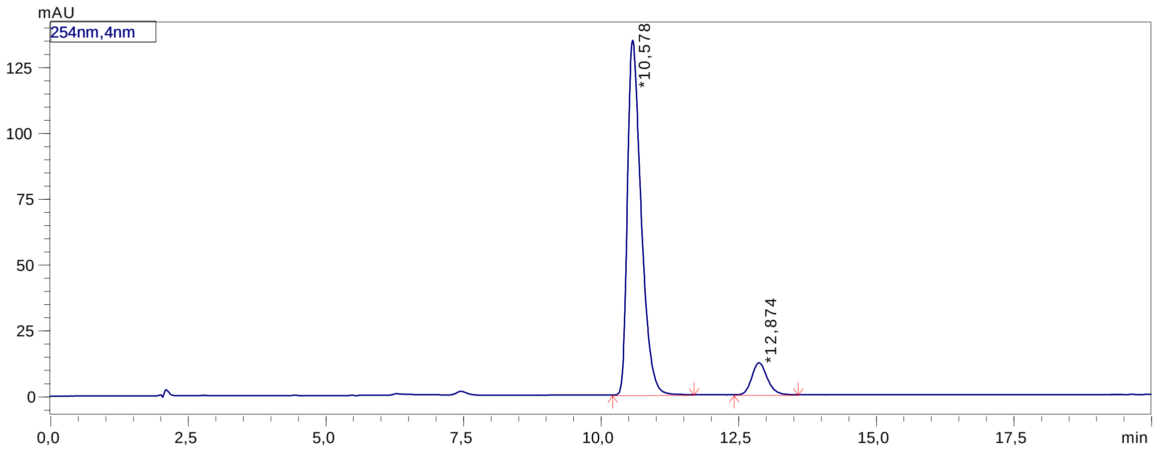

Peak#	Ret. Time	Area%
1	10.578	90.765
2	12.874	9.235
Total		100.00

HPLC Trace of Racemic 2l':

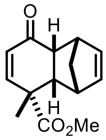

2l'

Datafile Name:GHS-GA-488.lcd
Sample Name:GHS-GA-488
Sample ID:GHS-GA-488

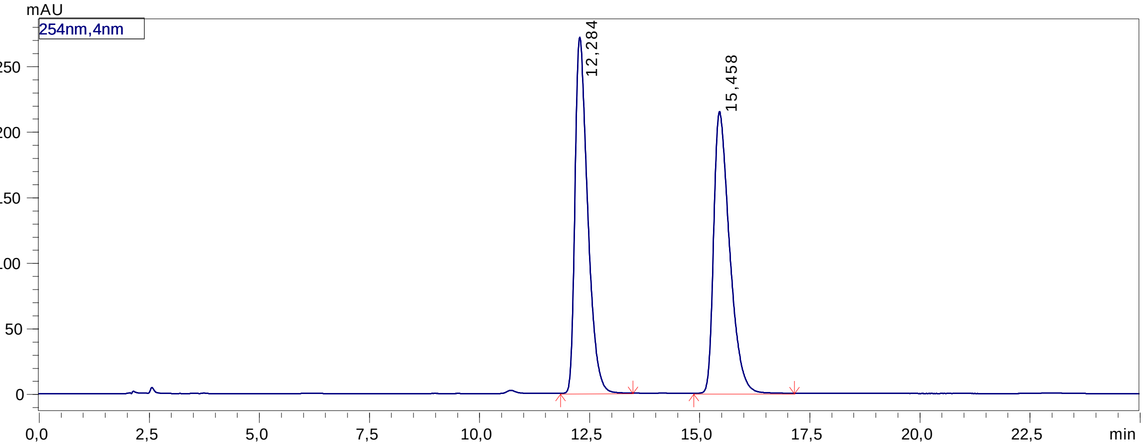

Peak#	Ret. Time	Area%
1	12.284	50.167
2	15.458	49.833
Total		100.00

HPLC Trace of Enantiopure 2l':

Datafile Name:GHS-GA-607_IC-3_5%_1mL002.lcd
Sample Name:GHS-GA-607
Sample ID:GHS-GA-607

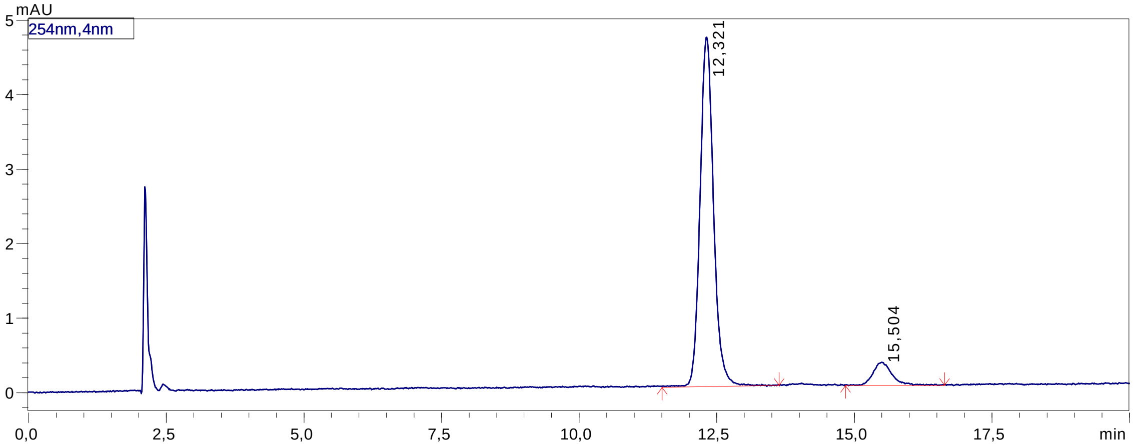

Peak#	Ret. Time	Area%
1	12.321	92.278
2	15.504	7.722
Total		100.00

HPLC Trace of Racemic 2jj and 2jj':

Agilent

2D-LC Results Report

Data File:	D:\Data\Kundendaten\Ghosh\GHS-GA-459-01003.D
Sample Name:	GHS-GA-459-01
Description:	1 µl GHS-GA-459-01<br>50 µL 2-Propanol in 150 µL Acetonitril<br>50 mm Eclipse Plus C18 1.8 µm, 4.6 mm i.D.<br>Methanol/Wasser-Gradient:<br>50% - 5' - 80% B<br>1.0 ml/min, 48.9 MPa, 308 K<br>UV 220 nm<br><br>HC peak based 150 mAU's<br>6x flush volume<br><br>2. Dimension:<br>150 mm 3-AmyCoat RP, 4.6 mm i.D., Säule 6<br>Acetonitril / Wasser-Gradient:<br>30% - 10' - 50% B<br>1.0 ml/min, 24.5 MPa, 298 K<br>UV, 220 nm

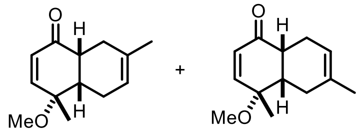

2jj (major) **2jj'** (minor)

Instrument:	2D-LC ohne FS	Location:	P1-D-01
Injection date:	01-Apr-19, 12:14:07	Injection volume:	1.00
Acq. method:	Gosh-HC.M	Acq. operator:	SYSTEM

¹D chromatogram(s)

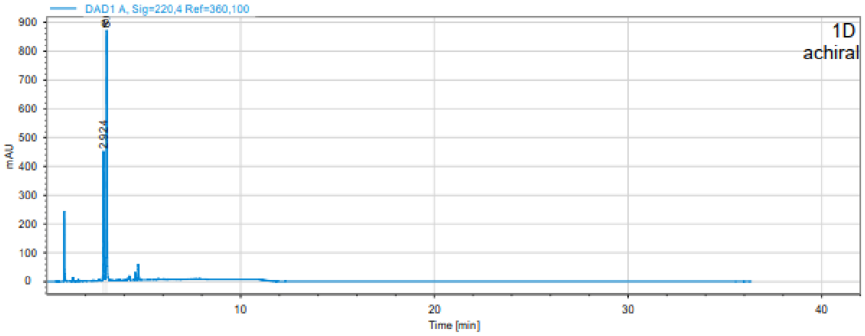

Sampling table (¹D)

Cut group	Cut #	¹D Cut start [min]	¹D Ret. time [min]	¹D Duration [min]	Trigger	²D Run start [min]	
	1	2.90	2.924	0.04	Peak	2.95	1. regioisomer
	2	3.05	3.081	0.04	Peak	27.25	2. regioisomer

Component table

Signal: DAD2 A, Sig=220,4 Ref=360,100

Component	¹D Sampling range [min]	Ret.Time ²D [min]	Area	Area%	
1	2.90 - 2.94	7.498	2565.488	19.738	E1/R1
2	2.90 - 2.94	8.788	2519.701	19.386	E2/R1
3	3.05 - 3.09	8.703	3979.531	30.617	E1/R2
4	3.05 - 3.09	9.874	3932.873	30.258	E2/R2

Printed 01.04.2019 13:48 Page 1

Agilent

2D-LC Results Report

Cut# : 1

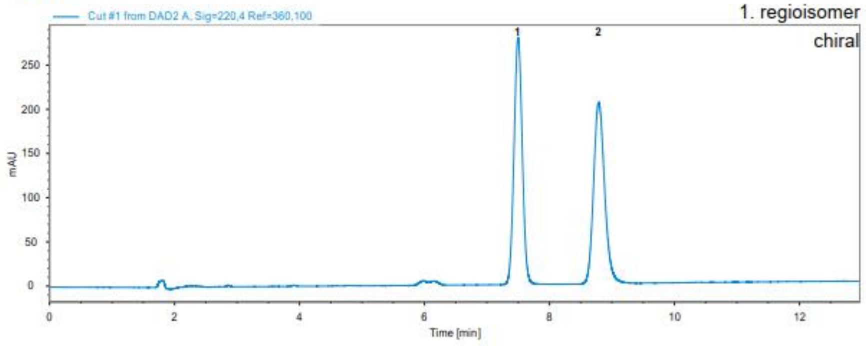

Signal: DAD2 A, Sig=220,4 Ref=360,100

Compound	Cut	Ret.Time	Area	Width	Height	Symmetry
1	1	7.498	2565.488<br>**2565.488**	0.143	279.430	0.954
2	1	8.788	2519.701<br>**2519.701**	0.188	205.308	0.822

Agilent

2D-LC Results Report

Cut# : 2

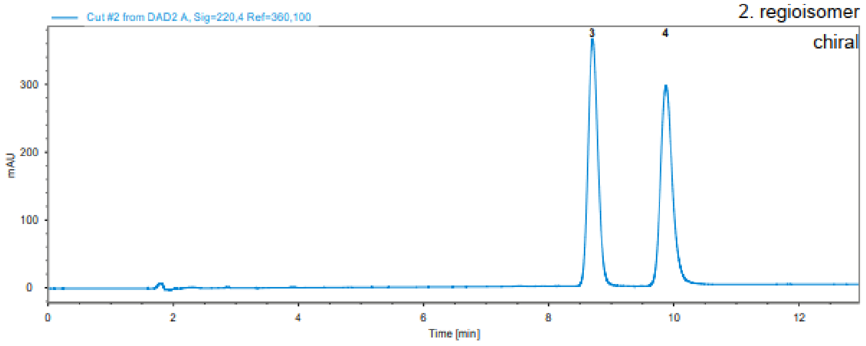

Signal: DAD2 A, Sig=220,4 Ref=360,100

Compound	Cut	Ret.Time	Area	Width	Height	Symmetry
3	2	8.703	3979.531<br>**3979.531**	0.170	364.983	0.842
4	2	9.874	3932.873<br>**3932.873**	0.206	294.885	0.760

HPLC Trace of Enantiopure 2jj and 2jj':

Agilent

2D-LC Results Report

Data File:	D:\Data\Kundendaten\Ghosh\GHS-GA-510-B003.D
Sample Name:	GHS-GA-510-B
Description:	1 µl GHS-GA-510-B<br>50 µL 2-Propanol SL in 150 µL Acetonitril<br>50 mm Eclipse Plus C18 1.8 µm, 4.6 mm i.D.<br>Methanol/Wasser-Gradient:<br>50% - 5' - 80% B<br>1.0 ml/min, 49.4 MPa, 308 K<br>UV 220 nm<br><br>HC peak based 100 mAU's<br>6x flush volume<br><br>2. Dimension:<br>150 mm 3-AmyCoat RP, 4.6 mm i.D., Säule 6<br>Acetonitril / Wasser-Gradient:<br>30% - 10' - 50% B<br>1.0 ml/min, 24.8 MPa, 298 K<br>UV, 220 nm

Instrument:	2D-LC ohne FS	Location:	P1-E-02
Injection date:	16-Apr-19, 10:50:22	Injection volume:	1.00
Acq. method:	Gosh-HC.M	Acq. operator:	SYSTEM

¹D chromatogram(s)

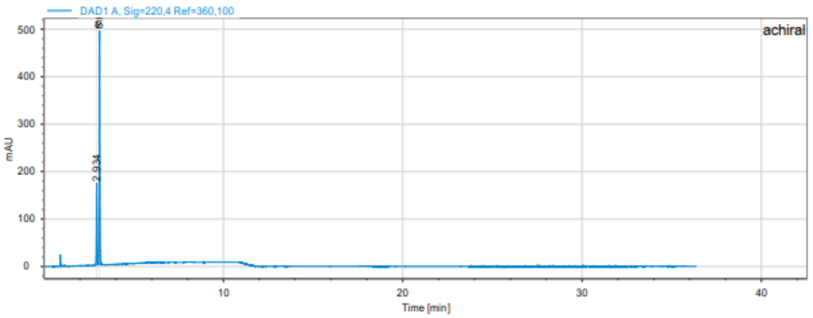

Sampling table (¹D)

Cut group	Cut #	¹D Cut start [min]	¹D Ret. time [min]	¹D Duration [min]	Trigger	²D Run start [min]	
	1	2.92	2.934	0.04	Peak	2.97	1. Diastereomer
	2	3.06	3.091	0.04	Peak	27.27	2. Diastereomer

Component table

Signal: DAD2 A, Sig=220,4 Ref=360,100

Component	¹D Sampling range [min]	Ret.Time ²D [min]	Area	Area%		
1	2.92 - 2.96	7.438	1342.253	20.423	E1/D1	ee = 34.0%
2	2.92 - 2.96	8.792	660.429	10.049	E2/D1	
3	3.06 - 3.10	8.621	3712.936	56.494	E1/D2	ee = 62.5%
4	3.06 - 3.10	9.898	856.592	13.034	E2/D2	

Printed 16.04.2019 11:34 Page 1

Peak#	Ret. Time	Area%	Name
1	7.438	20.423	E1/R2
2	8.621	56.494	E1/R1
3	8.792	10.049	E2/R2
4	9.898	13.034	E2/R1
Total		100.00	

R1(major regioisomer):81:19 er
R2(minor regioisomer):67:33 er

Agilent

2D-LC Results Report

Cut# : 1

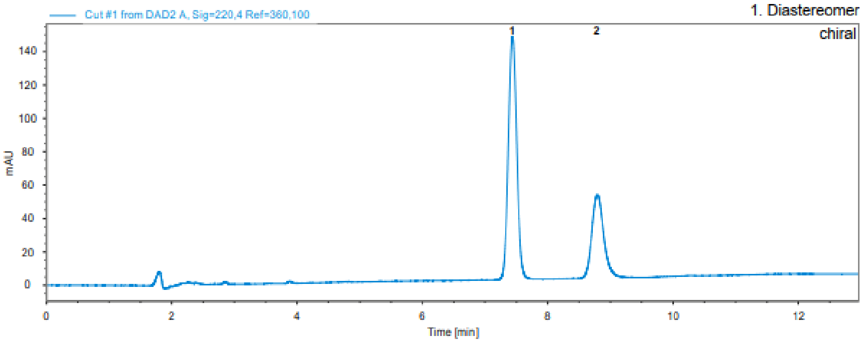

Signal: DAD2 A, Sig=220,4 Ref=360,100

Compound	Cut	Ret.Time	Area	Width	Height	Symmetry		
1	1	7.438	1342.253 **1342.253**	0.143	145.746	0.927	1. Enantiomer	ee = 34.0%
2	1	8.792	660.429 **660.429**	0.199	50.034	0.812	2. Enantiomer	

Agilent

2D-LC Results Report

Cut# : 2

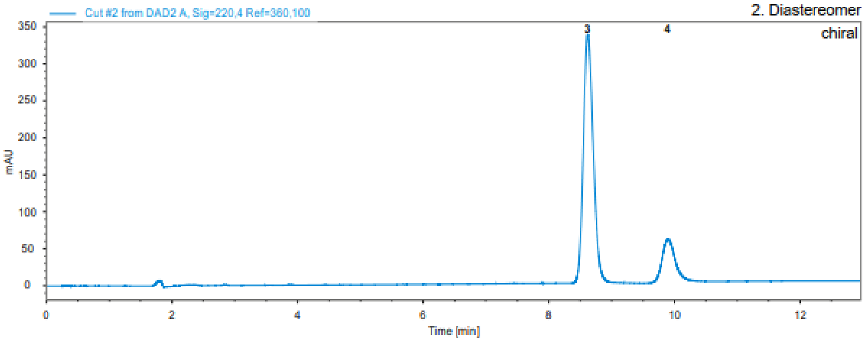

Signal: DAD2 A, Sig=220,4 Ref=360,100

Compound	Cut	Ret.Time	Area	Width	Height	Symmetry		
3	2	8.621	3712.936 **3712.936**	0.170	336.239	0.795	1. Enantiomer	ee = 62.5%
4	2	9.898	856.592 **856.592**	0.217	58.550	0.757	2. Enantiomer	

HPLC Trace of Racemic 7:

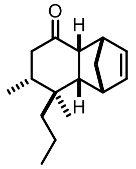

7

Datafile Name:GHS-GA-646relC3-99-1-1.4.lcd
Sample Name:GHS-GA-647re
Sample ID:GHS-GA-647re

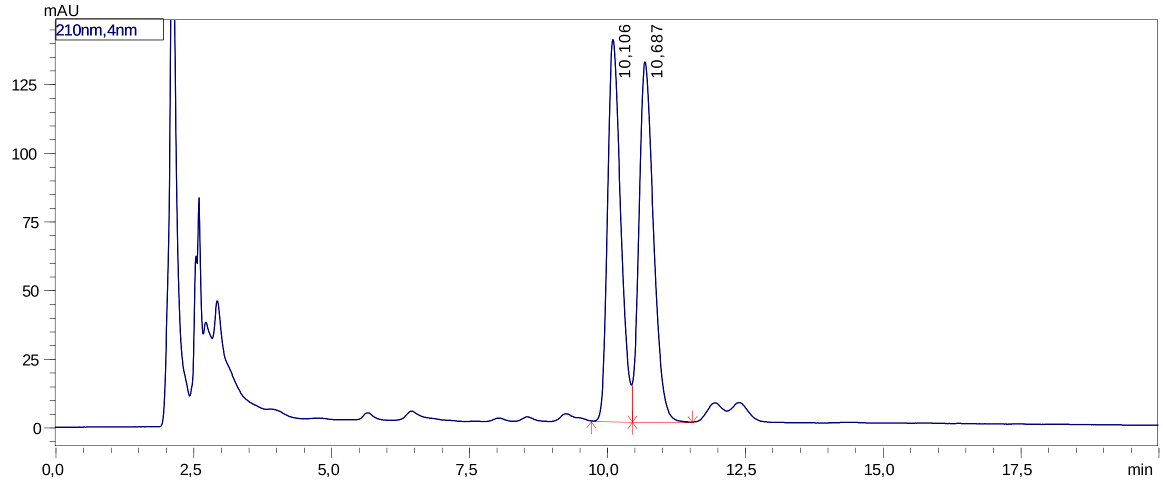

Peak#	Ret. Time	Area%
1	10.106	49.059
2	10.687	50.941
Total		100.00

HPLC Trace of Enantiopure 7:

Datafile Name:GHS-GA-658-IC3-99-1-1.4.lcd
Sample Name:GHS-GA-658
Sample ID:GHS-GA-658

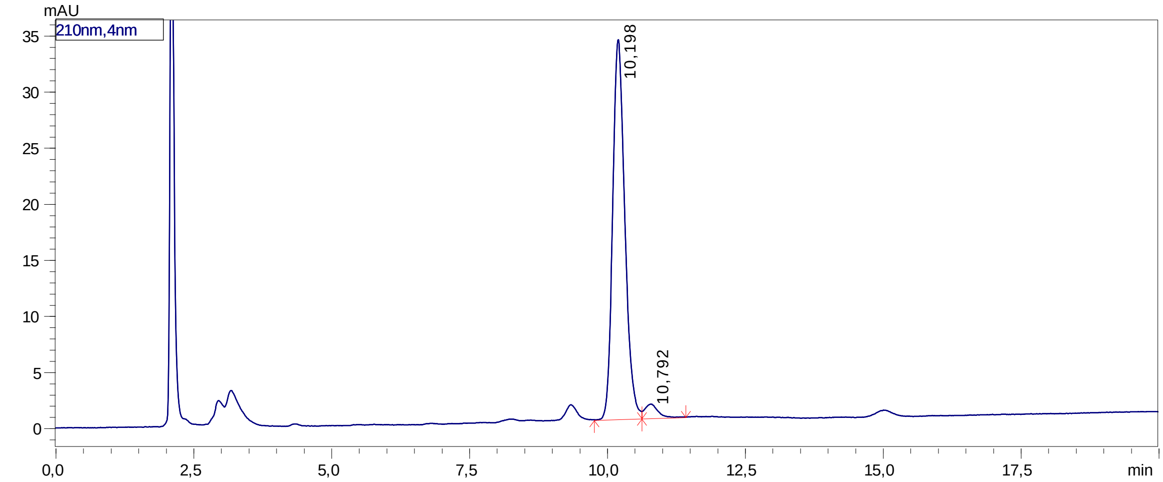

Peak#	Ret. Time	Area%
1	10.198	96.209
2	10.792	3.791
Total		100.00

GC Trace of Racemic 8:

Page 1-1

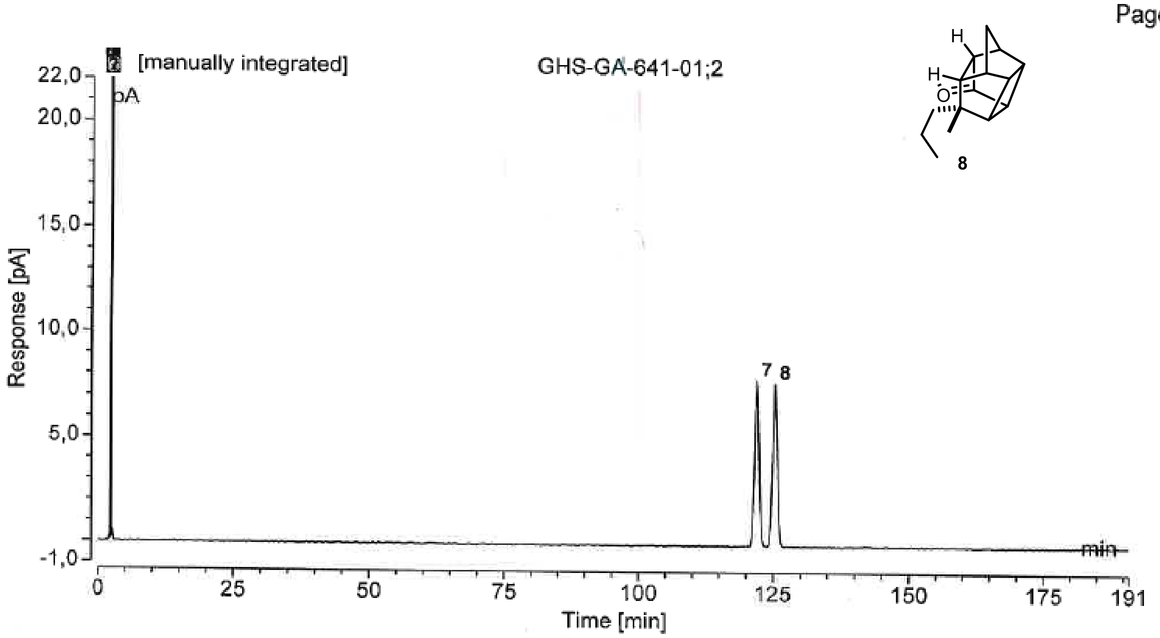

Sample:	GHS-GA-641-01;2	Instrument:	GC_313
Sequenz:	6651 GHS-GA PH	Measured:	20.08.19 10:10
Sequenz date:	19.08.19	Processing M.:	GHS-GA-641 raz.
		Report-File:	Verhältnis

Razemattrennung

No.	Ret.Time min	Rel.Area %	Peak Name
7	121,98	49,95	.
8	125,31	50,05	.

Instrument parameters:

Column:	30,0 m	BGB-176 0,25/0,25df G/618
Temperature:	220 / 120 iso / 350	
Gas:	0,50 bar	H2
Sample size:	0,2 µL	

HPLC Trace of Enantiopure 8:

Page 1-1

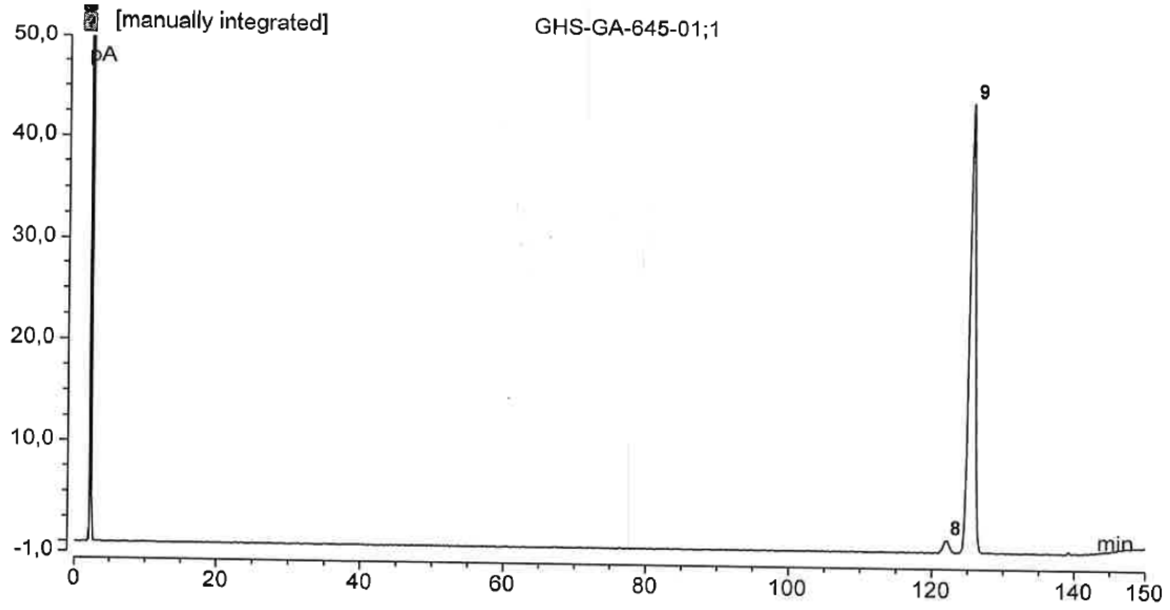

Sample:	**GHS-GA-645-01;1**	Instrument:	**GC_313**
Sequenz:	**6660 GHS-GA PH**	Measured:	**21.08.19 12:17**
Sequenz date:	**21.08.19**	Processing M.:	GHS-GA-641 raz.
		Report-File:	645-01 ee

ee-Verhältnis

No.	Ret.Time min	Rel.Area %	Peak Name
8	122,03	2,67	.
9	125,60	97,33	.

Instrument parameters:

Column:	30,0 m	
Temperature:	220 / 120, 130 min iso 6/min 220, 3 min iso / 350	
Gas:	0,50 bar	H2
Sample size:	1,0 µL	

HPLC Trace of Racemic 16:

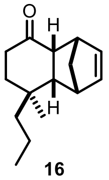

16

Datafile Name:GHS-GA-642AlC3-95-5-1.2.lcd
Sample Name:GHS-GA-642
Sample ID:GHS-GA-642

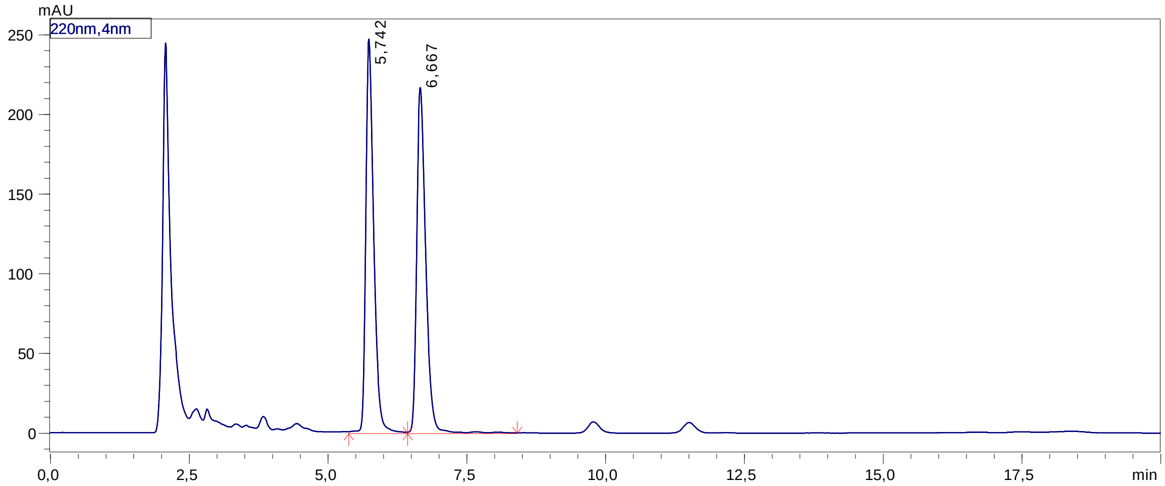

Peak#	Ret. Time	Area%
1	5.742	50.188
2	6.667	49.812
Total		100.00

HPLC Trace of Enantiopure 16:

Datafile Name:GHS-GA-643AlC3-95-5-1.3.lcd
Sample Name:GHS-GA-642
Sample ID:GHS-GA-642

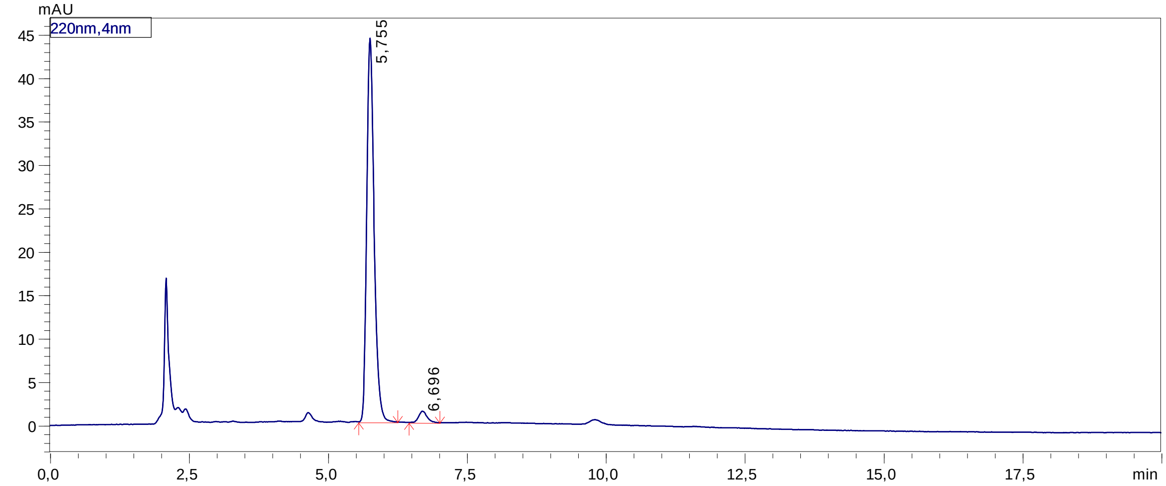

Peak#	Ret. Time	Area%
1	5.755	96.791
2	6.696	3.209
Total		100.00

GC Trace of Racemic 9b:

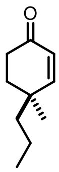

9b

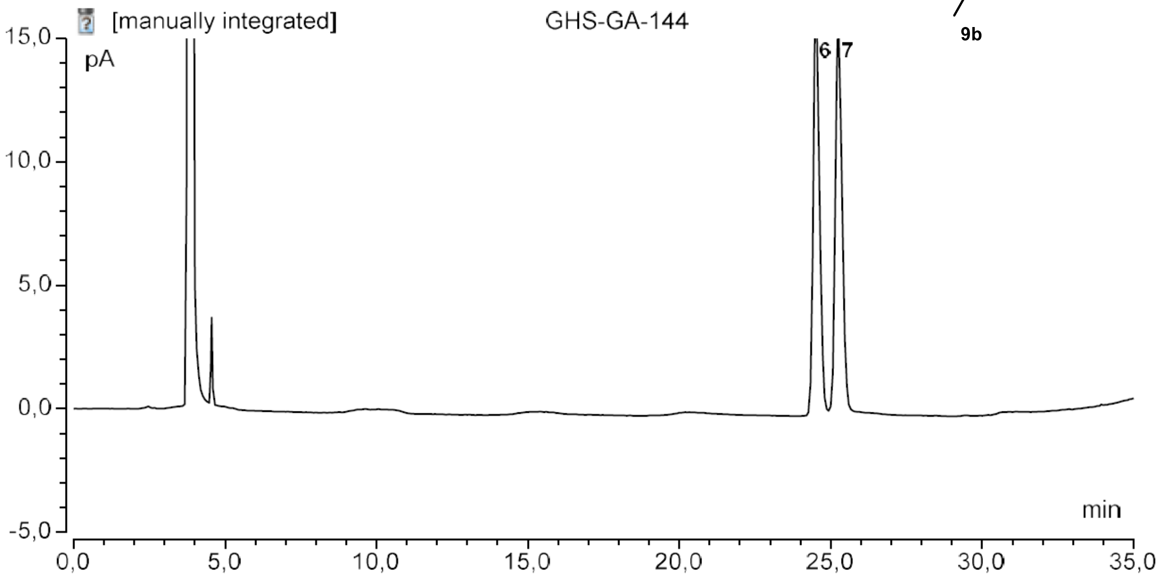

Sample:	**GHS-GA-144**	Instrument:	**GC_Front**
Sequenz:	**Robinson annulation**	Measured:	**02.08.19 11:52**
Sequenz date:	**02.08.19**	Processing M.:	MPI
		Report-File:	GC1_peak ratio

No.	Ret.Time min	Rel.Area %	Peak Name
6	24,51	49,43	Component 1
7	25,25	50,57	Component 2

Instrument parameters:

Column:	30 m	Cyclosil B
Temperature:	220/135 30min iso 12/min 220 5min iso/350	
Gas:	0,50 bar	H2
Sample size:	1,0 µL	

GC Trace of Enantiopure 9b:

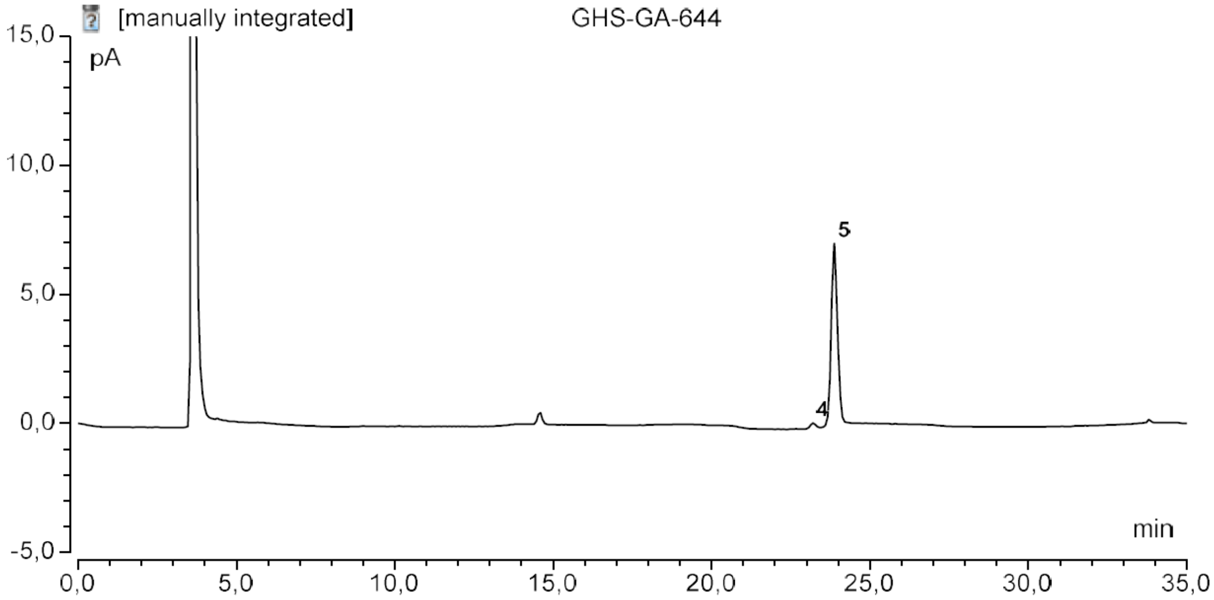

Sample:	**GHS-GA-644**	Instrument:	**GC_Front**
Sequenz:	**Robinson annulation**	Measured:	**16.08.19 14:13**
Sequenz date:	**02.08.19**	Processing M.:	MPI
		Report-File:	GC1_peak ratio

No.	Ret.Time min	Rel.Area %	Peak Name
4	23,20	2,33	Component 3
5	23,87	97,67	Component 6

Instrument parameters:

Column:	30 m	Cyclosil B
Temperature:	220/135 30min iso 12/min 220 5min iso/350	
Gas:	0,50 bar	H2
Sample size:	1,0 µL	

HPLC Trace of Racemic 10:

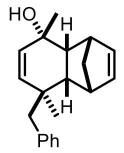

10

Datafile Name:GHS-GA-578cAS3- 97-3.1_0.5.lcd
Sample Name:GHS-GA-578crr
Sample ID:GHS-GA-578crr

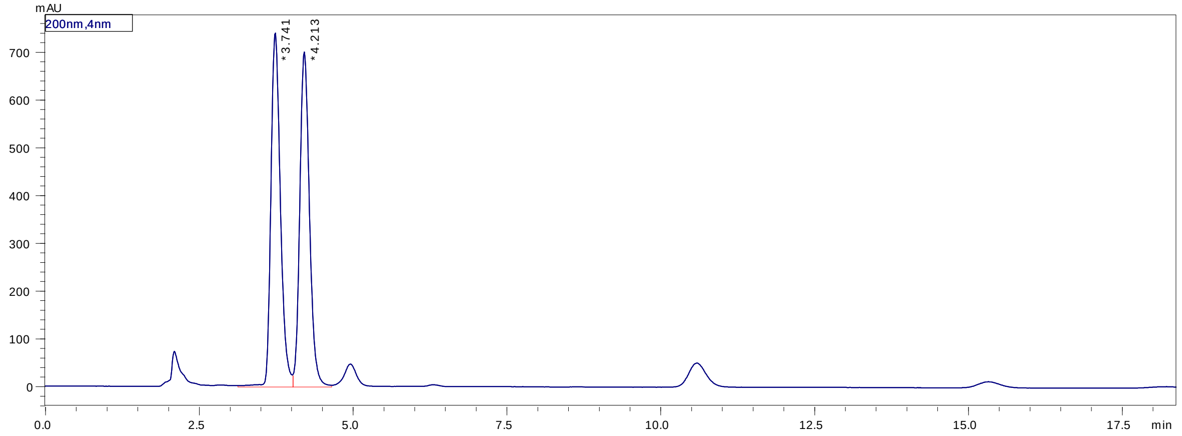

Peak#	Ret. Time	Area%
1	3.741	50.934
2	4.213	49.066
Total		100.00

HPLC Trace of Enantiopure 10:

Datafile Name:GHS-GA-592prAS3- 97-3.1_0.5.lcd
Sample Name:GHS-GA-592p
Sample ID:GHS-GA-592p

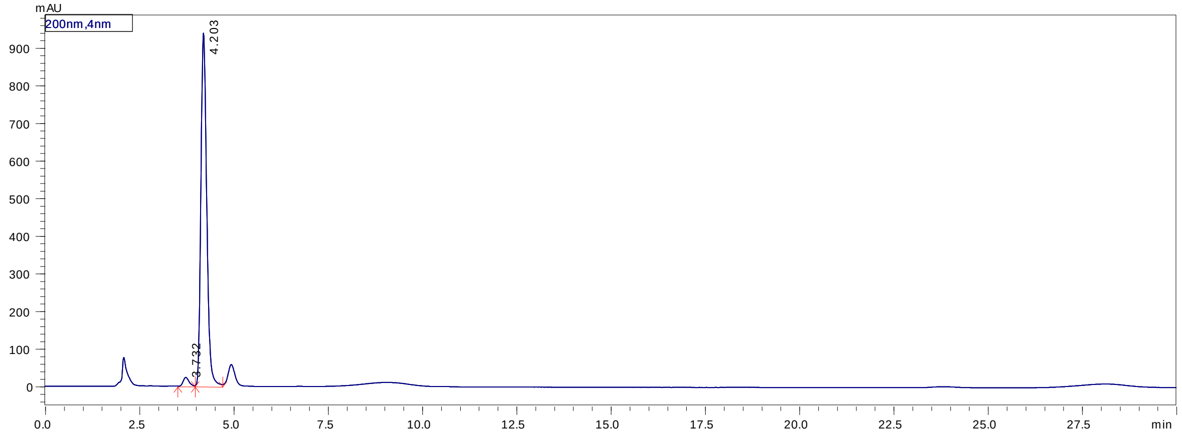

Peak#	Ret. Time	Area%
1	3.732	2.544
2	4.203	97.456
Total		100.00

HPLC Trace of Racemic 11:

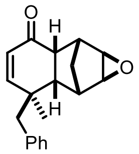

11

Datafile Name:GHS-GA-589 IA-3 95 5.1.lcd
Sample Name:GHS-GA-589
Sample ID:GHS-GA-589

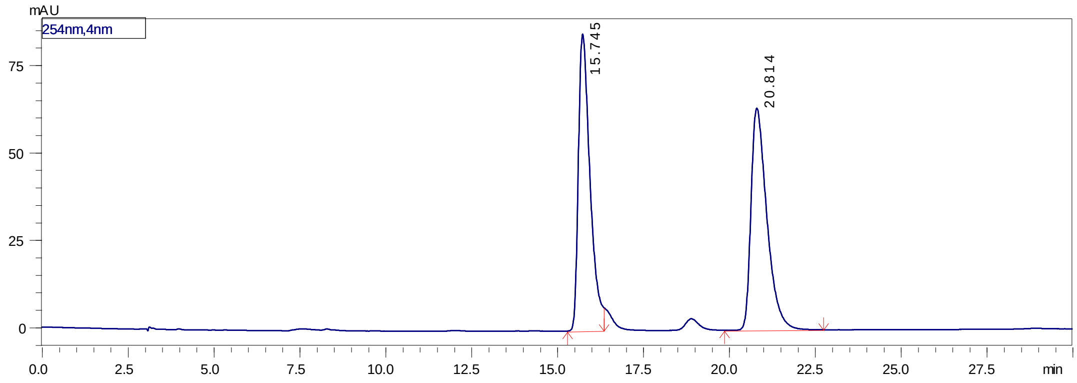

Peak#	Ret. Time	Area%
1	15.745	49.603
4	20.814	50.397
Total		100.00

HPLC Trace of Enantiopure 11:

Datafile Name:GHS-GA-593cIA- 95-5.1_0.5.lcd
Sample Name:GHS-GA-593c
Sample ID:GHS-GA-593c

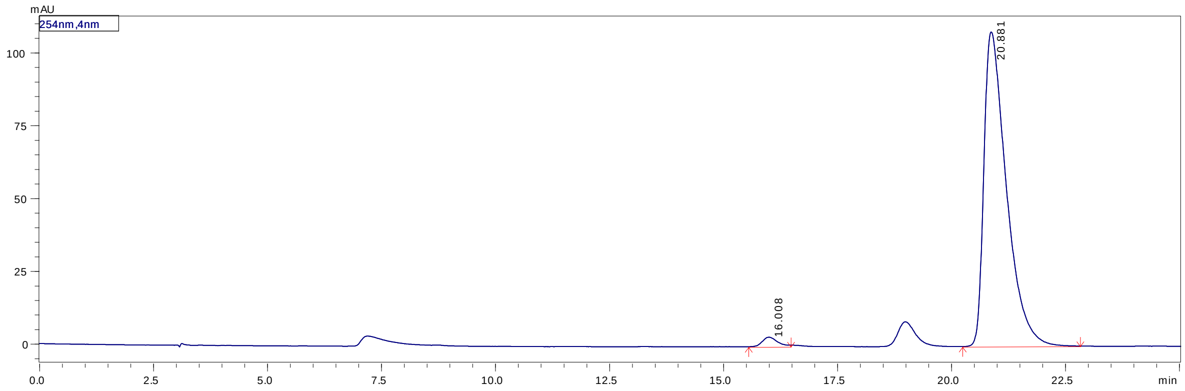

Peak#	Ret. Time	Area%
1	16.008	1.997
2	20.881	98.003
Total		100.00

HPLC Trace of Racemic 12:

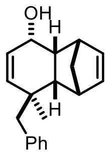

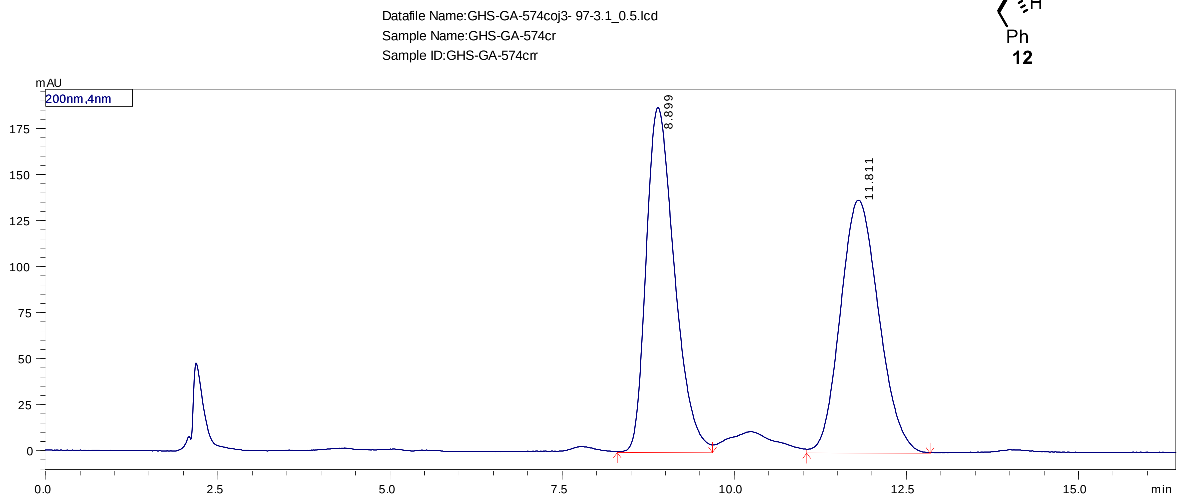

Peak#	Ret. Time	Area%
1	8.889	50.139
2	11.811	49.861
Total		100.00

HPLC Trace of Enantiopure 12:

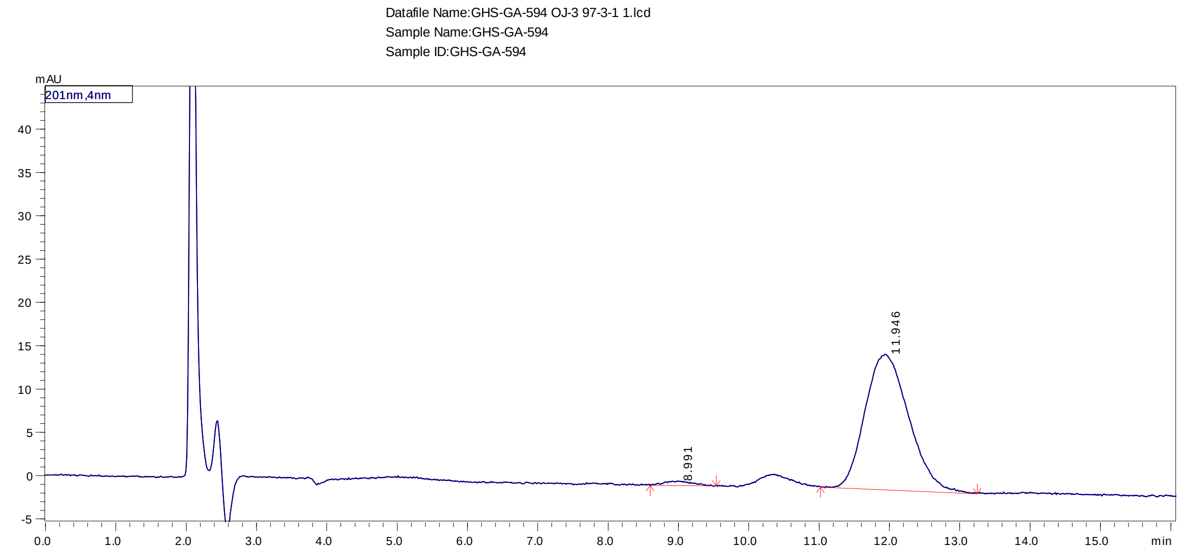

Peak#	Ret. Time	Area%
1	8.991	1.539
2	11.946	98.461
Total		100.00
